# Predicting mid-life capital formation with pre-school delay of gratification and life-course measures of self-regulation ^☆^

**DOI:** 10.1016/j.jebo.2019.08.016

**Published:** 2019-11-04

**Authors:** Daniel J. Benjamin, David Laibson, Walter Mischel, Philip K. Peake, Yuichi Shoda, Alexandra Steiny Wellsjo, Nicole L. Wilson

**Affiliations:** a Center for Economic and Social Research and Department of Economics, University of Southern California, Los Angeles, CA 90089, United States; b National Bureau of Economic Research, Cambridge, MA 02138, United States; c Department of Economics, Harvard University, Cambridge, MA 02138, United States; d Department of Psychology, Columbia University, New York, NY 10027, United States; e Department of Psychology, Smith College, Northampton, MA 01063, United States; f Department of Psychology, University of Washington, Seattle, WA 98195, United States; g Department of Economics, University of California, Berkeley, CA 94720, United States; h Department of Management, Lundquist College of Business, University of Oregon, Eugene, OR 97403, United States

**Keywords:** D910, D140, I310, I210, I120, Self-regulation, Delay of gratification, Mid-life capital formation

## Abstract

How well do pre-school delay of gratification and life-course measures of self-regulation predict mid-life capital formation? We surveyed 113 participants of the 1967–1973 Bing pre-school studies on delay of gratification when they were in their late 40’s. They reported 11 mid-life capital formation outcomes, including net worth, permanent income, absence of high-interest debt, forward-looking behaviors, and educational attainment. To address multiple hypothesis testing and our small sample, we pre-registered an analysis plan of well-powered tests. As predicted, a newly constructed and pre-registered measure derived from preschool delay of gratification does not predict the 11 capital formation variables (i.e., the sign-adjusted average correlation was 0.02). A pre-registered composite self-regulation index, combining preschool delay of gratification with survey measures of self-regulation collected at ages 17, 27, and 37, does predict 10 of the 11 capital formation variables in the expected direction, with an average correlation of 0.19. The inclusion of the preschool delay of gratification measure in this composite index does not affect the index’s predictive power. We tested several hypothesized reasons that preschool delay of gratification does not have predictive power for our mid-life capital formation variables.

## 1. Introduction

The capacity to self-regulate matters for a wide array of life outcomes. A critical component of self-regulation is the ability to delay gratification. Working at Stanford’s Bing Nursery School beginning in the late 1960’s, Mischel and colleagues conducted numerous experimental variations of the now classic “self-imposed delay of gratification” paradigm to examine the cognitive and contextual mechanisms that affect preschoolers’ ability to wait to obtain a more desired outcome (Mischel et al., 1989; Mischel, 2014). Follow-up investigations beginning in the 1980’s reported that preschool waiting time predicted better self-regulation as teenagers, especially among children who participated in certain experimental variations of the preschool delay task (Shoda et al., 1990). These “diagnostic” experimental conditions were challenging in that they combined the physical presence of the tempting outcomes with requiring children to rely on their own spontaneously generated strategies for coping with the frustration of waiting (Shoda et al., 1990). Longitudinal research with the Bing cohort has evolved into a multi-disciplinary exploration. Research on the Bing cohort now spans roughly 50 years, making it the longest study of the delay of gratification paradigm.^1^ Outcomes that have been studied include coping, social, and academic competence (Mischel et al., 1988; Mischel et al., 1989; Shoda et al., 1990), SAT scores (Shoda et al., 1990), substance use (Ayduk et al., 2000), borderline personality features (Ayduk et al., 2008), BMI (Schlam et al., 2013), executive functioning, and neural activation patterns (Berman et al., 2013; Casey et al., 2011). The predictive power of the delay of gratification task has also been documented in other samples with childhood and adolescent outcomes such as BMI (Connell and Francis, 2014; Francis and Susman, 2009; Seeyave et al., 2009), ADHD symptoms (Campbell and von Stauffenberg, 2009), academic competence (Duckworth et al., 2013; Watts et al., 2018), and social competence (Yang and Wang, 2007). As one would expect, partial regression coefficients between preschool delay and subsequent life outcomes are lower when early childhood, delay-related variables such as family background and early cognitive development are added to the model as predictors (Watts et al., 2018). In previous research in the Dunedin cohort, other survey and observer-reported measures of early life self-control have been found to be predictive of indices of *adult* outcomes, including socioeconomic status, financial planfulness, and income (Moffitt et al., 2011).

The network of documented longitudinal relations of preschool delay of gratification are extensive. Nonetheless, both media and academic accounts of the research commonly exaggerate the scope of the actual findings (Watts et al., 2018). In these accounts, preschool delay is cast as prognostic of almost all adult life milestones. In light of this common miscasting, it is important that ongoing research empirically examine whether and how self-control actually relates to later life outcomes. The current research explores one such connection: the relation to mid-life capital formation.

Economists conceptualize capital formation to include any costly activity that accumulates a resource (i.e., “capital”) that generates future benefits. This definition implies that capital formation includes investments in human capital (e.g., working hard at school or on the job to obtain knowledge/experience that will pay off later), investments in social capital (e.g., developing a network of professional contacts that will increase long-run professional success), and investments in financial capital (e.g., saving now so that the household can spend more later).

In the current paper, we revisit 113 individuals from the original Bing cohort, roughly 45 years after they participated in the original experiments. Within this sample, we examine associations between measures of self-regulation based on multiple assessments during the first four decades of life (including preschool delay) and a comprehensive array of mid-life measures of capital formation. In addition, we also study preschool waiting time on its own as a predictor of mid-life capital formation. To our knowledge, ours is the first attempt to look at these relationships because no cohort for which delay of gratification was measured in childhood has become old enough to survey in mid-life until now, with the exception of the Dunedin cohort, which is roughly ten years younger than the Bing cohort which we study. In the conclusion of this paper, we compare our results to the small set of partially overlapping results from the Dunedin study and find a remarkable degree of concordance despite the starkly different socioeconomic contexts of the two studies (and the somewhat younger age of the Dunedin study participants).

The rest of the paper proceeds as follows. In Section 2 we discuss our sample. In Section 3 we describe our key measures and our pre-registered analysis plan. In Section 4 we state our pre-registered hypotheses and report our main results. In Section 5 we present additional results. In Section 6 we conclude and discuss the generalizability of our findings. The Online Appendix (OA) contains further details about sample recruitment, survey design, and analyses, as well as a number of additional and robustness analyses that we conducted.

## 2. The Bing cohort and our survey sample

The original Bing experiments were conducted during 1967–1973 (first described in Mischel and Ebbesen, 1970). They included a total of 550 students from Stanford’s Bing Nursery School, aged about 4 years old (ranging from 2 to 6). Many of the participants are children of Stanford faculty and staff.

During 2012–2013, we contacted the 156 Bing cohort participants for whom we had current contact information (28% of the 550 total), and we invited them to participate in an online survey.^2^ The survey is reproduced in the OA. 113 participants completed it (72% of those we contacted).

Table 1 shows the demographics of our survey sample, as well as summary statistics for the measures we discuss in Section 3. Survey respondents averaged 46 years of age at the time they took the survey, 37% are male, and they are on average white, married with children, well educated, and wealthy. Table 1 also compares our survey sample with the full Bing sample on several variables we have for the full sample: sex, age during the delay of gratification task, and wait time. While our survey sample has fewer males (37% versus 48%), it does not differ substantially from the full Bing cohort on the other variables.

## 3. Methods

### 3.1. Capital formation measures

From the survey responses, we constructed the following 11 capital formation measures, which we use as dependent variables in our study:

- *Net worth* is the sum of assets minus the sum of debts.
- *Permanent income* is defined as total household income (the average of reported income in the calendar year preceding the survey and the calendar year when the respondent was age 35) divided by the reported number of adults living in the household. Income at age 35 is inflation-adjusted to dollars in the year before the survey.
- *Wealth-income ratio* is net worth divided by permanent income.
- *High interest-rate debt (reverse scaled)* is the annual amount of interest above 6% paid on high-interest rate debt. For each debt category, respondents selected an interest rate bucket for each type of debt. We assumed the interest rate is the midpoint of the selected category. For each high interest-rate debt category we calculate the amount of interest above 6% as the dollar amount of debt multiplied by the interest rate above 6%.
- *Credit card misuse (reverse scaled)* is an index with four components: high interest-rate debt, amount of carried credit card debt (i.e., not paid off each month), a binary measure for having been denied a credit card in the last year, and number of late payments on a credit card bill in the last year.
- *Delay choice* is the percent of questions for which the respondent selected the “money later” instead of the “money sooner” option in 40 tradeoff questions (20 questions with a choice between money today and thirty days from today and 20 questions with a choice between money thirty and sixty days from today).
- *Savings rate* is the self-reported percentage of income saved over the past few years, including retirement accounts and all other savings.
- *Financial health* is an index comprised of three components: self-ratings about having enough money to meet needs, difficulty in paying monthly bills, and current financial situation.
- *Educational attainment (years of education)* is calculated from reported degrees received.
- *Forward-looking behaviors* is an index comprised of 5 equal-weighted components: measures of diet, exercise habits, and BMI; smoking and alcohol behavior; preventative health and dental care; procrastination; and consideration of future financial consequences.
- *Social status* is self-reported placement in society on a 10-rung ladder. The ladder is described as representing where people stand in the United States, with the top of the ladder being those who have the most money, the most education, and the most respected job.

Summary statistics for these measures are in Table 1.

We applied a rank-order inverse normal transformation to all final variables – including both the capital formation variables described above, as well as the self-regulation variables described below – because our statistical procedures have better small-sample properties when the variables are normally distributed (for discussion of this transformation, see, e.g., Bishara and Hittner, 2012). In general, the raw data for our capital formation and self-regulation variables do not appear to be normally distributed (see OA Sections I and II for distributions of the raw and transformed variables). The rank-normalization approach has the advantage that we can apply it uniformly, rather than making different parametric assumptions for each variable (e.g., that wealth is log-normally distributed). To implement the transformation, we first calculated the rank order of the subject within the sample and then used the inverse normal cdf to fit the ranks to a standard normal distribution. The transformation preserves the rank ordering of the original variables but alters their scaling and may therefore affect the correlations with other variables. However, our transformation ensures that variables are approximately normal by construction and minimizes the effects of outliers. Because the transformation relies on rank order, the variables in our analyses represent a normalized measure of relative standing.

### 3.2. Self-regulation measures

We study two measures of self-regulation. The first measure, *rank-normalized delay (RND)*, is derived from children’s waiting times observed while participating in experimental variants of the self-imposed delay of gratification paradigm when the children were in preschool. The second measure, *rank-normalized self-regulatory index (RNSRI)*, consists of four equal-weighted components, each measuring self-regulation at a different age. One of the components of RNSRI is RND. We will describe RND followed by RNSRI.

RND is a new measure derived from preschool waiting that takes into account the fact that there were diverse treatment conditions in the wait-task experiments differing in a number of ways that affected how long children on average waited in each condition (including both “diagnostic” and “non-diagnostic” conditions, as we discuss below).^3^ In addition, RND also addressed the fact that wait times are censored (the criterion for successfully waiting was typically set to 15 min). Specifically, we constructed the RND measure in three steps: (a) we generated an expected log wait time for each treatment condition using a tobit random-effects model, controlling for the child’s sex and age at the time of the experiment; (b) we calculated the difference between each child’s actual log wait time and this expected log wait time; (c) we rank-normalized these differences. We now explain steps (a) and (b) in more detail.

For step (a), we used wait-time data from nearly all the Bing participants: a total of 543 children for whom data on wait time, age at which they did the wait-time task, and sex is available. The sample size per experimental condition is 3 to 165 participants. Our survey data, which is from a subset of 113 of these participants across 20 of the 21 experimental conditions, contains 1 to 34 participants per condition. For children who participated in multiple studies at the Bing School, we use only the wait time from their first delay of gratification study (because the psychological meaning of the waiting experiences in subsequent studies may be different from their first time).

**Fig. 1.**
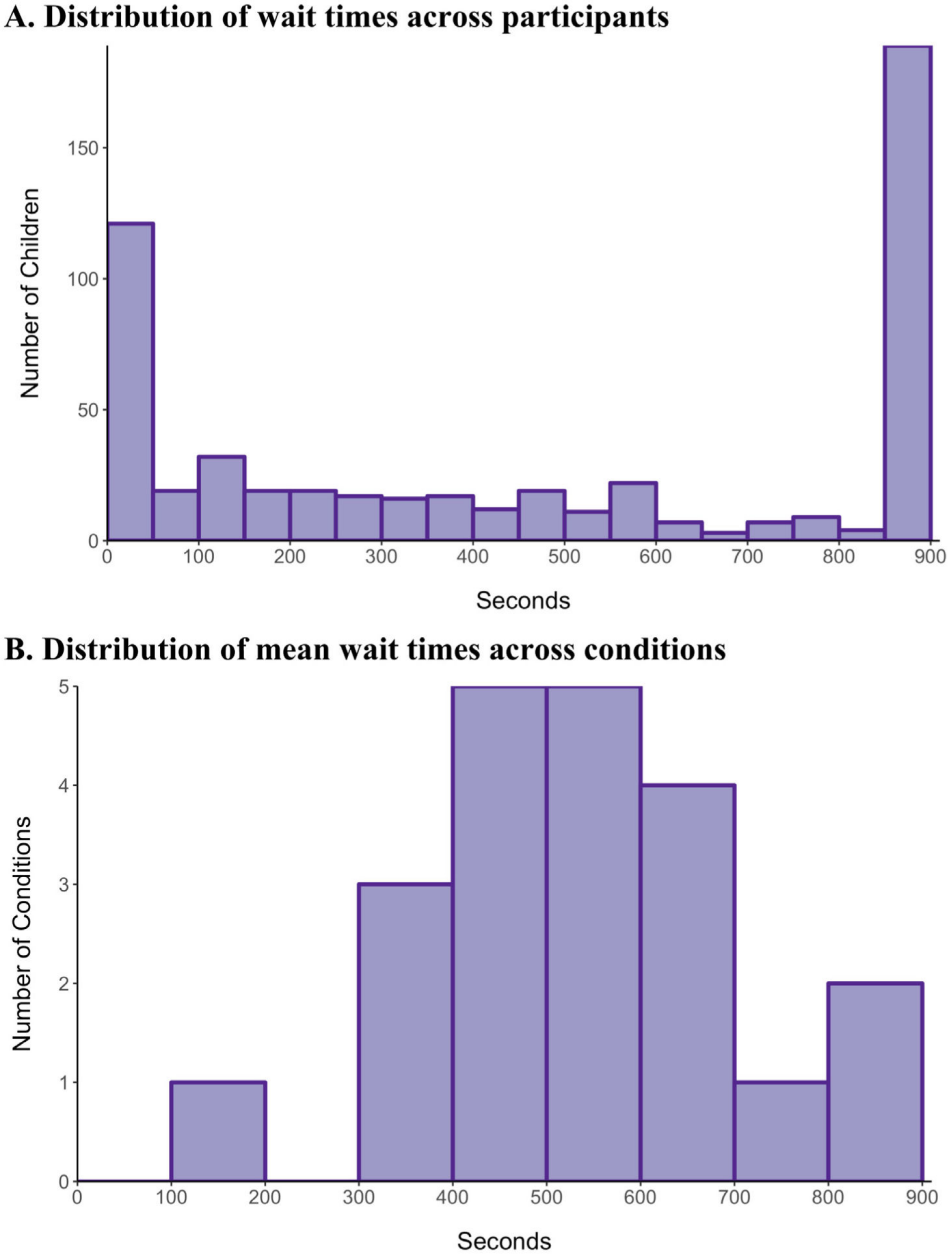
Distribution of wait times across Bing cohort participants and experimental conditions *Notes*: Distribution of wait times from the 543 children who participated in the original Bing experiments and for whom data on wait time, age at which they did the wait-time task, and sex is available. Figure 1A displays the distribution of individual wait times. Figure 1B displays the distribution of mean wait times across the 21 experimental conditions.

A histogram of the 543 raw wait times is shown in Fig. 1A. The distribution has many observations at just a few seconds, as well as a long right tail (censored at 900 s). For our model of expected wait times, we therefore adopted a log-normal distribution. Fig. 1B shows a histogram of mean raw wait times in the 21 experimental conditions. This distribution appears to have a long right tail, so we also assumed that the condition means are log-normally distributed.

We estimated the following tobit model of expected wait time:

$$y_{gi}^{*}=\alpha +\beta_{1}\times {\mathrm{age}}_{gi}+\beta_{2}\times {\mathrm{male}}_{gi}+\eta_{g}+\varepsilon_{i},$$

where *i* indexes individuals; *g* indexes experimental conditions; $y_{gi}^{*}$ is the underlying, uncensored, log wait time; *age_gi_* is age in months at time of the delay task; *male_gi_* is a binary indicator of sex; *η_g_* is the group random effect ~ *N*(0, $\sigma_{\eta }^{2}$) and *ε_gi_* is an individual-specific error ~ *N*(0, $\sigma_{\varepsilon }^{2}$). We modeled the effect of experimental condition as a random effect to deal with the very small sample sizes in some conditions. We used a tobit model to account for the maximum wait time of 900 s (or 15 min) imposed in the original experiments. The model has five parameters: a constant term *α*, the effect of being one month older *β*_1_, the effect of being male *β*_2_, the variance of the experimental-condition random effects $\sigma_{\eta }^{2}$, and the variance of the individual-specific error $\sigma_{\varepsilon }^{2}$.

We estimated the tobit random-effects model by maximum likelihood (using the GLLAMM procedure in Stata version 14.0). We calculated an empirical-Bayes estimate of the expected ln(wait time) for each participant as the expected value of the posterior distribution of the experimental condition’s random effect, treating the model’s parameter estimates as the true parameter values.

Table 2 shows the parameter estimates. The age coefficient of 0.08 (SE = 0.02) implies that, on average, every additional month of age when participating in the wait-time task is associated with waiting roughly 8% longer. This is consistent with prior work in the Bing and other samples on the development of delay of gratification strategies (Mischel and Mischel, 1983) and directionally consistent with prior results on the correlation between age and delay (e.g., Carlson et al. (2018) find a marginally significant positive correlation between wait time and age in the Bing and two other samples). The male coefficient of −0.67 (SE = 0.23) implies that, controlling for age, males on average wait less than 50% as long as females—a remarkably large difference between the sexes. Previous analyses of the Bing cohort have found mixed results with respect to gender. Mischel and Underwood (1974) do find that males on average wait less than females when the data is aggregated across experimental arms. However, other analyses of subsets of the Bing cohort have not found statistically different waiting times between boys and girls (Mischel and Moore, 1973, 1980; Mischel et al., 1988; Ayduk et al., 2000), perhaps due to the smaller sample sizes in these analyses. A recent exception is Carlson et al. (2018), who find that girls waited significantly longer than boys in the diagnostic subsample of Bing participants. In a different sample of 135 preschoolers in the 1980’s, Carlson et al. (2018) find a similar, though statistically insignificant, sex difference. In a third sample of 540 preschoolers in the 2000’s, they find no sex difference.

Turning to step (b), for each participant we calculate ln(subject’s actual wait time) – expected ln(wait time). If the participant’s wait time was at the boundary of 900 s, we used the estimated model to calculate the expected value of the uncensored wait time (conditional on the participant waiting at least 900 s, as well as on the participant’s age and sex) and used this expected value in place of the participant’s actual wait time. We calculated this expected value using simulation, using 10,000 draws from the model under the assumption that the parameter estimates in Table 2 are the true parameter values.

Our second measure, RNSRI, has four components: RND and survey measures of self-regulation measured at ages 17, 27, and 37. The latter three components are derived from the California Child Q-Set (CCQ), a set of personality descriptors (Block and Block, 1980). Versions of the CCQ were completed by participants’ parents with respect to their children’s personality around age 17 and by the participants themselves around ages 27 and 37.^4^

For each of the three ages, we constructed a measure of overall self-regulation from subsets of CCQ items, with items and weights determined by the following procedure. First, a preliminary set of 37 CCQ items relating to self-control was selected based on ratings by three of us (Peake, Shoda, and Wilson). These items were administered to an independent sample of 191 Smith College students. We conducted a principal components analysis, which found that all but one of these items loaded positively on the first unrotated principal component. In our Bing survey sample, using ratings obtained at ages 17, 27, and 37, we then conducted a confirmatory principal component analysis on the remaining 36 items. We eliminated five items from the analysis due to failure to demonstrate consistent positive loadings on the first unrotated principal component. From among the remaining 31 items, we formed two subscales, each consisting of 3 items, for “delay of gratification” and “general cognitive ability” (with the items selected based on their face validity for these concepts). We then factor-analyzed the remaining 25 items within the Smith sample using principal axis factoring and oblique rotation. We labeled the four oblique factors resulting from this analysis “attention,” “coping,” “goal pursuit,” and “concern for others”; see OA Section I-C for a list of the items included in each of the six subscales. For each participant at each age, we created rank-normalized subscale scores, giving equal weight to each item in the subscale. We then calculated a rank-normalized CCQ score for each respondent at each age, giving equal weight to each of the 6 subscales and then rank-normalizing the resulting variable.

We computed RNSRI by taking an equal-weighted mean of RND and rank-normalized CCQ score at ages 17, 27, and 37, and then rank-normalizing the resulting variable.

### 3.3. Statistical approach

With the data we had, we faced two main statistical challenges. First, our sample size is small: 113 survey respondents. Given the small magnitude of associations we anticipated, we expected to have low statistical power for detecting any particular association between a self-regulation measure and a capital formation measure. Moreover, due to low power, any association we identified as statistically significant would be likely to have an exaggerated effect-size estimate (e.g., Gelman and Carlin, 2014), a bias sometimes called the “statistical winner’s curse” (e.g., Ioannidis, 2008). Second, because the self-regulation and capital formation variables we examine could yield more than 600 different hypothesis tests, we faced a potential multiple-testing problem and its associated “data-mining” problems. Consequently, there would be a high rate of false positives at a *p*-value threshold of 0.05. Moreover, if we adopted a family-wise significance threshold such as a Bonferroni correction, we would exacerbate the problem of low power.

To address the potential multiple-testing problem, we pre-registered and time-stamped on Open Science Framework (https://osf.io/u39hg/) our coding of the variables and our planned series of analyses *before* calculating any correlations between independent variables and dependent variables.^5^ Moreover, we distinguished between four types of analyses: (i) tests of primary hypotheses, (ii) tests of secondary hypotheses, (iii) ex-post analyses, and (iv) robustness checks. Our pre-registration plan specified our tests of primary and secondary hypotheses and our robustness checks, and we committed to identifying any ex-post analyses as such in the paper. We further committed to treat tests of secondary hypotheses as exploratory. We pre-registered only two primary hypotheses, which we describe in Section 4. We report some of our secondary and ex-post analyses in Section 5, and we report all of them in the OA Section V.

Our planned series of analyses of our primary hypotheses were designed to address the challenge of our small sample size. We planned four analyses. First, with each of our capital formation measures as a dependent variable, we run an OLS regression on each measure of self-regulation, controlling for sex. (Regressions with the savings rate as the outcome also control for permanent income.) While we report the regression results because they are simple and serve as inputs to our other three tests, we do not use a *p*-value threshold to judge statistical significance due to the multiple-testing problem.

Second, to identify “significance” of associations between a self-regulation measure and a specific capital formation measure, we use a false discovery rate (FDR) threshold of 0.1. Compared with a family-wise error rate threshold, an FDR threshold has greater statistical power, albeit with a different interpretation: rather than controlling the error rate of any particular association, an FDR threshold of 0.1 means that among the *set* of “significant” associations, at most 10% are false positives. We compute the FDR of each association using the Benjamini and Hochberg (1995) algorithm, applied separately to the OLS *p*-values for each of the two self-regulation measures.

Third, for each self-regulation measure, we test for the *joint* significance of associations with our 11 capital formation measures. We use a Wald test with a *p*-value threshold of 0.05. Because the standard Wald test *p*-value is biased downward in small samples, we calculate an empirical *p*-value by comparing the observed Wald test statistic to a distribution of Wald test statistics under the null, which we generate by permuting our data. Specifically, we randomly permute the values of the dependent variable within the sample 1000 times, and we re-calculate the Wald test statistic each time. We calculate our empirical *p*-value as equal to the percentile where our observed Wald test statistic falls in the distribution of test statistics from the permuted samples.

Finally, to obtain an estimate of the magnitude of the association between each self-regulation measure and each capital formation measure, we report our Bayesian posterior mean and standard deviation for each coefficient. For each self-regulation measure, we pre-registered our prior about the mean and standard deviation of the OLS coefficients for the 11 capital formation measures. We calculate the posterior mean and standard deviation for each coefficient assuming normal distributions for both the prior and the observed OLS coefficient. Due to Bayesian “shrinkage” toward the prior, the Bayesian posterior means do not suffer from the “statistical winner’s curse” bias (Ioannidis, 2008) and can thus serve as our “best guess” effect-size estimates.

Before pre-registering our analysis, we used Monte Carlo (i.e., simulation) methods to confirm that given our priors, our planned FDR and Wald tests were well-powered.^6^ In Section 4, we compare our pre-specified priors to what we found.

Note that we originally hypothesized that the relationship between preschool waiting and mid-life capital formation would be stronger in the “diagnostic” conditions of the original Bing Study (for example, see Shoda et al., 1990; and p. 5 of our pre-registered analysis plan). Nevertheless, in order to maximize sample size, for tests of our primary hypotheses, we pooled respondents who participated in diagnostic and non-diagnostic experimental conditions, as we did in some of the prior work (Mischel et al., 1988; Ayduk et al., 2000, 2008; Schlam et al., 2013). Our pre-registered priors and corresponding power calculations take into account this pooling across conditions.

## 4. Pre-registered hypotheses, pre-registered predictions, and results

In this Section, we describe the two primary hypotheses that represent the core of our study.

**Hypothesis 1.** We hypothesize that RNSRI will have a positive, but relatively modest, correlation with the measures of mid-life capital formation.

Although we predicted that self-regulation plays a significant role in development, given that many other factors (peers, career choice, marital outcomes, family wealth, inheritance, chance, etc.) influence capital formation, we predicted that the correlation between self-regulation and capital formation is modest. Specifically, our pre-registered prior distribution for the correlation of RNSRI with each measure of mid-life capital formation had a mean of 0.15 (and standard deviation of 0.2). These predicted correlations are consistent with past results for these types of outcomes (e.g., Moffitt et al., 2011; Richard et al., 2003).

Tests of Hypothesis 1 are reported in the top panel of Table 3. Of the 11 capital formation measures, 10 are associated with the predicted sign. Six variables are significant at our FDR threshold of 0.1 (and the same six have *p*-values < 0.05): net worth, credit card misuse, financial health, forward-looking behaviors, educational attainment, and permanent income. In an *F*-test, the (joint) null that all 11 capital formation measures have no association is rejected (empirical Wald test *p*-value = 0.0005). OA Section V-A and V-D contains details of all these analyses, Bayesian posterior means and standard deviations for the correlation with each outcome, as well as pre-registered robustness analyses.

The average observed correlation across all 11 variables, $\bar{r}=0.19$ (SE = 0.04), is close to our pre-registered prior mean of 0.15. The average of our Bayesian posterior means is a correlation of 0.18.

**Hypothesis 2.** On its own, RND (measured around age four) will have only a very small correlation with the measures of mid-life capital formation.

As specified in our pre-registration, we predicted very small correlations between our measure of preschool waiting and measures of mid-life capital formation for three reasons (which we discuss further in Section 5). First, most of the respondents to the capital formation survey were tested in experimental conditions that have previously been designated as “non-diagnostic” (e.g., the experimenter suggested a specific self-regulation strategy to the child). Theoretical considerations and earlier findings (Shoda et al., 1990) led us to anticipate that variation within non-diagnostic conditions would generate less predictive power than variation within diagnostic conditions. Second, as noted for Hypothesis 1, self-control is only one of the many factors that influence mid-life capital formation. Third, the assessment of RND is separated in time from the capital formation variables by over 40 years. Research and theory suggest that while many people are stable in self-control over the life-course, many exhibit change, either towards increasing or decreasing self-control (Berman et al., 2013; Casey et al., 2011). In contrast, the other components of RNSRI average a separation of around 20 years from the capital formation variables and represent an average of many indicators of self-regulation. The predictive power of an index consisting of many items typically exceeds the predictive power of each of its components. Since RND is only one component of the 86-item RNSRI, we expected it to have less predictive power. Specifically, our pre-specified prior distribution for the correlation of RND with each measure of mid-life capital formation had a mean of 0.05.

Tests of Hypothesis 2 are reported in the bottom panel of Table 3. Of the 11 capital formation measures, 6 are positively correlated with RND, but none are significantly associated at our FDR threshold of 0.1 (and all have *p*-values > 0.05). In an *F*-test, the joint null of no effect for all 11 capital formation variables is not rejected (empirical Wald test *p*-value = 0.45). None of our pre-registered robustness analyses, including alternative measures of childhood wait time, substantively affects the conclusions from the bottom panel of Table 3 (see OA Section V-D).

The average observed correlation across all 11 variables, $\bar{r}=0.02$ (SE = 0.05), is close to our pre-registered prior mean of 0.05. The average of our Bayesian posterior means is a correlation of 0.03.

## 5. Additional analyses

### 5.1. Mechanisms

As explained above, three mechanisms motivated our pre-registered hypotheses that the index of self-regulatory measures would be moderately predictive, while the preschool delay of gratification task would be very weakly predictive. These mechanisms flow from standard statistical properties and our understanding of the psychological processes underlying the cumulative findings of past empirical studies:

1. The index of self-regulatory measures is comprised of 86 responses per participant, whereas the preschool delay of gratification task is a single behavioral task. An index of similar measures tends to have a higher signal-to-noise ratio than its components.
2. The preschool delay of gratification task is measured using a diagnostic variant of the task for 34 of our 113 participants; the remaining 79 participants experienced a non-diagnostic variant of the pre-school delay of gratification task. Pooling across diagnostic and non-diagnostic conditions weakens the correlation with outcome variables.
3. The index of self-regulatory measures is comprised of questions that are measured throughout the life course up to age 37 (specifically, ages 4, 17, 27, and 37), whereas the preschool delay of gratification task is measured at age 4. Self-regulation measured closer in time to the observed outcomes will be more strongly related to them.

We now discuss (pre-registered) secondary and (non-pre-registered) ex-post analyses that shed some light on these mechanisms. We find support for the first, suggestive evidence in favor of the second, and no support for the third.

To test the first mechanism, we evaluate the predictive power of *each* of the 85 components of the CCQ index compared to the predictive power of the RNSRI index taken as a whole. In this ex-post analysis, we regressed each of the capital formation measures on each of the 85 items in the RNCCQ indices (23 at age 17, 31 at age 27, and 31 at age 37) in separate regressions (i.e., 85 CCQ items × 11 outcomes = 935 regressions). In Table 4, we report the average coefficient across all 935 regressions of a capital formation measure on a rank-normalized CCQ item. This average correlation is 0.07 (SE = 0.03), which is smaller than 0.19 (SE = 0.04), the average correlation reported above between the RNSRI index and each of the 11 capital formation variables. In a related ex-post analysis, we found stronger correlations between self-regulation measures and the capital formation measures when we use an *index* of capital formation variables rather than examining them separately. We constructed the capital formation index by averaging the 11 capital formation measures for the 102 subjects with no missing outcome variables and then rank-normalizing this average. The correlation between RNSRI and the capital formation index is 0.43 (SE = 0.09), and 0.13 (SE = 0.10) between RND and the capital formation index; see OA.^7^

The second mechanism is a potential explanation for the weak relationships we find with RND. The preschool delay of gratification task was run in many different ways and only some of those trials were theorized to be diagnostic (Shoda et al., 1990). For example, in some trials the four-year-old participants were asked to think about the rewards in a particular way that was hypothesized to facilitate delay (e.g., think of marshmallows as clouds). In other trials, the participants were not given any suggested cognitive strategies. These two methods are referenced in the literature as *suggested ideation* and *spontaneous ideation*. In the original Bing studies, this ideation manipulation was crossed with a reward-exposure manipulation: in some cases the rewards were exposed (elevating appetitive responses and thereby making delay relatively more difficult), and in other cases the rewards were covered or removed. Experimental settings with spontaneous ideation and exposed rewards were theorized to be the most likely to be “diagnostic” of self-regulatory competencies. Indeed, it was only these settings that produced the significant correlation between preschool wait time and subsequent adolescent outcomes, including parental CCQ item ratings of adolescents employed in the current research and SAT scores (Shoda et al., 1990).

Specifically, in our current data, only 34 participants were assessed in the diagnostic version of the preschool delay task. We pre-registered an analysis to examine the predictive power of RND separately for participants in the diagnostic and non-diagnostic version of the preschool delay task, but we classified it as a secondary analysis because we anticipated large standard errors due to the smaller sample sizes. Based on past results, we hypothesized that RND would have more predictive power among the diagnostic participants. As shown in Table 4, in the diagnostic condition the average correlation between RND and the 11 human capital measures is 0.07 (SE = 0.09), which, incidentally, is equal to the average correlation between the individual components of the CCQ index and the 11 human capital measures. In contrast, the average correlation between preschool wait time in the *non*-diagnostic conditions and the 11 human capital measures is −0.01 (SE = 0.06). Hence, our evidence provides suggestive support for the earlier finding that the diagnostic conditions are the relevant ones for assessing individual differences in self-regulatory competence—albeit with two caveats, both related to the large standard errors: (i) the coefficients in both conditions are statistically indistinguishable from zero, and (ii) the diagnostic and non-diagnostic correlations are not statistically distinguishable from each other. If we take the point estimates at face value, they suggest that once we level the playing field, by comparing single questions from the self-regulatory index to the diagnostic variant of the preschool delay task, the predictive power of preschool waiting time is identical to the typical reported CCQ item assessing self-regulation later in the life-course. However, re-analysis of prior evidence raises questions about the consistency of the differences between diagnostic and non-diagnostic conditions. In an ex-post analysis suggested in the editorial process, we re-analyzed the correlation between BMI and preschool wait time in Schlam et al. (2013) separately for the diagnostic and non-diagnostic subsamples. Of the 164 subjects in the BMI study, 37 were in a diagnostic condition (25 of whom are also in the current survey sample). Contrary to our findings, the relationship between preschool delay (measured as the deviation from the condition mean) and BMI is weaker in the diagnostic group (*r* = −0.03, SE = 0.14) than in the non-diagnostic group (*r* = −0.24, SE = 0.08), though the difference is not statistically distinguishable. The correlations between BMI and delay deviation are similar if we minimize the effect of outliers by winsorizing the data. See OA Section V-C-8 for more detail. It is also worth noting that two studies using data from the NICHD-SECCYD find the expected *negative* relationship between wait time in the diagnostic version of the preschool task and adolescent BMI (Seeyave et al. (2009) and Francis and Susman (2009)). In light of all these results, evidence for the importance of the diagnostic methodology is mixed.

To test the third mechanism, in an ex-post analysis we examined the relationship between the date of measurement and predictive power. Surprisingly to us, we found no relationship between the age at which the self-regulation questions from the CCQ were administered and the predictive power of those questions for mid-life capital formation. Specifically, the mean correlation with the capital formation measures for the questions administered at age 17 is 0.08 (SE = 0.05), the mean correlation from the questions administered at age 27 is 0.07 (SE = 0.02), and the mean correlation for the questions administered at age 37 is 0.08 (SE = 0.02) (Table 4).^8^

### 5.2. Excluding RND from the self-regulation measure

We formulated our primary hypotheses in terms of RND and RNSRI, where the former self-regulation measure is a component of the latter. Among our pre-registered secondary analyses, we also examined an RNCCQ index, which is otherwise the same as RNSRI (i.e., it is composed of the CCQ indices from ages 17, 27, and 37) but excludes RND. This RNCCQ index has correlation *r* = 0.16 (SE = 0.09) with RND in the survey sample.^9^ Considering the predictive power of the RNCCQ index on its own, we find that its mean correlation with the capital formation measures is 0.20 (SE = 0.04) whether we control for RND (pre-registered secondary analysis) or not (ex-post analysis); see OA Sections V-B-2 and V-C-14 for more details. Thus, the inclusion of RND in the RNSRI index does not change its predictive power for the capital formation measures.

### 5.3. Money now vs. money later as a predictor

One of our capital formation variables, delay choice, is based on responses to money earlier vs. later questions.^10^ Early research on self-control in children relied heavily on delay choice data (Mischel, 1958, 1961a, 1961b; Mischel and Gilligan, 1964; Bandura and Mischel, 1965; Mischel and Staub, 1965; Mischel and Grusec, 1967). Recognition of the conceptual limitations of delay choice measures was a primary motivation for the development of the preschool delay of gratification paradigm (Mischel and Ebbesen, 1970; see Peake, 2017 for discussion). Nonetheless, monetary delay choice measures (e.g., money earlier vs. later) have been popular among economists and psychologists as a tool for assessing time preferences and hence, as a putative index of self-control and impulsive behavior (e.g. Thaler, 1981; see Stevens, 2017 for examples). We consider (monetary) delay choice to be a dependent variable in our primary analysis, but in an ex-post analysis, we examined it as a predictor of the remaining capital formation variables (as in Meier and Sprenger (2010), Meier and Sprenger (2013), and Falk et al. (2018)).

Delay choice is a rank-normalized variable measuring the percent of times a respondent selected the later response in a series of incentivized money earlier vs. later questions. The questions included 20 of the form “Which would you prefer? (A) getting $X today (B) getting $Y thirty days from today” and 20 of the form “Which would you prefer? (A) getting $X thirty days from today (B) getting $Y sixty days from today,” with values ranging from $10 to $29.

We regressed the remaining 10 capital formation measures on delay choice, controlling for sex. Although all 10 correlations have the predicted sign, none is significant at the 5% level. The average correlation with the capital formation measures is 0.11 (SE = 0.06). See OA Section V-C-9 for more detailed information.

### 5.4. Other secondary, robustness, and ex-post analyses

In OA, we report the results from all pre-registered secondary and robustness analyses as well as all ex-post analyses we conducted. In our secondary analyses, we examine additional outcome variables, secondary sets of independent variables (e.g., breaking out the RNSRI into its components), and comparisons of diagnostic and non-diagnostic conditions.

In our ex-post analyses, we report correlations between our key variables, results using individual questions from the RNCCQ as independent variables, an analysis of the characteristics of the Bing sample over time, an in-depth analysis of BMI as an outcome variable, results using delay choice as an independent variable, robustness of the primary analyses to controlling for age, results using an index of capital formation measures as the dependent variable, and an analysis of the effects of rank-normalization. We also report results from two ex-post analyses resulting from the editorial process: analyses of RNCCQ as an independent variable on its own, and a comparison of the BMI correlations in the diagnostic and non-diagnostic subsamples using BMI data collected in previous surveys.

In our robustness analyses, we test the robustness of our primary analyses to measuring wait time in seconds rather than log-seconds, winsorizing RNSRI and RND, estimating a non-linear effect of RND, using alternative imputation methods for the RNCCQ indices, using the delay deviation measure from prior work, and dropping survey respondents for whom we had to make assumptions in order to assign values to any of their capital formation measures.

## 6. Concluding remarks

We have reported pre-registered analysis of the latest survey wave of the Bing pre-school study on delay of gratification. Respondents were in their late 40’s at the time of this latest wave and were asked to report 11 mid-life capital formation outcomes (e.g., net worth and permanent income). Our analysis plan both described our methods and predicted what we would find. As predicted, a newly constructed measure derived from preschool delay of gratification does *not* predict the 11 capital formation variables (i.e., the sign-adjusted average correlation was 0.02). By contrast a composite self-regulation index, combining preschool delay of gratification with survey measures of self-regulation collected at ages 17, 27, and 37, does predict 10 of the 11 capital formation variables in the expected direction, with an average correlation of 0.19. The inclusion of the preschool delay of gratification measure in this composite index does not affect the index’s predictive power for two reasons. Most importantly, the index of self-regulatory measures is comprised of 86 responses per participant, whereas the preschool delay of gratification task is a single behavioral task. In addition, the preschool delay of gratification task is measured using a diagnostic variant of the task for 34 of our 113 participants; the remaining 79 participants experienced a non-diagnostic variant of the pre-school delay of gratification task.

The data we have analyzed is unique because the Bing cohort is the only sample where preschool delay of gratification has been studied long enough to examine relationships with mid-life outcomes. While the tests of our primary hypotheses were well powered, we caution that our sample is relatively small and not representative of the overall population—e.g., 97% of our sample has a four-year college degree (the exceptions are one participant who has a two-year college degree and two who have some college but no degree)—limiting the generalizability of the results.

However, we can compare our results to the small set of overlapping analyses that have been conducted using the Dunedin cohort, which began collecting data in 1972–1973 and has childhood self-regulation measures but no preschool delay of gratification measure.^11^ The cohort is from a small town in New Zealand with much lower levels of educational attainment (Moffitt et al., 2011). Specifically, 29% of the Dunedin sample is college educated (Belsky et al., 2016). Despite the stark socioeconomic differences, the self-regulation measures used in the Dunedin study have similar predictive power to the self-regulation measures in the Bing sample. For example, Moffitt et al. (2011) found that a 1 SD increase in childhood self-control as measured in the Dunedin study predicts a 0.24 SD increase in income at age 32. In the Bing sample, a 1 SD increase in RNSRI predicts a 0.32 SD increase in rank-normalized permanent income. Similarly, a 1 SD increase in the Dunedin self-control measure predicts a 0.12 SD decrease in credit card problems, while a 1 SD increase in the Bing RNSRI predicts a 0.18 SD decrease in rank-normalized credit card misuse. A 1 SD increase in the Dunedin self-control measure predicts a 0.14 SD decrease in money management difficulties, while a 1 SD increase in the Bing RNSRI predicts a 0.24 SD increase in financial health.^12^ Despite these intriguing similarities across the two samples, the issue of generalizability remains an important question to be addressed in future research as mid-life data becomes available in more childhood longitudinal cohorts.

## Supplementary Material

1

2

3

4

5

## Figures and Tables

**Table 1:** Means, medians, and standard deviations of survey measures (prior to rank-normalizations).

	Survey Sample (*N* = 113)			Full Sample (*N* = 543)			
	Mean	Median	SD	Mean	Median	SD	*p*-value
*Preschool measures*							
Male	37%			48%			0.01
Age at wait task (months)	51.6	52.0	5.6	52.1	52	5.7	0.36
Raw wait time (seconds)	509.7	556.0	369.1	470.1	440	367.7	0.20
Deviation from predicted preschool wait time (ln seconds)	0.08	0.51	2.37	0.00	0.27	2.30	0.66
*Demographics at economics survey*							
Age	46.0	46.0	2.0				
Married	79%						
Has children (among those with information)	89%						
Missing information on children	20%						
White	94%						
Asian	4%						
*Capital formation measures*							
Net worth ($ millions)	1.8	0.9	3.4				
Permanent income ($ thousands)	131	78	147				
Wealth-income ratio	13.5	10.1	14.6				
High interest-rate debt ($ thousands)	1.2	0.0	2.6				
Credit card misuse	–	–	–				
Delay choice (% later choices)	85%	89%	14%				
Savings rate	11.0%	10.0%	9.3%				
Financial health	–	–	–				
Educational attainment (years)	19.1	19.0	2.1				
Forward-looking behaviors	–	–	–				
Social Status	7.6	8.0	1.4				

*Notes*: Entries of “−” are for measures with no natural scale. *p*-value is from an OLS regression of each variable on a constant and an indicator variable for being in the survey sample. See OA Section II for more detailed information on sample size for each outcome.

**Table 2:** Parameter estimates from tobit random effects model of log preschool wait time, controlling for age, sex, and experimental condition.

	Wait time in log-seconds
*β*_1_: Age at delay of gratification task (months)	0.081*** (0.020)
*β*_2_: Male	−0.673*** (0.227)
*α*: Constant	2.289** (1.071)
*σ_ε_* : SD of Individual-Specific Error	2.469*** (0.101)
*σ_η_* : SD of Random Effect (Experimental Condition)	0.786*** (0.218)
*N*	543

*Notes:* Parameter estimates from a tobit random effects regression of preschool wait time (measured in log-seconds) on age at delay of gratification task, sex, and a constant. *σ_η_* is the estimated standard deviation of the experimental condition effect, and *σ_ε_* is the estimated standard deviation of the individual-specific error. Standard errors are in parentheses.

* *p* < 0.1

** *p* < 0.05

*** *p* < 0.01.

**Table 3:** Coefficients from OLS regressions of RNSRI (top panel) and RND (bottom panel) on each of the 11 capital formation measures, controlling for sex.

	(1) Net worth	(2) Perm income	(3) Wealth income ratio	(4) High interest-rate debt (reverse)	(5) Credit card misuse (reverse)	(6) Delay choice	(7) Savings rate	(8) Financial health	(9) Education years	(10) Forward looking behaviors	(11) Social status
RNSRI	0.31*** (0.10)	0.32 *** (0.09)	0.09 (0.10)	0.09 (0.08)	0.18** (0.08)	0.16* (0.09)	−0.01 (0.09)	0.24** (0.09)	0.24*** (0.09)	0.35*** (0.09)	0.14 (0.09)
*N*	106	109	106	106	110	109	103	110	110	110	110
RND	0.09 (0.10)	−0.08 (0.10)	0.15 (0.10)	−0.01 (0.08)	0.04 (0.08)	0.09 (0.09)	−0.06 (0.09)	−0.07 (0.09)	0.13 (0.09)	0.09 (0.09)	−0.13 (0.09)
*N*	109	112	109	109	113	112	106	113	113	113	113

*Notes*: All regressions control for sex, and regressions with savings rate as the dependent variable also include control for permanent income (coefficients not reported in the table are reported in the OA Section V-A). Standard errors are in parentheses.

* *p* < 0.1

** *p* < 0.05

*** *p* < 0.01.

**Table 4:** Mean of coefficients from regressions of the 11 capital formation measures on RNSRI, RND, and individual CCQ items.

Measure(s) of Self-Regulation	Type of Analysis	Mean of Coefficients Across Measures of Capital Formation
RNSRI (includes RND and Age 17, 27, and 37 CCQ indices)	Pre-registered primary	0.19 (0.04)
RND	Pre-registered primary	0.02 (0.05)
RND (diagnostic condition)	Pre-registered secondary	0.07 (0.09)
RND (non-diagnostic condition)	Pre-registered secondary	−0.01 (0.06)
All CCQ items (avg over 85 items)	Ex-post	0.07 (0.03)
Age 17 CCQ items (avg over 23 items)	Ex-post	0.08 (0.05)
Age 27 CCQ items (avg over 31 items)	Ex-post	0.07 (0.02)
Age 37 CCQ items (avg over 31 items)	Ex-post	0.08 (0.02)

*Notes*: All regressions control for sex, and regressions with savings rate as the dependent variable also include control for permanent income (coefficients on control variables not reported). For the regressions on CCQ items, the sign of each coefficient is adjusted to be positive before the coefficients are averaged. Bootstrapped standard errors are in parentheses.

## Footnotes

1 Other long-running longitudinal studies with measures of self-regulation include the Dunedin Multidisciplinary Health and Development Study and the NICHD Study of Early Child Care and Youth Development (SECCYD). The Dunedin cohort was first assessed at age 3 in the mid-1970’s and most recently assessed at age 38 (with an ongoing assessment at age 45). While the Dunedin study did include parent and teacher survey measures and observer reports of self-regulation in preschool, it did not include the Mischel delay of gratification task. The NICHD-SECCYD began collecting data in the early 1990’s when children were aged 0-3 and collected the final wave of data in the mid-2000’s when the children were 15 years old. The NICHD-SECCYD contains a version of the Mischel delay of gratification task as well as parent and teacher survey measures of self-regulation from birth through early adolescence.

2 The survey was approved by the institutional review boards at Harvard University and the University of Washington. All participants provided informed consent. Many of the questions used to form the economic outcome variables were taken from the Cognitive Economics Project (http://ebp-projects.isr.umich.edu/CogEcon/).

3 In prior work, wait times were adjusted to account for experimental condition by taking the difference from the mean wait time in a condition (equivalent to a fixed-effects specification). In the current work, we instead treat the experimental conditions as random effects (as described below) because mean wait time in a condition is estimated imprecisely when the per-condition sample size is small. Essentially, the random effects model predicts the effect of an experimental condition as a weighted average of the observed mean wait time in the condition (conditional on age and sex) with the mean wait time across experimental conditions. The larger the sample size for an experimental condition, the larger the weight assigned to the observed mean wait time in that condition. In addition to the treatment of experimental conditions, RND also differs from wait time used in prior work because it accounts for the child’s age and sex, it adjusts for the fact that observed wait times are capped at 15 minutes, and we measure wait time in log-seconds rather than seconds. Finally, we note that our new rank-normalized measure may or may not be an improvement on the old absolute-deviation-from-the-mean measure. See OA for more discussion of the methodological differences.

4 As we report in Section 5, contrary to what we expected, the predictive power of the self-regulation measures does *not* increase as the age of measurement gets closer to the age at which we measure the capital formation variables.

5 In a slight abuse of terminology, we will often refer to “correlations” between our independent and dependent variables. As described below, what we will actually report are *coefficients* from OLS regressions of our dependent variables on measures of self-regulation, controlling for sex and other variables. Because both the independent and dependent variables are normalized, the regression coefficients can be interpreted as partial correlation coefficients.

6 For example, for an average correlation of 0.15 between our dependent variables and an independent variable, our simulations yielded on average 3.9 of 11 dependent variables associated with a *p*-value less than 0.05, 3.4 associated at a false discovery rate threshold of 0.1, and in 97% of simulations, we reject the (joint) null that all 11 variables each have no effect with an empirical Wald test *p*-value less than 0.05. Instead, for an average correlation of 0.05, our simulations yielded on average 0.9 of 11 dependent variables associated with a *p*-value less than 0.05, 0.3 associated at a false discovery rate threshold of 0.1, and in 18% of simulations, we reject the (joint) null that all 11 variables each have no effect with an empirical Wald test *p*-value less than 0.05.

7 Some of the outcome variables, such as the total years of education and income at age 35, precede the age 37 self-regulation measurement. In ex-post analyses, we found that this does not drive our main results; excluding educational attainment, permanent income, and wealth-to-income ratio from the primary outcomes, the average correlation between RNSRI and the remaining outcomes is 0.18. This is consistent with our finding that self-regulation measured at age 37 is not a better predictor of our primary outcomes than measurements at age 17 and 27. Focusing on the three potentially problematic outcomes, while educational attainment does appear to be most strongly predicted by self-regulation at age 37, permanent income and wealth-to-income ratio are not.

8 Similarly, there is limited evidence for an age trend in the predictive power of the *indices* of CCQ questions. In ex-post analysis, we found that the average correlations between the mid-life capital formation measures and RNCCQ at ages 17, 27, and 37 are 0.15 (SE = 0.09), 0.17 (SE = 0.06), and 0.19 (SE = 0.05), respectively (see OA).

9 The overall correlation between RND and RNCCQ is calculated by combining RNCCQ data across ages 17, 27, and 37. The correlations for each respective age bucket are *r* = 0.06 (SE = 0.10), *r* = 0.23 (SE = 0.09), and *r* = 0.15 (SE = 0.10). Readers interested in other subcomponents of RNCCQ (including the factor-analytic subscales) can find the associated correlations with RND in the OA.

10 For a review and methodological critique of money earlier vs. later questions, see Cohen et al (forthcoming). For example, in principle choices in the money earlier vs. later paradigm should reveal market interest rates, transactions costs, and trustworthiness of the experimenter, and should *not* measure true time preferences (because money is temporally fungible).

11 Specifically, the measure of self-regulation used in Moffitt et al. (2011) is an index of examiner ratings of lack of control at ages 3 and 5; parent and teacher ratings of impulse aggression and hyperactivity at ages 5, 7, 9, and 11; parent and teacher ratings of lack of persistence and impulsivity at ages 9 and 11; and self-reported symptoms of hyperactivity, inattention, and impulsivity at age 11. This measure does not include performance on the delay of gratification task that we use to construct our RND measure.

12 Other outcomes studied in Moffitt et al. (2011) at age 32 include measures of physical health, depression, substance dependence, socioeconomic status, single-parent child rearing, financial planfulness, and criminal conviction.
