# Supplementary material for: Predicting mid-life capital formation with pre-school delay of gratification and life-course measures of self-regulation
Source: J Econ Behav Organ. Author manuscript; Available in PMC 2021 Jan 8. (PMC7792663; doi:10.1016/j.jebo.2019.08.016)
Supplement: 1 [file NIHMS1543524-supplement-1.pdf]

1. Introduction

BING SCHOOL LONGITUDINAL RESEARCH PROJECT

Dear Bing Nursery School Alumni,

The Columbia-Stanford University Bing School Longitudinal Research Project is continuing to yield important new findings that are advancing psychological science with new and surprising insights into development over the life span. It is also receiving increasing media attention. We are deeply grateful for your participation, which has made these contributions possible.

To move this project further into important new directions, we are inviting you to participate in a survey about people's financial well-being, relationships, health and health habits, and other behaviors. The survey typically takes about an hour or less to complete. As a token of our appreciation, you will receive a $100 gift certificate to Amazon.com for participating.

If you choose to participate in this study, you will be asked a series of questions about your thoughts and behaviors, your household finances, your relationships, and your health and health habits. One part of the study will also involve playing a game in which you'll have a chance to win a bonus in addition to the standard $100 Amazon.com gift certificate mentioned above. Further details about the study appear on the next page, where you will be given more information about the risks and benefits of participating in this study.

Your time, attention, and help are critical in making this work possible. Please know that we value and appreciate your participation deeply. If you have any questions or comments, please feel free to contact us at bingstdy@uw.edu.

Please print or save a copy of this page for your records. Then, please proceed to the next page to get further information and to either agree to participate in the study or to exit.

2. Informed Consent, Part 1

Researchers:

Nicole Wilson (Post-Doctoral Research Associate, University of Washington, Department of Psychology, (319)471-1899)

Yuichi Shoda (Professor, University of Washington, Department of Psychology, (206)543-2318)

Study Purpose and Procedures. The current study is part of the Stanford Bing School Longitudinal Research Project. In it, we are examining people's financial well-being, health, and other behaviors and personal characteristics. Participation in the study involves completing an online survey. If you choose to participate in the study, you will be asked to answer a series of questions about your household finances, your financial preferences, your general health and health habits, and other related thoughts, feelings, opinions, and behaviors.

Participation and Withdrawal. Participation in this study is completely voluntary. You may withdraw from the study at any time without penalty. You may also skip any question, but continue to complete the rest of the survey. If you begin the survey and do not complete a significant portion of it within one week, we may send you a reminder that you have not yet finished the survey. However, your participation is voluntary and you are not obligated to finish the survey even if you have started it.

Time to Complete the Survey. Although the amount of time it takes to complete the survey may vary from person to person, we anticipate that it will take most participants approximately one hour to complete.

Compensation. You will receive a $100 gift certificate to Amazon.com for participating in this study. As part of the survey, you will be asked to play some games in which you indicate your preference for certain financial options between $0 and $44. You will receive a bonus gift certificate based on your response to one of the game's questions. Specifically, you will receive the value that you preferred in that item (between $0 and $44) in the form of an additional Amazon.com gift certificate. You will not know until the end which item is selected for the bonus.

3. Informed Consent, Part 2

Risks, Discomfort, and Confidentiality. This section describes the potential risks and discomfort that you might experience by taking part in this study. It also describes the measures that we are taking to assure the confidentiality of information that you provide. Please be aware of these risks and the measures that we are taking to minimize them before you agree, or do not agree, to participate in this study.

If you choose to participate in the study, you may find that some of the questions that we will ask you are personal or sensitive in nature. Because questions about household finances, relationships, and personal health can be highly personal, we understand that the information you provide in this survey must be vigorously protected.

The primary risk associated with online survey studies such as this one is that an outside party (someone other than you and the researchers involved in the Bing School Longitudinal Research Project) could attempt to identify you or to access your responses. Below, we describe the steps we are taking to ensure your confidentiality:

1) We ask that you complete the online survey in a reasonably private setting and exercise caution while doing so. This will prevent others from observing your responses as you enter them (e.g., if you completed the survey on your laptop in a café and a stranger looked over your shoulder to see your responses).

(Continued on the next page)

4. Informed Consent, Part 3

2) You will not be asked to enter your name or any other personally identifying information into the online survey. Instead, we will ask you to enter a randomly-generated subject ID code that we have assigned to you. The link between your name and your subject ID code will be used to match up your data from the current study with data from earlier follow-up studies in which you have participated and to arrange for compensation for participating in the current study. To ensure the confidentiality of your data, that link will be stored in a secure environment and will never be included with your survey responses.

3) As you submit your responses, your data will be transmitted over the internet. That information will not include any personally identifying information such as names, social security numbers, or contact information, although your computer's IP address might be temporarily linked to your data. To ensure that data transmission is secure, our online data collection system uses Secure Socket Layer (SSL) encryption technology. This is a standard method used to ensure the security of online survey data, as well as other sensitive information (e.g., credit card information used for online purchases).

4) After your responses have been submitted and have been matched up with your data from earlier Bing follow-up studies, they will continue to be stored without any identifying information. Your responses will not be shared with any outside parties or agencies (e.g., the IRS or health insurance companies). Data from this research activity will be shared between researchers at Harvard University, University of Washington, and the National Bureau of Economic Research.

5) In any resulting publications, the data will be reported in a strictly anonymous form.

5. Informed Consent, Part 4

Study Benefits. We do not anticipate direct benefits to participants as a result of completing the survey, although participants may find the experience interesting and may enjoy learning about themselves while completing the survey. Although they may not directly benefit participants, we believe that the findings of the study will contribute to our understanding of development over the life course.

Contact Information. If you have any questions or concerns about this research, please contact us via e-mail at bingstdy@uw.edu or by telephone at (319)471-1899.

If you have questions about your rights as a research subject, concerns, suggestions, complaints, or reports of research-related harm that are not being addressed by the researchers, please contact either Jane Calhoun at (617)495-5459 (postal mail: Harvard University Committee on the Use of Human Subjects in Research, 1414 Massachusetts Avenue, Room 234, Cambridge, MA 02138, e-mail: jcalhoun@fas.harvard.edu) or the University of Washington's Human Subjects Division at (206)543-0098 (postal mail: Human Subjects Division, University of Washington, Box 359470, Seattle, WA 98195).

Consent to Participate. At the bottom of this page, please indicate whether or not you would like to participate in this study. Please also print or save a copy of this page for your records. If you agree to participate in the study, you are also agreeing to the following statement:

The nature and purpose of this research have been satisfactorily explained to me and I agree to become a participant in the study as described above. I understand that I am free to discontinue participation at any time if I so choose and that the investigator will gladly answer any questions that arise during the course of the research.

1. Please click below to indicate whether you agree to participate in this study.

- ◯ Yes, I agree to participate in this study.
- ◯ No, I do not agree to participate in this study.

A response to this question is required for participation.

6. No consent to participate

On the previous page, you responded that you would not like to participate in this study. If this is correct, please click the button below to exit this study. If you would like to participate in the study, please click the button below to return to the consent page. You can then click the "I agree to participate in this study" button and proceed with the survey.

1. What would you like to do?

- ◯ I would not like to participate in this study. Please exit the survey.
- ◯ Oops! I would like to participate in this study. Please take me back to the consent page.

7. No consent - exit page

Thank you for your time. Please close this browser to exit the survey.

8. Participant ID Codes

1. Please enter the 4-digit ID code that was included with the invitation to participate in this study.

A response to this question is required for participation. Entering the correct code is important, as it will allow us to match your survey responses with the data we have collected from you in earlier studies.

9.

Filling out the Survey

There is a progress meter at the top of each page that indicates approximately how much of the survey you have completed. This progress meter takes into account all of the pages in the online survey, including the consent materials and instructions.

As you are filling out the survey, you can take a break at any time. The survey will not time out, so you can leave the browser open and continue filling out the survey later, as long as you are working on a private computer so no one else will see your responses. It is also possible to close your browser during a break and then resume the survey later on. As long as you are on the same computer, simply click on the survey link again and you can continue the survey where you left off.

If you need to return to a previous page to check an instruction set or to change an answer, you can click the "Previous" button at the bottom of each page. Although it is possible to change an answer, we encourage you to only do so in order to correct an error, not to make your answers to different questions seem more consistent. Please do not hit the "Back" button on your browser.

(Continued)

10.

To measure the variables that we are interested in, we must sometimes ask similar questions in slightly different ways. To compare the results from this study to those from other larger longitudinal studies requires us to use measures geared to a broad range of participants. Even if some of these questions seem redundant or if the answers seem obvious, please try to answer them as accurately as possible.

Although we may ask questions that seem similar, please do not worry about making sure that your answers are consistent from question to question. Just treat each question individually and answer it as best you can.

We are now turning to the first set of questions. These demographic questions deal with your personal background and different characteristics of your family and household.

11.

1. What is your current age?

2. What is your sex?

- ◯ Male
- ◯ Female

3. What is your racial background (please check all that apply)?

- ☐ American Indian or Alaska Native
- ☐ Native Hawaiian or Other Pacific Islander
- ☐ Asian or Asian American
- ☐ Black or African American
- ☐ White or European American

Other (please specify)

4. What is your ethnicity (regardless of racial background)?

- ◯ Hispanic or Latino
- ◯ Not Hispanic or Latino

12.

1. Which of the following best describes your marital status?

- ◯ Never married
- ◯ Member of an unmarried couple (sharing a household)
- ◯ Married
- ◯ Separated
- ◯ Divorced
- ◯ Widowed

2. Including yourself, how many people in each of the following age groups is currently living in your household?

Children, ages 0-4 years	
Children, ages 5-12 years	
Children, ages 13-18 years	
Adults	

3. How many of these people contribute (in the form of income) to your household finances?

13.

1. Which of the following best describes your employment status?

- ◯ Employed (by a company or individual) for wages
- ◯ Self-employed
- ◯ A full-time homemaker
- ◯ A full-time student
- ◯ Currently seeking employment
- ◯ Retired
- ◯ Unable to work
- ◯ Not currently working or seeking employment for other reasons

2. What is the highest level of education you have attained?

- ◯ High school diploma or equivalent
- ◯ Some college education
- ◯ College degree
- ◯ Some graduate or professional education
- ◯ Graduate or professional degree

14.

For any degree(s) that you hold, please specify below the type of degree you have earned and specify the college, university, or institution that granted it.

1.

Type of Degree (e.g., B.A., M.S., etc.)

Name of College, University, or Institution Granting the Degree

2.

Type of Degree (e.g., B.A., M.S., etc.)

Name of College, University, or Institution Granting the Degree

3.

Type of Degree (e.g., B.A., M.S., etc.)

Name of College, University, or Institution Granting the Degree

4.

Type of Degree (e.g., B.A., M.S., etc.)

Name of College, University, or Institution Granting the Degree

5.

Type of Degree (e.g., B.A., M.S., etc.)

Name of College, University, or Institution Granting the Degree

15.

We are now turning to the next set of questions. These questions deal with your health.

1. In general, would you say your health is:

- ◯ Excellent
- ◯ Very Good
- ◯ Good
- ◯ Fair
- ◯ Poor

2. Compared to one year ago, how would you rate your health in general now?

- ◯ Much better now than one year ago
- ◯ Somewhat better now than one year ago
- ◯ About the same
- ◯ Somwhat worse now than one year ago
- ◯ Much worse now than one year ago

16.

1. The following items are about activities you might do in a typical day. Does your health now limit you in these activities? If so, how much?

	Yes, limited a lot	Yes, limited a little	No, not limited at all
Vigorous activities, such as running, lifting heavy objects, participating in strenuous sports	◯	◯	◯
Moderate activities, such as moving a table, pushing a vacuum cleaner, bowling, or playing golf	◯	◯	◯
Lifting or carrying groceries	◯	◯	◯
Climbing *several* flights of stairs	◯	◯	◯
Climbing *one* flight of stairs	◯	◯	◯
Bending, kneeling, or stooping	◯	◯	◯
Walking *more than a mile*	◯	◯	◯
Walking *several blocks*	◯	◯	◯
Walking *one block*	◯	◯	◯
Bathing or dressing yourself	◯	◯	◯

17.

1. During the past 4 weeks, have you had any of the following problems with your work or other regular daily activities *as a result of your physical health*?

	Yes	No
Cut down the amount of time you spent on work or other activities	◯	◯
Accomplished less than you would like	◯	◯
Were limited in the kind of work or other activities	◯	◯
Had difficulty performing the work or other activities (for example, it took extra effort)	◯	◯
Didn't do work or other activities as carefully as usual	◯	◯

2. During the past 4 weeks, have you had any of the following problems with your work or other regular daily activities as a *result of any emotional issues* (such as feeling depressed or anxious)?

	Yes	No
Cut down the amount of time you spent on work or other activities	◯	◯
Accomplished less than you would like	◯	◯
Were limited in the kind of work or other activities	◯	◯
Had difficulty performing the work or other activities (for example, it took extra effort)	◯	◯
Didn't do work or other activities as carefully as usual	◯	◯

18.

1. What degree of bodily pain have you had during the past 4 weeks?

- ◯ None
- ◯ Very mild
- ◯ Mild
- ◯ Moderate
- ◯ Severe
- ◯ Very severe

2. During the past 4 weeks, how much did bodily pain interfere with your normal work (including both work outside the home and housework)?

- ◯ Not at all
- ◯ A little bit
- ◯ Moderately
- ◯ Quite a bit
- ◯ Extremely

19.

1. During the past 4 weeks, *to what extent* has your physical health or emotional problems interfered with your normal social activities with family, friends, neighbors, or groups?

- ◯ Not at all
- ◯ Slightly
- ◯ Moderately
- ◯ Quite a bit
- ◯ Extremely

2. During the past 4 weeks, *how much of the time* has your physical health or emotional problems interfered with your social activities with family, friends, neighbors, or groups?

- ◯ All of the time
- ◯ Most of the time
- ◯ Some of the time
- ◯ A little bit of the time
- ◯ None of the time

20.

1. How true or false is each of the following statements for you?

	Defintely true	Mostly true	Not sure	Mostly false	Definitely false
I seem to get sick a little easier than other people	◯	◯	◯	◯	◯
I am as healthy as anybody I know	◯	◯	◯	◯	◯
I expect my health to get worse	◯	◯	◯	◯	◯
My health is excellent	◯	◯	◯	◯	◯

21.

1. These questions are about how you feel and how things have been with you during the past 4 weeks. For each question, please give the answer that comes closest to the way you have been feeling.

How much of the time during the past 4 weeks...

	All of the time	Most of the time	A good bit of the time	Some of the time	A little of the time	None of the time
Did you feel full of pep?	◯	◯	◯	◯	◯	◯
Have you been a very nervous person?	◯	◯	◯	◯	◯	◯
Have you felt so down in the dumps that nothing could cheer you up?	◯	◯	◯	◯	◯	◯
Have you felt calm and peaceful?	◯	◯	◯	◯	◯	◯
Did you have a lot of energy?	◯	◯	◯	◯	◯	◯
Have you felt downhearted and blue?	◯	◯	◯	◯	◯	◯
Did you feel worn out?	◯	◯	◯	◯	◯	◯
Have you been a happy person?	◯	◯	◯	◯	◯	◯
Did you feel tired?	◯	◯	◯	◯	◯	◯

22.

1. How many hours per week are you physically active (for example, walking, working around the house, working out)?

2. How many of those hours represent exercise primarily intended to improve or maintain your health or fitness?

3. If you do any exercise primarily for health or fitness, how would you rate its intensity?

- ◯ Low
- ◯ Medium
- ◯ High

23.

1. In a typical week, how often do you choose your food (the type and/or amount) with health and fitness concerns in mind?

- ◯ Every meal
- ◯ Most meals
- ◯ Some meals
- ◯ Few meals
- ◯ No meals

2. Are you currently following a specific diet plan?

- ◯ Yes
- ◯ No

3. If you are following a specific diet plan, please describe it here:

4. In a typical week, how often do you eat more than you think you should eat?

- ◯ Every meal
- ◯ Most meals
- ◯ Some meals
- ◯ Few meals
- ◯ No meals

24.

1. Do you smoke (including cigarettes, cigars, pipes, or anything else)?

- Yes
- No

2. If you smoke cigarettes, about how many packs do you smoke per week?

25.

1. On average, how many days per week do you drink alcohol?

2. On a day when you drink, about how many alcohol drinks do you typically have?

3. In the past month, what is the maximum number of alcohol drinks you've had in one day (24 hours)?

26.

1. When your doctor gives you a prescription (excluding birth control), do you follow it exactly (for example, by taking all of the medication and taking it on the prescribed schedule)?

- ◯ Always
- ◯ Usually
- ◯ Sometimes
- ◯ Rarely

2. How often do you visit your doctor for a routine check-up or preventive care?

- ◯ Two or more times per year
- ◯ Once per year
- ◯ Less than once per year
- ◯ Never

27.

1. How often do you visit your dentist for a routine check-up or cleaning?

- ◯ Two or more times per year
- ◯ Once per year
- ◯ Less than once per year
- ◯ Never

2. How often do you floss your teeth?

- ◯ Twice or more each day
- ◯ Once each day
- ◯ Most days each week
- ◯ Once or twice each week
- ◯ Rarely or never

28.

1. How tall are you?

	Feet	Inches
Height in feet and inches:		

2. Approximately how much do you currently weigh?

Weight in pounds:

29.

We're now moving on to the next set of questions. These questions deal with your assessments of your own behaviors and tendencies.

30.

1. How would you describe yourself: Are you generally willing to take risks or do you try to avoid taking risks? 0 means "not at all willing" and 10 means "very willing."

○ 0 ○ 1 ○ 2 ○ 3 ○ 4 ○ 5 ○ 6 ○ 7 ○ 8 ○ 9 ○ 10

2. Please indicate the degree to which the following statements describe your general tendencies:

	Very much like me	Somewhat like me	Somewhat unlike me	Not at all like me
**I needlessly delay finishing jobs, even though they are important.**	○	○	○	○
**I postpone starting in on things I don't like to do.**	○	○	○	○
**When I have a deadline, I wait until the last minute.**	○	○	○	○
**I delay making tough decisions.**	○	○	○	○
**I am on time for appointments.**	○	○	○	○

31.

1. Please indicate the degree to which the following statements describe your general tendencies:

	Very much like me	Somewhat like me	Somewhat unlike me	Not at all like me
I am on time for work.	◯	◯	◯	◯
I am on time for social events.	◯	◯	◯	◯
I get going quickly in the morning.	◯	◯	◯	◯
I usually carefully consider the future consequences of my current financial decisions.	◯	◯	◯	◯
I tend to eat healthfully.	◯	◯	◯	◯
I exercise often.	◯	◯	◯	◯

32.

We are now moving on to a set of questions about your relationship with your partner, if applicable. If you are not currently in a relationship, we will proceed to another set of questions. Please select the statement that best describes you below.

1. Are you currently married or living with a romantic partner?

- ◯ Yes
- ◯ No

1. All things considered, compared to the average couple, how happy are you in your present relationship?

○ 1 ○ 2 ○ 3 ○ 4 ○ 5 ○ 6 ○ 7

Very Unhappy Happy Perfectly Happy

2. State the approximate extent of agreement or disagreement between you and your partner on the following items.

	Always Agree	Almost Always Agree	Occasionally Disagree	Frequently Disagree	Almost Always Disagree	Always Disagree
Handling Family Finances	○	○	○	○	○	○
Matters of Recreation	○	○	○	○	○	○
Demonstrations of Affection	○	○	○	○	○	○
Friends	○	○	○	○	○	○
Sexual Relations	○	○	○	○	○	○
Conventionality (right, good, or proper conduct)	○	○	○	○	○	○
Philosophy of Life	○	○	○	○	○	○
Ways of Dealing with In-Laws	○	○	○	○	○	○

34.

1. When disagreements arise, which of these do they usually result in?

- ◯ You giving in
- ◯ Your partner giving in
- ◯ Agreement by mutual give and take

2. How often do you and your partner engage in outside interests together?

- ◯ All of the time
- ◯ Some of the time
- ◯ Very little of the time
- ◯ None of the time

3. In leisure time, which of these do you generally prefer?

- ◯ To be "on the go"
- ◯ To stay at home

4. In leisure time, which of these does your partner generally prefer?

- ◯ To be "on the go"
- ◯ To stay at home

35.

1. Do you ever wish that you had not married, committed to, or moved in with your partner?

- ◯ Frequently
- ◯ Occasionally
- ◯ Rarely
- ◯ Never

2. If you had your life to live over again, which of these would you most likely do?

- ◯ Marry or commit to the same person
- ◯ Marry or commit to a different person
- ◯ Not marry or commit to anyone at all

3. Do you ever confide in your spouse or partner?

- ◯ Almost never
- ◯ Rarely
- ◯ In most things
- ◯ In everything

36.

We are now moving on to a set of questions about personal finances. We'll begin by asking you about your thoughts and feelings regarding your finances.

37.

The questions in this part of the survey ask about your thoughts and feelings about your household's financial situation. There are no right or wrong answers. Please select the answers that best reflect your thoughts and feelings.

1. Using a scale from 0 to 10 where 0 means "the worst possible financial situation" and 10 means "the best possible financial situation," how would you rate your financial situation these days?

◯ 0 ◯ 1 ◯ 2 ◯ 3 ◯ 4 ◯ 5 ◯ 6 ◯ 7 ◯ 8 ◯ 9 ◯ 10

2. Looking back ten years ago, how would you rate your financial situation at that time using the same 0 to 10 scale?

◯ 0 ◯ 1 ◯ 2 ◯ 3 ◯ 4 ◯ 5 ◯ 6 ◯ 7 ◯ 8 ◯ 9 ◯ 10

3. Looking ahead ten years into the future, what do you expect your financial situation will be like at that time?

◯ 0 ◯ 1 ◯ 2 ◯ 3 ◯ 4 ◯ 5 ◯ 6 ◯ 7 ◯ 8 ◯ 9 ◯ 10

4. Using a 0 to 10 scale where 0 means "no control at all" and 10 means very much control," how would you rate the amount of control you have over your financial situation these days?

◯ 0 ◯ 1 ◯ 2 ◯ 3 ◯ 4 ◯ 5 ◯ 6 ◯ 7 ◯ 8 ◯ 9 ◯ 10

38.

1. Using a 0 to 10 scale where 0 means "no thought or effort" and 10 means “very much thought and effort," how much thought and effort do you put into your financial situation these days?

- ◯ 0
- ◯ 1
- ◯ 2
- ◯ 3
- ◯ 4
- ◯ 5
- ◯ 6
- ◯ 7
- ◯ 8
- ◯ 9
- ◯ 10

2. In general, would you say you (and your family living with you) have more money than you need, just enough for your needs, or not enough to meet your needs?

- ◯ More money than you need
- ◯ Just enough money
- ◯ Not enough money

3. How difficult is it for you (and your family living with you) to pay your monthly bills?

- ◯ Very difficult
- ◯ Somewhat difficult
- ◯ Not very difficult
- ◯ Not at all difficult

39.

1. If you were to consult a trustworthy and knowledgeable professional financial advisor, how much of your current financial arrangements do you think that the financial advisor would recommend changing? On the scale below, 0 means "no change" and 10 means "a total overhaul."

◯ 0 ◯ 1 ◯ 2 ◯ 3 ◯ 4 ◯ 5 ◯ 6 ◯ 7 ◯ 8 ◯ 9 ◯ 10

40.

Think of this ladder as representing where people stand in the United States. At the top of the ladder are the people who are the best off – - those who have the most money, the most education and the most respected jobs. At the bottom are the people who are the worst off –- who have the least money, least education, and the least respected jobs or no job. The higher up you are on this ladder, the closer you are to the people at the very top; the lower you are, the closer you are to the people at the very bottom.

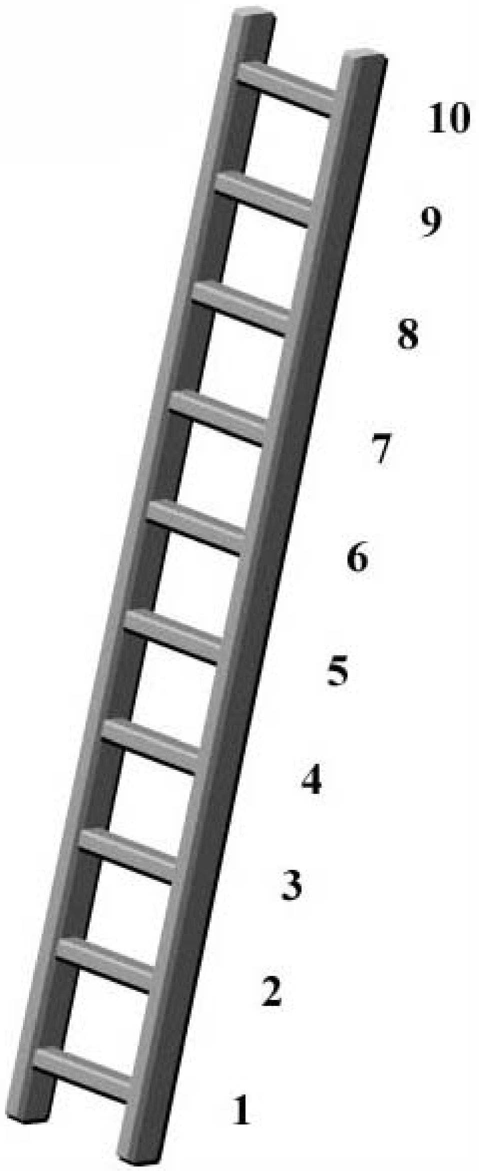

1. Where would you place yourself on this ladder? Please select the number that corresponds to the rung on the ladder where you think you stand at this time in your life, relative to other people in the United States.

- ◯ 10
- ◯ 9
- ◯ 8
- ◯ 7
- ◯ 6
- ◯ 5
- ◯ 4
- ◯ 3
- ◯ 2
- ◯ 1

41.

You are approximately halfway through the survey. We really appreciate your time and thoughtfulness in completing this survey. If you need to, you can take a break at any time and come return to complete the survey at your convenience.

42.

1. Imagine that the interest rate on your savings account was 1% per year and inflation was 2% per year. After 1 year, would you be able to buy more than, exactly the same as, or less than today with the money in this account?

- ◯ More than today
- ◯ Exactly the same as today
- ◯ Less than today

43.

1. If you were to invest money on your own for 10 years, what average return do you think you could earn per year above inflation, in percentage point terms?

- ○ 0 – 3%
- ○ 4 – 6%
- ○ 7 – 9%
- ○ 10 – 12%
- ○ 13 – 15%
- ○ 16 – 20%
- ○ Over 20%

2. Over the same 10-year period, what average return above inflation do you think a typical individual your age could earn, in percentage point terms?

- ○ 0 – 3%
- ○ 4 – 6%
- ○ 7 – 9%
- ○ 10 – 12%
- ○ 13 – 15%
- ○ 16 – 20%
- ○ Over 20%

44.

1. Historically (averaging across several decades), a diversified portfolio of U.S. stocks will increase in value by approximately 10% each year. However, the value tends to go up and down in the short term. Giving your best guess, what do you think will be the most likely outcome for the value of such a portfolio of U.S. stocks in the next year?

- ◯ It will increase by 25% or more
- ◯ It will increase by 15-24%
- ◯ It will increase by 5-14%
- ◯ It will remain about the same (an increase or decline of less than 5%)
- ◯ It will decline by 4-14%
- ◯ It will decline by 15-24%
- ◯ It will decline by 25% or more

45.

1. By next year at this time, what is the percent chance that a diversified portfolio of U.S. stocks will have <u>increased</u> in value by more than 10 percent compared to what they are worth today?

◯ 0% ◯ 10 ◯ 20 ◯ 30 ◯ 40 ◯ 50 ◯ 60 ◯ 70 ◯ 80 ◯ 90 ◯ 100

2. By next year at this time, what is the percent chance that a diversified portfolio of U.S. stocks will have <u>decreased</u> in value by more than 10 percent compared to what they are worth today?

◯ 0% ◯ 10 ◯ 20 ◯ 30 ◯ 40 ◯ 50 ◯ 60 ◯ 70 ◯ 80 ◯ 90 ◯ 100

46.

1. Imagine you are receiving an annuity benefit payment of $1,000 per month. Suppose you had a choice: either you could keep that $1,000 monthly benefit for life, or you could exchange it for a monthly benefit half that size, $500 per month for life, plus you'd get a one-time, lump sum payment. What is the smallest lump sum that you would be willing to accept in exchange for reducing your lifetime benefit by $500 per month?

- ◯ $0 - $25,000
- ◯ $25,001 - $50,000
- ◯ $50,001 - $75,000
- ◯ $75,001 - $100,000
- ◯ $100,001 - $150,000
- ◯ $150,001 - $200,000
- ◯ More than $200,000
- ◯ Would not take at any price

2. What is the primary reason for your decision?

- ◯ I want control over how my money is invested.
- ◯ I want access to the money in case I need it.
- ◯ I want to be certain that I have a regular monthly income even if I live a long time.
- ◯ I want to leave the money to others when I die.

47.

The next section presents several scenarios that involve choosing between different sums of money. Many of the questions are quite similar, but they differ in subtle but important ways. Because of this, we ask that you read and consider each one individually.

In the first set of scenarios, we will ask you to select between options like the ones shown below. There will be a number of similar questions in that set of scenarios, each varying in the amount of money that can be gained in options (A) and (B).

Ex.1. Which would you prefer?

(A) a 100% chance of getting $5

(B) a 50% chance of getting $10 and a 50% chance of getting $0

In a second set of scenarios, we will ask you to choose between different sums of money that would be received at different times, such as the following:

Ex.2. Which would you prefer?

(A) getting $10 today

(B) getting $20 30 days from today

There will be a number of similar questions in this set of scenarios as well, each varying in the amount of money that can be gained in options (A) and (B) *and* varying the amount of time that passes before you receive the sum of money.

48.

Please select your responses in these first two sets of scenarios as though you would get the money specified in each one, since you <u>will</u> receive the value specified (in the timeframe specified) in one of the items.

Once your questionnaire has been submitted and processed, you will receive a bonus gift certificate based on your response to one of the game's questions. You will be sent the value of the amount corresponding to your choice on that question in the form of an Amazon.com gift certificate. So, let's say that Ex. 1 on the previous page was the question selected. If you chose option A, you would receive a $5 gift certificate; if you chose option B, we would randomly select (with a 50-50% chance) either a $10 gift certificate or $0.

It helps us if you answer all the questions as truthfully as you can. There are no right and wrong answers here. Which choice you make is a matter of personal preference. Please pay careful attention to the amounts in each question and answer according to your own preferences.

We'll now turn to the first set of scenarios.

49.

In the scenarios below, if you select Choice A, you would have a 100% chance of getting $5. If you select Choice B, you would get one of two outcomes.

1. Which would you prefer?

- ◯ (A) a 100% chance of getting $5
- ◯ (B) a 50% chance of getting $6 and a 50% chance of getting $0

2. Which would you prefer?

- ◯ (A) a 100% chance of getting $5
- ◯ (B) a 50% chance of getting $7 and a 50% chance of getting $0

3. Which would you prefer?

- ◯ (A) a 100% chance of getting $5
- ◯ (B) a 50% chance of getting $8 and a 50% chance of getting $0

4. Which would you prefer?

- ◯ (A) a 100% chance of getting $5
- ◯ (B) a 50% chance of getting $9 and a 50% chance of getting $0

5. Which would you prefer?

- ◯ (A) a 100% chance of getting $5
- ◯ (B) a 50% chance of getting $10 and a 50% chance of getting $0

6. Which would you prefer?

- ◯ (A) a 100% chance of getting $5
- ◯ (B) a 50% chance of getting $11 and a 50% chance of getting $0

7. Which would you prefer?

- ◯ (A) a 100% chance of getting $5
- ◯ (B) a 50% chance of getting $12 and a 50% chance of getting $0

8. Which would you prefer?

- ◯ (A) a 100% chance of getting $5
- ◯ (B) a 50% chance of getting $13 and a 50% chance of getting $0

9. Which would you prefer?

- ◯ (A) a 100% chance of getting $5
- ◯ (B) a 50% chance of getting $14 and a 50% chance of getting $0

50.

In the scenarios below, if you select Choice A, you would have a 100% chance of getting $10. If you select Choice B, you would get one of two outcomes.

1. Which would you prefer?

- ◯ (A) a 100% chance of getting $10
- ◯ (B) a 50% chance of getting $16 and a 50% chance of getting $0

2. Which would you prefer?

- ◯ (A) a 100% chance of getting $10
- ◯ (B) a 50% chance of getting $17 and a 50% chance of getting $0

3. Which would you prefer?

- ◯ (A) a 100% chance of getting $10
- ◯ (B) a 50% chance of getting $18 and a 50% chance of getting $0

4. Which would you prefer?

- ◯ (A) a 100% chance of getting $10
- ◯ (B) a 50% chance of getting $19 and a 50% chance of getting $0

5. Which would you prefer?

- ◯ (A) a 100% chance of getting $10
- ◯ (B) a 50% chance of getting $20 and a 50% chance of getting $0

6. Which would you prefer?

- ◯ (A) a 100% chance of getting $10
- ◯ (B) a 50% chance of getting $21 and a 50% chance of getting $0

7. Which would you prefer?

- ◯ (A) a 100% chance of getting $10
- ◯ (B) a 50% chance of getting $22 and a 50% chance of getting $0

8. Which would you prefer?

- ◯ (A) a 100% chance of getting $10
- ◯ (B) a 50% chance of getting $23 and a 50% chance of getting $0

9. Which would you prefer?

- ◯ (A) a 100% chance of getting $10
- ◯ (B) a 50% chance of getting $24 and a 50% chance of getting $0

51.

In the scenarios below, if you select Choice A, you would have a 100% chance of getting $20. If you select Choice B, you would get one of two outcomes.

1. Which would you prefer?

- ◯ (A) a 100% chance of getting $20
- ◯ (B) a 50% chance of getting $36 and a 50% chance of getting $0

2. Which would you prefer?

- ◯ (A) a 100% chance of getting $20
- ◯ (B) a 50% chance of getting $37 and a 50% chance of getting $0

3. Which would you prefer?

- ◯ (A) a 100% chance of getting $20
- ◯ (B) a 50% chance of getting $38 and a 50% chance of getting $0

4. Which would you prefer?

- ◯ (A) a 100% chance of getting $20
- ◯ (B) a 50% chance of getting $39 and a 50% chance of getting $0

5. Which would you prefer?

- ◯ (A) a 100% chance of getting $20
- ◯ (B) a 50% chance of getting $40 and a 50% chance of getting $0

6. Which would you prefer?

- ◯ (A) a 100% chance of getting $20
- ◯ (B) a 50% chance of getting $41 and a 50% chance of getting $0

7. Which would you prefer?

- ◯ (A) a 100% chance of getting $20
- ◯ (B) a 50% chance of getting $42 and a 50% chance of getting $0

8. Which would you prefer?

- ◯ (A) a 100% chance of getting $20
- ◯ (B) a 50% chance of getting $43 and a 50% chance of getting $0

9. Which would you prefer?

- ◯ (A) a 100% chance of getting $20
- ◯ (B) a 50% chance of getting $44 and a 50% chance of getting $0

52.

In the scenarios below, if you select Choice A, you would have a 100% chance of getting $20. If you select Choice B, you would get one of two outcomes.

1. Which would you prefer?

- (A) a 100% chance of getting $20
- (B) a 50% chance of getting $21 and a 50% chance of getting $19

2. Which would you prefer?

- (A) a 100% chance of getting $20
- (B) a 50% chance of getting $22 and a 50% chance of getting $18

3. Which would you prefer?

- (A) a 100% chance of getting $20
- (B) a 50% chance of getting $23 and a 50% chance of getting $17

4. Which would you prefer?

- (A) a 100% chance of getting $20
- (B) a 50% chance of getting $24 and a 50% chance of getting $16

5. Which would you prefer?

- (A) a 100% chance of getting $20
- (B) a 50% chance of getting $25 and a 50% chance of getting $15

6. Which would you prefer?

- (A) a 100% chance of getting $20
- (B) a 50% chance of getting $26 and a 50% chance of getting $14

7. Which would you prefer?

- (A) a 100% chance of getting $20
- (B) a 50% chance of getting $27 and a 50% chance of getting $13

8. Which would you prefer?

- (A) a 100% chance of getting $20
- (B) a 50% chance of getting $28 and a 50% chance of getting $12

9. Which would you prefer?

- (A) a 100% chance of getting $20
- (B) a 50% chance of getting $29 and a 50% chance of getting $11

53.

We are now going to present you with a second set of scenarios. As in the last section, the bonus amount you receive may be based on your response to one of these questions. Again, there are no right or wrong choices, since we are simply measuring your personal preferences.

Unlike the last set, these questions will specify not just an amount of money, but also a period of time that you would be willing to wait before receiving your reward.

54.

In the scenarios below, if you select Choice A, you would get the dollar amount specified today. If you select Choice B, you would get the larger dollar amount thirty days from today.

1. Which would you prefer?

- ◯ (A) getting $10 today
- ◯ (B) getting $20 thirty days from today

2. Which would you prefer?

- ◯ (A) getting $10 today
- ◯ (B) getting $21 thirty days from today

3. Which would you prefer?

- ◯ (A) getting $12 today
- ◯ (B) getting $29 thirty days from today

4. Which would you prefer?

- ◯ (A) getting $11 today
- ◯ (B) getting $21 thirty days from today

5. Which would you prefer?

- ◯ (A) getting $12 today
- ◯ (B) getting $27 thirty days from today

6. Which would you prefer?

- ◯ (A) getting $12 today
- ◯ (B) getting $20 thirty days from today

7. Which would you prefer?

- ◯ (A) getting $10 today
- ◯ (B) getting $14 thirty days from today

8. Which would you prefer?

- ◯ (A) getting $11 today
- ◯ (B) getting $25 thirty days from today

9. Which would you prefer?

- ◯ (A) getting $12 today
- ◯ (B) getting $15 thirty days from today

10. Which would you prefer?

- ◯ (A) getting $11 today
- ◯ (B) getting $20 thirty days from today

11. Which would you prefer?

- ◯ (A) getting $12 today
- ◯ (B) getting $23 thirty days from today

12. Which would you prefer?

◯ (A) getting $11 today ◯ (B) getting $12 thirty days from today

13. Which would you prefer?

◯ (A) getting $11 today ◯ (B) getting $16 thirty days from today

14. Which would you prefer?

◯ (A) getting $12 today ◯ (B) getting $25 thirty days from today

15. Which would you prefer?

◯ (A) getting $12 today ◯ (B) getting $17 thirty days from today

16. Which would you prefer?

◯ (A) getting $10 today ◯ (B) getting $13 thirty days from today

17. Which would you prefer?

◯ (A) getting $10 today ◯ (B) getting $23 thirty days from today

18. Which would you prefer?

◯ (A) getting $10 today ◯ (B) getting $27 thirty days from today

19. Which would you prefer?

◯ (A) getting $12 today ◯ (B) getting $13 thirty days from today

20. Which would you prefer?

◯ (A) getting $10 today ◯ (B) getting $25 thirty days from today

55.

In the scenarios below, if you select Choice A, you would get the dollar amount specified thirty days from today. If you select Choice B, you would get the larger dollar amount sixty days from today.

1. Which would you prefer?

- ◯ (A) getting $10 thirty days from today
- ◯ (B) getting $21 sixty days from today

2. Which would you prefer?

- ◯ (A) getting $12 thirty days from today
- ◯ (B) getting $27 sixty days from today

3. Which would you prefer?

- ◯ (A) getting $12 thirty days from today
- ◯ (B) getting $29 sixty days from today

4. Which would you prefer?

- ◯ (A) getting $10 thirty days from today
- ◯ (B) getting $16 sixty days from today

5. Which would you prefer?

- ◯ (A) getting $12 thirty days from today
- ◯ (B) getting $15 sixty days from today

6. Which would you prefer?

- ◯ (A) getting $11 thirty days from today
- ◯ (B) getting $13 sixty days from today

7. Which would you prefer?

- ◯ (A) getting $11 thirty days from today
- ◯ (B) getting $18 sixty days from today

8. Which would you prefer?

- ◯ (A) getting $12 thirty days from today
- ◯ (B) getting $18 sixty days from today

9. Which would you prefer?

- ◯ (A) getting $11 thirty days from today
- ◯ (B) getting $15 sixty days from today

10. Which would you prefer?

- ◯ (A) getting $12 thirty days from today
- ◯ (B) getting $20 sixty days from today

11. Which would you prefer?

- ◯ (A) getting $10 thirty days from today
- ◯ (B) getting $18 sixty days from today

12. Which would you prefer?

- (A) getting $11 thirty days from today
- (B) getting $29 sixty days from today

13. Which would you prefer?

- (A) getting $12 thirty days from today
- (B) getting $14 sixty days from today

14. Which would you prefer?

- (A) getting $11 thirty days from today
- (B) getting $12 sixty days from today

15. Which would you prefer?

- (A) getting $12 thirty days from today
- (B) getting $25 sixty days from today

16. Which would you prefer?

- (A) getting $12 thirty days from today
- (B) getting $16 sixty days from today

17. Which would you prefer?

- (A) getting $11 thirty days from today
- (B) getting $25 sixty days from today

18. Which would you prefer?

- (A) getting $11 thirty days from today
- (B) getting $17 sixty days from today

19. Which would you prefer?

- (A) getting $11 thirty days from today
- (B) getting $23 sixty days from today

20. Which would you prefer?

- (A) getting $12 thirty days from today
- (B) getting $21 sixty days from today

56.

We are now going to present you with a third type of scenario. These questions involve either being certain that you will neither gain nor lose money, or taking a chance that you might either gain or lose money.

Unlike the last section, the scenarios from here on are hypothetical. You will not actually be paid as a result of your choices and you will not lose any money, but please read each scenario carefully and select as though you would lose or gain the money specified. There are no right or wrong choices, since we are simply measuring your personal preferences.

57.

In the scenarios below, if you select Choice A, you would have a 100% chance of getting $0. If you select Choice B, you would get one of two outcomes. You would have a 50% chance of gaining the dollar amount specified and a 50% chance of losing $5.

1. Which would you prefer?

- ◯ (A) a 100% chance of getting $0
- ◯ (B) a 50% chance of gaining $1 and a 50% chance of losing $5

2. Which would you prefer?

- ◯ (A) a 100% chance of getting $0
- ◯ (B) a 50% chance of gaining $2 and a 50% chance of losing $5

3. Which would you prefer?

- ◯ (A) a 100% chance of getting $0
- ◯ (B) a 50% chance of gaining $3 and a 50% chance of losing $5

4. Which would you prefer?

- ◯ (A) a 100% chance of getting $0
- ◯ (B) a 50% chance of gaining $4 and a 50% chance of losing $5

5. Which would you prefer?

- ◯ (A) a 100% chance of getting $0
- ◯ (B) a 50% chance of gaining $5 and a 50% chance of losing $5

6. Which would you prefer?

- ◯ (A) a 100% chance of getting $0
- ◯ (B) a 50% chance of gaining $6 and a 50% chance of losing $5

7. Which would you prefer?

- ◯ (A) a 100% chance of getting $0
- ◯ (B) a 50% chance of gaining $7 and a 50% chance of losing $5

8. Which would you prefer?

- ◯ (A) a 100% chance of getting $0
- ◯ (B) a 50% chance of gaining $8 and a 50% chance of losing $5

9. Which would you prefer?

- ◯ (A) a 100% chance of getting $0
- ◯ (B) a 50% chance of gaining $9 and a 50% chance of losing $5

58.

In the scenarios below, if you select Choice A, you would have a 100% chance of getting $0. If you select Choice B, you would get on of two outcomes. You would have a 50% chance of gaining the dollar amount specified and a 50% chance of losing $10.

1. Which would you prefer?

- ◯ (A) a 100% chance of getting $0
- ◯ (B) a 50% chance of gaining $6 and a 50% chance of losing $10

2. Which would you prefer?

- ◯ (A) a 100% chance of getting $0
- ◯ (B) a 50% chance of gaining $7 and a 50% chance of losing $10

3. Which would you prefer?

- ◯ (A) a 100% chance of getting $0
- ◯ (B) a 50% chance of gaining $8 and a 50% chance of losing $10

4. Which would you prefer?

- ◯ (A) a 100% chance of getting $0
- ◯ (B) a 50% chance of gaining $9 and a 50% chance of losing $10

5. Which would you prefer?

- ◯ (A) a 100% chance of getting $0
- ◯ (B) a 50% chance of gaining $10 and a 50% chance of losing $10

6. Which would you prefer?

- ◯ (A) a 100% chance of getting $0
- ◯ (B) a 50% chance of gaining $11 and a 50% chance of losing $10

7. Which would you prefer?

- ◯ (A) a 100% chance of getting $0
- ◯ (B) a 50% chance of gaining $12 and a 50% chance of losing $10

8. Which would you prefer?

- ◯ (A) a 100% chance of getting $0
- ◯ (B) a 50% chance of gaining $13 and a 50% chance of losing $10

9. Which would you prefer?

- ◯ (A) a 100% chance of getting $0
- ◯ (B) a 50% chance of gaining $14 and a 50% chance of losing $10

59.

In the scenarios below, if you select Choice A, you would have a 100% chance of getting $0. If you select Choice B, you would get one of two outcomes. You would have a 50% chance of gaining the dollar amount specified and a 50% chance of losing $20.

1. Which would you prefer?

- ◯ (A) a 100% chance of getting $0
- ◯ (B) a 50% chance of gaining $16 and a 50% chance of losing $20

2. Which would you prefer?

- ◯ (A) a 100% chance of getting $0
- ◯ (B) a 50% chance of gaining $17 and a 50% chance of losing $20

3. Which would you prefer?

- ◯ (A) a 100% chance of getting $0
- ◯ (B) a 50% chance of gaining $18 and a 50% chance of losing $20

4. Which would you prefer?

- ◯ (A) a 100% chance of getting $0
- ◯ (B) a 50% chance of gaining $19 and a 50% chance of losing $20

5. Which would you prefer?

- ◯ (A) a 100% chance of getting $0
- ◯ (B) a 50% chance of gaining $20 and a 50% chance of losing $20

6. Which would you prefer?

- ◯ (A) a 100% chance of getting $0
- ◯ (B) a 50% chance of gaining $21 and a 50% chance of losing $20

7. Which would you prefer?

- ◯ (A) a 100% chance of getting $0
- ◯ (B) a 50% chance of gaining $22 and a 50% chance of losing $20

8. Which would you prefer?

- ◯ (A) a 100% chance of getting $0
- ◯ (B) a 50% chance of gaining $23 and a 50% chance of losing $20

9. Which would you prefer?

- ◯ (A) a 100% chance of getting $0
- ◯ (B) a 50% chance of gaining $24 and a 50% chance of losing $20

60.

We're now turning to a different set of questions, although they may seem similar in nature to ones you answered earlier. Like the last set of questions, this set is hypothetical in nature, so you will not be receiving any of the sums of money specified in the questions.

61.

1. Suppose that you unexpectedly inherited one million dollars from a distant relative. You are immediately faced with the opportunity to take a one-time risky, but possibly rewarding investment option that has a 50-50 chance of doubling the money to two million dollars within a month and a 50-50 chance of reducing the money by one-third, to 667 thousand dollars, within a month.

Would you take the risky investment option or not?

- ◯ Yes
- ◯ No

62.

1. Suppose that the chances were 50-50 that the risky investment would double the money to two million dollars and 50-50 that it would cut it in half, to 500 thousand dollars.

Would you take the risky investment option or not?

- ◯ Yes
- ◯ No

63.

1. Suppose that the chances were 50-50 that the risky investment option would double your money to two million dollars and 50-50 that it would reduce it by seventy-five percent, to 250 thousand dollars.

Would you take the risky investment option or not?

- ◯ Yes
- ◯ No

64.

1. Suppose that the chances were 50-50 that the risky investment option would double the money to two million dollars and 50-50 that it would cut it by twenty percent, to 800 thousand dollars.

Would you take the risky investment option or not?

- ◯ Yes
- ◯ No

65.

1. Suppose the chances were 50-50 that the risky investment option would double your money to two million dollars and 50-50 that it would reduce it by ten percent, to 900 thousand dollars.

Would you take the risky investment option or not?

- ○ No
- ○ Yes

66.

The next set of questions asks about the different sources of income you, your spouse or partner, and other family members in your household may have had over the last calendar year. Please consider the income of all household members and any income that may be received by the household as a whole.

If you are not sure of the exact figures, please give your best estimate. If you would like to consult your records to provide more accurate responses, please feel free to do so. We understand that some of the questions might seem detailed. We are trying to get a fairly accurate picture of people's household finances, so we really appreciate your help in providing this information.

We also understand that questions about your finances are personal in nature. Please be assured that we are taking steps to keep your responses secure and confidential. Your responses are being transmitted securely and will not be shared with anyone outside of our research team.

Although we very much appreciate getting full responses, we also respect your decision to not answer certain questions. Therefore, it is also possible to skip questions that you would prefer not to answer and proceed with the remaining questions in the survey.

67.

For the following questions, please respond based on the pre-tax income of all of your household members over the last calendar year. Remember that your responses will remain confidential.

1. In the last calendar year, what was your pre-tax household income from the following sources?

A. Income from wages or salaries:

B. Income from self-employment:

C. Income from public and private pensions:

D. Income from Social Security:

E. Income from unemployment benefits:

F. Income from any other social benefits, assistance, or grants (e.g., veteran's benefits, disability benefits):

G. Income from investments, savings, insurance, or property (including dividends, interest, and rent from rental property):

H. Income from capital gains:

I. Income from any other sources:

68.

1. If you combine the income from all sources, what was the total pre-tax income of your household in the last calendar year? (If you don’t know the exact figure, please give your best estimate.)

2. What was the total pre-tax annual income of your household when you were 35 years old? (If you don’t know the exact figure, please give your best estimate.)

69.

The next set of questions asks about the different types of assets and debts belonging to you, your spouse or partner, and other family members in your household. If you are not sure of the exact figures, please give a rough estimate. If you would like to consult your records to provide more accurate responses, please feel free to do so.

70.

For the following questions, please base your responses on the assets held by your household. Even a rough estimate (e.g., to the nearest $10,000) provides valuable information. Remember that your responses will remain confidential.

1. What is the current balance or total value of financial assets held by your household in the following types of accounts:

Checking accounts

Savings accounts

Money market accounts

Certificates of deposit (CDs)

Tax-deferred retirement accounts (e.g., IRAs, 401(k) accounts, Keogh accounts)

Other tax-deferred savings accounts (e.g., education savings accounts, health savings accounts)

Bonds not included in tax-deferred accounts

Stocks not included in tax-deferred accounts

Other assets not listed above

2. Focusing on the savings that you have invested in stocks, approximately how much is invested in the following sources?

Stock in a company that currently employs you or your spouse/partner

Stock in a company that previously employed you or your spouse/partner

71.

1. On average each year, over the past few (e.g., 3-5) years, what percent of your income have you saved? Please include all forms of saving, including retirement accounts and other savings accounts.

2. People often consult with financial planners and managers to help make their financial decisions. To what extent do you (along with your spouse/partner) make your financial decisions on your own or with the advice of a financial planner or manager?

1	2	3	4	5
Make all the decisions on my/our own		Make some of the decisions on my/our own		A financial planner/manager makes all the decisions

3. On average, about how often do you (or your spouse/partner) buy or sell stocks, bonds, or other investments, or change the allocations in a retirement account?

- More than once per week
- A few times per month
- A few times per year
- Once or more every five years
- Once or more every decade
- Rarely or never
- Not sure

72.

Financial planners often divide people's accumulated savings (including all dividends and interest earned) into stocks, bonds, and short-term assets such as checking accounts, savings accounts, money market accounts, certificates of deposit, short-term Treasury bills, and cash.

Including what is in retirement accounts such as IRAs and 401(k) plans and what is in educational savings accounts as part of your accumulated savings, please answer the following questions about the allocation of your savings among these categories.

1. Approximately what percentage of your savings is held in the following types of assets? (The total should add up to 100%.)

Stocks

Medium- to long-term bonds

Short-term assets (e.g., checking & savings accounts, money market accounts, certificates of deposit, short-term Treasury bills, & cash)

73.

1. In the past calendar year, has the value of your stocks increased, decreased, or remained about the same?

- Increased
- Decreased
- Remained the same
- Don't know

2. If your stocks have either increased or decreased in the past calendar year, please indicate approximately by what percentage they have increased or decreased.

Increased by approximately

Decreased by approximately

74.

1. Do you own or rent your primary residence?

- ◯ Own
- ◯ Rent
- ◯ Other (please specify)

2. If you rent your primary residence, what is the rent per month?

1. Please indicate the <u>total market value</u> for each of the following types of assets. That is, what would they be worth if they were sold today? If you are not sure of the exact figures, please provide a rough estimate.

Your primary residence	
Additional residence(s) or vacation home(s), not including rental properties	
Rental properties	
Other real estate	
Business(es) or farm(s)	
Vehicles (any cars, trucks, boats, trailers, motor homes, airplanes or other vehicles)	
Other assets such as trusts, commodities, valuable art, jewelry, coins, collectibles, or other items	

76.

1. Please indicate the amount you currently owe for any of the following items. If you are not sure of the exact figures, please provide a rough estimate.

- Home mortgage for your primary residence
- Second mortgage, home equity loans or lines of credit, or home improvement loans for your primary residence
- Mortgage(s), home equity loans or lines of credit, or other loans for your secondary residence(s)
- Mortgage(s), home equity loans, lines of credit, or other loans for your rental property
- Other real estate loans
- Business or farm loans
- Vehicle loans
- Credit cards or other revolving accounts (only if you carry forward a balance; do not include balances that are paid off each month)
- Installment loans for major purchases (e.g., appliances or furniture)
- Educational loans
- Other personal loans
- Medical bills
- Health insurance
- Legal fees
- Alimony or child support

2. Please indicate the interest rate for each of those items. If you are not sure of the exact figures, please provide your best estimate.

	0-4%	5-8%	9-12%	13-16%	17-20%	21-24%	>24%
Home mortgage for your primary residence	○	○	○	○	○	○	○
Second mortgage or other loans for your primary residence	○	○	○	○	○	○	○
Mortages or loans for your secondary residence(s)	○	○	○	○	○	○	○
Mortages or loans for your rental property	○	○	○	○	○	○	○
Other real estate loans	○	○	○	○	○	○	○
Business or farm loans	○	○	○	○	○	○	○
Vehicle loans	○	○	○	○	○	○	○
Credit cards (if carrying a balance)	○	○	○	○	○	○	○
Installment loans for major purchases (e.g., appliances or furniture)	○	○	○	○	○	○	○
Medical bills	○	○	○	○	○	○	○
Educational loans	○	○	○	○	○	○	○
Other personal loans	○	○	○	○	○	○	○

77.

1. Have you been denied credit for any type of <u>loan</u> within the last calendar year?

◯ Yes

◯ No

2. Have you been denied credit for any type of <u>credit card</u> within the last calendar year?

◯ Yes

◯ No

3. How many times have you made a late payment on any <u>loans</u> in the last calendar year?

4. How many times have you made a late payment on any <u>credit card bills</u> in the last calendar year?

78.

1. How often are you surprised at how much you’ve charged on your credit cards?

- ◯ Often
- ◯ Sometimes
- ◯ Rarely
- ◯ Never

2. Each month, approximately how much do you owe on revolving credit card accounts?

3. Of that balance, how much do you pay each month?

4. Approximately how much do you spend on credit card interest and late fees each month?

1. Are you enrolled in automatic bill pay programs for your credit cards and other monthly bills?

- ◯ Yes, for all of my credit cards and bills
- ◯ Yes, for most of my credit cards and bills
- ◯ Yes, for some of my credit cards and bills
- ◯ No, not for any of my credit cards and bills

2. Do you use personal accounting software (e.g., Quicken) regularly to help manage your finances?

- ◯ Yes
- ◯ No

3. What sources of information did you use to assist you in answering these questions about your finances? Check all that apply.

- ☐ Account statements (paper or online)
- ☐ Tax returns
- ☐ Personal accounting software (e.g., Quicken or MS Money)
- ☐ I asked someone else
- ☐ I did not use anything
- ☐ Other (please specify)

80. Comment Page

1. If there is any additional information that you think we should know or if you have any comments or suggestions regarding the survey, please write them below.

81. Final Page

BING SCHOOL LONGITUDINAL
RESEARCH PROJECT

You're finished with this survey! Thank you so much for participating in this study! We value your time and appreciate the contribution that you've made to this research project.

If you have any questions or comments regarding this survey, please contact us via e-mail at bingstdy@uw.edu.
