# Supplementary material for: Predicting mid-life capital formation with pre-school delay of gratification and life-course measures of self-regulation
Source: J Econ Behav Organ. Author manuscript; Available in PMC 2021 Jan 8. (PMC7792663; doi:10.1016/j.jebo.2019.08.016)
Supplement: 3 [file NIHMS1543524-supplement-3.pdf]

Online Appendix for Predicting Mid-Life Capital Formation with Pre-School Delay of Gratification and Life-Course Measures of Self-Regulation

Daniel J. Benjamin, David Laibson, Walter Mischel, Philip K. Peake, Yuichi Shoda, Alexandra Steiny Wellsjo, and Nicole L. Wilson

I. Detailed variable definitions and summary statistics: self-regulation 5

A. Rank normalized delay (RND) 5
1. Background and summary of raw wait times 5
2. Brief summary 7
3. Motivation and overview 8
4. Data 10
5. Model 10
6. Calculation of the deviation from the predicted delay 12
7. Calculation of rank normalized delay 12
8. Results 13
Results of analysis using wait time in log seconds: Tobit random effects parameter estimates 13
Summary of predicted wait times from the log normal model by condition 14
Results of analysis using wait time in seconds: Tobit random effects parameter estimates 15
Summary of predicted wait times from the normal model by condition 16
B. Preschool waiting time in previous studies of the Bing longitudinal sample 18
C. RNCCQ index at age 17 (1984 parent-assessment), age 27 (1993 self-assessment), and age 37 (2003 self-assessment) 18
D. Rank normalized self-regulatory index (RNSRI) 21
E. CCQ index in previous studies of the Bing longitudinal sample 22
F. Multiple imputation approach of RNCCQ index for robustness checks 22
1. More formal description of our multiple imputation procedure 23
2. Methodology after generating the imputed datasets 24
G. Age in months at time of original delay experiment 25
H. Sex 26

II. Detailed variable definitions and summary statistics: Economics wave (“survey sample”) ... 27

A. General information about variables from the economics wave 27
B. Net worth 27
C. Permanent income 29
D. Wealth-income ratio 31
E. High interest-rate debt 31
Reported interest paid on debt 33
Distribution of debt and annual amount of interest above 6% across debt categories 33
F. Credit card misuse 34
Component 1: Amount of carried credit card debt 35
Component 2: Annual amount of interest above 6% on high cost credit card debt 35
Component 3: Binary measure of having been denied credit for a credit card in the last year 36
Component 4: Number of late payments in the last year 36
G. Delay choice 37

H. Savings rate 38
I. Financial health 39
J. Educational attainment (years of education) 40
K. Education scale from Ayduk et al (2000) 41
L. Quantitative and verbal SAT scores 42
Quantitative score 42
Verbal score 42
M. Forward-looking behaviors 43
Subgroup 1: Diet, Exercise, and BMI 45
Subgroup 2: Smoking and Drinking Behavior 45
Subgroup 3: Preventative health and dental care 45
Subgroup 4: Agreement with statements about procrastination 46
Subgroup 5: Carefully consider future consequences of current financial decisions 46
N. Social status 47
O. Current emotional state and satisfaction with present relationship 48
P. Age at the time of the economics survey 52
Q. Values of underlying outcome variables 0.5 SD above and below the mean of the transformed variable 53

III. Recruitment of subjects for follow-up assessments in 1984, 1993, 2003, and 2013 54

A. 1984 follow-up (description from Shoda et al. 1990)[3] 54
B. 1993 follow-up (description from Ayduk et al. 2000) 54
C. 2003 follow-up 55
D. 2013 economics follow-up ("survey sample") 55

IV. Statistical methodology (strategy for addressing multiple testing problems) 56

A. Primary analyses 56
B. Secondary analyses 56
C. Priors for Bayesian analyses 56
D. Detailed methodology: Bayesian data analysis 57
Discussion of priors for Bayesian analysis 58
E. Detailed methodology: False discovery rate control 59

V. Supplementary tables 61

A. Primary analyses 61
1. Measure of self-regulation is RNSRI 62
OLS Regressions: coefficients, standard errors, and nominal p-value 62
Indication of significance controlling the false discovery rate at 0.1 63
Bayesian posterior distribution of the coefficients 64
Empirical Wald test 65
2. Measure of self-regulation is RND 66
OLS Regressions: coefficients, standard errors, and nominal *p*-value 66
Indication of significance controlling the false discovery rate at 0.1 67
Bayesian posterior distribution of the coefficients 68
Empirical Wald test 69
B. Secondary analyses 70

1. Secondary outcome variables 70
Measure of self-regulation is RNSRI 71
Measure of self-regulation is RND 73
2. Secondary sets of independent variables 75
Independent variables: RND and RNCCQ (aggregated over ages 17, 27, and 37) 76
Independent variables: RND and separate RNCCQ indices at each age (17, 27, and 37) 78
Independent variables: RND and each of the six subscales of the RNCCQ (averaged across ages) 80
3. Analysis of diagnostic vs. non-diagnostic conditions 83
Sample – diagnostic group only 84
Sample – non-diagnostic group only 85
p-value from z-test that the difference between the average of coefficients in the diagnostic vs. non-diagnostic group is 0 85
Interaction with dummy for diagnostic group 86
C. Ex-post analyses 87
1. Correlation between RND and the RNCCQ 87
2. Relationships between RND and RNCCQ indices at each age (pairwise) 88
3. Average of coefficients from regressions of each question in the RNCCQ index individually and the primary outcomes 90
4. Average of coefficients from regressions of RNCCQ at each age in separate regressions 90
Regression of primary outcomes on age 17 RNCCQ index 91
Regression of primary outcomes on age 27 RNCCQ index 92
Regression of primary outcomes on age 37 RNCCQ index 93
5. Comparison of the Bing Sample over time: compare full sample of 543 to the subsamples surveyed in the economic survey and age ages 17, 27, and 37 94
Number of people reporting in each sample 96
6. BMI as an outcome variable 97
7. Comparison with results from analyses of BMI data obtained in previous follow-ups of the Bing sample 98
8. Correlation of delay and BMI data in diagnostic and non-diagnostic subsamples – analysis resulted from editorial processes 102
9. Delay choice as independent variable 104
10. Primary analyses including age at time of economic survey as additional control variable. 105
11. Relationship between an aggregate index of the primary capital formation variables and measures of self-regulation 107
12. Correlation matrix of all primary outcome variables and components of the RNSRI 109
13. The effects of the normalizing transformations on the correlation between RND and Age 17 (1984) RNCCQ index 109
14. Independent variable is RNCCQ – analysis resulted from editorial processes 111
D. Robustness analyses 113
1. Assume normality (rather than log-normality) of wait times in marshmallow task. Measure of self-regulation is a modified version of RND: expected deviation from predicted wait time in uncensored seconds (rank-normalized). 114
2. Winsorizing RNSRI and RND variables at the 5th and 95th percentiles 115
3. Include RND spline with knots at 33.3rd and 66.7th percentiles 117
4. Robustness to imputation of RNCCQ indices 121

Average of available measures: to construct the RNSRI, we average non-missing RNCCQ indices (i.e., only include the available data years, with no imputation). In the RNSRI, we give the available RNCCQ indices ¾ weight and RND ¼ weight. 121
Multiple imputation (MI): we fill in missing RNCCQ indices using a multiple imputation approach. 122
5. Original delay deviation measure (wait time in seconds minus condition mean wait times in seconds) 123
6. Drop subjects for whom we made assumptions 125
VI. Survey Instrument 127
VII. Pre-registration Document 127
VIII. References 127

I. Detailed variable definitions and summary statistics: self-regulation

A. Rank normalized delay (RND)

1. Background and summary of raw wait times

Children participated in one of 21 different experimental conditions in the original delay of gratification experiments. To operationalize a measure of delay of gratification for the current research, we begin with the raw wait times described below and employ a statistical adjustment strategy that attempts to account for these conditional differences along with both age and sex. The result of these statistical adjustments is a score that reflects the difference between the participants' raw score and the score predicted from age, sex, and experimental condition. The inverse normal transformation is then applied to these difference scores to yield our main dependent variable, rank normalized delay, which preserves the rank ordering of participants with regard to the difference scores, while the intervals between the scores are adjusted to conform to the Gaussian normal distribution.

The following table provides the descriptive summary of the wait times obtained in the original 21 experimental conditions for the entire Bing sample and the follow-up sample used in the current research.

- Raw wait time (measured in seconds) taken from the subject's first delay-of-gratification experiment. Wait time ranges from 1 second to 900 seconds (the maximum possible wait time for the majority of conditions in the study design).
- Wait times are categorized into 21 conditions to account for differences in study designs across subjects (e.g., spontaneous vs. suggested ideation, rewards exposed vs. obscured, type of rewards, type of ideation, etc.).
- One subject had a wait time of 0 seconds. We assume this is measurement error as it must take some non-zero time to alert the experimenter to end the waiting period. We assign this subject a wait time of 1 second, the next lowest observed wait time.
- "Condition" in the table below refers to the condition in the original experiments conducted from the late 1960s to mid 1970s, numbered (roughly) chronologically. In all, there were 13 studies, which contained a combined total of 21 different conditions.
- For children who participated in multiple studies at the Bing School, we used the waiting time from the first delay-of-gratification study. For example, if a child participated in both Mischel & Ebbesen (1970) and Mischel, Ebbesen, & Zeiss (1972), for the purpose of the Bing Longitudinal Project, only the child's waiting time from Mischel & Ebbesen (1970) was used. This is why in the tables below, some of the conditions have small sample sizes, even though in the original experiments, which also included those for whom it was not their first exposure to a delay-of-gratification study, the sample sizes were larger. We focused on each child's first time participating in a delay-of-gratification study because the psychological meaning of the waiting experiences in subsequent studies may be different from their first time. For example, if a child waited for the maximum 15 minutes the first time, s/he now knows that the delay period may be as long as 15 minutes, which may change his/her experience while participating in the second delay-of-gratification study. Similarly, if a child did not wait the first time, s/he may be more determined to wait when given a second chance. These differences did not affect the interpretability of the original experiments because prior participation in a delay-of-gratification study was randomly distributed across conditions. However, in the present study, and as indicated in the preregistration document, we chose to use only the waiting time from each child's first time participating in a delay-of-gratification study.
- Condition type refers to a categorical variable that describes the type of experimental condition along two dimensions: a) whether the rewards were present during delay or not and b) whether children were provided with a specific thought manipulation (instructions on what to think or do

while waiting) or not. In prior work with the Bing cohort, individual differences in delay are shown to be primarily predictive of later outcomes only in situations where children must face the rewards and are not provided with instructions on what to think or do (Cond type 1 in the table).

Raw wait time comparison of survey subsample and full sample, for the 21 delay conditions

Condition	Cond type	Entire Bing Sample			Survey Follow-Up Subsample		
		N	*M* Wait Time	*SD* Wait Time	N	*M* Wait Time	*SD* Wait Time
1	3	5	194.00	394.85	1	3.00	.
2	3	30	304.73	367.09	7	167.86	320.63
3	4	14	349.43	361.86	4	379.00	392.02
4	1	166	381.14	366.41	34	435.82	361.03
5	2	25	401.36	293.15	3	373.33	327.21
6	4	34	411.74	340.60	6	408.50	350.97
7	4	5	412.40	450.33	2	454.00	630.74
8	4	9	422.00	387.67	1	900.00	.
9	3	4	461.00	507.06	1	7.00	.
10	2	134	507.39	368.99	21	654.52	354.84
11	4	9	511.00	345.89	1	389.00	.
12	3	7	518.14	476.29	2	451.50	634.27
13	3	5	549.40	480.30	1	900.00	.
14	4	9	593.00	303.74	3	549.67	347.53
15	4	17	623.76	349.29	3	771.33	222.86
16	3	8	643.63	377.54	5	489.80	413.00
17	4	18	657.06	328.61	7	623.43	382.53
18	2	34	668.82	244.40	9	707.78	185.60
19	3	9	735.33	301.96	1	900.00	.
20	4	5	838.60	137.29	1	900.00	.
21	3	3	900.00	0.00	0	.	.

Note. 103 Ss from Bing Sample have raw wait times but are not assigned to 1 of the 21 conditions because delaying was not self-imposed for these Ss. 7 Ss from the Survey Subsample have raw wait times but are not assigned to 1 of the 21 conditions because delaying was not self-imposed for these Ss. Within the Survey Subsample, there were no Ss assigned to condition 21. 1 S in the Survey Subsample does not have a wait time. Cond type: 1 = No thought manipulation, rewards present; 2 = No thought manipulation; rewards absent; 3 = Thought manipulation, rewards present; 4 = Thought manipulation, rewards absent.

Summary statistics of raw wait time in the survey subsample

Variable	Mean	Std Dev	N	Missing	Total
Raw wait time (seconds)	509.7	369.1	113	0	113
Raw wait time (log seconds)	5.3	2.0	113	0	113

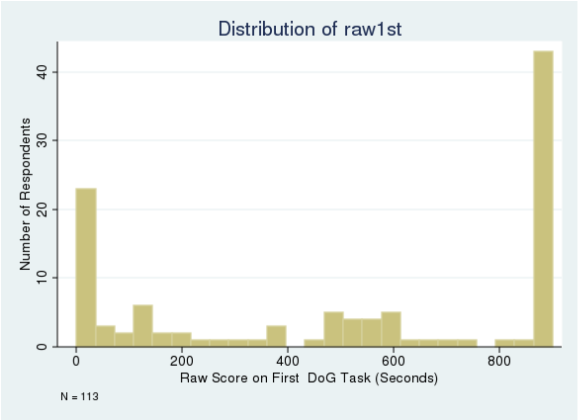

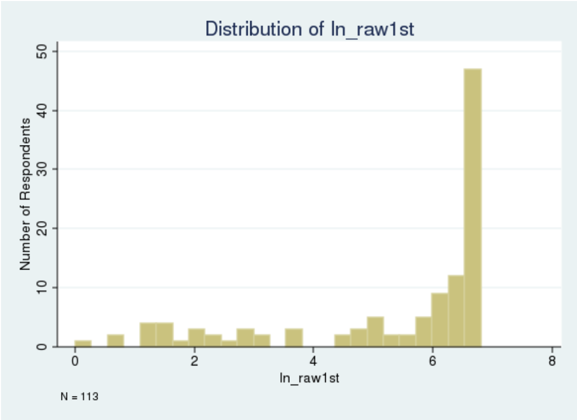

2. Brief summary

Past studies have shown raw wait times differ meaningfully across treatment conditions, by age, and by sex. Therefore we employ a statistical adjustment strategy that calculates each participant's deviation from the wait time predicted by a Tobit random effects model conditional on their age, sex, treatment condition, and accounting for the censoring of wait times at 900 seconds.

Using a random effects model, we take an empirical Bayes approach to estimating a predicted wait time for each subject controlling for his or her age, sex, and experimental condition. We treat the condition effect on wait time as a random effect drawn from a normal distribution. We include age and a male dummy as covariates in the model. The approach is Bayesian in that a prior distribution is used to inform the estimates of the condition effects (the prior matters most in conditions with few subjects). The approach is empirical in the sense that the prior distribution of random effects is estimated from the data. In a simple version of the method (not what we actually use), the estimated mean wait time for a condition is a weighted average of the sample mean of wait time and the estimated overall mean of wait times across all conditions. For conditions with larger N, the estimated mean for the condition is more heavily weighted toward the observed sample mean. In the version of the method we actually use for primary analyses, we assume wait times are log-normally distributed, we use a Tobit random effects model to address the censoring of wait times at 900 seconds, and we control for age and sex in the model.

Note: Rank normalized delay was referred to as preschool waiting time in our preregistration document and is defined as "the inverse normal transformation of the expected deviation from predicted delay time in uncensored log seconds". In addition to rank normalized relay, we also describe "rank normalized delay (seconds)" an alternative version of this variable used in a robustness analysis. Rank normalized delay (seconds) assumes normality of wait times instead of log normality and is calculated as the expected deviation from predicted wait time in uncensored seconds (i.e., without first applying log transformations to the raw waiting times).

3. Motivation and overview

We would like to compare subjects' wait times in the different conditions of the original delay of gratification experiments. We believe wait times differ meaningfully across some experimental conditions (i.e., individuals are expected to wait longer when rewards are covered rather than exposed), by age, and by sex and therefore would like to standardize individual performance in the delay of gratification task across conditions, ages, and sex.

In all prior research exploring longitudinal relations to preschool waiting, wait time was operationalized as the difference between observed wait time (raw seconds) and predicted wait time as represented by the experimental condition mean. This traditional measure is referred to as "delay deviation" in our secondary, ex-post, and robustness analysis. For the current research, we are adopting a more complex operationalization of preschool waiting that we refer to as rank normalized delay.

To predict wait time for each individual, we include a linear effect of age and a male dummy in the regression model. Because we have a small *N* in some conditions, we use a random effects model to estimate an expected effect on wait time for each experimental condition, controlling for age and sex. We assume that the condition effects are drawn from a distribution with an underlying mean and standard deviation. The random effects model predicts a condition effect that is a weighted average of the observed effect of the condition (conditional on age and sex) and the mean of all condition effects (assumed to be 0). Because we are treating the differences between conditions as random effects rather than fixed effects, we estimate only six parameters in the model: a constant term, the effect of being one month older, the effect of being male, the variance of the condition random effects, and the variance of the idiosyncratic error.

We use a Tobit random effects model to account for the fact that the structure of the original experiments imposed a maximum wait time of 900 seconds (or 15 min). The Tobit random effects model accounts for this censoring of wait times and generates estimates of underlying, uncensored wait times.

The distribution of all 543 raw wait times has many observations at a short wait time as well as a long right tail (censored at 900 seconds). We therefore assume that individual wait times are log-normally distributed. The distribution of mean raw wait times in the 21 conditions also appears to have a long right tail, so we also treat the underlying condition means as log-normally distributed. All primary analyses are run using the natural log of wait time in seconds.

For a set of secondary analyses, we test sensitivity of the results to the assumption that wait times are log-normally distributed by repeating the Tobit random effects analysis for wait time in seconds (rather than log-seconds). This analysis provides us with individual deviations from predicted wait times in seconds (given condition, age, and sex), which we use as an alternative independent variable.

Here is the distribution of all 543 raw wait times (seconds and log of seconds):

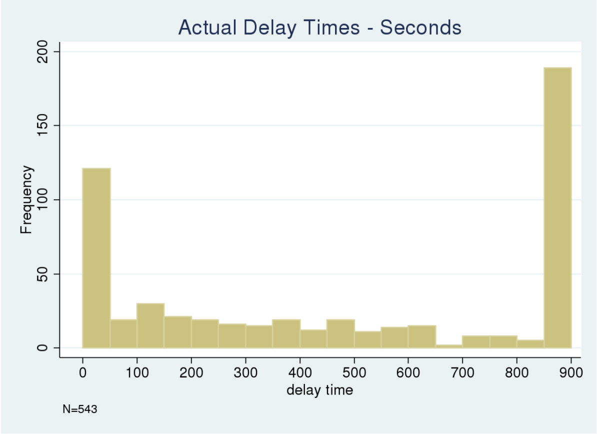

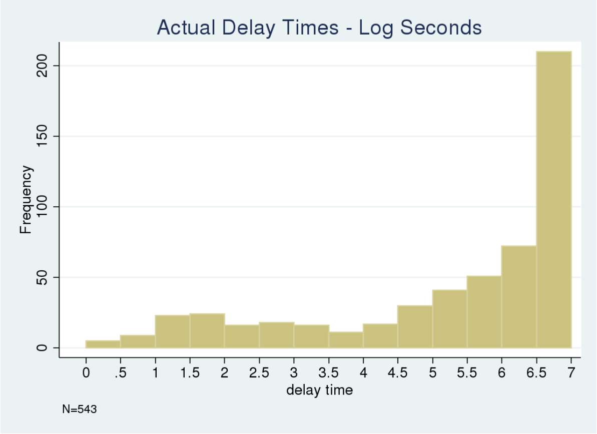

Here is the distribution of 21 Condition Means (mean seconds and log of mean seconds):

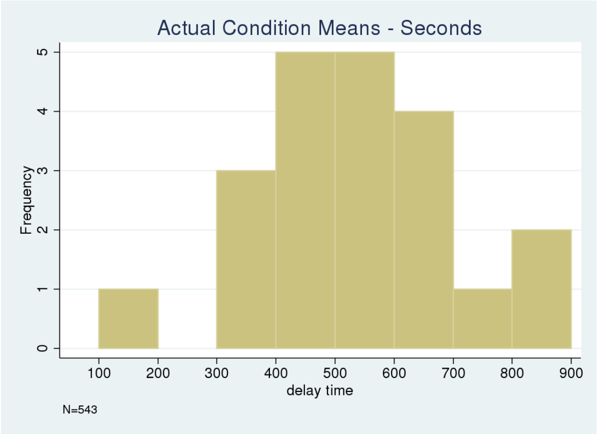

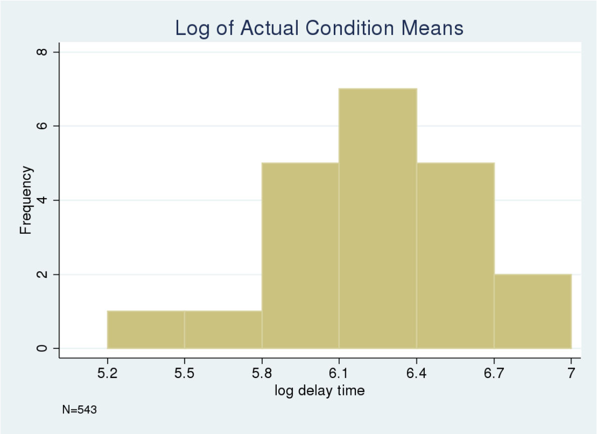

From the random effects model, we predict an expected wait time for each person in uncensored log seconds conditional on their age, sex, and experimental condition.

In a linear case (non-Tobit), the estimated effect on log wait time of being in a condition X can be thought of as a weighted average of 0 (i.e., no effect) and the difference between the sample mean log wait time in condition X and the mean log wait time across all conditions, controlling for age and sex. For conditions with larger *N*, the estimated condition effect is more heavily weighted toward the observed deviation from the underlying mean log wait time. The Tobit random effects model accounts for the censoring of wait and estimates the (latent) uncensored log wait time for each subject.

Finally, we calculate each subject's expected deviation from the wait time predicted by the regression model as ln(subject's actual wait time) – predicted ln(wait time). If the subject's wait time is at the boundary of 900 seconds, we calculate the expected value of the uncensored wait time (given that the subject would have waited at least 900 seconds) and use the estimated uncensored wait time in place of the subject's actual wait time at the boundary. We then apply the inverse normal transformation to this

expected deviation, and this is the variable that is ultimately be used as an independent variable in our key analyses.

4. Data

There are 21 conditions (or groups) with a total of 543 subjects for which wait time, age, and sex data are available (*N* with complete data ranges from 3-165 per condition). The 2012 survey includes a subset of 113 of these subjects across 20 conditions (*N* ranges from 1-34 per condition).

Note: We account for a small number of treatment conditions where the maximum wait time was 10 minutes rather than 15. This affects 8 subjects who waited the full 600 seconds. This differs from the pre-registration plan, in which we assumed all subjects had a maximum wait time of 15 min.

5. Model

There are 21 groups with N_g observations in each group. The natural log of wait time for individual i in group g is y_{gi} and is censored above at ln(900 seconds). The underlying, uncensored, log wait time is y_{gi}^*.

Each individual's uncensored wait time is $y_{gi}^* = \alpha + \beta_1 \times age_{gi} + \beta_2 \times male_{gi} + \eta_g + \varepsilon_{gi}$, where age_{gi} is age in months at the delay task, $male_{gi}$ is a binary indicator of sex, η_g is the group random effect $\sim N(0, {\sigma_\eta}^2)$ and ε_{gi} is the idiosyncratic error $\sim N(0, {\sigma_\varepsilon}^2)$.

The likelihood for each observed wait time y_{gi} in group g consists of two parts:

$$L_{gi} = \begin{cases} \frac{1}{\sigma_\varepsilon} \phi\left(\frac{y_{gi} - \left(\alpha + \beta_1 \times age_{gi} + \beta_2 \times male_{gi} + \eta_g\right)}{\sigma_\varepsilon}\right) & \text{if uncensored} \\ 1 - \Phi\left(\frac{\ln(900) - \left(\alpha + \beta_1 \times age_{gi} + \beta_2 \times male_{gi} + \eta_g\right)}{\sigma_\varepsilon}\right) & \text{if censored} \end{cases}$$

or

$$L_{gi} = \begin{cases} \frac{1}{\sigma_\varepsilon} \phi\left(\frac{y_{gi} - \left(\alpha + \beta_1 \times age_{gi} + \beta_2 \times male_{gi} + \eta_g\right)}{\sigma_\varepsilon}\right) & \text{if uncensored} \\ \Phi\left(\frac{\left(\alpha + \beta_1 \times age_{gi} + \beta_2 \times male_{gi} + \eta_g\right) - \ln(900)}{\sigma_\varepsilon}\right) & \text{if censored} \end{cases}$$

where ϕ is the standard normal probability density function and Φ is the standard normal cumulative distribution function. So, the likelihood contribution for each individual is the probability density of the normal distribution for uncensored observations and the likelihood of being censored if the observed individual is at the boundary.

The likelihood for each group g, integrating over all random effects η_g, is

$$L_g = \int_{-\infty}^{\infty} \left(\prod_{i=1}^{N_g} \left[\Phi\left(\frac{(\alpha + \beta_1 \times age_{gi} + \beta_2 \times male_{gi} + \eta_g) - \ln(900)}{\sigma_\varepsilon} \right) \right]^{B_{gi}} * \left[\frac{1}{\sigma_\varepsilon} \phi\left(\frac{y_{gi} - (\alpha + \beta_1 \times age_{gi} + \beta_2 \times male_{gi} + \eta_g)}{\sigma_\varepsilon} \right) \right]^{1-B_{gi}} \right) \frac{1}{\sigma_\eta} \phi\left(\frac{\eta_g}{\sigma_\eta} \right) d\eta_g$$

where B_{gi} is an indicator for individual i being at the boundary ($y_{gi} = \ln(900)$).

The likelihood function is maximized with the Stata function GLLAMM using adaptive quadrature, a technique for approximating specific integrals with a weighted sum of function values at some specified points (Rabe-Hesketh, Skrondal, and Pickles (2004) and Skrondal and Rabe-Hesketh (2004)).

We obtain maximum likelihood estimates of α, the constant, β_1, the coefficient on age, β_2, the coefficient on the male dummy, ${\sigma_\eta}^2$, the variance of the condition random effects, and ${\sigma_\varepsilon}^2$, the idiosyncratic variance.

The condition effects on wait times (i.e., η_g) are not directly estimated in the random effects model. We use the Stata GLLAMM procedure to obtain an empirical Bayes prediction of the condition expected wait time (in log seconds). The empirical Bayes predictor is the expected value of the posterior distribution of random effects, $p(\eta_g | \mathbf{y}; \widehat{\boldsymbol{\theta}})$ which treats the parameter estimates $\widehat{\boldsymbol{\theta}}$ (the vector of estimates for $\alpha, \beta_1, \beta_2, {\sigma_\eta}^2, {\sigma_\varepsilon}^2$) as known, in addition to the observed wait times $\mathbf{y}$.

If $p(\eta_g | \widehat{\boldsymbol{\theta}})$ is the prior distribution of the random effects before "seeing" the data for group g and $p(y_{gi} | \eta_g, \widehat{\boldsymbol{\theta}})$ is the likelihood contribution for each individual y_{gi} given $\eta_g, \widehat{\boldsymbol{\theta}}$, then the posterior distribution of the random effects is

$$p(\eta_g | \mathbf{y}; \widehat{\boldsymbol{\theta}}) = \frac{p(\mathbf{y}, \eta_g | \widehat{\boldsymbol{\theta}})}{p(\mathbf{y} | \widehat{\boldsymbol{\theta}})} = \frac{p(\eta_g | \widehat{\boldsymbol{\theta}}) \prod_{i=1}^{N_g} p(y_{gi} | \eta_g, \widehat{\boldsymbol{\theta}})}{\int_{-\infty}^{\infty} p(\eta_g | \widehat{\boldsymbol{\theta}}) \prod_{i=1}^{N_g} p(y_{gi} | \eta_g, \widehat{\boldsymbol{\theta}}) \, d\eta_g}$$

Then the empirical Bayes estimate of the random effect η_g is

$${\eta_g}^{EB} = E[\eta_g | \mathbf{y}; \widehat{\boldsymbol{\theta}}] = \frac{\int_{-\infty}^{\infty} \eta_g p(\eta_g | \widehat{\boldsymbol{\theta}}) \prod_{i=1}^{N_g} p(y_{gi} | \eta_g, \widehat{\boldsymbol{\theta}}) \, d\eta_g}{\int_{-\infty}^{\infty} p(\eta_g | \widehat{\boldsymbol{\theta}}) \prod_{i=1}^{N_g} p(y_{gi} | \eta_g, \widehat{\boldsymbol{\theta}}) \, d\eta_g}$$

For the specific case that we use—the Tobit random effects model—the empirical Bayes estimate for the random effect is

$${\eta_g}^{EB} = \frac{\int_{-\infty}^{\infty} \eta_g \frac{1}{\sigma_\eta} \phi\left(\frac{\eta_g}{\sigma_\eta}\right) \left\{ \prod_{i=1}^{N_g} \left[\Phi\left(\frac{\hat{y}_{gi} - \ln(900)}{\sigma_\varepsilon} \right) \right]^{B_{gi}} \left[\frac{1}{\sigma_\varepsilon} \phi\left(\frac{y_{gi} - \hat{y}_{gi}}{\sigma_\varepsilon} \right) \right]^{1-B_{gi}} \right\} d\eta_g}{\int_{-\infty}^{\infty} \frac{1}{\sigma_\eta} \phi\left(\frac{\eta_g}{\sigma_\eta}\right) \left\{ \prod_{i=1}^{N_g} \left[\Phi\left(\frac{\hat{y}_{gi} - \ln(900)}{\sigma_\varepsilon} \right) \right]^{B_{gi}} \left[\frac{1}{\sigma_\varepsilon} \phi\left(\frac{y_{gi} - \hat{y}_{gi}}{\sigma_\varepsilon} \right) \right]^{1-B_{gi}} \right\} d\eta_g}$$

where $\hat{y}_{gi} = \alpha + \beta_1 \times age_{gi} + \beta_2 \times male_{gi} + \eta_g$

(and this formula is calculated using the estimates for $\alpha, \beta_1, \beta_2, {\sigma_\eta}^2, {\sigma_\varepsilon}^2$). The empirical Bayes estimates for the random effects are outputted automatically from the GLLAMM post-estimation procedure.

6. Calculation of the deviation from the predicted delay

Deviation from predicted delay = expectation of actual uncensored log wait time – empirical Bayes prediction of the uncensored log wait time

If not at the boundary, the expectation of actual uncensored log wait time is the log of observed wait time.

If at the boundary, the expectation of actual uncensored log wait time is the expected value of the uncensored log wait time given the subject waited the full 900 seconds. The uncensored wait times are assumed to be log normally distributed with mean equal to the empirical Bayes predicted log wait time for that subject and standard deviation equal to the idiosyncratic standard deviation of the log wait times. The expectation of uncensored delay given the subject is observed at the boundary are calculated in Stata by simulation. We generate normally distributed data (10,000 observations) using the empirical Bayes predicted uncensored log wait time for the subject and idiosyncratic standard deviation. The expected wait time given the subject is at the boundary is the mean of all observations in the simulated data above the boundary.

7. Calculation of rank normalized delay

Finally, we apply the inverse normal transformation (INT) to the deviation from predicted delay in order to create the measure of rank normalized delay.

In general, the data for our outcome and self-regulation variables do not appear to be normally distributed. Rather than making different parametric assumptions for each variable (e.g., that wealth is log-normally distributed), we instead apply the rank-order inverse normal transformation (INT) to all variables. With the INT, we first calculate the rank-order of the subject within the sample and then use the inverse normal cdf to fit the ranks to a standard normal distribution. The transformation alters the scaling of the original variables and may therefore affect the relationship with other variables. However, our transformation ensures that variables are approximately normal by construction and minimizes the effects of outliers. Because the INT relies on rank-order, the variables in our analyses represent a normalized measure of relative standing. We refer to the transformed variables with the prefix RN. The methodology for calculating the inverse normal transformation (or rank normalization) is as follows:

- Calculate percentile rank (0,1)
    - Rank each observation from 1 to N (1 is lowest, equal observations are assigned the average rank)
    - We use Hazen's percentile rank = (Rank – 0.5)/N
- Transform the percentile rank scores into z-scores (standard normal scores) using an inverse normal function. The z-scores are normally distributed with mean equal to zero and standard deviation of one.

8. Results

Results of analysis using wait time in log seconds: Tobit random effects parameter estimates

	Delay in Log-Seconds
β_1: Age at Delay (months)	0.081***
	(0.020)
β_2: Male	-0.673***
	(0.227)
α: Constant	2.289**
	(1.071)
SD of Idiosyncratic Error	
σ_ε	2.469***
	(0.101)
SD of Random Effect	
σ_η	0.786***
	(0.218)
N	543

Standard errors in parentheses
* $p < 0.1$, ** $p < 0.05$, *** $p < 0.01$

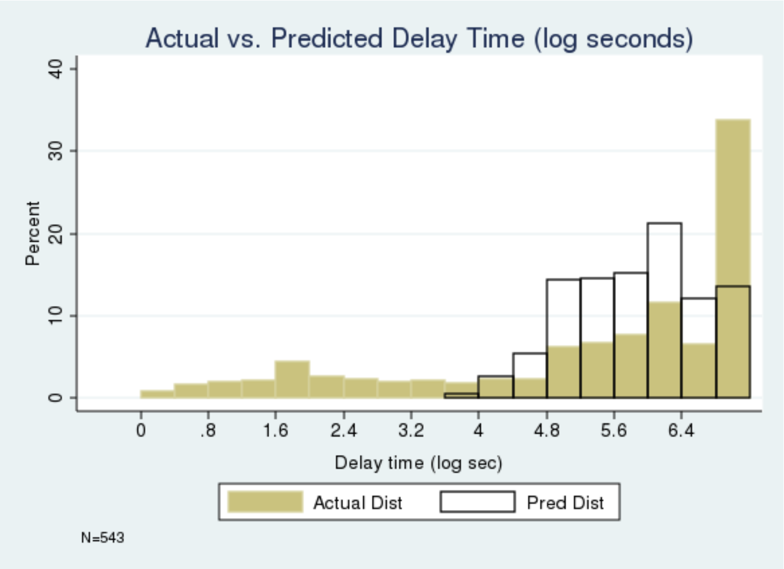

Summary of predicted wait times from the log normal model by condition

Condition	*N*	Actual Data: Mean of wait time (sec)	Actual Data: Mean of log wait time (log sec)	Estimated: Mean of predicted (latent) log wait times (log sec)	Estimated: Mean of expected value at boundary for subjects at 900s (log sec)
1	5	194.00	3.37	5.28	8.31
2	30	304.73	4.24	4.90	8.28
3	14	349.43	4.94	5.64	8.57
4	165	382.56	4.74	5.21	8.35
5	25	401.36	5.57	5.97	8.48
6	33	424.06	5.39	5.91	8.48
7	5	412.40	5.01	6.13	8.57
8	8	474.00	5.62	6.21	8.60
9	4	461.00	4.79	6.12	8.77
10	133	504.44	5.43	6.11	8.59
11	7	523.00	5.99	6.42	8.86
12	7	518.14	4.67	5.84	8.48
13	5	549.40	5.06	6.29	8.72
14	9	593.00	6.24	6.65	8.85
15	17	623.76	5.79	6.50	8.62
16	8	643.63	5.80	6.66	8.80
17	18	657.06	6.23	7.05	8.93
18	34	668.82	6.41	7.47	9.05
19	8	714.75	6.38	7.08	8.83
20	5	838.60	6.72	7.28	8.89
21	3	900.00	6.80	7.04	8.87

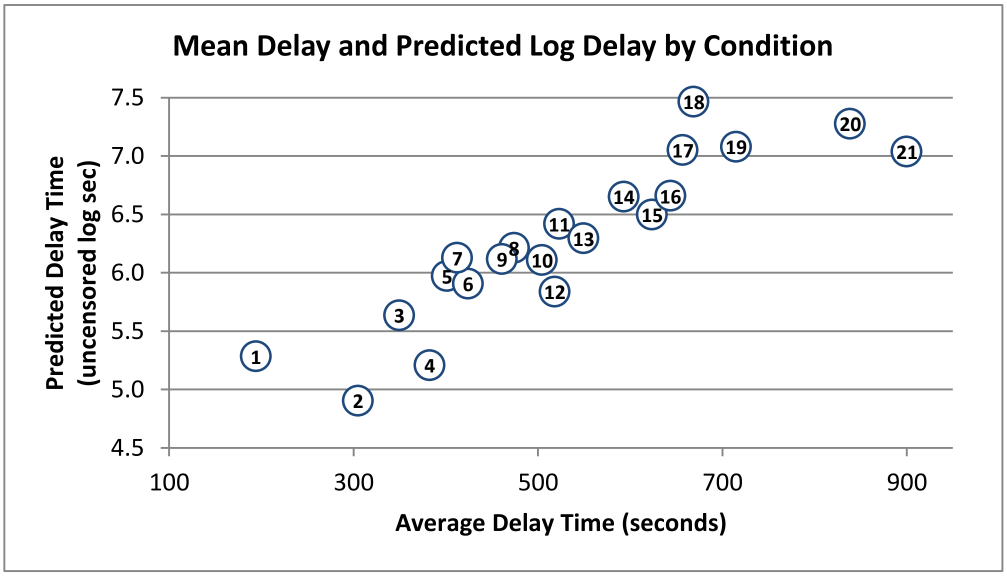

Results of analysis using wait time in seconds: Tobit random effects parameter estimates

	Wait Time in Seconds
β_1: Age at Delay Task (months)	15.753***
	(4.002)
β_2: Male	-123.960***
	(45.677)
α: Constant	-119.266
	(215.060)
SD of Idiosyncratic Error	
σ_ε	499.933***
	(20.932)
SD of Random Effect	
σ_η	154.205***
	(45.428)
N	543

Standard errors in parentheses
* $p < 0.1$, ** $p < 0.05$, *** $p < 0.01$

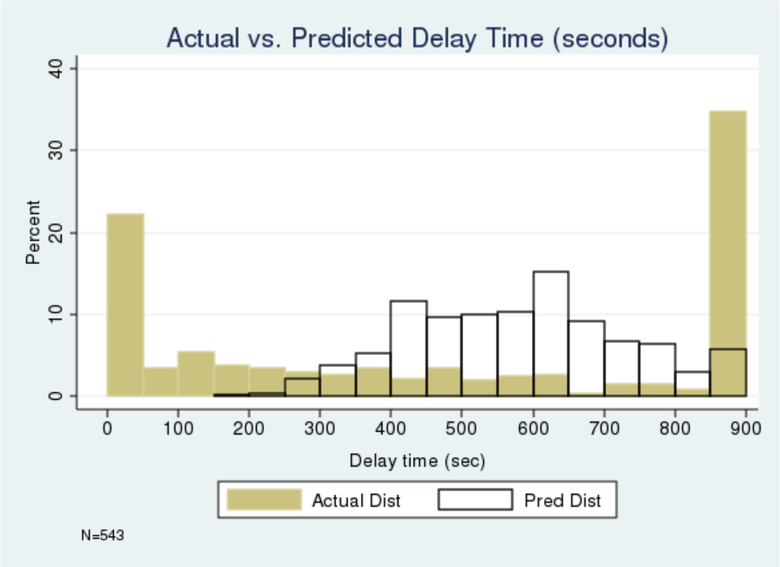

Summary of predicted wait times from the normal model by condition

Condition	*N*	Actual Data: Mean of wait time (sec)	Estimated: Mean of predicted (latent) log wait times (sec)	Estimated: Mean of expected value at boundary for subjects at 900s (sec)
1	5	194	505	1178
2	30	305	425	1178
3	14	349	505	1210
4	165	383	469	1187
5	25	401	512	1184
6	33	424	534	1192
7	5	412	628	1222
8	8	474	617	1219
9	4	461	650	1260
10	133	504	628	1222
11	7	523	653	1261
12	7	518	643	1221
13	5	549	700	1254
14	9	593	696	1253
15	17	624	732	1236
16	8	644	757	1263
17	18	657	796	1272
18	34	669	861	1288
19	8	715	818	1264
20	5	839	871	1279
21	3	900	824	1276

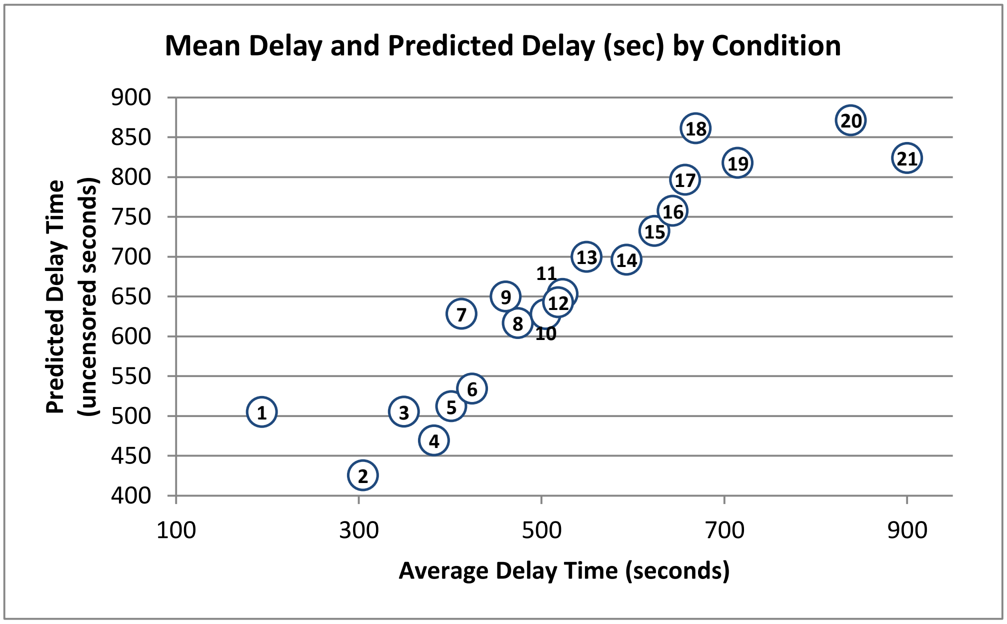

Summary of steps from raw wait time to rank normalized delay

- Step 1: Transform raw wait times using the log transformation
- Step 2: Estimate a Tobit random effects model on log wait times
- Step 3: Simulate expected actual uncensored log wait time and calculate empirical Bayes prediction of the uncensored log wait time
- Step 4: Calculate deviation from the predicted wait time as actual minus expected wait time
- Step 5: Transform deviation using the inverse normal transformation to get rank normalized delay (RND)

Summary statistics for the survey subsample – inverse normal transformation applied to deviation from the predicted wait time (in log seconds)

Variable	Mean	Std Dev	N	Missing	Total
Deviation from the predicted wait time	0.08	2.37	113	0	113
Rank normalized delay (RND)	0.00	1.00	113	0	113

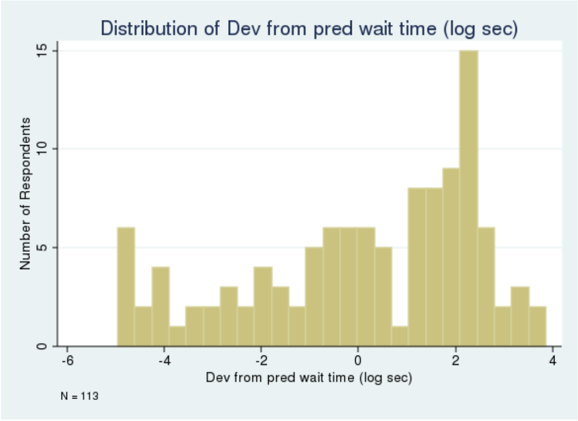

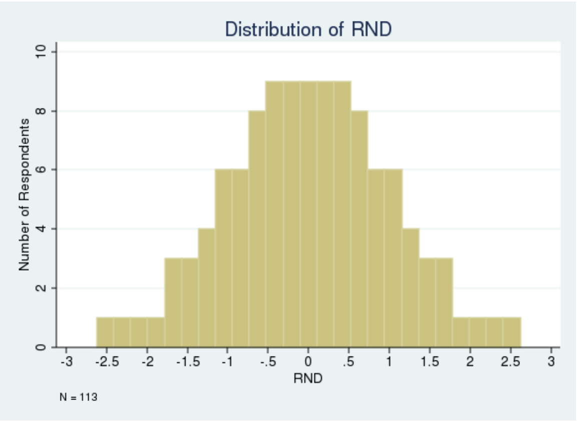

Summary statistics for the survey subsample – inverse normal transformation applied to deviation from the predicted wait time (measured in seconds) calculated for robustness analysis

Variable	Mean	Std Dev	N	Missing	Total
Deviation from the predicted wait time (sec)	40.32	465.08	113	0	113
Rank normalized delay (RND sec)	0.00	1.00	113	0	113

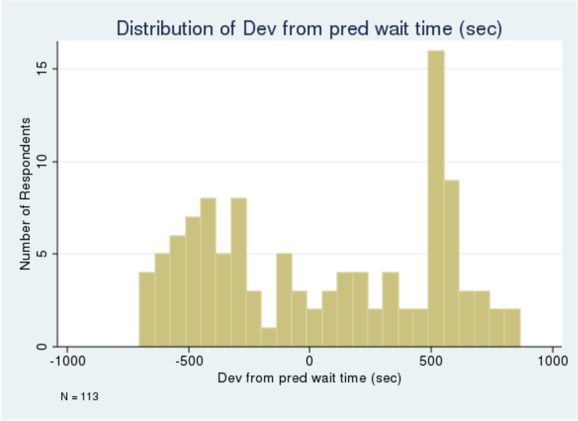

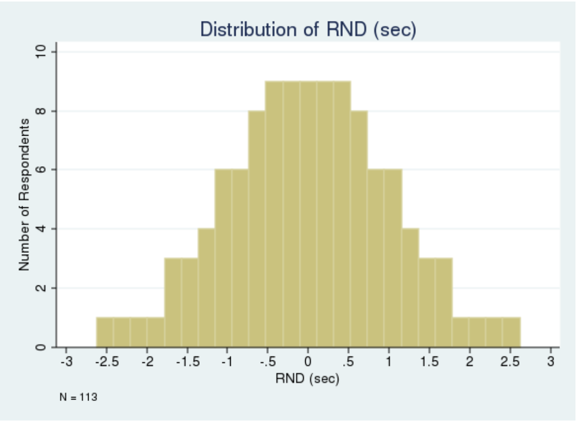

Note that even though the natural log transformation is monotonic and does not affect the rank ordering of individuals (and therefore does not affect the result of applying the inverse normal transformation), in the present case it can potentially change the final rank ordering because we adjust for the waiting condition (as well as their age and sex) before applying the inverse normal transformation.

B. Preschool waiting time in previous studies of the Bing longitudinal sample

- In contrast to the RND as described in Section A above, previous reports from the Bing longitudinal study used a simpler measure of preschool delay behavior.
- In particular, in these previous reports the only adjustment made to raw wait time was to subtract the mean wait time in the subject's experimental condition (e.g., rewards visible to the waiting preschoolers vs. rewards covered and not visible). As described in Section A, there were 21 different types of conditions. For the purpose of examining individual differences among the participants, these condition-to-condition differences constitute unwanted variance. Thus, a given participant's wait time was adjusted by simply subtracting from it the mean wait time for the condition in which the participant waited (which we refer to as Delay Deviation). For example, if a participant waited for 350 seconds in a condition in which children on average waited for 400 seconds, then the participant's Delay Deviation was 350 - 400 = -50, indicating that s/he waited 50 seconds less than the average participant in this condition.
- No other transformation was applied in previous studies.
- In Section C-13 and D-5 of the Supplementary tables, we report the results using this more traditional method for computing a measure of preschool waiting time.

C. RNCCQ index at age 17 (1984 parent-assessment), age 27 (1993 self-assessment), and age 37 (2003 self-assessment)

- Reports of self-control were assessed at three time points using a modified versions of the California Child Q-set (Block & Block, 1980) that was completed by participants' parents with respect to their children in 1984, and by the participants' themselves in 1993, and then again in 2003.
    - Note on CCQ scores in 1984: There were two follow up surveys, one in 1982 and one in 1984. At each follow-up, both parents (i.e., mother and father) were asked to complete the CCQ. So, CCQ scores from 1984 are actually based on between 1 (from 1 parent at one time point) and 4 (from 2 parents at 2 time points) responses. At each time point, the responses were first standardized within each rater (i.e., mother and/or father), and then all responses (again, between 1 and 4 total) were averaged
    - 1993 and 2003 CCQ data were standardized within participants prior to computing CCQ indices
- The CCQ index used in the current report is made up of 31 CCQ items split into 6 subscales (attention, coping, goal pursuit, concern for others, delay, and general cognitive ability). The final index variable was created using expert ratings, responses from 191 Smith college students, and the Bing data in 84, 93, and 03. Because of the transformational strategy used in forming this index, we refer to the resulting dependent measures within each year as the Rank Normalized CCQ (RNCCQ) indices.
- A preliminary set of CCQ items relating to self-control was selected based on three expert ratings of each item in the full q-set for its relevance to aspects of self-control. The 37 items deemed relevant were administered to an independent sample of Smith College students. A principal components analysis revealed that all but one of these items loaded positively on the first

unrotated factor. The remaining 36 items were examined using a confirmatory principal component analysis using ratings obtained in 1984, 1993, and 2003. Five items were eliminated from the analysis due to failure to demonstrate consistent positive loadings on the first unrotated principal component. From the remaining 31 items, two subscales consisting of 3 items each were formed on face value for the items relation to delay of gratification and general cognitive ability. The remaining 25 items were then factor analyzed within the Smith sample using principal axis factoring and oblique rotation noting that we fully expect positive correlations among the subscale. The four oblique factors resulting from this analysis were preliminarily attention, coping, goal pursuit, and concern for others. The item content of these scales as well as the delay and general cognitive ability subscale are listed below:

- Attention Subscale
    - *Is attentive and able to concentrate*
    - *Is planful, thinks ahead*
    - *Is restless and fidgety* (lower score corresponds to higher self-control; reverse scored)
    - *Is easily distracted* (lower score corresponds to higher self-control; reverse scored)
    - *Is reflective; thinks and deliberates before speaking or acting*
    - *Uses and responds to reason*
- Coping Subscale
    - *Can recuperate or recover after stressful experiences*
    - *Tends to withdraw and disengage when under stress* (lower score corresponds to higher self-control; reverse scored)
    - *Tends to go to pieces under stress; becomes rattles and disorganized*
    - *Overreacts to minor frustrations; is easily irritated and/or angered* (lower score corresponds to higher self-control; reverse scored)
    - *Reverts to more immature behavior when under stress* (lower score corresponds to higher self-control; reverse scored)
    - *Tends to become rigidly repetitive or immobilized under stress* (lower score corresponds to higher self-control; reverse scored)
    - *Exhibits self-control when frustrated*
    - *Tends to get sidetracked by minor setbacks or obstacles* (lower score corresponds to higher self-control; reverse scored)
- Goal Pursuit Subscale
    - *Is persistent in activities; does not give up easily*
    - *Is competent, skillful*
    - *Has high standards of performance for self*
    - *Is resourceful in initiating activities*
    - *Becomes strongly involved in the things I do*
    - *Is productive, gets things done*
- Concern for Others Subscale
    - *Shows concern for moral issues (e.g., reciprocity, fairness, and the welfare of others)*
    - *Can be trusted, is dependable*
    - *Is considerate and thoughtful of others*
    - *Is calm and relaxed; easygoing*
    - *Is curious and exploring; eager to learn and open to new experiences*
- Delay Subscale
    - *Settles for smaller but immediately available outcomes rather than pursuing larger ones* (lower score corresponds to higher self-control; reverse scored)

    - *Frequently yields to temptation* (lower score corresponds to higher self-control; reverse scored)
    - *Is unable to delay gratification; cannot wait for satisfactions* (lower score corresponds to higher self-control; reverse scored)
- General Cognitive Ability Subscale
    - *Genuinely values intellectual and cognitive matters*
    - *Has high intellectual capability*
    - *Is verbally fluent; can express ideas well in language*
- The following items were not included in the 1984 follow-up and thus are excluded from the 1984 CCQ index
    - *Is easily distracted*
    - *Exhibits self-control when frustrated*
    - *Tends to get sidetracked by minor setbacks or obstacles*
    - *Is productive, gets things done*
    - *Is considerate and thoughtful of others*
    - *Settles for smaller but immediately available outcomes rather than pursuing larger ones*
    - *Frequently yields to temptation*
    - *Genuinely values intellectual and cognitive matters*
- Note: "Tends to spend extra money rather than save or invest" was excluded from consideration for the index as it is too descriptive of the primary outcome variables
- The items are aggregated in the following way:
    - Each item is transformed using the inverse-normal transformation
    - Transformed items are averaged at the year-subscale level giving equal weight to each available item
    - The subscale score is then transformed using the inverse-normal transformation
    - For subjects with a RNCCQ index in at least one year, missing subscales are imputed from subscale data in available years. For example, to impute a 1984 attention score for someone who has a 1993 and 2003 attention score, we run a regression of 1984 scores on 1993 and 2003 scores and use the estimated coefficients to predict the 1984 attention score for the individual. Similarly, for someone who is missing both 1984 and 1993 attention scores, we predict the 1984 attention score from a regression on 2003 attention scores
    - Imputed scores for each subscale are transformed using the inverse-normal transformation
    - Subscales are then averaged within each year (giving equal weight to each subscale) to obtain aggregate RNCCQ indices in 1984, 1993, and 2003
- For the secondary analyses, we create two aggregate measures:
    - An aggregate RNCCQ index across all three years is created by applying the inverse normal transformation to each year's CCQ index and averaging across the three years, giving each year equal weight
    - We also construct aggregate subscales of the RNCCQ index across all three years by averaging the three transformed subscales, giving each year equal weight (e.g., to create the attention subscale, we average across the 1984, 1993 and 2003 transformed attention subscales)
- 44 subjects have only one measure, 33 have 2 measures, and 33 have all three measures. 3 subjects are missing all three RNCCQ indices and are excluded from this aggregate measure
- 4 subjects are missing one item that makes up the 84, 93, or 03 RNCCQ index
- 1 subject is missing 11 items of the 31 that make up the 03 RNCCQ index

- All 5 have at least one item in each subscale, so subscales are calculated using the average of available items
- Note: in the registration document, "RNCCQ index" was labeled "self-control index"

D. Rank normalized self-regulatory index (RNSRI)

- A key dependent variable for the primary analysis reported here is the RNSRI. This is an index that combines rank normalized delay (RND) derived from the preschool studies and the RNCCQ indices at age 17, 27, and 37. The index is an average of each of the four measures (transformed by the inverse normal transformation), giving equal weight to each measure. The final index is the re-transformed using the inverse normal transformation to obtain the rank normalized self-regulatory index (RNSRI). These are the component parts:
    - Rank normalized delay (RND) - Expected deviation from predicted preschool wait time in uncensored log seconds
    - Age 17 RNCCQ index
    - Age 27 RNCCQ index
    - Age 37 RNCCQ index
- This aggregate measure is only calculated for subjects with at least one CCQ index (so 3 subjects are excluded)

E. CCQ index in previous studies of the Bing longitudinal sample

- The RNCCQ index differs from similar indices used in prior studies. In particular, previous indices were derived from a smaller set of CCQ items, primarily based on face validity, and were not transformed using the INT.
- Similar to the way preschool waiting time was indexed in previous studies (discussed in Section B above), previous publications from the Bing Longitudinal Study computed CCQ-based indices without applying inverse normal transformations (INT), which may amplify small variations as it "corrects" skewed distributions into a normal one. In addition, the application of INT to each of the subscales before averaging them together to form an overall index potentially increases noise when the subscales contain unequal numbers of items. For example, the General Cognitive Ability subscale consists of only 3 items, while the Coping subscale consists of 8 items. Assuming that the amount of noise contained in each item is equal, the General Cognitive Ability scale contains a greater amount of noise compared to the Coping subscale, because the former is an average of only 3 items, while the latter is an average of 8 items. In previous publications from the Bing Longitudinal Study, a CCQ Index was formed by simply averaging all the items without first forming subscales, and without any transformation at any stage of the aggregation process. Thus each item is given the same weight in the process of forming the total score.
- Second, in 1984, there was only one item, CCQ item #65, on the Delay subscale. The other two items in the Delay subscale were added in later years. The fact that there was only one item in this subscale in 1984 potentially makes the issue described above more consequential. In addition, this item contained some very confusing language, making it difficult to see if endorsing this item simply indicates a greater ability to delay gratification, or if endorsing it also implies excessive and unnecessary delay of gratification. In the late 1980s we recognized this problem and removed the confusing language from subsequent follow-ups. On its own, this may not be a serious issue because it involves only one of the many items that make up the overall rank normalized self-regulatory index, but the fact that INT was applied every step of the way (first at the item level, then at the subscale level), and the fact that in 1984 this was the only item in the Delay subscale, amplifies the potential problem.
- Because of these concerns, we examine the effects of the normalizing transformations on the correlation between RND and Age 17 (1984) RNCCQ index in an ex-post analysis reported in Supplementary tables C-13.

F. Multiple imputation approach of RNCCQ index for robustness checks

As a robustness check, test the sensitivity of our results to the imputation method used for missing RNCCQ indices age ages 17, 27, and 37.

To calculate an aggregate RNCCQ index, we would like to (1) normalize the CCQ indices in each year (1984, 1993, and 2003) using the inverse-normal transformation, (2) average the three scores, and (3) re-normalize the final variable to get an aggregate RNCCQ index as measured at ages 17, 27, and 37. To handle missing data in this robustness check, we perform an additional step between steps 1 and 2 to impute missing CCQ scores in each year. All imputation analyses are limited to the 110 subjects with a CCQ index for at least one year.

As a robustness check, we use multiple imputation, a technique that incorporates random error into the imputed value to reflect uncertainty. The general idea of multiple imputation is to generate $m > 1$ different imputed datasets, perform the analysis on each of the completed datasets (1 through m), and pool the results of the individual analyses to get a single coefficient and standard error that simultaneously accounts for uncertainty in the coefficient estimates and uncertainty in the imputed values. Like other imputation methods, multiple imputation assumes that the data are missing at random conditional on

observed variables, i.e., that the probability that the data are missing does not depend on unobserved variables.

There are different ways to draw the random error that enters into multiple imputation. We adopt one standard approach, in which it is assumed that the variables—those with missing values (the CCQ measures) and those used to predict the variables with missing values—are drawn from a multivariate normal distribution. We view this approach as natural in our context because the inverse-normal transformation that we apply to many of the variables in our analysis ensures that their marginal distributions are normal.

A key modeling decision is which variables to include as predictors of the RNCCQ indices. Best practice is to include *all* variables that play into the ultimate analyses of interest, including the dependent variables (von Hippel 2007)[20]. This is because excluding variables from the imputation that is included in analyses (such as the dependent variables) can lead to coefficient estimates that are biased toward 0.[1] In our case, we specify a multivariate normal imputation model including the three RNCCQ scores, rank normalized delay (both in log-seconds and seconds), sex, all primary outcome variables, and all secondary outcome variables with the exception of SAT scores. All variables are transformed using the inverse-normal transformation with the exception of sex. SAT scores are excluded from the imputation model because the small sample size ($N = 31$) results in parameter estimates that do not converge; consequently, analyses of correlations between imputed CCQ indices and SAT scores may be biased toward 0. We include sex in the model even though it is binary because the multivariate normal imputation procedure has proven to be robust to some types of non-normality, including the presence of binary covariates (e.g., Lee & Carlin 2010). We do not believe inclusion of sex greatly impacts the model, either by model fit or for the prediction of RNCCQ indices.

In the remainder of this section, we describe the multiple imputation procedure in more detail. For a complete treatment, see Little and Rubin (2002), Schafer and Olsen (1998), and the Stata Multiple Imputation Manual.

1. More formal description of our multiple imputation procedure

Data augmentation (DA) is the term used to refer to a Bayesian approach to impute values assuming an underlying multivariate normal model. DA first draws a random imputation of missing data given assumed values of the parameters, and then draws new parameters from a Bayesian posterior distribution based on the observed data and values imputed in the previous step. This process is repeated, and the distribution of parameters converges to a posterior distribution of the parameters that averages over the missing data. The distribution of missing data converges to a predictive distribution.

To be more precise, let the data be $\mathbf{X} = (\mathbf{X_o}, \mathbf{X_m})$ where $\mathbf{X_o}$ is the observed part and $\mathbf{X_m}$ is the missing part from a normal distribution $\Pr(\mathbf{X}|\theta) = N(\beta, \Sigma)$, where θ are the unknown model parameters (conditional

---

[1] It may be counterintuitive to include the dependent variables, but as von Hippel (2007) explains: "imputers sometimes worry that, by including Y in the imputation step, they are assuming something unwarranted about the X-Y relationship. This concern is misplaced. By including Y in the imputations, you are not assuming that Y has any particular relationship with X; the relationship could be positive, negative or zero, and any of these possibilities is accounted for by the imputation model. On the other hand, if you exclude Y from the imputations, you are making an assumption. You are assuming that there is no direct relationship between X and Y".

expectations β and elements of the covariance matrix Σ). The goal is to replace missing values with draws from the predictive distribution of the missing data given the observed data $\Pr(\mathbf{X_m}|\mathbf{X_o})$, which depends on the posterior distribution of the unknown parameters, $\Pr(\theta|\,\mathbf{X_o})$. DA augments the observed data with the latent unobserved missing data to estimate the conditional posterior distribution $\Pr(\theta|\,\mathbf{X_o},\mathbf{X_m})$. For a current $\theta^{(t)}$, we draw $\mathbf{X}_{\boldsymbol{m}}^{(\boldsymbol{t+1})}$ from its conditional predictive distribution, $\Pr\left(\mathbf{X_m}|\mathbf{X_o},\theta^{(t)}\right)$. Next, we draw $\theta^{(t+1)}$ from its conditional posterior distribution given the augmented data Pr $\left(\theta|\mathbf{X_o},\mathbf{X}_{\boldsymbol{m}}^{(\boldsymbol{t+1})}\right)$. This process is repeated until the specified number of imputations has been generated.

To obtain starting values for this procedure, an expectation-maximization (EM) algorithm is used. EM is an iterative method that predicts missing values based on the assumed values for the parameters, uses the predictions to update the parameter estimates, and repeats the process until convergence to ML parameter estimates. For the EM algorithm, we assume a non-informative uniform prior distribution for the model parameters (β,Σ). EM convergence can be measured by the lack of change in the parameter estimates and the resulting log-likelihood. The number of iterations required for convergence is output directly from Stata.

A subset of the draws $\mathbf{X}_{\boldsymbol{m}}^{(\boldsymbol{t})}$ from the DA procedure are ultimately used as the imputed values. Multiple imputations begin after a specified "burn-in" period of *b* iterations. Because each iteration is dependent on the previous iteration, the values for each imputation are taken every *k* iterations, where *k* is the "burn-between" period. DA convergence is more difficult to measure than EM convergence but can be controlled by the burn-in and burn-between periods. The burn-in period, *b*, is set to obtain convergence to a stationary distribution $\Pr(\theta,\mathbf{X_m}|\,\mathbf{X_o})$ prior to recording imputed values. This is typically less that the number of iterations required by EM convergence, so a rule of thumb is to set *b* to a value greater than the reported iterations required for convergence of EM. The burn-between period, *k*, should be set large enough that the autocorrelation for all parameters has died down to 0 by lag *k*. We follow procedures described in the Stata Multiple Imputation manual to select values for *b* and *k*. Imputations are recorded at $t_i = b + (i-1)k$, for $i = 1 - m$.

While more imputation draws is always better in principle, in practice there is a computational cost. Because our multiple imputation procedure is not very computationally intensive, we use *m* = 100, at the high end of values considered sufficient in the literature (Graham et al 2007).

2. Methodology after generating the imputed datasets

After imputing missing values, we re-transform each imputation of the RNCCQ indices using the inverse-normal transformation. Then, we average the three transformed variables within each imputation to get an aggregate RNCCQ index for each imputation run, 1 through *m*. Finally, we apply the inverse-normal transformation again to get the final CCQ index for each individual in each imputation run.

Aggregating across simulation runs, pooled regression coefficients and standard errors are calculated according to Rubin's combination rules (Rubin 1987). In particular, the estimated coefficient is the average of the coefficients estimated in each imputed dataset. With *m* imputations,

$$\hat{\theta} = \frac{1}{m}\sum_{i=1}^{m}\hat{\theta}_i.$$

The variance of the estimate is

$$\mathrm{Var}(\hat{\theta}) = W + \left(1 + \frac{1}{m}\right) B,$$

where W is the mean within-imputation variance and B is the between-imputation variance:

$$W = \frac{1}{m}\sum_{i=1}^{m} W_i \text{ and } B = \frac{1}{m-1}\sum_{i=1}^{m}\left(\hat{\theta}_i - \hat{\theta}\right)^2.$$

G. Age in months at time of original delay experiment

- Subject's age in months when first completed the delay experiment

Summary Statistics for the Survey Subsample

Variable	Mean	Std Dev	N	Missing	Total
Age (months)	51.6	5.6	113	0	113

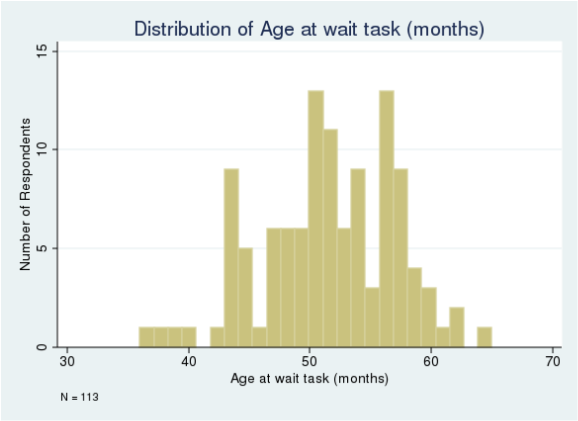

H. Sex

- Binary variable for whether the subject is male
  - *What is your sex? Male/Female*
- We use sex from the original Bing dataset (from original delay experiment) as it is available for all 113 respondents in our analysis. For 111 subjects, this matches sex in the 2012 survey (2 subjects are missing sex in the 2012 survey)

Summary Statistics for the Survey Subsample

**Sex**	**N**	**Percent**
Female	71	63%
Male	42	37%
Total	113	100%

II. Detailed variable definitions and summary statistics: Economics wave ("survey sample")

A. General information about variables from the economics wave

- Many of the survey questions were drawn from the Cognitive Economics Project.
- In general, the data for our outcome and self-regulation variables do not appear to be normally distributed. Rather than making different parametric assumptions for each variable (e.g., that wealth is log-normally distributed), we instead apply the rank-order inverse normal transformation (INT) to all variables. With the INT, we first calculate the rank-order of the subject within the sample and then use the inverse normal cdf to fit the ranks to a standard normal distribution. The transformation alters the scaling of the original variables and may therefore affect the relationship with other variables. However, our transformation ensures that variables are approximately normal by construction and minimizes the effects of outliers. Because the INT relies on rank-order, the variables in our analyses represent a normalized measure of relative standing. We refer to the transformed variables with the prefix RN.
    - The methodology for calculating the inverse normal transformation (or rank normalization) is as follows:
        - Calculate Percentile Rank (0,1)
        - Rank each observation from 1 to N (1 is lowest, equal observations are assigned the average rank)
        - We use Hazen's percentile rank = (Rank – 0.5)/N
    - Transform the percentile rank scores into z-scores (standard normal scores) using an inverse normal function. The z-scores are normally distributed with mean equal to zero and standard deviation of one.

B. Net worth

- Net worth = sum of assets – sum of debt
- Assets
    - *What is the current balance or total value of financial assets held by your household in the following types of accounts: Checking accounts, Savings accounts, Money market accounts, Certificates of deposit (CDs), Tax-deferred retirement accounts (e.g., IRAs, 401(k) accounts, Keogh accounts), Other tax-deferred savings accounts (e.g., education savings accounts, health savings accounts), Bonds not included in tax-deferred accounts, Stocks not included in tax-deferred accounts, Other assets not listed above*
    - *Please indicate the <u>total market value</u> for each of the following types of assets. That is, what would they be worth if they were sold today? If you are not sure of the exact figures, please provide a rough estimate. Your primary residence, Additional residence(s) or vacation home(s), not including rental properties, Rental properties, Other real estate, Business(es) or farm(s), Vehicles (any cars, trucks, boats, trailers, motor homes, airplanes or other vehicles), Other assets such as trusts, commodities, valuable art, jewelry, coins, collectibles, or other items*
- Debt
    - *Please indicate the <u>amount you currently owe</u> for any of the following items. If you are not sure of the exact figures, please provide a rough estimate. Home mortgage for your primary residence, Second mortgage, home equity loans or lines of credit, or home improvement loans for your primary residence, Mortgage(s), home equity loans or lines of credit, or other loans for your secondary residence(s), Mortgage(s), home equity loans, lines of credit, or other loans for your rental property, Other real estate loans, Business or farm loans, Vehicle loans, Credit cards or other revolving accounts (only if you carry forward a balance; do not include balances that are paid off each month),*

Installment loans for major purchases (e.g., appliances or furniture), Educational loans, Other personal loans, Medical bills

- Note: Three respondents were assumed to have filled in monthly mortgage payments rather than total amount owed. All three had high interest rates (6.5%) so we assumed they were unable to re-finance and therefore had <20% home equity. We assumed these respondents had 25 years left on a 30-year loan and were therefore paying almost all interest in their monthly payments. One respondent's implied debt (monthly payments * 12 / interest rate) was more than the value of the home, so we assumed 0% home equity. For the other two respondents, we assume 10% home equity (midpoint between 0 and 20%)
- Note: Two respondents were assumed to fill in monthly vehicle payments rather than total amount owed. We calculate the outstanding balance assuming the respondent is 3 years into a 6-year loan
- Note: One respondent was assumed to fill in monthly education loan payments rather than total amount owed. We calculate the outstanding balance assuming the respondent is 15 years into a 20-year loan
- Adjustments to reported total debt:
    - In calculating total debt, we exclude amount owed on health insurance, legal fees, and alimony
    - We fill in $0 credit card debt for 26 respondents who [in final specifications we will test sensitivity to excluding these respondents]:
        - Reported ≥ $0 debt in other categories but left credit card debt blank and report owing $0 on credit cards each month (N=8)
        - Reported ≥ $0 debt in other categories but left credit card debt blank and pay off 100% of the amount they owe on their credit cards each month (N=18)
        - Note: 58/59 people who reported having 0 credit card debt reported paying 100% of the monthly amount they owe.
    - Filling in $0 for people missing credit card debt as described above leaves 5 people for whom it is not clear whether they do or don't have credit card debt. We leave these subjects as missing credit card debt since we can't make an assumptions about whether they have credit card debt
    - 3 respondents were assumed to fill in monthly mortgage payments (for first and second mortgages) rather than total amount owed
        - All three had high interest rates (6.5%) so we assumed they were unable to re-finance and therefore had <20% home equity. We assumed these respondents had 25 years left on a 30 year loan and were therefore paying almost all interest in their monthly payments
        - One respondent's implied debt (monthly payments × 12 / interest rate) was more than the value of the home, so we assumed 0% home equity. For the other two respondents, we assume 10% home equity (midpoint between 0 and 20%)
        - For the total interest on high cost debt measure, we assume that monthly mortgage payments are almost all interest and therefore calculate interest over 6% as (interest rate – 6% / interest rate) × monthly payment × 12
    - 2 respondents were assumed to fill in monthly vehicle payments rather than total amount owed
        - For total amount owed on vehicle loans, we use the following formula to back out the initial total amount owed on the loan, b, assuming the respondent is 3 years into a 6 year loan: $\int_0^T pe^{-rt}dt = b$, where p = principal, r = interest rate, T = total number of years, i.e., 6.

- Then, we use a debt calculator to determine the outstanding balance after 3 years of payments. http://www.interest.com/debt/calculators/amortization-calculator/
- For interest above 6% on vehicle loans, we use the standard method (interest rate – 6% × total amount owed) using the implied total amount owed as calculated above.

- 1 respondent assumed to fill in monthly education loan payments rather than total amount owed
  - For total amount owed on education loans, we use the following formula to back out the initial total amount owed on the loan, b, assuming the respondent is 15 years into a 20 year loan: $\int_0^T pe^{-rt}dt = b,$ where p = principal, r = interest rate, T = total number of years, i.e., 15.
  - Then, we use a debt calculator to determine the outstanding balance after 15 years of payments. http://www.interest.com/debt/calculators/amortization-calculator/
  - For interest above 6% on education loans, we use the standard method (interest rate – 6% * total amount owed) using the implied total amount owed as calculated above
- Based on the pattern of responses to credit card questions, we assume one respondent switched total amount owed and monthly credit card payment.
- One respondent assumed to report mortgage in thousands of dollars.
- After all adjustments, 93/113 subjects have total debt > 0, 16 have 0 debt, and 4 are missing debt

- 4 respondents are missing net worth: 3 are missing both total assets and total debts and 1 is missing only total debt (we decided not to make an assumption about debt for this individual)
- No respondents have 0 net worth; 3 have negative net worth

Variable	Mean	Std Dev	N	Missing	Total
Net worth (not RN)	1,794,297	3,437,586	109	4	113
Net worth (RN)	0.00	1.00	109	4	113

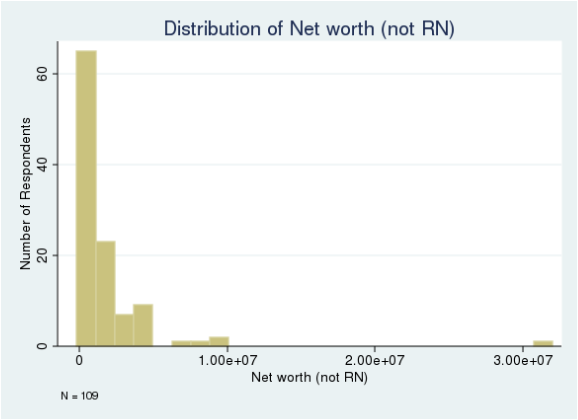

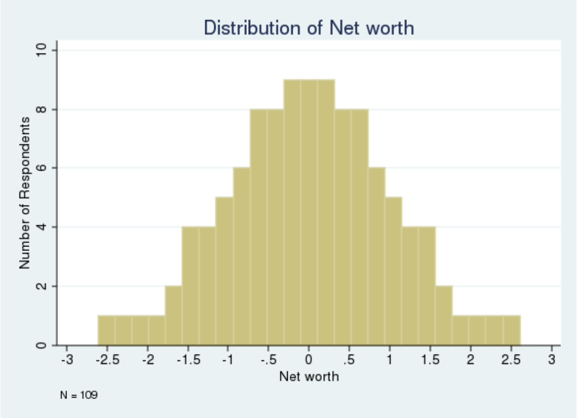

C. Permanent income

- Permanent income is defined as the average of household income in the last calendar year and at age 35 divided by the number of adults living in the household:

$$\frac{\text{Average of household income in the last calendar year and at age 35 (scaled for inflation)}}{\text{Number of adults in the household}}$$

- We use the average of household income in the last calendar year and at age 35 (scaled for inflation) if both are available and non-zero. If either measure is missing or zero, we use only the available, non-zero income
    - Household income in last calendar year is the sum of household income from the following sources:
    - *In the last calendar year, what was your pre-tax household income from the following sources? Income from wages or salaries, Income from self-employment, Income from public and private pensions, Income from Social Security, Income from unemployment benefits, Income from any other social benefits, assistance, or grants (e.g., veteran's benefits, disability benefits), Income from investments, savings, insurance, or property (including dividends, interest, and rent from rental property), Income from any other sources*
- Stated total household income at 35 is scaled for inflation to the last calendar year using the CPI when the respondent was 35 and the CPI in the year prior to when the respondent completed the survey
    - *What was the total pre-tax annual income of your household when you were 35 years old? (If you don't know the exact figure, please give your best estimate.)*
- Number of adults currently living in the household
    - *Including yourself, how many people in each of the following age groups is currently living in your household? Adults*
    - Note: if this question was left blank, we assume two adults if married and one adult if never married or divorced
- If the number of adults is missing or 0, then filled in using the marital status variables
    - If married then adults=2 (N=5); if never married then adults=1 (N=3); if divorced then adults=1 (N=3)
- One respondent is missing aggregated income in last calendar year, so we use stated total income in last calendar year
- Two respondents were assumed to report income in thousands of dollars.
- Two respondents reported \$0 income at 35 (both have > \$0 income in last calendar year). For these, permanent income is calculated using only income in the last calendar year
- One respondent is missing all three income measures and is therefore excluded from analyses of income

Variable	Mean	Std Dev	N	Missing	Total
Permanent income (not RN)	130,738	146,879	112	1	113
Permanent income (RN)	0.00	1.00	112	1	113

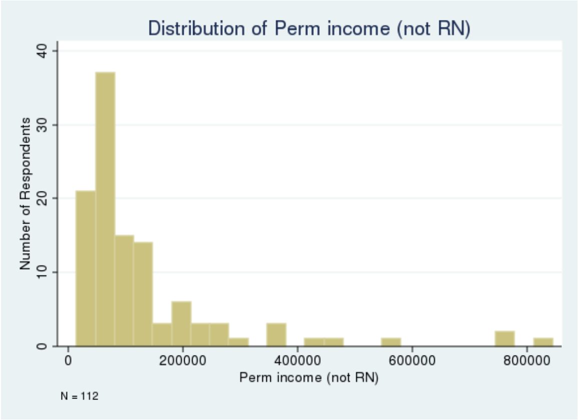

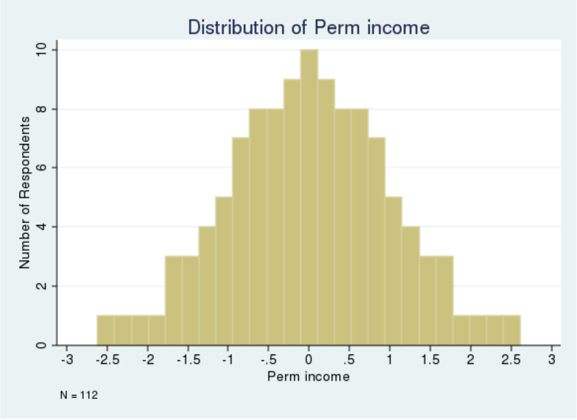

D. Wealth-income ratio

- Net worth divided by permanent income

Variable	Mean	Std Dev	N	Missing	Total
Wealth-income ratio (not RN)	13.5	14.6	109	4	113
Wealth-income ratio (RN)	0.00	1.00	109	4	113

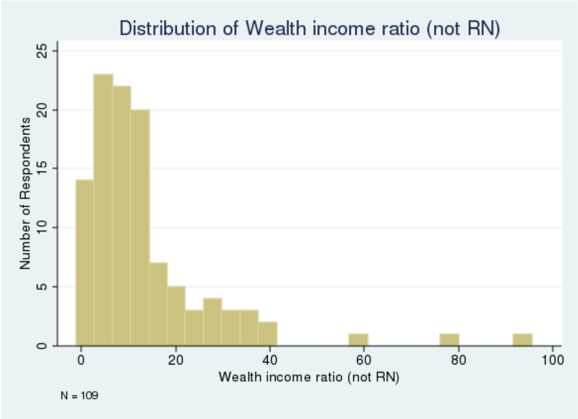

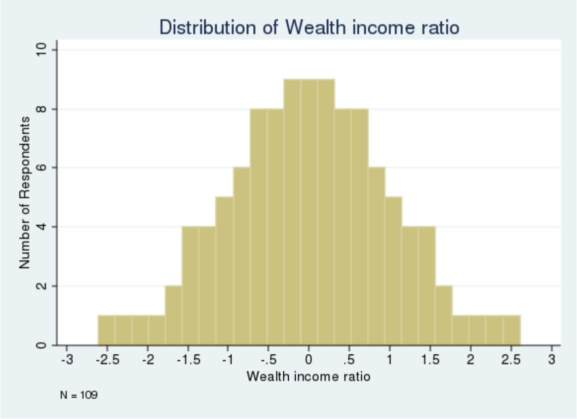

E. High interest-rate debt

- This variable calculates the annual amount of interest above 6% paid on high-interest rate debt
- Respondents selected an interest rate bucket for each type of debt. We assumed the interest rate is the midpoint of the selected category (i.e., if selected 5-8% bucket, the interest rate is assumed to be 6.5%)
- "High interest-rate debt" is any debt for which the interest rate is greater than 6%
  - We chose the 6% interest rate threshold as an interest rate above which there is, for the most part, no good excuse to be paying that high an interest rate. We assume subjects with debt at an interest rate above 6% are either:
    1. Financially incompetent (e.g., have not refinanced their mortgage rates despite low interest rates in the last few years)
    2. In financial distress (e.g., have high leverage ratio on their collateralized debts or have bad credit)
- For each high interest-rate debt category we calculate the amount of interest above 6% as the dollar amount of debt multiplied by the interest rate above 6%

$$\sum_{All\ high\ cost\ debt\ categories} (interest\ rate - 6\%) \times total\ amount\ owed$$

- *Please indicate the <u>amount you currently owe</u> for any of the following items. If you are not sure of the exact figures, please provide a rough estimate. Home mortgage for your primary residence, Second mortgage, home equity loans or lines of credit, or home improvement loans for your primary residence, Mortgage(s), home equity loans or lines of credit, or other loans for your secondary residence(s), Mortgage(s), home equity loans, lines of credit, or other loans for your rental property, Other real estate loans, Business or farm loans, Vehicle loans, Credit cards or other revolving accounts (only if you carry forward a balance; do not include balances that are*

paid off each month), Installment loans for major purchases (e.g., appliances or furniture), Educational loans, Other personal loans, Medical bills

- *Please indicate the* <u>*interest rate*</u> *for each of those items. If you are not sure of the exact figures, please provide your best estimate.*

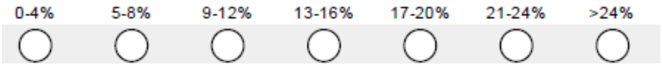

- One respondent's interest rate was reassigned from a mortgage category with no debt to a mortgage category with debt but no corresponding interest rate.
- Note: 5 subjects have non-zero credit card debt at 0-4%
- If a subject has debt > 0 but is missing a corresponding interest rate, we assume the unweighted average reported interest rate among subjects with non-zero debt in the category
- Note: This variable was labeled "Annual amount of interest above 6% on high cost debt" in the original registration document

Variable	Mean	Std Dev	N	Missing	Total
High interest-rate debt (not RN)	1,153	2,558	109	4	113
High interest-rate debt (RN, reverse)	-0.06	0.84	109	4	113

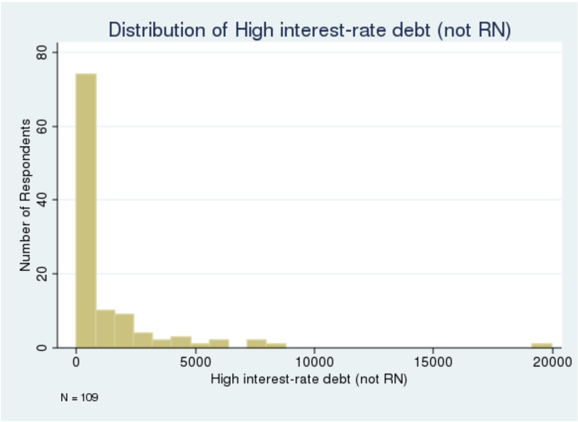

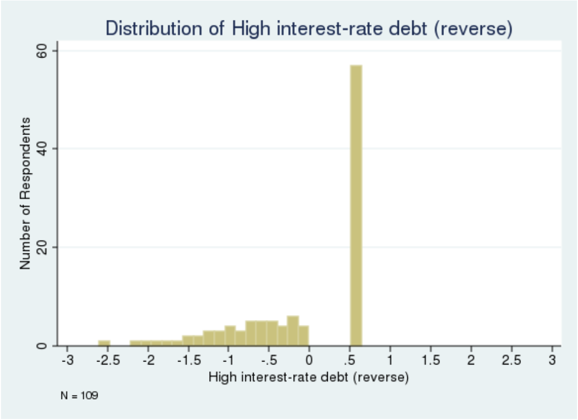

Reported interest paid on debt

**Description**	**Average Debt (>0)**	**Category variables count and percent of non-missing**						**N reporting interest rate / N with non-zero debt**	**Average Interest Rate (assumed for missing)**
		**0-4%**	**5-8%**	**9-12%**	**13-16%**	**17-20%**	**21-24%**		
Home mortgage for your primary residence	$375,359	50	30					80/80	3.69%
Second mortgage or other loans for your primary residence	$108,530	15	8	2				25/27	4.12%
Mortgages or loans for your secondary residence(s)	$263,375	4	4					8/8	4.25%
Mortgages or loans for your rental property	$474,867	6	9					15/15	4.70%
Other real estate loans	$263,250	3	1					4/4	3.13%
Business or farm loans	$231,667	1		1				2/3	6.25%
Vehicle loans	$17,661	17	7			1		25/28	3.92%
Credit cards (if carrying a balance)	$20,700	5	5	3	3	4	1	21/23	10.19%
Installment loans for major purchases (e.g., appliances or furniture)	$3,250	2			1			3/4	6.17%
Educational loans	$32,692	5	8					13/13	4.77%
Other personal loans	$4,700		1					1/1	6.50%
Medical bills	$30,818	5						5/8	2.00%

Distribution of debt and annual amount of interest above 6% across debt categories

**Description**	Sum of Debt ($ and percent of total)		Sum of annual interest over 6% ($ and percent of total)	
Home mortgage for your primary residence	$30,028,700	66%	$52,135	41%
Second mortgage or other loans for your primary residence	$2,930,300	6%	$11,073	9%
Mortgages or loans for your secondary residence(s)	$2,107,000	5%	$6,775	5%
Mortgages or loans for your rental property	$7,123,000	16%	$16,790	13%
Other real estate loans	$1,053,000	2%	$2,840	2%
Business or farm loans	$695,000	2%	$2,363	2%
Vehicle loans	$494,500	1%	$1,675	1%
Credit cards (if carrying a balance)	$476,100	1%	$30,329	24%
Installment loans for major purchases (e.g., appliances or furniture)	$13,000	0%	$87	0%
Educational loans	$425,000	1%	$1,540	1%
Other personal loans	$4,700	0%	$23	0%
Medical bills	$246,540	1%	$0	0%

F. Credit card misuse

- The measure of misuse of credit cards is calculated as the average of the rank normalized responses to the following four questions about the subject's credit card usage. Percentile ranks for the sample are calculated after recoding the variables such that a higher score corresponds to higher misuse of credit cards.
    1. **Amount of carried credit card debt:** *Please indicate the amount you currently owe for any of the following items: Credit cards or other revolving accounts (only if you carry forward a balance; do not include balances that are paid off each month)*
    2. **Annual amount of interest above 6% on high cost credit card debt:** *Please indicate the amount you currently owe for any of the following items: Credit cards or other revolving accounts (only if you carry forward a balance; do not include balances that are paid off each month). Please indicate the interest rate for each of those items. If you are not sure of the exact figures, please provide your best estimate*
    3. **Binary measure of having been denied credit for a credit card in the last year:** *Have you been denied credit for any type of credit card within the last calendar year?*
    4. **Number of late payments in the last year:** *How many times have you made a late payment on any credit card bills in the last calendar year?*
- Note: For respondents who did not fill in a value for carried credit card debt, we assume $0 credit card debt if they reported non-zero debt in other debt categories in the same question and either report owing $0 on their credit cards each month or report paying off 100% of their credit card bill each month

Variable	Mean	Std Dev	N	Missing	Total
Credit card misuse (not RN)	0.06	0.46	113	0	113
Credit card misuse (RN, reverse)	-0.07	0.81	113	0	113

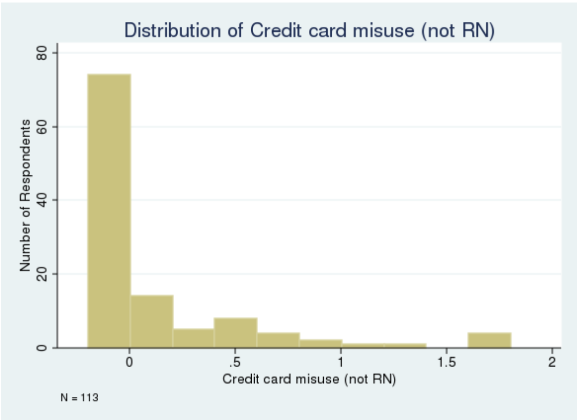

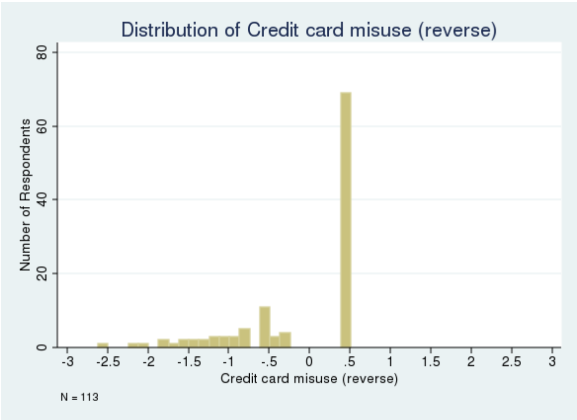

Component 1: Amount of carried credit card debt

Variable	Mean	Std Dev	N	Missing	Total
Amount of credit card debt (not RN)	4,408	13,788	108	5	113
Amount of credit card debt (RN)	0.08	0.70	108	5	113

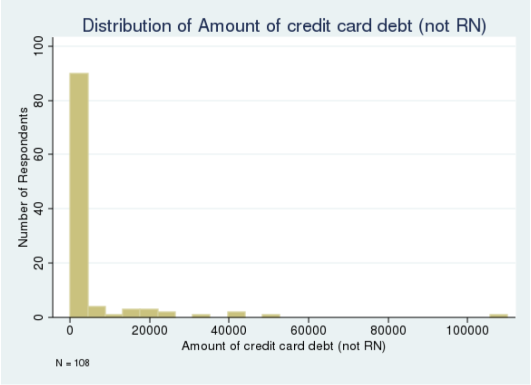

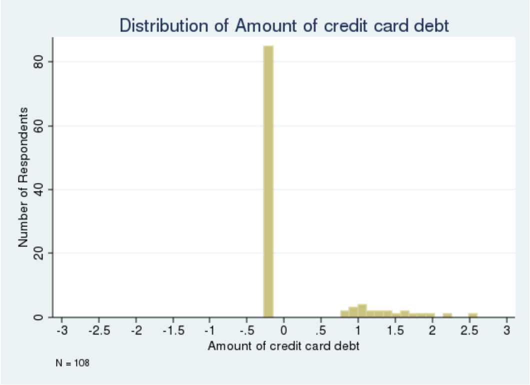

Component 2: Annual amount of interest above 6% on high cost credit card debt

Variable	Mean	Std Dev	N	Missing	Total
Annual interest above 6% on credit card debt (not RN)	281	993	108	5	113
Annual interest above 6% on credit card debt (RN)	0.07	0.66	108	5	113

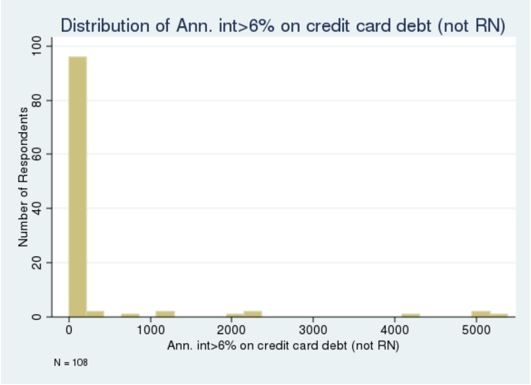

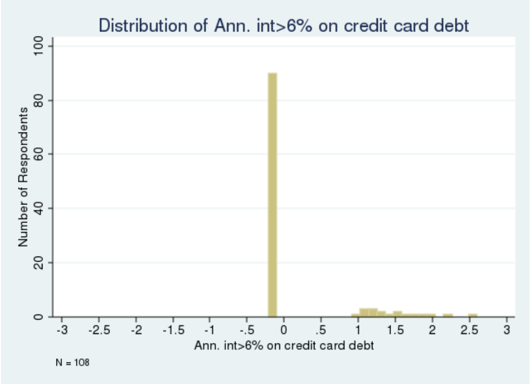

Component 3: Binary measure of having been denied credit for a credit card in the last year

Variable	Mean	Std Dev	N	Missing	Total
Denied credit (not RN)	0.07	0.26	113	0	113
Denied credit (RN)	0.05	0.49	113	0	113

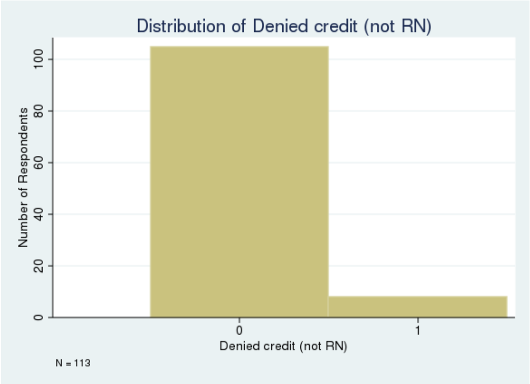

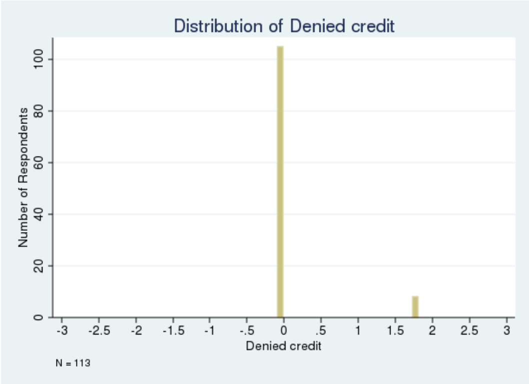

Component 4: Number of late payments in the last year

Variable	Mean	Std Dev	N	Missing	Total
Number of late payments (not RN)	0.29	0.81	112	1	113
Number of late payments (RN)	0.07	0.66	112	1	113

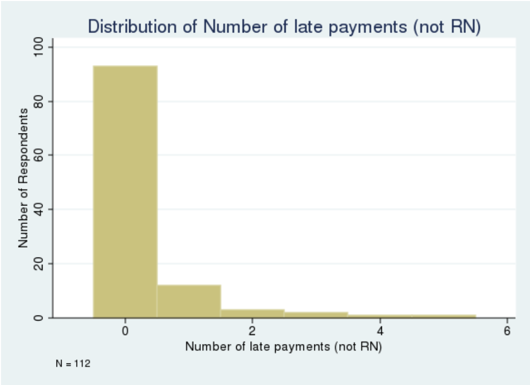

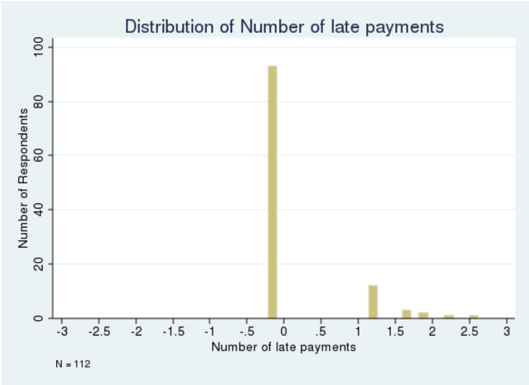

G. Delay choice

- Percent of questions for which the respondent selected the "money later" instead of the "money sooner" option in the tradeoff questions
    - *Which would you prefer? (A) getting $X today (B) getting $Y thirty days from today*
    - *Which would you prefer? (A) getting $X thirty days from today (B) getting $Y sixty days from today*
- One respondent who answered only 2 tradeoff questions is excluded
- 100 of the 113 respondents answered all 40 tradeoff questions. 12 answered 36-39 questions
- 32 subjects were perfectly patient
- Note: delay choice was labeled as "Percent of later choices in money sooner vs. later tradeoffs" in the registration document

Variable	Mean	Std Dev	N	Missing	Total
Delay Choice (not RN)	85%	14%	112	1	113
Delay Choice (RN)	-0.03	0.92	112	1	113

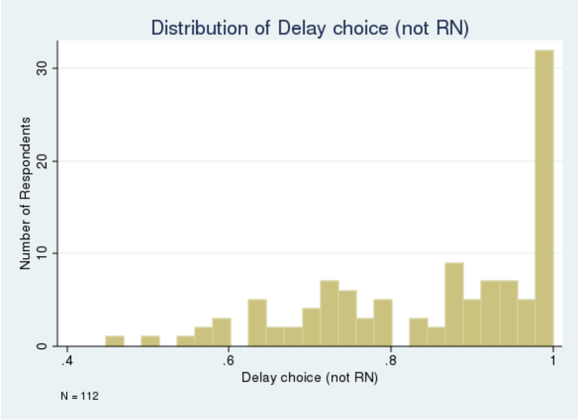

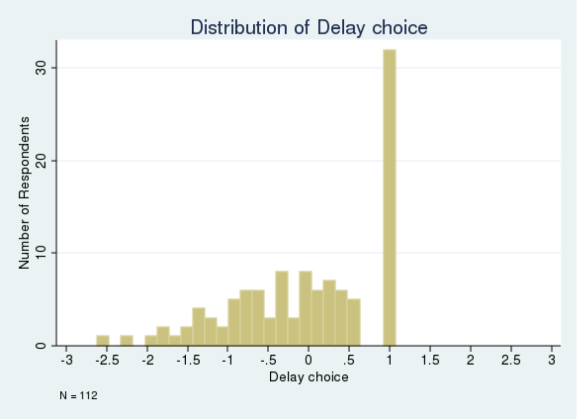

H. Savings rate

- Savings rate over the last few years as a percentage of income
- Note: Reported savings rates of 70% and higher are suspect and excluded from analyses (N=4). These four respondents are believed to be out of the work force or second-earners in their household.
- *On average each year, over the past few (e.g., 3-5) years, what percent of your income have you saved? Please include all forms of saving, including retirement accounts and other savings accounts.*
- 3 subjects did not report savings rate
- Savings rate by sex is not very different (excluding 4 female outliers ≥ 70%)

Variable	Mean	Std Dev	N	Missing	Total
Savings Rate (not RN)	11	9	106	7	113
Savings Rate (RN)	0.01	0.96	106	7	113

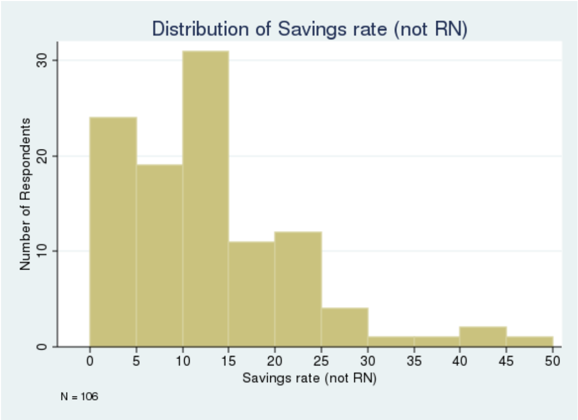

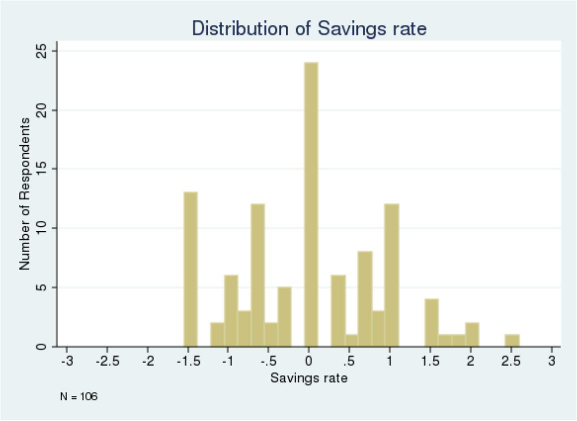

Scatter plot of savings rate (without outlies above 70%) and permanent income. Savings rate (not including outliers ≥ 70%) and rank normalized permanent income are positively correlated (at the 1% level)

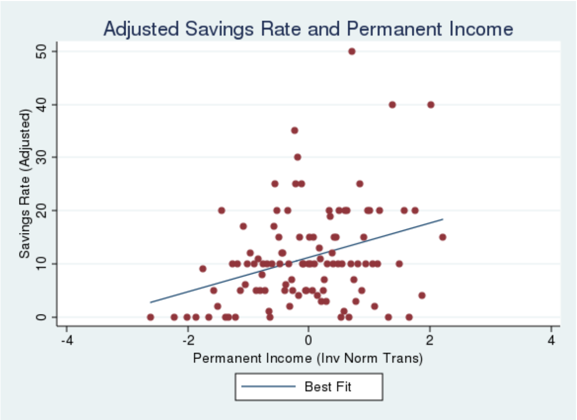

Summary statistics of savings rate by sex

Savings Rate (not RN) by sex	Mean	Std Dev	N	Missing	Total
Female	10.4	10.1	64	7	71
Male	12.0	8.0	42	0	42

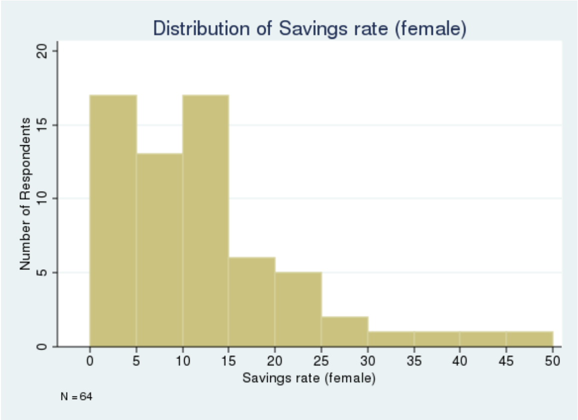

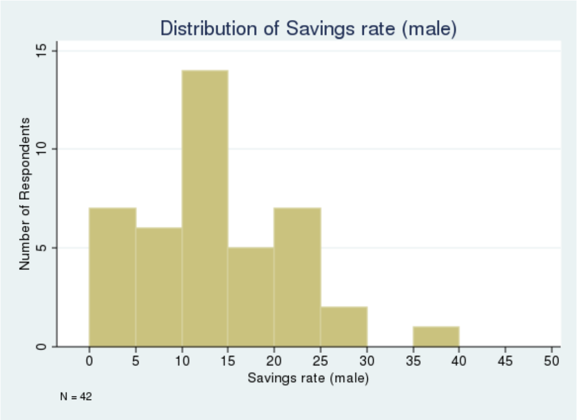

I. Financial health

- Financial health is calculated as the average of the rank normalized responses to the following three questions about the subject's financial situation. Percentile ranks for the sample are calculated after recoding the variables such that a higher score corresponds to a better financial health
    1. *In general, would you say you (and your family living with you) have more money than you need, just enough for your needs, or not enough to meet your needs? (more money = better financial situation)*
    2. *How difficult is it for you (and your family living with you) to pay your monthly bills? Very difficult – Not at all difficult (less difficult = better financial situation)*
    3. *Using a scale from 0 to 10 where 0 means "the worst possible financial situation" and 10 means "the best possible financial situation," how would you rate your financial situation these days? (higher rating = better financial situation)*
- One person did not answer money meets needs. All other subjects had data for all three measures
- Note: financial health is labeled "general measure of financial situation" in the registration document

Variable	Mean	Std Dev	N	Missing	Total
Financial health (not RN)	-0.02	0.73	113	0	113
Financial health (RN)	-0.01	0.98	113	0	113

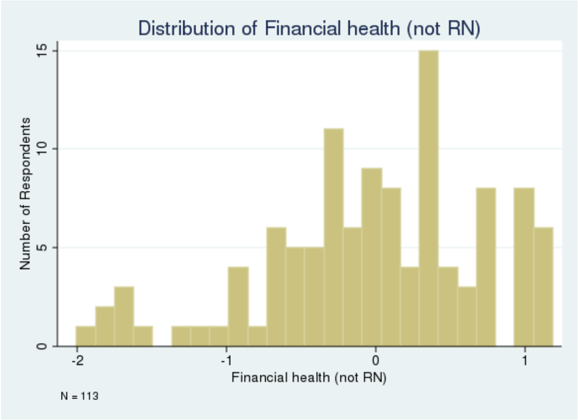

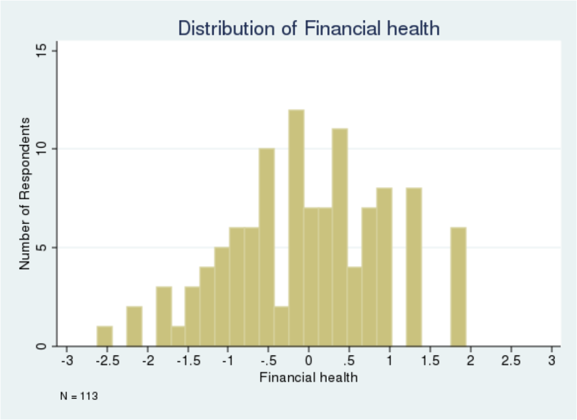

J. Educational attainment (years of education)

- We calculate the number of years of education based on reported degrees received. This is calculated as the sum of 13 years (kindergarten through high school), the base years of education, and any additional years
- All subjects had at least some college, so we assume all have 13 years of education for kindergarten – 12th grade
- "Base years of education" is determined by the highest of the following degrees
  - Associate's Degree: 2 years post-high school
  - Bachelor's Degree: 4 years post-high school
  - Master's Degree: 6 years post-high school
  - PhD: 9 years post high-school
- "Additional years" are added for each of the following degrees
  - Law Degree: + 3 years
  - Medical Degree: + 4 years if highest degree is BA or MA, + 3 years if PhD
  - Second Master's degree: + 2 years
  - Teaching Credential: + 1 year
  - One year post-Master's program: + 1 year
- *For any degree(s) that you hold, please specify below the type of degree you have earned and specify the college, university, or institution that granted it*
- *What is the highest level of education you have attained? High school diploma or equivalent, Some college education, College degree, Some graduate or professional education, Graduate or professional degree*
- Two respondents did not answer the series of questions about degrees and institutions, they both reported "some college education" and were assigned 2 years post-high school
- Note: this variable differs from registration definition. In the registration, we defined years of education post-high school. This variable is the same as the registered variable + 13 years of education.

Variable	Mean	Std Dev	N	Missing	Total
Educational Attainment (years, not RN)	19.1	2.1	113	0	113
Educational Attainment (RN years)	0.02	0.92	113	0	113

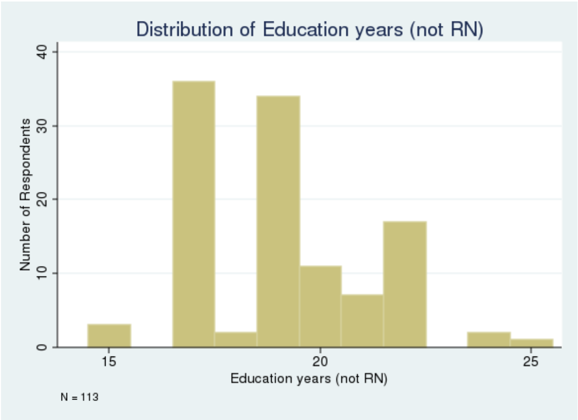

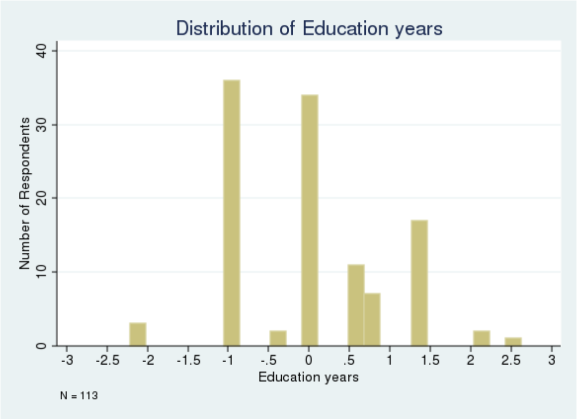

K. Education scale from Ayduk et al (2000)

- Highest educational level: high school = 1, correspondence courses = 2, college = 3, master's = 4, and PhD = 5
- We classify medical degrees as doctorates (5), law degrees as between a Master's and PhD/Medical Degree (4.5), and 2-year degrees with correspondence courses

**Education Level**	**N**	**Percent**	**Percent in Ayduk et al (2000) N=152**
1 = high school	2	2%	11%
2 = correspondence courses/ 2-year degree	1	1%	5%
3 = Bachelor's	38	34%	56%
4 = Master's	39	35%	20%
4.5 = Law Degree	10	9%	--
5 = Medical Degree/ Doctorate	23	20%	7%
NA	--	--	2%
Total	113	100%	

Variable	Mean	Std Dev	N	Missing	Total
Education Scale (not RN)	3.84	0.86	113	0	113
Education Scale (RN)	0.00	0.87	113	0	113

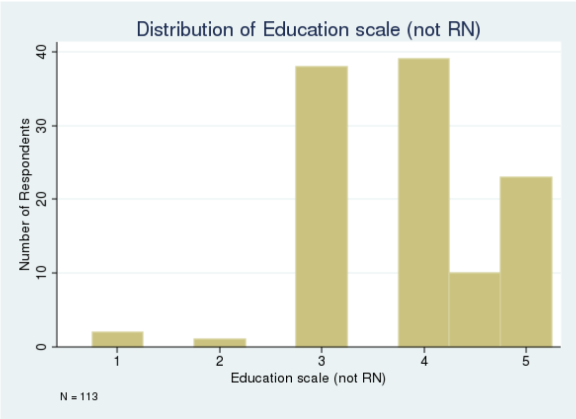

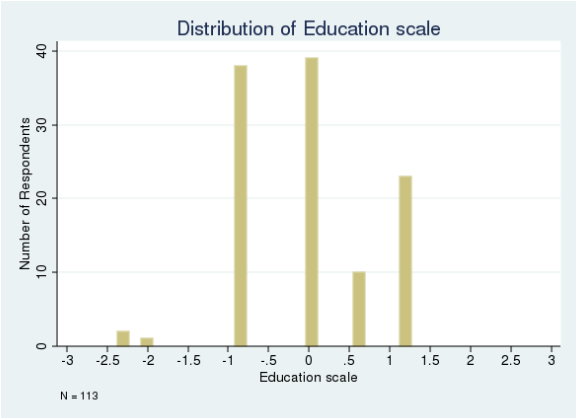

L. Quantitative and verbal SAT scores

- Parent-reported quantitative and verbal SAT scores from the 1984 follow-up (subjects were about 18 years old)
- ETS was able to check the correlation between parent reported and actual SAT scores for 69 of 94 subjects whose parents reported SAT scores. The correlation was 0.94 (Shoda, Mischel, Peake 1990).

Variable	Mean	Std Dev	N	Missing	Total
Quantitative SAT Score (not RN)	626	89	31	82	113
Verbal SAT Score (not RN)	616	74	31	82	113
Quant SAT (RN)	0.00	0.99	31	82	113
Verbal SAT (RN)	0.0	1.0	31	82	113

Quantitative score

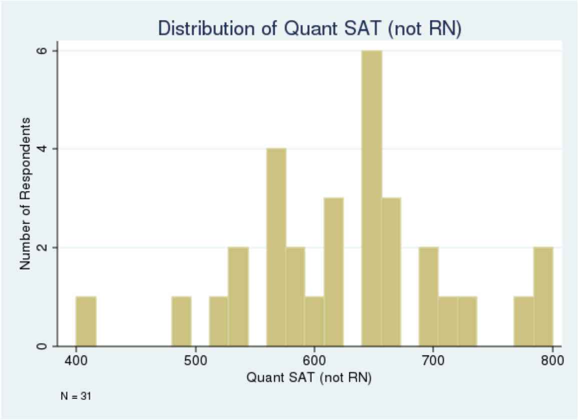

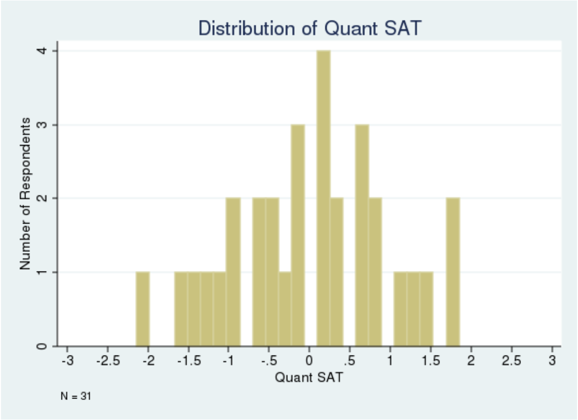

Verbal score

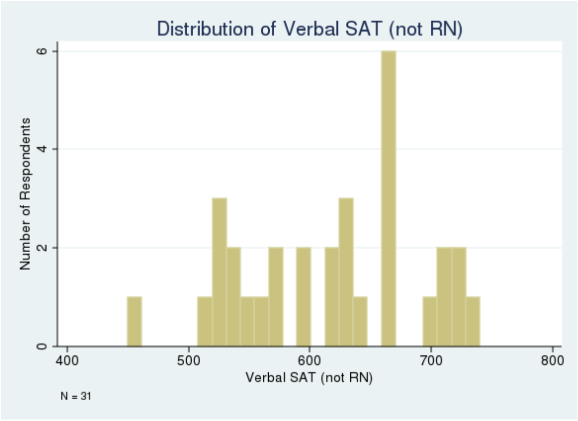

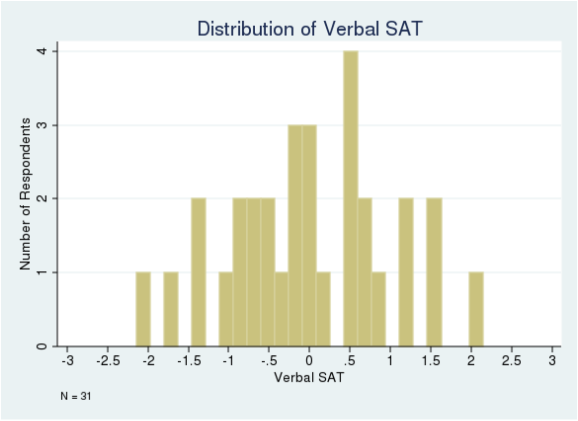

M. Forward-looking behaviors

- Z-scores are calculated by taking inverse normal of the percentile ranks for each question after recoding the variables such that a higher score corresponds to better self-regulation. To create the composite measure, Z-scores are aggregated first by sub-section (e.g., diet, exercise), then by category (e.g., diet+ exercise + BMI), and finally across all five categories. Scores are renormalized (using the percentile transformation) at each step of the aggregation
- Note: each of the 5 subcategories below will be tested separately as secondary analyses
- Diet + Exercise + BMI
  - Diet
    - *In a typical week, how often do you choose your food (the type and/or amount) with health and fitness concerns in mind? (more meals = better regulated)*
    - *In a typical week, how often do you eat more than you think you should eat? (more meals = less regulated)*
    - *Please indicate the degree to which the following statements describe your general tendencies: I tend to eat healthfully. (more like me = better regulated)*
  - Exercise
    - *How many of those hours [that you are active] represent exercise primarily intended to improve or maintain your health or fitness? (more hours = better regulated)*
    - *Please indicate the degree to which the following statements describe your general tendencies: I exercise often. (more like me = better regulated)*
  - BMI calculated as (weight in pounds / (height in inches * height in inches)) x 703
    - *How tall are you? Approximately how much do you currently weigh? (higher BMI = less regulated)*
    - Note: percentile and inverse normal calculations for BMI are done separately for men and women (i.e., z-scores are normally distributed for men and women separately) as women tend to have lower BMI (significantly different in our sample)
- Smoking and Alcohol
  - Smoking behavior: each subject's smoking behavior is ranked such that non-smokers have the lowest rank and smokers are ranked by number of packs per week. (higher rank smoking behavior = less regulated)
    - *Do you smoke (including cigarettes, cigars, pipes, or anything else)? (smoker = less regulated)*
    - *If you smoke cigarettes, about how many packs do you smoke per week? (more packs per week = less regulated)*
    - One smoker did not indicate packs per week and is assumed to smoke the average number of packs per week of other non-smokers. Two smokers indicated 0 packs per week
  - Alcohol consumption for people who drink alcohol. This measure excludes people who reported 0 or missing for all three measures (days per week when drinking, drinks per day, and max drinks in last month) as they may be recovering alcoholics, not drink for religious reasons, etc.
    - Number of drinks in a typical week calculated as days per week when drinking alcohol x typical number of drinks per day when drinking. (more drinks per week = less regulated)
      - *On average, how many days per week do you drink alcohol?*
      - *On a day when you drink, about how many alcohol drinks do you typically have?*
    - *In the past month, what is the maximum number of alcohol drinks you've had in one day (24 hours)? (more drinks = less regulated)*

- Preventative health and dental care
    - Preventative healthcare
        - *When your doctor gives you a prescription (excluding birth control), do you follow it exactly (for example, by taking all of the medication and taking it on the prescribed schedule)? (always adherent = more regulated)*
        - *How often do you visit your doctor for a routine check-up or preventive care? (more often = more regulated)*
    - Preventative dental care
        - *How often do you visit your dentist for a routine check-up or cleaning? (more often = more regulated)*
        - *How often do you floss your teeth? (more often = more regulated)*
- Agreement with statements about procrastination
    - *Please indicate the degree to which the following statements describe your general tendencies: (more like me = less regulated)*
        - *I needlessly delay finishing jobs, even though they are important.*
        - *I postpone starting in on things I don't like to do.*
        - *When I have a deadline, I wait until the last minute.*
        - *I delay making tough decisions.*
- Carefully consider future consequences of current financial decisions
    - *Please indicate the degree to which the following statements describe your general tendencies: I usually carefully consider the future consequences of my current financial decisions. (more like me = more regulated)*
- Two subjects in our sample are technically underweight but we have decided to include their BMI scores as their scores are not far below the 18.5 threshold
- Measure of Alcohol Behavior:
    - If drinks per week is 0, we assume drinks per day drinking is 0 (N=3) and max number of drinks per day in the last month is 0 (N=2) if missing
    - Respondents are excluded from the measure of drinking if they have 0 or missing responses for all three measures: days of drinking, drinks per day, maximum number of drinks in last month

Variable	Mean	Std Dev	N	Missing	Total
Forward-looking behaviors (not RN)	-0.01	0.55	113	0	113
Forward-looking behaviors (RN)	0.00	1.00	113	0	113

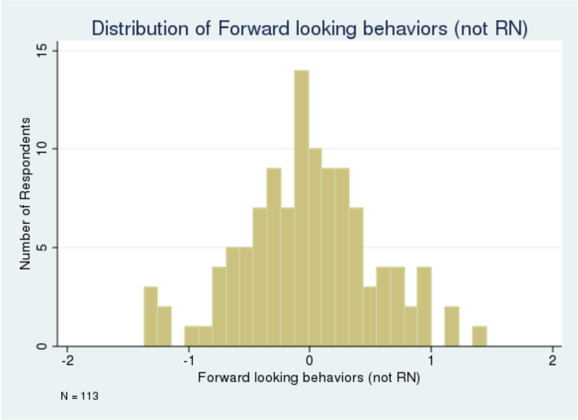

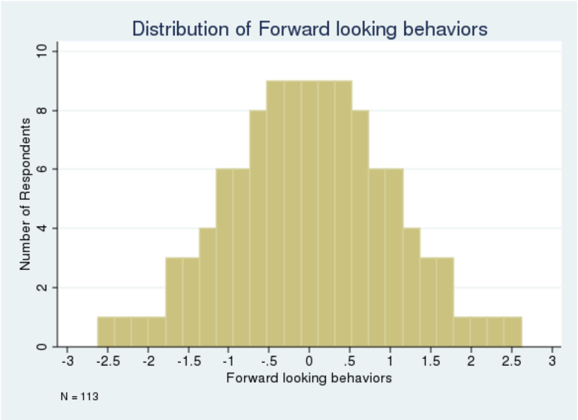

Subgroup 1: Diet, Exercise, and BMI

Variable	Mean	Std Dev	N	Missing	Total
Diet, Exercise, and BMI (not RN)	0.00	0.71	113	0	113
Diet, Exercise, and BMI (RN)	0.00	1.00	113	0	113

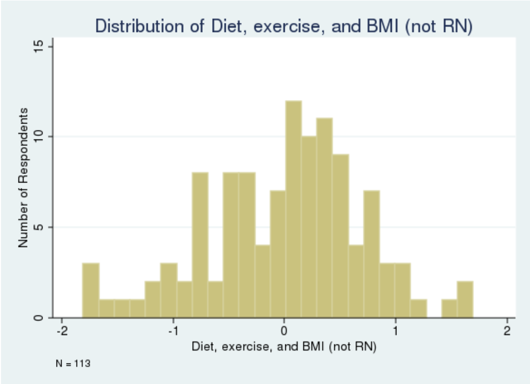

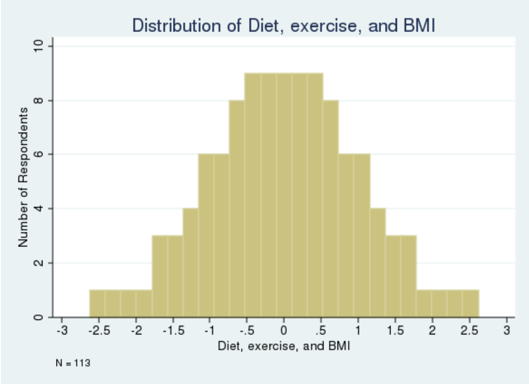

Subgroup 2: Smoking and Drinking Behavior

Variable	Mean	Std Dev	N	Missing	Total
Smoking and drinking behavior (not RN)	-0.03	0.60	113	0	113
Smoking and drinking behavior (RN)	0.00	0.99	113	0	113

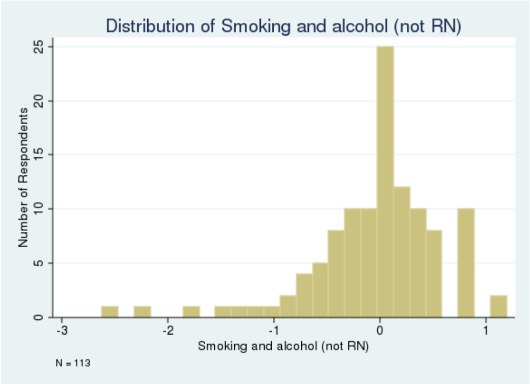

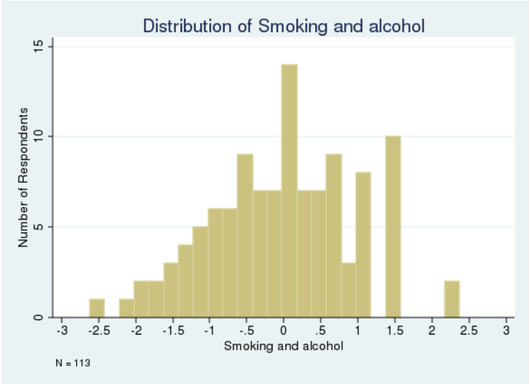

Subgroup 3: Preventative health and dental care

Variable	Mean	Std Dev	N	Missing	Total
Preventative care (not RN)	-0.02	0.77	113	0	113
Preventative care (RN)	0.00	1.00	113	0	113

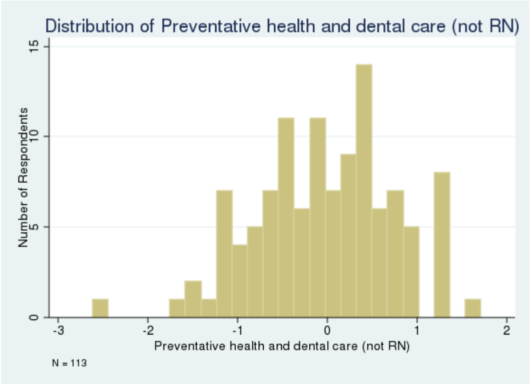

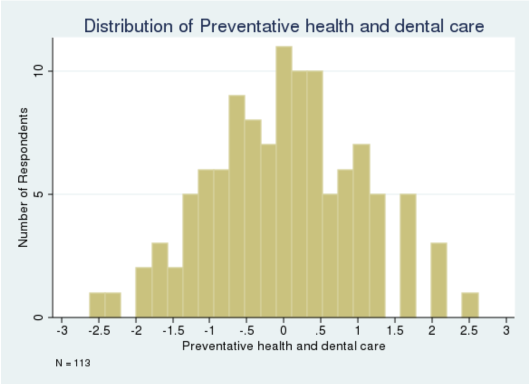

Subgroup 4: Agreement with statements about procrastination

Variable	Mean	Std Dev	N	Missing	Total
Procrastination (not RN)	-0.01	0.69	113	0	113
Procrastination (RN)	-0.01	0.98	113	0	113

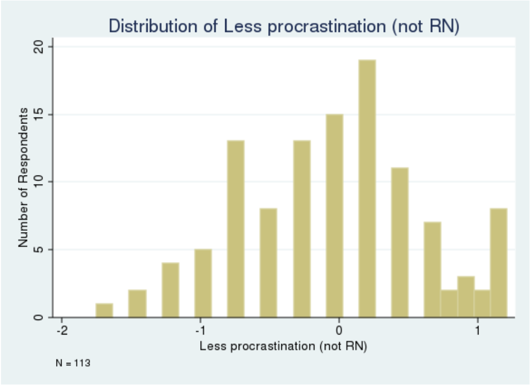

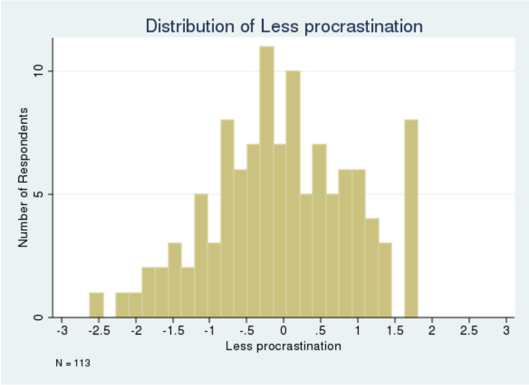

Subgroup 5: Carefully consider future consequences of current financial decisions

Variable	Mean	Std Dev	N	Missing	Total
Consider Consequences (not RN, reverse)	1.6	1	113	0	113
Consider Consequences (RN, reverse)	-0.05	0.80	113	0	113

- Note: the coding of consider finance is reversed ("very much like me to carefully future consequences of my current financial decisions" = 1, "not at all like me" = 4)
- Rank normalized variable is switched so "very much like me" has the highest score

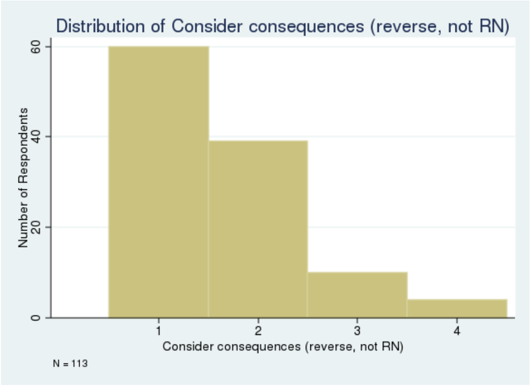

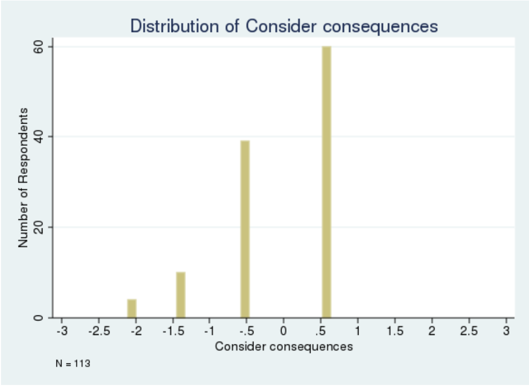

N. Social status

- *Think of this ladder as representing where people stand in the United States. At the top of the ladder are the people who are the best off - - those who have the most money, the most education and the most respected jobs. At the bottom are the people who are the worst off - - who have the least money, least education, and the least respected jobs or no job. The higher up you are on this ladder, the closer you are to the people at the very top; the lower you are, the closer you are to the people at the very bottom. Where would you place yourself on this ladder? Please select the number that corresponds to the rung on the ladder where you think you stand at this time in your life, relative to other people in the United States.*
- Higher scores correspond to higher placement on the ladder of people in the US and rank normalized

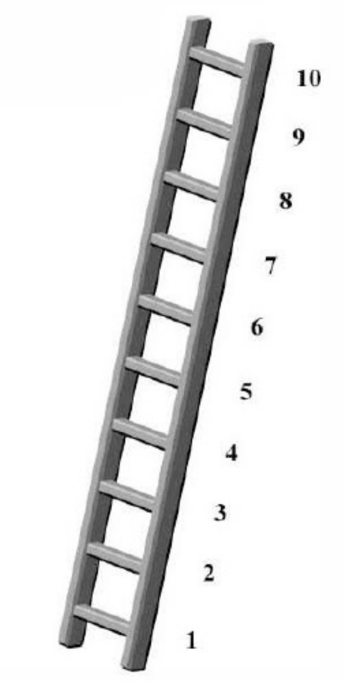

Variable	Mean	Std Dev	N	Missing	Total
Social Status (not RN)	7.6	1	113	0	113
Social Status (RN)	-0.01	0.93	113	0	113

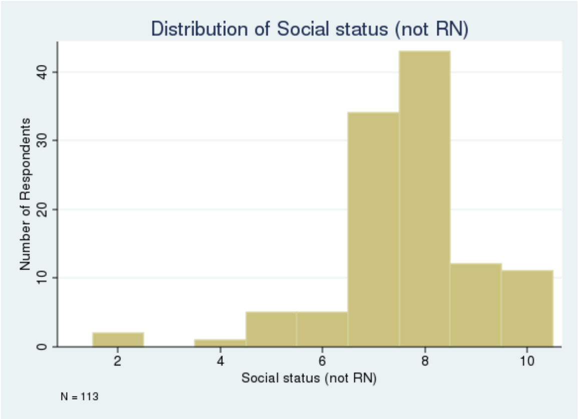

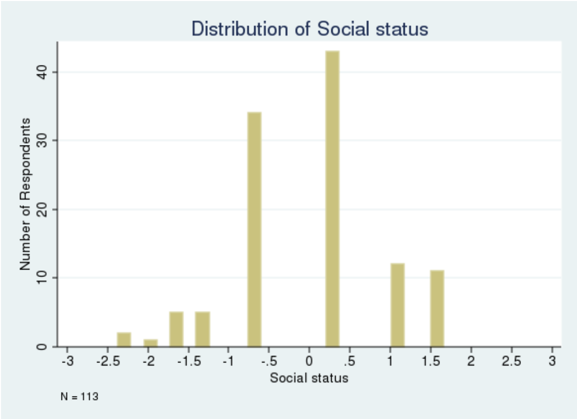

O. Current emotional state and satisfaction with present relationship

- This is a secondary outcome as we consider it a noisy measure of emotional well-being and happiness in relationships that focuses on current emotional states and relationships rather than stable emotional and personality traits
  - The MOS SF-36 is designed to assess patient outcomes after a procedure or treatment and focuses on emotions in the last 4 weeks (Ware and Sherbourn 1992)
  - Rather than being able to measure happiness across all past relationships, the satisfaction with present relationship measure is dependent on the individual's current relationship and their perception of "the average couple"
- After creating the aggregate variables for each subsection listed below, Z-scores are calculated by taking the inverse normal of the percentile ranks for each category. To create the composite measure, Z-scores are averaged across all five categories and renormalized at the end
- <u>Role limitations due to emotional problems in the last 4 weeks</u>: Scored based on the Medical Outcomes Study 36-item short-form (MOS SF-36) where "Yes" = 0 and "No" = 100. The three scores are averaged to a composite score and then rank normalized
  - *During the past 4 weeks, have you had any of the following problems with your work or other regular daily activities as a result of any emotional issues (such as feeling depressed or anxious)?*
    - *Cut down the amount of time you spent on work or other activities*
    - *Accomplished less than you would like*
    - *Were limited in the kind of work or other activities*
    - *Had difficulty performing the work or other activities (for example, it took extra effort)*
    - *Didn't do work or other activities as carefully as usual*
- <u>Health interfering with social activities in the last 4 weeks</u>: Scored based on the MOS SF-36 as described below. The two scores are averaged to a composite score and then rank normalized
  - *During the past 4 weeks, to what extent has your physical health or emotional problems interfered with your normal social activities with family, friends, neighbors, or groups? (Not at all = 100, Slightly=75, Moderately=50, Quite a bit=25, Extremely=0)*
  - *During the past 4 weeks, how much of the time has your physical health or emotional problems interfered with your social activities with family, friends, neighbors, or groups? (All of the time=0, Most of the time=25, Some of the time=50, A little of the time=75, None of the time=100)*
- <u>Emotions felt in the last 4 weeks</u>: Scored based on the MOS SF-36 in increments of 20 points from 0 to 100 in the directions described below (e.g., more often = higher score). The scores are averaged to a composite score and then rank normalized.
  - *These questions are about how you feel and how things have been with you during the past 4 weeks. For each question, please give the answer that comes closest to the way you have been feeling. How much of the time during the past 4 weeks... (Responses: All of the time, Most of the time, A good bit of the time, Some of the time, A little of the time, None of the time)*
    - *Have you been a very nervous person? (more often = lower score)*
    - *Have you felt so down in the dumps that nothing could cheer you up? (more often = lower score)*
    - *Have you felt calm and peaceful? (more often = higher score)*
    - *Have you felt downhearted and blue? (more often = lower score)*
    - *Have you been a happy person? (more often = higher score)*
- <u>Energy/fatigue in the last 4 weeks:</u> Scored based on the MOS SF-36 in increments of 20 points from 0 to 100 in the directions described below (e.g., more often = higher score). The scores are averaged to a composite score and then rank normalized

- *These questions are about how you feel and how things have been with you during the past 4 weeks. For each question, please give the answer that comes closest to the way you have been feeling. How much of the time during the past 4 weeks... (All of the time = lowest, None of the time = highest)*
    - *Did you feel full of pep? (more often = higher score)*
    - *Did you have a lot of energy? (more often = higher score)*
    - *Did you feel worn out? (more often = lower score)*
    - *Did you feel tired? (more often = lower score)*

- <u>Satisfaction with present relationship</u>: Z-scores are calculated by taking inverse normal of the percentile ranks for each of the following three questions after recoding the variables such that a higher score corresponds to happier in relationship. Scores are averaged and then re-normalized (using the percentile transformation) before including in the aggregate measure.
    - *All things considered, compared to the average couple, how happy are you in your present relationship?*
    - *Do you ever wish that you had not married, committed to, or moved in with your partner?*
    - Binary variable equal to 1 if selected "marry or commit to the same person"
        - *If you had your life to live over again, which of these would you most likely do? Marry or commit to the same person, Marry or commit to a different person, Not marry or commit to anyone at all*
    - Note: Individuals who did not answer the relationship questions (e.g., people who are divorced or not in a relationship) are missing this component of the index

Variable	Mean	Std Dev	N	Missing	Total
Current emotional state and satisfaction with present relationship (RN)	0.00	1.00	113	0	113
Components:					
1. Role limitations due to emotional problems in the last 4 weeks	86.02	25.90	113	0	113
2. Health interfering with social activities in the last 4 weeks	88.61	18.57	113	0	113
3. Emotions felt in the last 4 weeks	76.88	13.42	113	0	113
4. Energy/fatigue in the last 4 weeks	62.57	18.09	113	0	113
5. Satisfaction with present relationship (RN)	-0.02	0.94	97	16	113

Current emotional state and satisfaction with present relationship (RN)

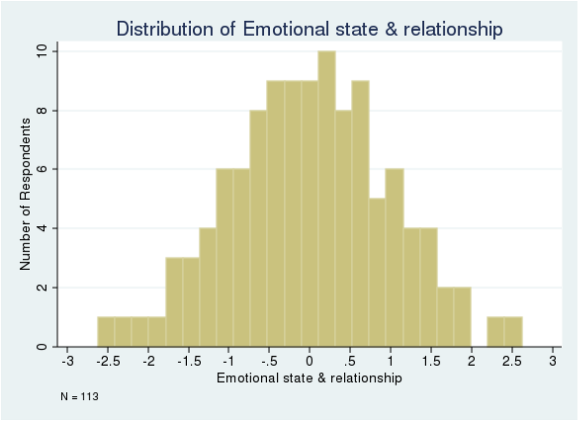

Component 1: Role limitations due to emotional problems in the last 4 weeks (not RN)

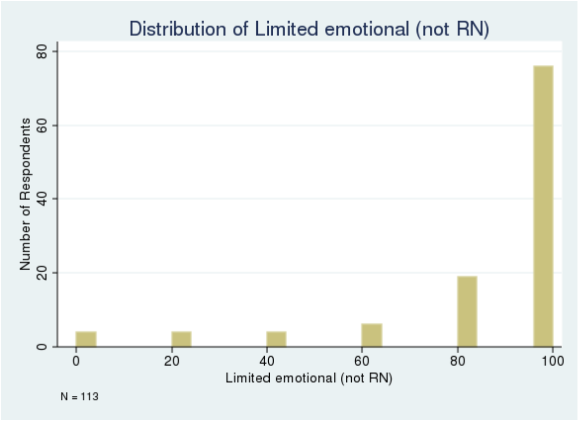

Component 2: Health interfering with social activities in the last 4 weeks (not RN)

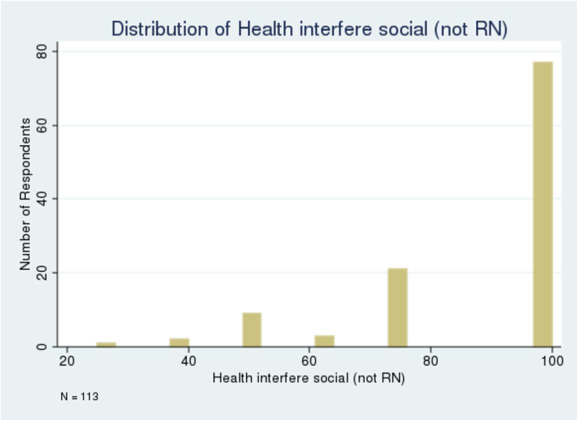

Component 3: Emotions felt in the last 4 weeks (not RN)

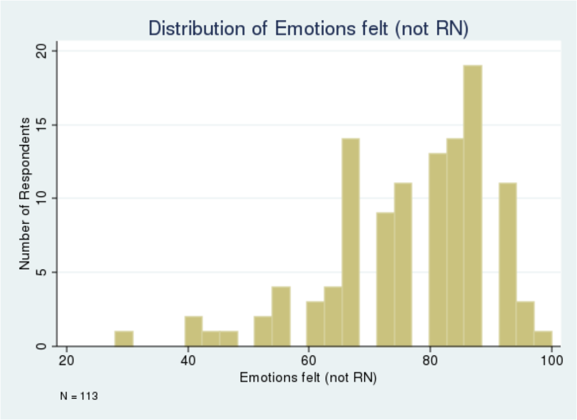

Component 4: Energy/fatigue in the last 4 weeks (not RN)

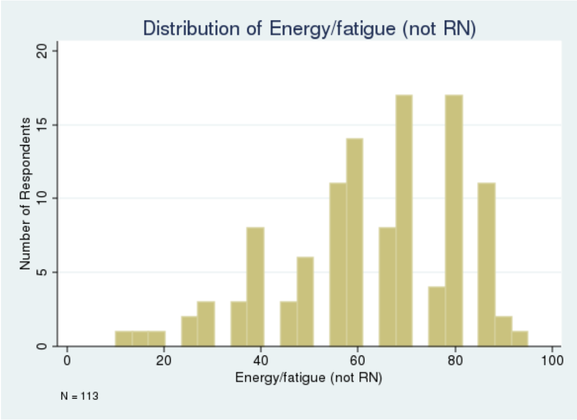

Component 5: Satisfaction with present relationship (RN)

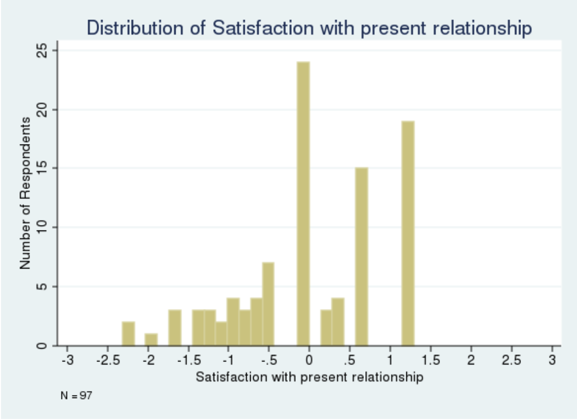

P. Age at the time of the economics survey

- *What is your current age?*

Variable	Mean	Std Dev	N	Missing	Total
Age (not RN)	46.0	2.05	113	0	113

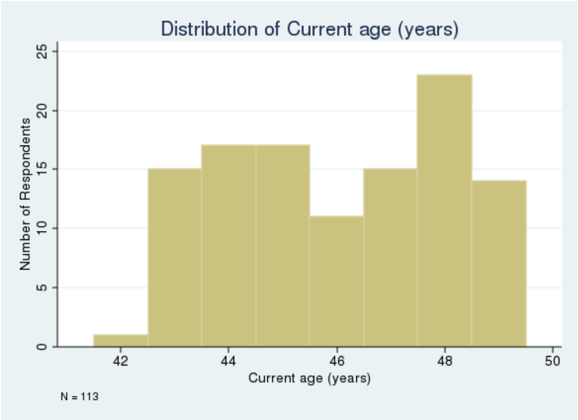

Q. Values of underlying outcome variables 0.5 SD above and below the mean of the transformed variable

These are constructed by:

1. Calculate the mean and SD of the transformed variable. As expected, this is 0 and 1 for most variables. This differs for variables with a pile –up of mass at particular points (e.g., high interest-rate debt has a lot of mass at $0, percent of later choices has a lot of mass at 100%, social status is a discrete variable).

2. Find the observations that are closest to mean ± ½ SD of the transformed variable.

**Primary Outcome**	**Mean**	**0.5 SD below Mean**	**0.5 SD above Mean**
Net worth	938,000	536,000	1,482,000
Perm income	77,626	63,703	116,771
Wealth income ratio	10.1	5.9	13.2
High interest-rate debt	2	0	805
Credit card misuse	*no underlying variable*		
Delay choice	88%	78%	95%
Savings rate	10	6	13
Financial health	*no underlying variable*		
Educational attainment	19	18	20
Forward looking behavior	*no underlying variable*		
Social status	8	7	8

III. Recruitment of subjects for follow-up assessments in 1984, 1993, 2003, and 2013

A. 1984 follow-up (description from Shoda et al. 1990)

- Preschool children's delay of gratification behavior was assessed during a period of approximately 6 years (1967-1973) in a series of experiments conducted at the Bing School at Stanford University (e.g, Mischel & Ebbesen, 1970; Mischel et al., 1972). In those studies, a total of 653 children (316 boys, 337 girls) participated in at least one experiment, 550 of which participated in a standard self-imposed delay situation.
- "About l0 years later (1981-1982), a short questionnaire concerning the coping and cognitive competence of the children and the California Child Q-set (CCQ) were mailed to the 125 parents whose addresses could be located, yielding 95 respondents (see Mischel et al, 1988). To expand the sample of respondents, a second follow-up based on a more extensive address search was conducted in 1984. In the second mailing, all parents were sent a new expanded questionnaire about coping and competence (the Adolescent Coping Questionnaire; ACQ) and a biographical information sheet on which they indicated their children's SAT scores. The CCQs were also sent to those who either did not respond to the previous mailing or did not receive the mailing because their addresses were not available at that time. Materials were mailed to parents of 506 subjects. This yielded responses from parents of 90 subjects whose data were not available in the first-wave follow-up, as well as additional data from the parents who had responded in the first mailing. As a result, the sample for which CCQ was available increased from 67 to 165, and we obtained parental ratings on the new 14-item ACQ for 134 children, as well as reports of 94 children's SAT scores. Together with the first-wave mailing, a sample of 185 children was now available on whom there was at least one follow-up measure."
- "Of the 653 original subjects, 103 were not tested in a standard self-imposed delay situation and therefore were not used. Of the remaining 550, no address was known for 114, and follow-up materials were re turned by the post office as undeliverable for an additional 32. Thus, 404 subjects probably received our follow-up material (barring unreported mail loss), of which the present sample of 185 represents a return rate of 46%. The 185 subjects in the present sample delayed an average of 62 s longer than the 219 who did not respond to our mailing (512.8 s vs. 450.7 s), but the difference was not significant at the .05 level, $t(402) = 1.7$, $p=.09$."

B. 1993 follow-up (description from Ayduk et al. 2000)

- "In the adult follow-up assessment conducted between 1993 and 1995, questionnaires were sent to the parents of 444 participants for whom we had identified any possible current address. The mailing included a questionnaire for parents to complete, plus a separate envelope that contained questionnaires that parents were asked to address and send to their children. A total of 71 questionnaires were returned as "not deliverable," and 10 questionnaires were returned but not completed because the children were deceased. With these adjustments, the total potential number of responses was 363 participants. In all, 187 parents and 152 children returned questionnaires. In 56 cases, questionnaires were received from the parents but not their children. In 21 cases, questionnaires were received from the children but not their parents. Thus, questionnaires were available from both a parent and his or her child for a total of 131 participants (53 men and 78 women). Of these 131 parent responses, 60% were completed solely by the mother, 14% were completed solely by the father, and 26% were completed jointly by both parents. There were no cases in which each parent completed a separate questionnaire. The participants who responded to the adult follow-up (N=152) did not differ significantly in age from the larger pool of children when the initial preschool delay measure was obtained ($t < 1$), nor did they differ in the actual length of the self-imposed delay period (i.e., voluntary waiting time; $t < 1$). For the 131 responding participants for whom we also had parent-reported data, the delay times were not significantly different from those for whom we had only parent-reported data (N=56; $t < 1$) or

from those for whom we had only self-reported data (N=21; r < 1). The mean age of the participants in the 1993 follow-up was 27 years, 1 month (SD = 19 months)."

C. 2003 follow-up

- Ayduk et al. 2008: "As part of this ongoing longitudinal project, a new follow-up wave was initiated in 2003 and those whose current address was available (N = 306) were sent an initial invitation letter and a demographics form. Those who responded affirmatively to this invitation (N=205) were then sent several questionnaire packages approximately 6 months to a year later that included the PAI-BOR, an adult version of the RSQ, and the ACQ. Participants who completed all 3 of these questionnaires form the focus of the current study (N = 104, 66 women, 38 men). Delay of gratification data from age 4 was available for 80 of these participants. At the time of the initial mailing, the mean age in this sample was 38.88 years (SD = 2.01, range: 34 – 42.66). Of the sample, 76.70% was married or engaged and another 11.65% was in unmarried relationships, and 65.69% had at least one child. All but two had college or graduate degrees (36.54% Bachelors, 34.62% Masters, 26.92% Ph.D., J.D. or M.D.). Majority of the participants were of Caucasian origin."
- Casey et al. 2011: "Of the 562 participants for whom we had preschool delay scores at age 4 y, 155 completed the 1993 follow-up and 135 completed the 2003 follow-up."
- Schlam et al. 2013: "As part of a recent follow-up (approved by the Columbia University Institutional Review Board), we sent participants 2 mailings asking them to report their height and weight. The first follow-up, which also included demographic questions, was mailed in December 2002/January 2003 to all participants for whom we had a valid address (N= 306). The second follow-up was mailed approximately 17 months later in May 2004. (We sent 2 mailings in an effort to obtain data from as many subjects in the original sample as possible.) All participants provided written informed consent for the follow-up. The current study includes the 164 participants (57% women) who completed the delay of gratification task at age 4 and reported their height and weight approximately 30 years later at the first follow-up (N=146; 58% women) and/or the second follow-up (N=97; 60% women). Seventy-nine participants (62% women) responded to both follow-ups."

D. 2013 economics follow-up ("survey sample")

- As of Spring, 2012, we had email addresses in our database for 180 Bing Ss. We emailed invitation letters to all 180 Ss. Of these 180, 61 emails bounced back as undeliverable. We then sent invitations via postal mail to the last known mailing address for these 61 Ss. Of these 61 Ss, 27 Ss responded with current contact information, and 11 of the letters were returned as undeliverable. We emailed invitation letters to the 27 Ss who responded with current contact information. Summary: Of the 180, we currently have verified email addresses for 146 (i.e., 146 have either sent us an email or responded to our email by participating in the survey); for 34 of the 180 participants, we know the email addresses we have for them are not valid (e.g., email sent to them have bounced back).
- We sent invitation letters out via postal mail to 135 Ss for whom we did not have email addresses. These Ss most recently participated in 2003 and 1993. 57 of these letters were returned as undeliverable. 10 Ss responded with current contact information. All 10 were sent invitation letters for the current follow-up. Summary: Of the 135, we now have verified contact info (e.g., email address) for 10; For 57 of the 135 Ss, we know the contact information we have is not valid (e.g., postal mail sent to these addresses have been returned as undeliverable); For 68 of the 135 Ss, we are uncertain as to whether our contact information is valid (postal mail was not returned, but we have not heard back from these Ss).
- Overall summary: we have active and verified (as of 2012) contact information for 156 participants.

IV. Statistical methodology (strategy for addressing multiple testing problems)

A. Primary analyses

- To address the issue of multiple hypothesis testing, we report the following for each primary test:
  1. Coefficient, standard error, and nominal *p*-value
  2. Indication of significance from an analysis controlling the false discovery rate at 0.1. This analysis is done separately for each of the two primary RHS variables - the rank normalized self-regulatory index (RNSRI) and rank normalized delay (RND)
  3. Bayesian "best guess" and SE for each coefficient given the priors listed below
- We also report the results of an enrichment analysis that tests joint significance of our primary hypotheses using a Wald test. We evaluate significance using an empirical p-value calculated from a distribution of Wald statistics created by randomly resampling the key independent variable from the sample (with replacement), repeating all analyses, and re-running the Wald test. We evaluate significance using a 0.05 empirical p-value threshold. We run separate empirical Wald tests for each of the two primary RHS variables (the RNSRI and RND)

B. Secondary analyses

- For analyses with secondary outcomes, we report coefficients, standard errors, nominal *p*-values, and Bayesian "best guess" and SE for each coefficient given the priors listed below
- For secondary tests (i.e., comparison of diagnostic to non-diagnostic conditions) and secondary sets of independent variables, we report coefficients, standard errors, and nominal *p*-values

C. Priors for Bayesian analyses

- We set our priors for Bayesian analysis based on the key independent variable in the primary analyses:
  - Correlations with the rank normalized self-regulatory index (RNSRI): mean of prior distribution = 0.15
  - Correlations with rank normalized delay (RND): mean of prior distribution = 0.05
- We assume a standard deviation of the prior correlation of 0.2 for all analyses

D. Detailed methodology: Bayesian data analysis

Fix any one of our dependent variables of interest y (e.g., permanent income). We estimate a multivariate linear regression model, $y_i = \boldsymbol{x_i}'\boldsymbol{\beta} + \varepsilon_i$, where $i = 1, \dots, N$ indexes individuals, $\varepsilon_i \sim_{\text{i.i.d.}} N(0, \eta^2)$, with η^2 known and $\boldsymbol{x_i}$ fixed. Let $x_{ij} \in \boldsymbol{x_i}$ be the key independent variable (either the RNSRI or RND) and β_j be the corresponding coefficient in vector $\boldsymbol{\beta}$. We have a Gaussian prior on the population coefficient $\beta_j \sim N(\theta, \nu^2)$. Below, after deriving the posterior distribution for β_j, we discuss how we set values of θ and ν^2.

The OLS estimate for the coefficient vector is given by the usual formula $\widehat{\boldsymbol{\beta}} = (\boldsymbol{X}'\boldsymbol{X})^{-1}\boldsymbol{X}'\boldsymbol{y}$, where $\boldsymbol{y}$ and $\boldsymbol{X}$ denote, respectively, the vector of observed values of the dependent variable and the matrix of observed values of the independent variables across the N individuals. Define the matrix $\boldsymbol{\Sigma} \equiv \eta^2(\boldsymbol{X}'\boldsymbol{X})^{-1}$. As is well known, $\widehat{\boldsymbol{\beta}}|\boldsymbol{\beta}, \boldsymbol{\Sigma} \sim N(\boldsymbol{\beta}, \boldsymbol{\Sigma})$. Hence the conditional distribution of the jth element of $\widehat{\boldsymbol{\beta}}$, denoted $\hat{\beta}_j$, depends only on the jth element of $\boldsymbol{\beta}$, denoted β_j, and the jj^{th} entry of $\boldsymbol{\Sigma}$, denoted σ_j^2. Specifically, $\hat{\beta}_j|\beta_j, \sigma_j^2 \sim N(\beta_j, \sigma_j^2)$. It follows that the vector $(\hat{\beta}_j, \sigma_j^2)$ is a sufficient statistic for the posterior distribution of β_j (i.e., the posterior distribution of β_j depends on $(\boldsymbol{y}, \boldsymbol{X})$ only through $(\hat{\beta}_j, \sigma_j^2)$). We can therefore write the posterior distribution of β_j as $f(\beta_j|\hat{\beta}_j, \sigma_j^2)$, where we use $f(\cdot)$ to denote a probability density function.

The prior for β_j is

$$f(\beta_j) = \frac{1}{\sqrt{2\pi\nu^2}} e^{-\frac{1}{2\nu^2}(\beta_j-\theta)^2},$$

and the likelihood of observing $\hat{\beta}_j$, given the true population β_j and the observed $\boldsymbol{X}$, is

$$f(\hat{\beta}_j|\beta_j, \sigma_j^2) = \frac{1}{\sqrt{2\pi\sigma_j^2}} e^{-\frac{1}{2\sigma_j^2}(\hat{\beta}_j-\beta_j)^2}.$$

Standard calculations yield the posterior distribution,

$$\beta_j|\hat{\beta}_j, \sigma_j^2 \sim N\left(\frac{\nu^2}{\sigma_j^2+\nu^2}\hat{\beta}_j + \frac{\sigma_j^2}{\sigma_j^2+\nu^2}\theta, \frac{\sigma_j^2\nu^2}{\sigma_j^2+\nu^2}\right).$$

In the last part of this section, we explain how we set the parameters of the prior distribution, θ and ν^2. Without loss of generality, orient all the dependent variables so that their expected correlation with the self-regulatory independent variable is positive. Regarding θ, the prior on the partial correlation between y_i and x_{ij} (controlling for the other independent variables in $\boldsymbol{x_i}$):

- When the independent variable x_{ij} is the *RNSRI*, we set θ=0.15.
- When the independent variable is *RND*, we set θ=0.05.

For all analyses, we fix the standard deviation of the correlation, ν, at 0.2. See the section below for discussion of these prior distributions and related existing evidence.

Note: In the original pre-registration document (Section X.B. Bayesian data analysis), we describe a procedure for translating a prior on the partial correlation into a prior on the regression coefficient, taking into account the fact that the variables may not be distributed standard normal. Because the key independent variables (RNSRI and RND) and all of the primary and secondary outcome variables are inverse-normal transformed, we do not need to apply this procedure and instead interpret the regression coefficients in our primary and secondary analyses as partial correlations.

Discussion of priors for Bayesian analysis

1. Mean of the correlation between outcome measures and self-regulatory measures

Recall that in our Bayesian data analysis, we set the mean of our prior distribution such that it corresponds to a partial correlation between each outcome variable and the independent variable of…

- …0.15 when the independent variable is the *RNSRI*.
- …0.05 when the independent variable is *RND* in preschool

For all three independent variables, we set the standard deviation of our prior distribution to correspond to a standard deviation of 0.20 for the partial correlation. In this section, we briefly discuss some of our reasoning and some of the related evidence that helped motivate these calibrations for the parameter values.

In the prior work most similar to our own, Moffitt et al. (2011) explore correlations between a multi-year self-control index (a composite of behaviors measured from age 3 to 11) and several wealth outcomes measured at age 32. Controlling for gender, they find correlations with wealth outcomes are in the range of 0.15-0.27. Controlling for childhood socioeconomic status, IQ, and gender, they find correlations with wealth outcomes are in the range of 0.08-0.23. Moffitt et al. use different measures of self-control and a different set of outcome variables so these findings are not fully analogous with our own analyses, but their results provide a helpful point of reference and suggest that our prior mean 0.15 is perhaps slightly conservative.

In a meta-analysis, Richard, Bond, and Stokes-Zoota (2003, p.337) find that the average "person effect"—the estimated relationship between a social behavior and a demographic, personality, or other dispositional variable—across 16,282 estimated effects is 0.19. Compared to this benchmark, 0.15 again seems somewhat conservative.

For correlations between our outcomes and rank normalized delay in preschool, we use as our benchmark prior an even lower correlation of 0.05. We hypothesized that behavior in the delay task has less explanatory power than the self-regulatory index as it is a single measure of self-regulatory ability measured much earlier in life (and further from the outcomes) than the self-regulatory index.

2. Standard deviation of the correlation between outcome measures and self-regulatory measures

Finally, we discuss how we set the standard deviation for our prior. We note that, unlike for the mean where a value closer to zero is clearly more conservative, it is ambiguous whether a larger or smaller standard deviation is more conservative: a larger standard deviation means that the prior distribution has less overall impact on the conclusions relative to the data, but a smaller deviation shrinks large estimated

effects more toward the (conservative) mean of the prior distribution. We think that a standard deviation of 0.20 roughly captures our degree of uncertainty about the strength of the correlation for both independent variables. For example, it means that our approximate 95% confidence interval (mean ± 2 standard deviations) on the correlation between the rank normalized self-regulatory index and an outcome variable is (-0.25, 0.55). By way of comparison, Richard, Bond, and Stokes-Zoota (2003, p.336) find that about 5% of published findings in social psychology report correlation coefficients larger than 0.50.

E. Detailed methodology: False discovery rate control

Let $H_{01}, H_{02}, \dots, H_{0N}$ denote *N* the null hypotheses we want to test. Suppose that of these hypotheses, the null hypothesis is actually true in N_0 cases, and the non-null is true in $N_1 (= N - N_0)$ cases. We want to adopt a decision rule, a rule that uses the p-values to declare R of the hypotheses as significant and N - R as non-significant (e.g., the Bonferroni decision rule would be: reject H_{0i} if $p_i < 0.05/N$). Some of the hypotheses declared significant are actually null; let *a* denote the number of these. Some of the hypotheses declared significant are actually non-null; let *b* denote the number of these.

One approach to avoiding false positives is to adopt a decision rule that bounds the probability that *at least one* true null hypothesis is declared significant. This probability, Pr $(a > 0)$, is called the family-wise error rate (FWER). The most well-known method of controlling the FWER is the Bonferroni decision rule. In our context, controlling the FWER would not be very informative because, given our relatively small sample size, our power to reject the family-wise null (that at least one of the null hypotheses is true) is very small.

The approach we focus on instead is to adopt a decision rule that bounds the (expected) proportion of true null hypotheses that are declared significant. This proportion, $\frac{a}{R}$, is called the false discovery rate (FDR). Just as a rejection threshold must be chosen in classical testing for a single null hypothesis (conventionally 0.05), controlling the FDR requires specifying a FDR threshold q (conventionally 0.10).

The most widely known method of controlling the FDR is Benjamini and Hochberg's (1995) algorithm. It proceeds as follows. Compute a p-value for each $i = 1, \dots, N$. Re-index the p-values in increasing order: $p_{(1)} \le p_{(2)} \le \cdots \le p_{(N)}$. Find the largest index i_{max} such that

$$p_{(i)} \le \frac{i}{N} q.$$

The decision rule, denoted $BH(q)$, is: declare all hypotheses $i \le i_{max}$ as significant and all hypotheses $i > i_{max}$ as non-significant.

Benjamini and Hochberg (1995) proved that if $p_1, \dots, p_N$ are mutually independent, then under $BH(q)$,

$$E\left(\frac{a}{R}\right) = \left(\frac{N_0}{N}\right) q \le q.$$ [34]

In words, the expected proportion of false discoveries is at most q. In our application, because the life outcomes we study are correlated, the assumption of independent p-values across hypotheses almost surely does not hold. Below, we return to this point and explain why the decision rule $BH(q)$ typically performs well even when the independence assumption does not hold.

To help clarify that point, and to aid in interpreting the decision rule $BH(q)$, note that $BH(q)$ also has an empirical Bayes interpretation (Efron, Tibshirani, Storey, and Tusher, 2001). Let $F(p)$ denote the true (unknown) cdf of the p-values, and $F_0(p)$ denote the theoretical cdf of the p-values under the null hypothesis. Using Bayes' Rule,

$$\Pr\left(\text{hypothesis } (i) \text{ is actually null} \mid \text{its } p-\text{value } \leq p_{(i)}\right) = \frac{F_0\left(p_{(i)}\right)\Pr(\text{hypothesis } (i) \text{ is actually null})}{F(p_{(i)})}$$

Note that because each $p_{(i)} \sim U(0,1)$, we know that $F_0\left(p_{(i)}\right) = p_{(i)}$. We can estimate $F(p)$ by its empirical counterpart, $\bar{F}(p) \equiv \frac{\#\{p_i \leq p\}}{N}$, and note that (due to the assumption of independence across hypotheses) $\bar{F}\left(p_{(i)}\right) \equiv \frac{i}{N}$. We can replace $\Pr(\text{hypothesis } (i) \text{ is actually null})$ by its average value, $\frac{N_0}{N}$. Substituting,

$$\widehat{\Pr}\left(\text{hypothesis } (i) \text{ is actually null} \mid \text{its } p-\text{value } \leq p_{(i)}\right) = \left(\frac{N_0}{N}\right)\frac{p_{(i)}}{\left(\frac{i}{N}\right)}$$

If the threshold rule holds with equality at i_{max}, $p(i_{max}) = \frac{i_{max}}{N}q$, then

$$\widehat{\Pr}\left(\text{hypothesis } (i_{max}) \text{ is actually null} \mid \text{its } p-\text{value } \leq p_{(i_{max})}\right) = \left(\frac{N_0}{N}\right)q \leq q.$$

All hypotheses $i \leq i_{max}$ are weakly less likely than i_{max} to be null, given their p-values. Therefore, for all hypotheses with $p_{(i)} \leq p_{(i_{max})}$, $\widehat{\Pr}\left(\text{hypothesis } (i) \text{ is actually null} \mid p_{(i)} \leq p\right)$ is at most q. In words, under decision rule $BH(q)$, the probability that a hypothesis declared to be significant is actually null is at most q.

The empirical Bayes justification of the decision rule $BH(q)$ makes it easier to see why the independence assumption is not crucial. In the empirical Bayes argument, the independence assumption is used only in making the claim that $\bar{F}(p)$ is an unbiased estimate of $F(p)$. Efron (2010, Lemma 2.1 and p.55) shows that, in the absence of the independence assumption, $\bar{F}(p)$ is biased upward—and hence the decision rule $BH(q)$ is even more conservative. Moreover, Efron argues that in practice, the bias is small. The main cost of greater correlation in the p-values is that the estimated false discovery rate has greater sampling variation.

V. Supplementary tables

A. Primary analyses

- We test, in separate regressions, the relationship between each of 11 primary outcomes and 2 primary measures of self-regulatory ability – rank normalized self-regulatory index (RNSRI) and rank normalized delay (RND)– for a total of 22 primary analyses
  - Primary outcomes (all rank normalized): net worth, permanent income, wealth-income ratio, high interest-rate debt, delay choice, credit card misuse, savings rate, financial health, educational attainment, index of forward-looking behaviors, and social status
  - Primary right hand side variables: RNSRI and RND
- We control for sex in all analyses (note that we do not control for age at the time of the delay task because, in the wait time analyses where age would be relevant, the variable we use—deviation from expected wait time—already takes age into account)
- Each regression is of the following form

$$y = \alpha + \beta_1 \times x + \beta_2 \times male$$

  where y is one of the primary outcomes, x is one of the primary right hand side variables (RNSRI or RND), $male$ is a dummy for being male, and α is a constant. β_1 is the coefficient of interest
- In analyses with savings rate as the primary outcome measure, we also include permanent income as an additional covariate
- Consistent with our pre-registered analysis plan, we report the following for the primary analyses:
  - Coefficient, standard error, and nominal p-value
  - Indication of significance from an analysis controlling the false discovery rate at 0.1. This analysis is done separately for each of the two primary RHS variables (RNSRI and RND).
  - Bayesian "best guess" and SE for each coefficient given the priors listed below
  - Results of an enrichment analysis that tests joint significance of our primary hypotheses using a Wald test. We evaluate significance using an empirical p-value calculated from a distribution of Wald statistics created by randomly resampling the key independent variable from the sample (with replacement), repeating all analyses, and re-running the Wald test. We evaluate significance using a 0.05 empirical p-value threshold. We ran separate empirical Wald tests for each of the two primary RHS variables (RNSRI and RND)

1. Measure of self-regulation is RNSRI

RNSRI is index that includes RND and RNCCQ at age 17, 27, and 37 all equally weighted.

OLS Regressions: coefficients, standard errors, and nominal p-value

	(1) Net worth	(2) Perm income	(3) Wealth income ratio	(4) High interest-rate debt (reverse)	(5) Credit card misuse (reverse)	(6) Delay choice	(7) Savings rate	(8) Financial health	(9) Education years	(10) Forward looking behaviors	(11) Social status
RNSRI	0.306***	0.323***	0.086	0.092	0.175**	0.155*	-0.006	0.240**	0.244***	0.350***	0.142
	(0.095)	(0.092)	(0.099)	(0.084)	(0.077)	(0.088)	(0.094)	(0.092)	(0.086)	(0.086)	(0.088)
Male	0.002	-0.066	0.063	0.085	0.040	-0.064	0.231	0.151	-0.045	-0.592***	0.316*
	(0.195)	(0.190)	(0.203)	(0.171)	(0.158)	(0.183)	(0.183)	(0.189)	(0.178)	(0.179)	(0.182)
Perm income							0.336***				
							(0.099)				
Constant	0.017	0.038	-0.027	-0.095	-0.090	0.003	-0.060	-0.052	0.016	0.233**	-0.106
	(0.120)	(0.115)	(0.125)	(0.105)	(0.095)	(0.111)	(0.114)	(0.114)	(0.108)	(0.108)	(0.110)
N	106	109	106	106	110	109	103	110	110	110	110
R^2	0.091	0.106	0.008	0.014	0.047	0.030	0.129	0.065	0.070	0.207	0.049

Standard errors in parentheses
* $p < 0.1$, ** $p < 0.05$, *** $p < 0.01$

Average of the coefficients: 0.192
Bootstrapped standard error (ex-post): 0.039
[Ex-post analysis: p-value of test comparing average to 0: 9.513e-07, p-value comparing average to 0.15: 0.288]
Coefficients with the predicted sign: 10/11

Measure of self-regulation is RNSRI: index that includes RND and RNCCQ at age 17, 27, and 37 all equally weighted.

Indication of significance controlling the false discovery rate at 0.1

**Outcome**	**Coefficient**	***p*-value**	***p*-value Threshold**	**FDR Significant?**
Net worth	0.306	0.002	0.027	1
Perm income	0.323	0.001	0.018	1
Wealth income ratio	0.086	0.388	0.091	0
High interest-rate debt (rev)	0.092	0.273	0.082	0
Credit card misuse (rev)	0.175	0.024	0.055	1
Delay choice	0.155	0.082	0.064	0
Savings rate	-0.006	0.950	0.100	0
Financial health	0.240	0.010	0.045	1
Education years	0.244	0.006	0.036	1
Forward looking behaviors	0.350	0.000	0.009	1
Social status	0.142	0.111	0.073	0

Measure of self-regulation is RNSRI: index that includes RND and RNCCQ at age 17, 27, and 37 all equally weighted.

Bayesian posterior distribution of the coefficients

Prior on the correlation: mean 0.15 and standard deviation of 0.2

Outcome	Prior Mean	Square Root of Prior Variance	Observed Mean	Square Root of Observed Variance	Posterior Mean	Square Root of Posterior Variance
Net worth	0.150	0.200	0.306	0.095	0.277	0.086
Perm income	0.150	0.200	0.323	0.092	0.293	0.084
Wealth income ratio	0.150	0.200	0.086	0.099	0.099	0.089
High interest-rate debt (rev)	0.150	0.200	0.092	0.084	0.101	0.077
Credit card misuse (rev)	0.150	0.200	0.175	0.077	0.172	0.071
Delay choice	0.150	0.200	0.155	0.088	0.154	0.081
Savings rate	0.150	0.200	-0.006	0.094	0.022	0.085
Financial health	0.150	0.200	0.240	0.092	0.224	0.083
Education years	0.150	0.200	0.244	0.086	0.229	0.079
Forward looking behaviors	0.150	0.200	0.350	0.086	0.318	0.079
Social status	0.150	0.200	0.142	0.088	0.143	0.081
**Average**	0.150		0.192		0.185	

Measure of self-regulation is RNSRI: index that includes RND and RNCCQ at age 17, 27, and 37 all equally weighted.

Empirical Wald test

We evaluate joint significance using an empirical p-value calculated as the percentile of the p-value assuming asymptotic theory in a distribution of p-values that assume asymptotic theory. Each p-value assuming asymptotic theory in the simulated distribution represents a p-value from the Wald test conducted on a dataset with the key independent variable for each individual replaced with a random draw (with replacement) from the set of observed values of the key independent variable and the primary dependent variables maintained as their actual values for each individual.

The histogram is the distribution of *p*-values assuming asymptotic theory from all simulation runs (each with a random resampling of the RNSRI variable). The red line indicates the *p*-value assuming asymptotic theory from the Wald test with the actual RNSRI variable. The empirical *p*-value is the percentile of the *p*-value assuming asymptotic theory with the actual RNSRI variable (red line) in the simulated distribution of *p*-values assuming asymptotic theory (histogram).

Note: theoretical distribution of *p*-values is skewed due to small N and high correlation between outcome variables

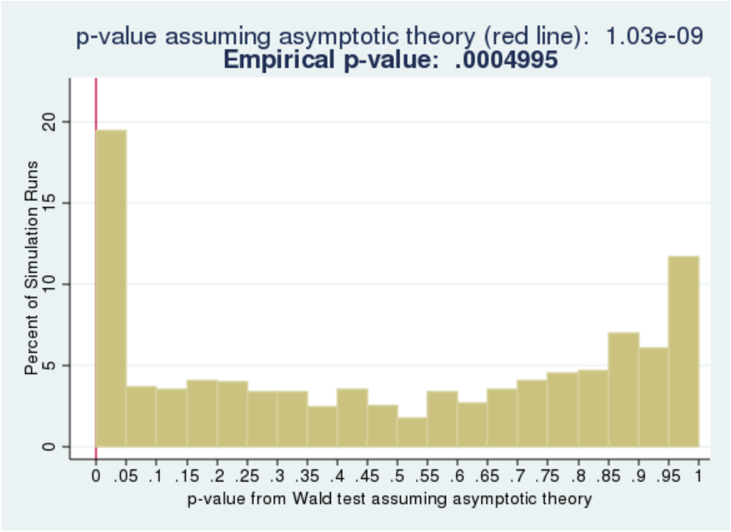

2. Measure of self-regulation is RND

RND is expected deviation from predicted wait time in uncensored log seconds (measured in a delay of gratification study at Bing preschool).

OLS Regressions: coefficients, standard errors, and nominal *p*-value

	(1) Net worth	(2) Perm income	(3) Wealth income ratio	(4) High interest-rate debt (reverse)	(5) Credit card misuse (reverse)	(6) Delay choice	(7) Savings rate	(8) Financial health	(9) Education years	(10) Forward looking behaviors	(11) Social status
RND	0.092	-0.077	0.153	-0.007	0.044	0.086	-0.062	-0.073	0.133	0.092	-0.134
	(0.098)	(0.097)	(0.097)	(0.083)	(0.077)	(0.088)	(0.089)	(0.094)	(0.087)	(0.091)	(0.087)
Male	-0.022	-0.043	0.013	0.102	0.062	-0.126	0.276	0.194	-0.055	-0.622***	0.307*
	(0.199)	(0.197)	(0.198)	(0.168)	(0.159)	(0.181)	(0.180)	(0.193)	(0.180)	(0.187)	(0.180)
Perm income							0.341***				
							(0.091)				
Cons	0.012	0.015	0.000	-0.103	-0.094	0.013	-0.079	-0.077	0.037	0.231**	-0.121
	(0.123)	(0.120)	(0.122)	(0.104)	(0.096)	(0.110)	(0.113)	(0.117)	(0.109)	(0.114)	(0.109)
N	109	112	109	109	113	112	106	113	113	113	113
R^2	0.008	0.007	0.024	0.003	0.005	0.012	0.142	0.013	0.021	0.095	0.042

Standard errors in parentheses
* $p < 0.1$, ** $p < 0.05$, *** $p < 0.01$

Average of the coefficients: 0.022
Bootstrapped standard error (ex-post): 0.047
[Ex-post analysis: p-value of test comparing average to 0: 0.631, p-value comparing average to 0.05: 0.556]
Coefficients with the predicted sign: 6/11

Measure of self-regulation is RND: Expected deviation from predicted wait time in uncensored log seconds (measured in a delay of gratification study at Bing preschool)

Indication of significance controlling the false discovery rate at 0.1

**Outcome**	**Coefficient**	***p*-value**	***p*-value Threshold**	**FDR Significant?**
Net worth	0.092	0.350	0.055	0
Perm income	-0.077	0.427	0.064	0
Wealth income ratio	0.153	0.117	0.009	0
High interest-rate debt (rev)	-0.007	0.934	0.100	0
Credit card misuse (rev)	0.044	0.570	0.091	0
Delay choice	0.086	0.331	0.045	0
Savings rate	-0.062	0.489	0.082	0
Financial health	-0.073	0.438	0.073	0
Education years	0.133	0.131	0.027	0
Forward looking behaviors	0.092	0.316	0.036	0
Social status	-0.134	0.129	0.018	0

Measure of self-regulation is RND: Expected deviation from predicted wait time in uncensored log seconds (measured in a delay of gratification study at Bing preschool)

Bayesian posterior distribution of the coefficients

Prior on the correlation: mean 0.05 and standard deviation of 0.2

Outcome	Prior Mean	Square Root of Prior Variance	Observed Mean	Square Root of Observed Variance	Posterior Mean	Square Root of Posterior Variance
Net worth	0.050	0.200	0.092	0.098	0.084	0.088
Perm income	0.050	0.200	-0.077	0.097	-0.053	0.087
Wealth income ratio	0.050	0.200	0.153	0.097	0.134	0.087
High interest-rate debt (rev)	0.050	0.200	-0.007	0.083	0.001	0.076
Credit card misuse (rev)	0.050	0.200	0.044	0.077	0.045	0.072
Delay choice	0.050	0.200	0.086	0.088	0.080	0.080
Savings rate	0.050	0.200	-0.062	0.089	-0.043	0.081
Financial health	0.050	0.200	-0.073	0.094	-0.051	0.085
Education years	0.050	0.200	0.133	0.087	0.120	0.080
Forward looking behaviors	0.050	0.200	0.092	0.091	0.085	0.083
Social status	0.050	0.200	-0.134	0.087	-0.104	0.080
**Average**	0.050		0.022		0.027	

Measure of self-regulation is RND: Expected deviation from predicted wait time in uncensored log seconds (measured in a delay of gratification study at Bing preschool)

Empirical Wald test

We evaluate joint significance using an empirical *p*-value calculated as the percentile of the *p*-value assuming asymptotic theory in a distribution of *p*-values that assume asymptotic theory. Each *p*-value assuming asymptotic theory in the simulated distribution represents a *p*-value from the Wald test conducted on a dataset with the key independent variable for each individual replaced with a random draw (with replacement) from the set of observed values of the key independent variable and the primary dependent variables maintained as their actual values for each individual.

The histogram is the distribution of *p*-values assuming asymptotic theory from all simulation runs (each with a random resampling of the RND variable). The red line indicates the *p*-value assuming asymptotic theory from the Wald test with the actual RND variable. The empirical *p*-value is the percentile of the *p*-value assuming asymptotic theory with the actual RND variable (red line) in the simulated distribution of *p*-values assuming asymptotic theory (histogram).

Note: the distribution of *p*-values assuming asymptotic theory is skewed due to small *N* and high correlation between outcome variables

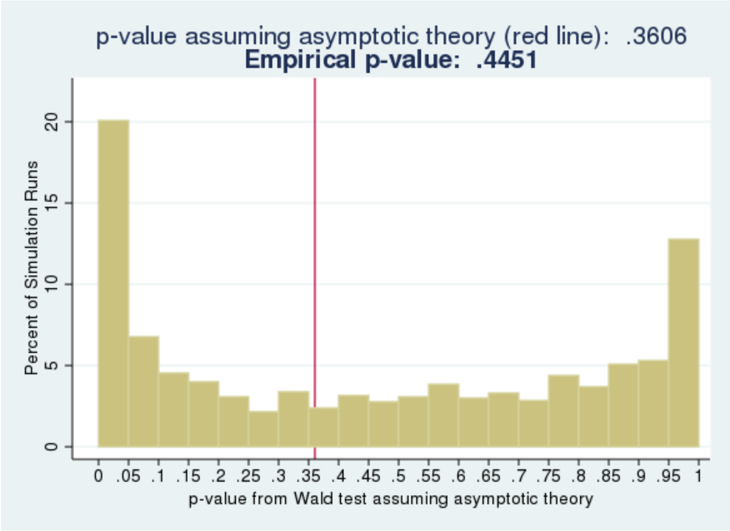

B. Secondary analyses

- We pre-registered three sets of secondary analyses:
    - Secondary outcome variables
    - Secondary sets of independent variables
    - Analysis of Diagnostic vs. Non-Diagnostic Conditions

1. Secondary outcome variables

- We test 10 secondary outcome variables with both primary right hand side variables:
    - The five components of the index of forward-looking behaviors: diet, exercise, and BMI; smoking and alcohol use; preventative health and dental care; agreement with statements about procrastination; and carefully consider future consequences of current financial decisions
    - Savings rate as an outcome variable without controlling for permanent income
    - Quantitative and Verbal SAT scores
    - Education scale as described in Ayduk et al. (2000)
    - Current emotional state and satisfaction with present relationship
- For these analyses, we report coefficients, standard errors, nominal *p*-values, and Bayesian “best guess” and SE for each coefficient given the priors listed below

Measure of self-regulation is RNSRI

RNSRI is index that includes RND and RNCCQ at age 17, 27, and 37 all equally weighted.

OLS Regressions: coefficients, standard errors, and nominal *p*-value

	(1)<br>Diet, exercise, and BMI	(2)<br>Smoking and alcohol	(3)<br>Preventative health and dental care	(4)<br>Less procrast-ination	(5)<br>Consider consequences	(6)<br>Savings rate	(7)<br>Verbal SAT	(8)<br>Quant SAT	(9)<br>Education scale	(10)<br>Emotional state & relationship
RNSRI	0.391***	0.058	0.103	0.228**	0.169**	0.110	0.265*	0.198	0.262***	0.086
	(0.088)	(0.091)	(0.090)	(0.093)	(0.076)	(0.092)	(0.150)	(0.143)	(0.081)	(0.096)
Male	-0.313*	-0.466**	-0.665***	-0.170	0.032	0.239	-0.252	0.796**	0.002	0.236
	(0.182)	(0.189)	(0.186)	(0.192)	(0.158)	(0.192)	(0.371)	(0.354)	(0.167)	(0.198)
Constant	0.115	0.189*	0.254**	0.060	-0.060	-0.078	0.069	-0.269	-0.019	-0.080
	(0.110)	(0.114)	(0.112)	(0.116)	(0.095)	(0.120)	(0.211)	(0.201)	(0.101)	(0.119)
N	110	110	110	110	110	103	31	31	110	110
R^2	0.176	0.058	0.118	0.061	0.044	0.028	0.118	0.190	0.089	0.020

Standard errors in parentheses
* $p < 0.1$, ** $p < 0.05$, *** $p < 0.01$

Average of the coefficients: 0.187
Coefficients with the predicted sign: 10/10

Measure of self-regulation is RNSRI

RNSRI is an index that includes RND and RNCCQ at age 17, 27, and 37 all equally weighted.

Bayesian posterior distribution of the coefficients

Prior on the correlation: mean 0.15 and standard deviation of 0.2

Outcome	Prior Mean	Square Root of Prior Variance	Observed Mean	Square Root of Observed Variance	Posterior Mean	Square Root of Posterior Variance
Diet, exercise, and BMI	0.150	0.200	0.391	0.088	0.351	0.081
Smoking and alcohol	0.150	0.200	0.058	0.091	0.074	0.083
Preventative health and dental care	0.150	0.200	0.103	0.090	0.111	0.082
Less procrastination	0.150	0.200	0.228	0.093	0.214	0.084
Consider consequences	0.150	0.200	0.169	0.076	0.167	0.071
Savings rate	0.150	0.200	0.110	0.092	0.117	0.084
Verbal SAT	0.150	0.200	0.265	0.150	0.223	0.120
Quant SAT	0.150	0.200	0.198	0.143	0.182	0.116
Education scale	0.150	0.200	0.262	0.081	0.246	0.075
Emotional state & relationship	0.150	0.200	0.086	0.096	0.098	0.086

Measure of self-regulation is RND

RND is expected deviation from predicted wait time in uncensored log seconds (measured in a delay of gratification study at Bing preschool).

OLS Regressions: coefficients, standard errors, and nominal *p*-value

	(1) Diet, exercise, and BMI	(2) Smoking and alcohol	(3) Preventative health and dental care	(4) Less procrast-ination	(5) Consider consequences	(6) Savings rate	(7) Verbal SAT	(8) Quant SAT	(9) Education scale	(10) Emotional state & relationship
RND	0.064	0.089	-0.010	0.026	0.071	-0.072	-0.021	0.061	0.126	-0.030
	(0.095)	(0.091)	(0.091)	(0.094)	(0.076)	(0.094)	(0.179)	(0.167)	(0.083)	(0.095)
Male	-0.301	-0.561***	-0.629***	-0.195	0.022	0.288	-0.299	0.761**	-0.009	0.238
	(0.195)	(0.187)	(0.187)	(0.193)	(0.157)	(0.191)	(0.390)	(0.364)	(0.171)	(0.196)
Constant	0.112	0.204*	0.233**	0.066	-0.059	-0.102	0.096	-0.250	0.004	-0.088
	(0.118)	(0.114)	(0.114)	(0.117)	(0.096)	(0.120)	(0.222)	(0.207)	(0.104)	(0.119)
N	113	113	113	113	113	106	31	31	113	113
R^2	0.024	0.080	0.095	0.010	0.008	0.025	0.021	0.139	0.021	0.014

Standard errors in parentheses
* $p < 0.1$, ** $p < 0.05$, *** $p < 0.01$

Average of the coefficients: 0.030
Coefficients with the predicted sign: 6/10

Measure of self-regulation is RND: Expected deviation from predicted wait time in uncensored log seconds (measured in a delay of gratification study at Bing preschool)

Bayesian posterior distribution of the coefficients

Prior on the correlation: mean 0.05 and standard deviation of 0.2

Outcome	Prior Mean	Square Root of Prior Variance	Observed Mean	Square Root of Observed Variance	Posterior Mean	Square Root of Posterior Variance
Diet, exercise, and BMI	0.050	0.200	0.064	0.095	0.062	0.086
Smoking and alcohol	0.050	0.200	0.089	0.091	0.083	0.083
Preventative health and dental care	0.050	0.200	-0.010	0.091	0.001	0.083
Less procrastination	0.050	0.200	0.026	0.094	0.031	0.085
Consider consequences	0.050	0.200	0.071	0.076	0.068	0.071
Savings rate	0.050	0.200	-0.072	0.094	-0.050	0.085
Verbal SAT	0.050	0.200	-0.021	0.179	0.010	0.133
Quant SAT	0.050	0.200	0.061	0.167	0.056	0.128
Education scale	0.050	0.200	0.126	0.083	0.115	0.077
Emotional state & relationship	0.050	0.200	-0.030	0.095	-0.015	0.086

2. Secondary sets of independent variables

- We test the following sets of secondary RHS variables in combination with all primary and secondary outcome variables:
    - RND (deviation from predicted wait time in log seconds) and aggregate RNCCQ index
        - $y = \alpha + \beta_1 \times RND + \beta_2 \times RNCCQ + \beta_3 \times male$
    - RND (deviation from predicted wait time in log seconds) and separate RNCCQ indices at each age (17, 27, and 37)
        - $y = \alpha + \beta_1 \times RND + \beta_2 \times (Age\ 17\ RNCCQ\ index) + \beta_3 \times (Age\ 27\ RNCCQ\ index) + \beta_4 \times (Age\ 37\ RNCCQ\ index) + \beta_5 \times male$
    - RND (deviation from predicted wait time in log seconds) and each of the six subscales of the RNCCQ index (averaged across years)
        - $y = \alpha + \beta_1 \times RND + \beta_2 \times attention\ subscale + \beta_3 \times coping\ subscale + \beta_4 \times goal\ pursuit\ subscale + \beta_5 \times concern\ for\ others\ subscale + \beta_6 \times delay\ subscale + \beta_7 \times general\ cognitive\ ability\ subscale\ \ \beta_8 \times male$

- For the analyses with secondary sets of independent variables, we report coefficients, standard errors, and nominal *p*-values

Independent variables: RND and RNCCQ (aggregated over ages 17, 27, and 37)

OLS regressions for primary outcome variables: coefficients, standard errors, and nominal *p*-value

Note: RND and RNCCQ index are the two sub-components of the RNSRI (where RND has ¼ weight and RNCCQ index has ¾ weight).

	(1) Net worth	(2) Perm income	(3) Wealth income ratio	(4) High interest-rate debt (reverse)	(5) Credit card misuse (reverse)	(6) Delay choice	(7) Savings rate	(8) Financial health	(9) Education years	(10) Forward looking behaviors	(11) Social status
RND	0.040	-0.145	0.143	-0.014	0.022	0.067	-0.070	-0.112	0.085	0.047	-0.162^{*}
	(0.097)	(0.090)	(0.101)	(0.085)	(0.078)	(0.090)	(0.092)	(0.091)	(0.087)	(0.088)	(0.087)
RNCCQ	0.297^{***}	0.429^{***}	0.001	0.083	0.150^{*}	0.118	0.008	0.307^{***}	0.237^{***}	0.337^{***}	0.229^{**}
	(0.097)	(0.090)	(0.100)	(0.085)	(0.079)	(0.090)	(0.099)	(0.091)	(0.088)	(0.088)	(0.088)
Male	0.021	0.004	0.027	0.097	0.046	-0.071	0.244	0.197	-0.044	-0.578^{***}	0.368^{**}
	(0.198)	(0.184)	(0.205)	(0.174)	(0.161)	(0.185)	(0.185)	(0.187)	(0.179)	(0.181)	(0.179)
Perm income							0.328^{***}				
							(0.103)				
Constant	0.006	0.007	-0.010	-0.101	-0.092	0.006	-0.065	-0.070	0.016	0.228^{**}	-0.126
	(0.121)	(0.111)	(0.125)	(0.106)	(0.097)	(0.112)	(0.115)	(0.112)	(0.108)	(0.109)	(0.108)
N	106	109	106	106	110	109	103	110	110	110	110
R^2	0.092	0.184	0.021	0.012	0.037	0.026	0.134	0.104	0.083	0.205	0.101

Standard errors in parentheses
* $p < 0.1$, ** $p < 0.05$, *** $p < 0.01$

	Average of coefficients (bootstrapped SE)	Coefficients with the predicted sign
RND	-0.009 (0.05)	6/11
RNCCQ	0.200 (0.04)	11/11

Independent variables: RND and RNCCQ (aggregated over ages 17, 27, and 37)

OLS regressions for secondary outcome variables: coefficients, standard errors, and nominal *p*-value

	(1) Diet, exercise, and BMI	(2) Smoking and alcohol	(3) Preventative health and dental care	(4) Less procrast-ination	(5) Consider consequences	(6) Savings rate	(7) Verbal SAT	(8) Quant SAT	(9) Education scale	(10) Emotional state & relationship
RND	0.009	0.082	-0.010	-0.004	0.041	-0.101	-0.076	0.025	0.074	-0.064
	(0.089)	(0.092)	(0.091)	(0.094)	(0.078)	(0.095)	(0.171)	(0.166)	(0.082)	(0.097)
RNCCQ	0.403***	0.015	0.119	0.232**	0.146*	0.147	0.299**	0.196	0.259***	0.114
	(0.090)	(0.093)	(0.092)	(0.095)	(0.078)	(0.093)	(0.145)	(0.140)	(0.082)	(0.097)
Male	-0.285	-0.482**	-0.654***	-0.153	0.034	0.269	-0.253	0.791**	0.007	0.258
	(0.183)	(0.190)	(0.188)	(0.194)	(0.160)	(0.193)	(0.370)	(0.358)	(0.168)	(0.199)
Constant	0.105	0.196*	0.250**	0.054	-0.060	-0.088	0.065	-0.270	-0.020	-0.088
	(0.110)	(0.114)	(0.113)	(0.117)	(0.096)	(0.120)	(0.210)	(0.204)	(0.101)	(0.120)
N	110	110	110	110	110	103	31	31	110	110
R^2	0.187	0.062	0.121	0.062	0.038	0.045	0.155	0.197	0.103	0.027

Standard errors in parentheses
* $p < 0.1$, ** $p < 0.05$, *** $p < 0.01$

	Average of coefficients	Coefficients with the predicted sign
RND	-0.002	5/10
RNCCQ	0.193	10/10

Independent variables: RND and separate RNCCQ indices at each age (17, 27, and 37)

OLS regressions for primary outcome variables: coefficients, standard errors, and nominal *p*-value

	(1) Net worth	(2) Perm income	(3) Wealth income ratio	(4) High interest-rate debt (reverse)	(5) Credit card misuse (reverse)	(6) Delay choice	(7) Savings rate	(8) Financial health	(9) Education years	(10) Forward looking behaviors	(11) Social status
RND	0.052	-0.163*	0.158	-0.025	-0.002	0.050	-0.057	-0.109	0.093	0.025	-0.168*
	(0.100)	(0.092)	(0.103)	(0.087)	(0.079)	(0.091)	(0.094)	(0.093)	(0.089)	(0.089)	(0.089)
Age 17 RNCCQ	0.143	0.041	0.077	0.019	-0.019	0.056	0.033	0.060	0.046	0.041	0.072
	(0.117)	(0.107)	(0.121)	(0.102)	(0.092)	(0.107)	(0.107)	(0.109)	(0.104)	(0.104)	(0.105)
Age 27 RNCCQ	-0.005	0.283*	-0.113	0.126	0.270**	0.246*	-0.111	0.033	-0.019	0.288**	0.159
	(0.158)	(0.147)	(0.163)	(0.138)	(0.127)	(0.147)	(0.153)	(0.150)	(0.144)	(0.144)	(0.144)
Age 37 RNCCQ	0.208	0.152	0.050	-0.039	-0.076	-0.158	0.078	0.250*	0.224	0.063	0.016
	(0.155)	(0.145)	(0.160)	(0.136)	(0.125)	(0.145)	(0.146)	(0.148)	(0.141)	(0.142)	(0.142)
Male	0.018	-0.047	0.051	0.100	0.028	-0.041	0.248	0.157	-0.080	-0.608***	0.369*
	(0.207)	(0.192)	(0.213)	(0.181)	(0.165)	(0.192)	(0.193)	(0.195)	(0.187)	(0.187)	(0.188)
Perm income							0.341***				
							(0.104)				
Constant	0.009	0.025	-0.017	-0.103	-0.085	-0.006	-0.066	-0.055	0.029	0.239**	-0.126
	(0.124)	(0.113)	(0.128)	(0.108)	(0.097)	(0.113)	(0.118)	(0.115)	(0.110)	(0.110)	(0.111)
N	106	109	106	106	110	109	103	110	110	110	110
R^2	0.093	0.186	0.028	0.018	0.067	0.047	0.139	0.106	0.082	0.220	0.094

Standard errors in parentheses

* $p < 0.1$, ** $p < 0.05$, *** $p < 0.01$

	Average of coefficients	Coefficients with the predicted sign
RND	-0.013	5/11
Age 17 RNCCQ index	0.052	10/11
Age 27 RNCCQ index	0.105	7/11
Age 37 RNCCQ index	0.070	8/11

Independent variables: RND and separate RNCCQ indices at each age (17, 27, and 37)

OLS regressions for secondary outcome variables: coefficients, standard errors, and nominal *p*-value

	(1) Diet, exercise, and BMI	(2) Smoking and alcohol	(3) Preventative health and dental care	(4) Less procrast-ination	(5) Consider consequences	(6) Savings rate	(7) Verbal SAT	(8) Quant SAT	(9) Education scale	(10) Emotional state & relationship
RND	-0.007	0.086	-0.045	-0.016	0.041	-0.097	-0.083	0.010	0.080	-0.086
	(0.090)	(0.093)	(0.090)	(0.093)	(0.079)	(0.098)	(0.173)	(0.164)	(0.084)	(0.098)
Age 17	0.182*	0.156	0.024	-0.175	-0.009	0.048	0.357	0.396*	0.100	-0.070
RNCCQ	(0.106)	(0.109)	(0.105)	(0.109)	(0.093)	(0.112)	(0.210)	(0.199)	(0.098)	(0.115)
Age 27	0.306**	0.057	0.438***	0.048	-0.011	0.001	-0.005	0.029	0.038	0.243
RNCCQ	(0.146)	(0.150)	(0.145)	(0.149)	(0.127)	(0.156)	(0.261)	(0.247)	(0.135)	(0.158)
Age 37	-0.002	-0.182	-0.312**	0.368**	0.187	0.111	0.016	-0.199	0.147	-0.054
RNCCQ	(0.144)	(0.148)	(0.143)	(0.147)	(0.125)	(0.153)	(0.272)	(0.257)	(0.133)	(0.156)
Male	-0.256	-0.398**	-0.622***	-0.296	-0.009	0.258	0.024	1.221***	0.000	0.221
	(0.190)	(0.196)	(0.189)	(0.195)	(0.166)	(0.203)	(0.456)	(0.431)	(0.176)	(0.206)
Constant	0.094	0.165	0.238**	0.106	-0.045	-0.083	-0.041	-0.437*	-0.018	-0.075
	(0.112)	(0.115)	(0.111)	(0.115)	(0.098)	(0.123)	(0.235)	(0.222)	(0.104)	(0.121)
N	110	110	110	110	110	103	31	31	110	110
R^2	0.200	0.088	0.185	0.132	0.053	0.043	0.210	0.285	0.093	0.046

Standard errors in parentheses
* $p < 0.1$, ** $p < 0.05$, *** $p < 0.01$

	Average of coefficients	Coefficients with the predicted sign
RND	-0.012	4/10
Age 17 RNCCQ index	0.101	7/10
Age 27 RNCCQ index	0.114	8/10
Age 37 RNCCQ index	0.008	5/10

Independent variables: RND and each of the six subscales of the RNCCQ (averaged across ages)

OLS regressions for primary outcome variables: coefficients, standard errors, and nominal p-value

	(1) Net worth	(2) Perm income	(3) Wealth income ratio	(4) High interest-rate debt (reverse)	(5) Credit card misuse (reverse)	(6) Delay choice	(7) Savings rate	(8) Financial health	(9) Education years	(10) Forward looking behaviors	(11) Social status
RND	0.073	-0.110	0.166*	0.008	0.043	0.040	-0.082	-0.076	0.067	0.086	-0.129
	(0.095)	(0.091)	(0.099)	(0.087)	(0.078)	(0.092)	(0.093)	(0.093)	(0.089)	(0.087)	(0.089)
Attention	0.120	0.063	-0.005	0.101	-0.076	-0.120	-0.226*	0.078	-0.032	0.143	-0.027
	(0.123)	(0.116)	(0.128)	(0.113)	(0.100)	(0.118)	(0.116)	(0.119)	(0.114)	(0.112)	(0.114)
Coping	0.011	0.194*	-0.076	0.073	0.121	-0.095	-0.082	0.077	-0.010	0.191*	0.137
	(0.114)	(0.109)	(0.119)	(0.105)	(0.095)	(0.113)	(0.114)	(0.113)	(0.108)	(0.106)	(0.108)
Goal Pursuit	0.244**	0.244**	0.110	0.073	0.124	0.134	0.148	0.200*	0.141	0.039	0.154
	(0.114)	(0.109)	(0.119)	(0.105)	(0.094)	(0.111)	(0.115)	(0.113)	(0.108)	(0.106)	(0.108)
Concern for	-0.180*	-0.098	-0.242**	-0.011	0.088	0.079	0.090	-0.086	-0.015	0.048	-0.127
Others	(0.103)	(0.098)	(0.108)	(0.095)	(0.085)	(0.102)	(0.101)	(0.101)	(0.097)	(0.096)	(0.097)
Delay Ability	0.221**	0.140	0.238**	-0.020	0.110	0.142	0.066	0.143	0.043	0.171*	0.078
	(0.111)	(0.106)	(0.116)	(0.101)	(0.090)	(0.108)	(0.109)	(0.108)	(0.103)	(0.102)	(0.103)
General	-0.034	0.092	-0.089	-0.119	-0.106	0.159*	0.058	-0.017	0.281***	-0.164*	0.068
Cognitive Ability	(0.097)	(0.093)	(0.101)	(0.089)	(0.079)	(0.094)	(0.097)	(0.095)	(0.091)	(0.089)	(0.091)
Male	0.046	-0.079	0.052	0.082	-0.030	-0.037	0.213	0.168	-0.038	-0.613***	0.271
	(0.200)	(0.193)	(0.209)	(0.183)	(0.166)	(0.196)	(0.198)	(0.199)	(0.190)	(0.187)	(0.190)
Perm income							0.334***				
							(0.105)				
Constant	-0.014	0.038	-0.033	-0.097	-0.064	-0.005	-0.053	-0.059	0.014	0.241**	-0.090
	(0.118)	(0.112)	(0.123)	(0.108)	(0.096)	(0.114)	(0.118)	(0.115)	(0.110)	(0.108)	(0.110)
N	106	109	106	106	110	109	103	110	110	110	110
R^2	0.207	0.243	0.129	0.060	0.130	0.072	0.176	0.146	0.126	0.282	0.144

Standard errors in parentheses

* $p < 0.1$, ** $p < 0.05$, *** $p < 0.01$

	Average of coefficients	Coefficients with the predicted sign
RND	0.008	7/11
Attention Subscale	0.002	5/11
Coping Subscale	0.049	7/11
Goal Pursuit Subscale	0.146	11/11
Concern for Others Subscale	-0.041	4/11
Delay Ability Subscale	0.121	10/11
General Cognitive Ability Subscale	0.012	5/11

Independent variables: RND and each of the six subscales of the RNCCQ (averaged across ages)

OLS regressions for secondary outcome variables: coefficients, standard errors, and nominal *p*-value

	(1) Diet, exercise, and BMI	(2) Smoking and alcohol	(3) Preventative health and dental care	(4) Less procrast-ination	(5) Consider consequences	(6) Savings rate	(7) Verbal SAT	(8) Quant SAT	(9) Education scale	(10) Emotional state & relationship
RND	0.027	0.083	-0.017	0.059	0.080	-0.107	-0.038	0.084	0.055	-0.024
	(0.087)	(0.095)	(0.095)	(0.090)	(0.077)	(0.097)	(0.135)	(0.163)	(0.083)	(0.097)
Attention	0.039	-0.024	-0.051	0.276**	0.164	-0.202*	0.524***	-0.020	-0.009	0.175
	(0.112)	(0.123)	(0.122)	(0.115)	(0.099)	(0.121)	(0.168)	(0.203)	(0.107)	(0.125)
Coping	-0.005	0.006	0.028	0.282**	0.185*	-0.023	0.417*	0.500*	0.078	0.214*
	(0.106)	(0.116)	(0.115)	(0.109)	(0.094)	(0.118)	(0.208)	(0.251)	(0.101)	(0.118)
Goal Pursuit	0.171	-0.100	-0.014	0.069	-0.005	0.216*	-0.227	-0.281	0.106	-0.132
	(0.106)	(0.116)	(0.115)	(0.109)	(0.094)	(0.118)	(0.184)	(0.222)	(0.101)	(0.118)
Concern for Others	0.062	0.107	0.096	-0.068	-0.081	0.064	-0.471**	-0.305	-0.001	-0.024
	(0.095)	(0.104)	(0.104)	(0.098)	(0.084)	(0.106)	(0.187)	(0.226)	(0.091)	(0.106)
Delay Ability	0.397***	0.067	0.136	-0.119	0.029	0.113	-0.044	0.324*	-0.018	0.027
	(0.101)	(0.111)	(0.110)	(0.104)	(0.090)	(0.113)	(0.138)	(0.167)	(0.096)	(0.113)
General Cognitive Ability	-0.052	-0.083	0.020	-0.233**	-0.146*	0.097	0.340**	0.119	0.297***	-0.212**
	(0.089)	(0.097)	(0.097)	(0.091)	(0.079)	(0.100)	(0.126)	(0.152)	(0.084)	(0.099)
Male	-0.240	-0.475**	-0.654***	-0.237	-0.016	0.214	-0.320	0.471	-0.018	0.208
	(0.186)	(0.204)	(0.203)	(0.192)	(0.165)	(0.207)	(0.327)	(0.396)	(0.177)	(0.207)
Constant	0.089	0.193	0.250**	0.085	-0.042	-0.065	0.070	-0.177	-0.011	-0.070
	(0.108)	(0.118)	(0.117)	(0.111)	(0.095)	(0.124)	(0.172)	(0.208)	(0.103)	(0.120)
N	110	110	110	110	110	103	31	31	110	110
R^2	0.290	0.089	0.135	0.223	0.137	0.087	0.600	0.408	0.152	0.108

Standard errors in parentheses
* $p < 0.1$, ** $p < 0.05$, *** $p < 0.01$

	Average of coefficients	Coefficients with the predicted sign

RND	0.020	6/10
Attention Subscale	0.087	5/10
Coping Subscale	0.168	8/10
Goal Pursuit Subscale	-0.020	4/10
Concern for Others Subscale	-0.062	4/10
Delay Ability Subscale	0.091	7/10
General Cognitive Ability Subscale	0.015	5/10

3. Analysis of diagnostic vs. non-diagnostic conditions

- We run the primary analyses with the key independent variable RND for the diagnostic condition (rewards exposed/spontaneous ideation) separately from the non-diagnostic conditions (pooling conditions with suggested ideation and/or rewards covered) (see Shoda, Mischel and Peake, 1990). We predicted larger effects in the diagnostic condition and test this in two ways:
    - After orienting the coefficients such that higher values correspond to higher self-control, we calculate the average of coefficients across the outcome variables for the diagnostic and non-diagnostic subgroups separately. We bootstrap standard errors for these averages and test for equality across groups by testing whether the difference in averages is statistically distinguishable from 0. [Note: in the pre-registration document we proposed an F-test to test whether the sum of coefficients in the diagnostic condition is significantly different from the sum of coefficients in the non-diagnostic conditions]
    - We also test the differential predictive power of wait time in the diagnostic condition by testing the following model on the full sample where y represents each of the primary outcome variables and *diagnostic* is a binary indicator for being in the diagnostic condition. β_3 is the coefficient of interest in these secondary tests
        - $y = \alpha + \beta_1 \times delay\ ability + \beta_2 \times diagnostic + \beta_3 \times delay\ ability\ \times diagnostic\ + \beta_4 \times\ male$
- For these secondary analyses, we report coefficients, standard errors, and nominal p-values for the coefficients, average of the coefficients across the outcome variables with bootstrapped standard errors, as well as the p-value from the z-test described above.

Sample – diagnostic group only

Measure of self-regulation is RND: Expected deviation from predicted wait time in uncensored log seconds

OLS Regressions: coefficients, standard errors, and nominal p-value

	(1) Net worth	(2) Perm income	(3) Wealth income ratio	(4) High interest-rate debt (reverse)	(5) Credit card misuse (reverse)	(6) Delay choice	(7) Savings rate	(8) Financial health	(9) Education years	(10) Forward looking behaviors	(11) Social status
RND	0.185	-0.048	0.227	0.026	-0.034	0.277*	-0.104	-0.019	0.103	0.123	-0.000
	(0.153)	(0.159)	(0.158)	(0.117)	(0.118)	(0.151)	(0.165)	(0.142)	(0.142)	(0.130)	(0.156)
Male	-0.209	-0.206	-0.023	-0.322	0.360	-0.393	0.111	0.270	0.068	-0.585*	0.206
	(0.373)	(0.390)	(0.386)	(0.287)	(0.293)	(0.375)	(0.392)	(0.353)	(0.351)	(0.323)	(0.386)
Perm income							0.295				
							(0.199)				
Constant	0.053	-0.049	0.070	0.168	-0.095	0.084	0.002	-0.171	-0.095	0.149	-0.039
	(0.223)	(0.231)	(0.231)	(0.172)	(0.172)	(0.224)	(0.261)	(0.207)	(0.206)	(0.190)	(0.226)
N	32	33	32	32	34	33	29	34	34	34	34
R^2	0.051	0.015	0.068	0.042	0.047	0.117	0.093	0.019	0.020	0.107	0.009

Standard errors in parentheses

* $p < 0.1$, ** $p < 0.05$, *** $p < 0.01$

	Average of coefficients (bootstrapped SE)	Ex-post analysis: p-value of test comparing average to 0	Coefficients with the predicted sign
RND – diagnostic group	0.067 (0.087)	p=0.443	6/11

Sample – non-diagnostic group only

Measure of self-regulation is RND: Expected deviation from predicted wait time in uncensored log seconds

OLS Regressions: coefficients, standard errors, and nominal p-value

	(1) Net worth	(2) Perm income	(3) Wealth income ratio	(4) High interest-rate debt (reverse)	(5) Credit card misuse (reverse)	(6) Delay choice	(7) Savings rate	(8) Financial health	(9) Education years	(10) Forward looking behaviors	(11) Social status
RND	0.035	-0.075	0.092	-0.026	0.078	-0.055	-0.031	-0.108	0.168	0.077	-0.245**
	(0.131)	(0.127)	(0.128)	(0.113)	(0.104)	(0.111)	(0.113)	(0.128)	(0.116)	(0.128)	(0.110)
Male	0.042	0.015	0.022	0.274	-0.049	-0.031	0.339	0.154	-0.110	-0.644***	0.343*
	(0.239)	(0.232)	(0.234)	(0.206)	(0.190)	(0.203)	(0.205)	(0.234)	(0.212)	(0.233)	(0.200)
Perm income							0.362***				
							(0.106)				
Constant	-0.013	0.043	-0.037	-0.220*	-0.087	-0.043	-0.106	-0.042	0.102	0.265*	-0.177
	(0.150)	(0.143)	(0.147)	(0.129)	(0.118)	(0.126)	(0.128)	(0.145)	(0.131)	(0.144)	(0.124)
N	77	79	77	77	79	79	77	79	79	79	79
R^2	0.001	0.005	0.007	0.024	0.008	0.004	0.169	0.014	0.029	0.093	0.090

Standard errors in parentheses
* $p < 0.1$, ** $p < 0.05$, *** $p < 0.01$

	Average of coefficients (bootstrapped SE)	Ex-post analysis: p-value of test comparing average to 0	Coefficients with the predicted sign
RND – non-diagnostic group	-0.008 (0.057)	*p*=0.888	5/11

p-value from z-test that the difference between the average of coefficients in the diagnostic vs. non-diagnostic group is 0: 0.473

Interaction with dummy for diagnostic group

Measure of self-regulation is RND: Expected deviation from predicted wait time in uncensored log seconds
OLS Regressions: coefficients, standard errors, and nominal p-value

	(1) Net worth	(2) Perm income	(3) Wealth income ratio	(4) High interest-rate debt (reverse)	(5) Credit card misuse (reverse)	(6) Delay choice	(7) Savings rate	(8) Financial health	(9) Education years	(10) Forward looking behaviors	(11) Social status
RND	0.038	-0.073	0.093	-0.019	0.074	-0.051	-0.029	-0.109	0.167	0.077	-0.244^{**}
	(0.130)	(0.129)	(0.129)	(0.110)	(0.104)	(0.117)	(0.118)	(0.126)	(0.118)	(0.123)	(0.117)
Diagnostic Condition	-0.027	-0.170	0.090	0.167	0.136	-0.003	0.031	-0.089	-0.134	-0.096	0.089
	(0.214)	(0.212)	(0.213)	(0.181)	(0.170)	(0.193)	(0.206)	(0.206)	(0.193)	(0.201)	(0.191)
Diagnostic x RND	0.131	0.013	0.131	0.008	-0.089	0.313^{*}	-0.084	0.095	-0.056	0.048	0.238
	(0.199)	(0.197)	(0.198)	(0.168)	(0.157)	(0.177)	(0.186)	(0.191)	(0.179)	(0.186)	(0.177)
Male	-0.029	-0.048	0.009	0.106	0.069	-0.135	0.277	0.188	-0.058	-0.627^{***}	0.303^{*}
	(0.201)	(0.199)	(0.199)	(0.170)	(0.160)	(0.180)	(0.181)	(0.194)	(0.181)	(0.189)	(0.180)
Perm income							0.343^{***}				
							(0.093)				
Constant	0.015	0.067	-0.032	-0.154	-0.132	-0.004	-0.080	-0.055	0.082	0.259^{*}	-0.161
	(0.141)	(0.137)	(0.139)	(0.119)	(0.111)	(0.125)	(0.126)	(0.135)	(0.126)	(0.131)	(0.125)
N	109	112	109	109	113	112	106	113	113	113	113
R^2	0.012	0.013	0.030	0.012	0.013	0.040	0.144	0.017	0.026	0.097	0.060

Standard errors in parentheses
* $p < 0.1$, ** $p < 0.05$, *** $p < 0.01$

	Average of coefficients	Coefficients with the predicted sign
Diagnostic x RND	0.068	8/11

C. Ex-post analyses

In this section we report the results from the following ex-post analyses. These analyses were run after looking at the results from our pre-registered primary and secondary analyses. We further identify ex-post analyses that were conducted as part of the editorial process.

1. We report the correlation between RND and the RNCCQ (averaged across ages)
2. We calculate pairwise correlations between RND and the RNCCQ indices at each age, reapplying the inverse normal transformation to the relevant comparison groups
3. Regress each of the primary outcome variables on each of the 85 items in the RNCCQ index (23 in the 1984 index and 31 in each of the 1993 and 2003 surveys) in separate regressions (i.e., 85 CCQ items x 11 outcomes = 935 regressions). We report the average coefficient (sign-adjusted) for all CCQ items and the average coefficient (sign-adjusted) among CCQ items in the same year
4. Regress each of the primary outcome variables on RNCCQ indices in each year separately (i.e., 3 RNCCQ indices x 11 outcomes = 33 regressions)
5. To compare the survey subsample to the full Bing sample, we run OLS regressions of each variable from the original delay experiment on a constant and dummy indicator for being in the survey subsample. We also do the same analysis comparing the full sample to each of the follow-up waves at age 17, 27, and 37.
6. We test BMI as an additional outcome variable, running regressions of BMI on both of the primary explanatory variables (RNSRI and RND)
7. We compare the results from the BMI analyses above with the results obtained in previous follow-up assessments of BMI in the Bing sample
8. We look at the correlation between delay and BMI separately in the diagnostic and non-diagnostic subsamples – analysis resulted from editorial processes
9. We regress each of the primary outcome variables (except percent of later choices) on percent of later choices variable
10. We test the robustness of our primary analyses to including age at the time of the economic survey as an additional control variable
11. We aggregate the 11 primary outcome variables to construct a capital formation index and regress the index on RND and RNSRI. We also regress the index on RND and the RNCCQ broken down first by age and then by subscale
12. We report the correlations between all primary outcome variables and components of the RNSRI. These results are reported in an Excel file “Correlation Table.xlsx”
13. We examine the effects of the normalizing transformations on the correlation between RND and Age 17 (1984) RNCCQ index
14. Independent variable is RNCCQ – analysis resulted from editorial processes

1. Correlation between RND and the RNCCQ

0.16 (SE=0.09, *p*-value=0.09, corresponds to cell M16 in the Online Appendix Excel file “Correlation Table.xlsx”)

N=110 subjects in the survey subsample with a RND and RNCCQ (i.e., responded to at least one follow-up survey at age 17, 27, or 37).

2. Relationships between RND and RNCCQ indices at each age (pairwise)

This table reports OLS regression coefficients and standard errors in parentheses. Details of the analysis:

- Uses the full sample of 543 subjects with RND (wait time, condition categorization, and age at wait task)
- No imputation for missing RNCCQ indices
- All variables are rank-normalized within the relevant comparison group (i.e., within each cell)

	**RND**	**Age 17 RNCCQ index** (Parental rating)	**Age 27 RNCCQ index** (Self-rating)
**Age 17 RNCCQ index** (Parental rating)	0.13^{*} (0.08) N=163		
**Age 27 RNCCQ index** (Self-rating)	0.23^{***} (0.08) N=151	0.39^{***} (0.10) N=82	
**Age 37 RNCCQ index** (Self-rating)	0.16^{*} (0.09) N=134	0.27^{**} (0.12) N=69	0.63^{***} (0.09) N=71

$^{*}\ p < 0.1$, $^{**}\ p < 0.05$, $^{***}\ p < 0.01$

This table reports OLS standardized regression coefficients and standard errors in parentheses. Details of the analysis:

- Uses the sample of 110 subjects in the survey subsample with RND who participated in any of the surveys at age 17, 27, or 37 (this excludes 3 subjects in the survey subsample who did not participate in any of the earlier follow-up surveys)
- Missing RNCCQ indices are imputed from available years (see Detailed variable definitions and summary statistics: self-regulation for details)
- RNCCQ indices are rank-normalized within the sample of 110. RND is rank-normalized within the full survey sample of 113
- This table reports correlations that correspond to those reported in the Excel file "Correlation Table.xlsx" in cells M17-M19, O18-O19, and P19

	**RND**	**Age 17 RNCCQ index** (Parental rating)	**Age 27 RNCCQ index** (Self-rating)
**Age 17 RNCCQ index** (Parental rating)	0.06<br>(0.10)<br>N=110		
**Age 27 RNCCQ index** (Self-rating)	0.23***<br>(0.09)<br>N=110	0.48***<br>(0.08)<br>N=110	
**Age 37 RNCCQ index** (Self-rating)	0.15<br>(0.10)<br>N=110	0.47***<br>(0.08)<br>N=110	0.78***<br>(0.06)<br>N=110

* $p < 0.1$, ** $p < 0.05$, *** $p < 0.01$

3. Average of coefficients from regressions of each question in the RNCCQ index individually and the primary outcomes

We regress each of the primary outcome variables on each of the 85 items in the RNCCQ index (23 in the 1984 index and 31 in each of the 1993 and 2003 surveys) in separate regressions (i.e., 85 CCQ items x 11 outcomes = 935 regressions). CCQ items are rank-normalized. We report the average coefficient (sign-adjusted) for all rank normalized CCQ items and the average coefficient (sign-adjusted) among rank-normalized CCQ items in the same year. Bootstrapped standard errors are reported in parentheses.

	Average of coefficients across CCQ items and outcomes (bootstrapped SE)	*p*-value
All CCQ items	0.07 (0.03)	*p*=0.003
Age 17 CCQ items	0.08 (0.05)	*p*=0.116
Age 27 CCQ items	0.07 (0.02)	*p*=0.006
Age 37 CCQ items	0.08 (0.02)	*p*=0.00005

4. Average of coefficients from regressions of RNCCQ at each age in separate regressions

We regress each of the primary outcome variables on RNCCQ indices in each year separately (i.e., 3 RNCCQ indices x 11 outcomes = 33 regressions), without imputing missing RNCCQ indices. All regressions control for sex. Regressions with savings rate as the outcome also control for permanent income. We report the average coefficient across the 11 primary outcome variables for each of the three RNCCQ indices. We also report the individual coefficients, standard errors, and nominal *p*-values in the tables below.

	Average of coefficients across primary outcomes (bootstrapped SE)	N
Age 17 RNCCQ	0.15 (0.09)	52
Age 27 RNCCQ	0.17 (0.06)	71
Age 37 RNCCQ	0.19 (0.05)	86

Regression of primary outcomes on age 17 RNCCQ index

OLS Regressions: coefficients, standard errors, and nominal *p*-value

Note: Only includes subjects for whom we had an age 17 RNCCQ index

	(1) Net worth	(2) Perm income	(3) Wealth income ratio	(4) High interest-rate debt (reverse)	(5) Credit card misuse (reverse)	(6) Delay choice	(7) Savings rate	(8) Financial health	(9) Education years	(10) Forward looking behaviors	(11) Social status
Age 17 RNCCQ (No Imputation)	0.337**	0.176	0.143	0.151	0.114	0.101	0.020	0.179	0.012	0.351**	0.033
	(0.161)	(0.131)	(0.159)	(0.134)	(0.127)	(0.129)	(0.138)	(0.130)	(0.122)	(0.139)	(0.125)
Male	0.640**	0.399	0.386	0.291	0.030	0.157	0.484*	0.684**	-0.339	0.037	0.679**
	(0.317)	(0.272)	(0.313)	(0.263)	(0.264)	(0.268)	(0.287)	(0.270)	(0.253)	(0.288)	(0.259)
Perm income							0.225				
							(0.147)				
Constant	-0.316*	-0.110	-0.279	-0.158	-0.004	-0.055	-0.027	-0.261*	0.209	0.115	-0.201
	(0.181)	(0.155)	(0.179)	(0.150)	(0.150)	(0.152)	(0.160)	(0.153)	(0.143)	(0.163)	(0.147)
N	51	52	51	51	52	52	52	52	52	52	52
R^2	0.119	0.057	0.036	0.038	0.017	0.015	0.121	0.121	0.041	0.124	0.129

Standard errors in parentheses
* $p < 0.1$, ** $p < 0.05$, *** $p < 0.01$

Average of the coefficients: 0.147
Coefficients with the predicted sign: 11/11

Regression of primary outcomes on age 27 RNCCQ index

OLS Regressions: coefficients, standard errors, and nominal *p*-value

Note: Only includes subjects for whom we had an age 27 RNCCQ index

	(1) Net worth	(2) Perm income	(3) Wealth income ratio	(4) High interest-rate debt (reverse)	(5) Credit card misuse (reverse)	(6) Delay choice	(7) Savings rate	(8) Financial health	(9) Education years	(10) Forward looking behaviors	(11) Social status
Age 27 RNCCQ (No Imputation)	0.231*	0.393***	0.034	0.147	0.235**	0.093	-0.211*	0.164	0.151	0.469***	0.163
	(0.127)	(0.110)	(0.123)	(0.097)	(0.091)	(0.114)	(0.119)	(0.117)	(0.113)	(0.114)	(0.114)
Male	0.201	-0.188	0.409	0.163	0.309	-0.193	0.237	0.067	-0.309	-0.488**	0.213
	(0.268)	(0.234)	(0.260)	(0.206)	(0.193)	(0.241)	(0.225)	(0.248)	(0.238)	(0.240)	(0.242)
Perm income							0.463***				
							(0.132)				
Constant	-0.128	0.033	-0.203	-0.008	-0.109	0.120	-0.071	-0.045	0.143	0.106	-0.211
	(0.155)	(0.133)	(0.150)	(0.119)	(0.110)	(0.138)	(0.133)	(0.141)	(0.136)	(0.137)	(0.138)
N	69	71	69	69	71	70	66	71	71	71	71
R^2	0.055	0.165	0.037	0.041	0.117	0.020	0.176	0.029	0.050	0.242	0.039

Standard errors in parentheses
* $p < 0.1$, ** $p < 0.05$, *** $p < 0.01$

Average of the coefficients: 0.170
Coefficients with the predicted sign: 10/11

Regression of primary outcomes on age 37 RNCCQ index

OLS Regressions: coefficients, standard errors, and nominal *p*-value

Note: Only includes subjects for whom we had an age 37 RNCCQ index

	(1) Net worth	(2) Perm income	(3) Wealth income ratio	(4) High interest-rate debt (reverse)	(5) Credit card misuse (reverse)	(6) Delay choice	(7) Savings rate	(8) Financial health	(9) Education years	(10) Forward looking behaviors	(11) Social status
Age 37 RNCCQ (No Imputation)	0.309***	0.357***	0.052	0.104	0.180**	0.086	0.070	0.306***	0.219**	0.268***	0.141
	(0.104)	(0.101)	(0.106)	(0.095)	(0.090)	(0.101)	(0.106)	(0.102)	(0.094)	(0.099)	(0.090)
Male	-0.174	-0.188	-0.055	0.132	-0.141	-0.061	0.120	0.053	0.048	-0.575***	0.260
	(0.216)	(0.209)	(0.219)	(0.196)	(0.186)	(0.208)	(0.206)	(0.211)	(0.193)	(0.206)	(0.187)
Perm income							0.245**				
							(0.114)				
Constant	0.186	0.181	0.100	-0.103	-0.034	-0.018	0.009	0.093	0.050	0.320**	0.095
	(0.131)	(0.126)	(0.133)	(0.119)	(0.111)	(0.125)	(0.128)	(0.127)	(0.116)	(0.123)	(0.112)
N	84	85	84	84	86	86	81	86	86	86	86
R^2	0.101	0.136	0.003	0.022	0.050	0.009	0.088	0.100	0.064	0.143	0.055

Standard errors in parentheses
* $p < 0.1$, ** $p < 0.05$, *** $p < 0.01$

Average of the coefficients: 0.190
Coefficients with the predicted sign: 11/11

5. Comparison of the Bing Sample over time: compare full sample of 543 to the subsamples surveyed in the economic survey and age ages 17, 27, and 37

To compare the survey subsample (N=113) to the full Bing sample (N=543 with wait time, condition categorization, and age at wait task), we run OLS regressions of each variable from the original delay experiment on a constant and dummy indicator for being in the survey subsample. We report the means of each variable for the full sample and the survey subsample as well as the *p*-value of the coefficient on the indicator variable indicating if the subject is in the survey subsample.

Note: These variables are not rank-normalized. 7 subjects in the original Bing sample of 550 are excluded from this analysis as they are missing age at wait task and therefore deviation from predicted preschool wait time.

**Variable**	**Survey Subsample (mean or percent)**	**Full Sample (mean or percent)**	***p*-value from OLS regression coefficient on Survey Subsample indicator variable**
Deviation from predicted preschool wait time (ln seconds)	0.08	0.00	0.657
Deviation from predicted preschool wait time (seconds)	40.32	-0.08	0.305
Original deviation from condition mean wait time (seconds)	31.88	2.09	0.307
Raw wait time (seconds)	509.7	470.1	0.198
Male	37%	48%	0.009
Age at wait task (months)	51.6	52.1	0.364

Using the same approach, we compare the full Bing sample to the subsamples that participated in the follow-ups ate age 17, 27, and 37 (N=163, N=151, and N=134, respectively). In the table below we report the mean for each subsample and in brackets the *p*-value of the coefficient on the indicator variable indicating if the subject is in the given subsample.

**Variable**	**Age 17 Sample**	**Age 27 Sample**	**Age 37 Sample**
Deviation from predicted preschool wait time (ln seconds)	0.22 [*p*=0.143]	0.05 [*p*=0.725]	0.18 [*p*=0.302]
Deviation from predicted preschool wait time (seconds)	63.28 [*p*=0.040]	7.45 [*p*=0.817]	39.38 [*p*=0.264]
Original deviation from condition mean wait time (seconds)	55.60 [*p*=0.019]	17.35 [*p*=0.527]	47.47 [*p*=0.082]
Raw wait time (seconds)	516.1 [*p*=0.056]	497.5 [*p*=0.282]	529.6 [*p*=0.031]
Male	45% [*p*=0.317]	38% [*p*=0.005]	42% [*p*=0.094]
Age at wait task (months)	51.9 [*p*=0.732]	52.2 [*p*=0.817]	52.5 [*p*=0.282]

Number of people reporting in each sample

Number of Follow-Up Surveys Participated In	Follow-Up Surveys Participated In	N
0		266
1	Age 17	59
	Age 27	31
	Age 37	14
	Econ	3
2	Age 17 & 27	26
	Age 17 & 37	11
	Age 17 & Econ	1
	Age 27 & 37	8
	Age 27 & Econ	15
	Age 37 & Econ	28
3	Age 17, 27, & 37	15
	Age 17, 27, & Econ	8
	Age 17, 37, & Econ	10
	Age 27, 37, & Econ	15
4	Age 17, 27, 37, & Econ	33
Total		543

6. BMI as an outcome variable

We include BMI as an additional outcome variable, running regressions of BMI on both of the primary explanatory variables (RNSRI and RND). All variables except for male dummy are standardized by subtracting mean and dividing by SD.

OLS Regressions: standardized coefficients, standard errors, and nominal *p*-value

	(1)<br>BMI	(2)<br>BMI
RND	-0.027	
	(0.090)	
RNSRI		-0.297***
		(0.087)
Male	0.692***	0.696***
	(0.186)	(0.180)
Constant	-0.257**	-0.271**
	(0.113)	(0.108)
Observations	113	110
R^2	0.112	0.203

Standard errors in parentheses
* $p < 0.1$, ** $p < 0.05$, *** $p < 0.01$

7. Comparison with results from analyses of BMI data obtained in previous follow-ups of the Bing sample

In this section, we compare the results reported above with the results previously reported in Schlam et al. (2013). The earlier data were based on two follow-up mailing sent to participants between 2002 and 2004.

<u>The sample reported in Schlam et al. (2013)</u>

The first mailing was sent in December 2002/January 2003 to all participants for whom we had a valid address (*N* = 306). The second was mailed approximately 17 months later in May 2004. 146 Ss responded to the first mailing; 97 Ss responded to the second mailing. The total sample used for the analyses was 164 Ss who reported height and weight at either the first follow-up and/or the second follow-up. (For the 79 Ss responded to both follow-ups, the BMI scores from the two follow-ups were averaged to create a single BMI score for these Ss). One data point from the first follow-up and two from the second follow-up were excluded because these women had recently been pregnant.

<u>The overlap between the Schlam et al. (2013) sample and the current sample</u>

There were 20 Ss in the economics follow-up for whom there are no BMI data from sample reported in Schlam et al, 2013. In addition, 71 Ss in follow-up reported in Schlam et al., 2013, did not participate in the current follow-up. To summarize, this leaves 93 Ss with data from both follow ups (i.e., the current follow-up and the follow-up reported in Schlam et al., 2013).

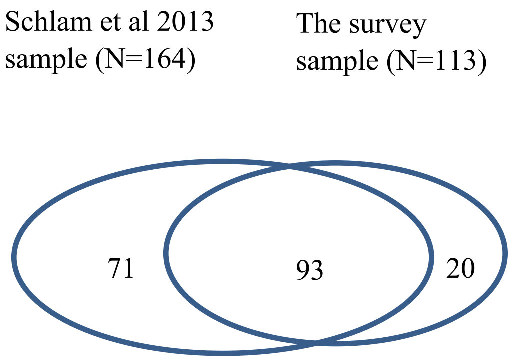

There are three ways in which the analyses if BMI data from the current, economics, survey differ from those reported in Schlam et al. (2013): (1) they represent different subsets of the larger Bing sample; (2) the BMI data reported in Schlam et al. (2013) were collected over a decade before the present BMI data; and (3) the present analysis used RND, rather than the preschool wait times centered on the mean wait times for each type of waiting condition. The table below reports results of analyses in which these three aspects were varied one by one, in order to illustrate the effect of varying each aspect. Specifically, the first row reports the regression coefficient predicting the BMI obtained in 2002-2004, using centered (i.e., "deviation") wait time, in the sample reported in Schlam et al. (2013). The second row reports the results of the same analysis, but only among the 93 participants who are present in both the 2002-2004 BMI data collection and the present economics survey.

Decomposition of Changes in Delay-BMI Relation (bold face shows what is changed from the row above)

Sample (Data available in …)	β [*p*-value]	Predictor	Criterion
164 Ss (2002-2004)	-.190 [*p*=.009]	Delay Deviation	BMI 2002-2004
**93 Ss (2002-2004 and 2012)**	-.085 [*p*=.364]	Delay Deviation	BMI 2002-2004
93 Ss (2002-2004 and 2012)	-.072 [*p*=.462]	Delay Deviation	**BMI 2012**
93 Ss (2002-2004 and 2012)	-.066 [*p*=.507]	**RND**	BMI 2012
**113 Ss (2012)**	-.027 [*p*=.762]	RND	BMI 2012

Note. Coefficients were obtained from hierarchical regression analyses in which predictor variables listed above were entered in Step 2. Sex was entered as a predictor in Step 1. As such, all coefficients reported represent the relation between predictor and criterion variables after controlling for sex. βs represent predicted change in BMI (in *SD*s) from a one *SD* change in delay (e.g., β = -.190 means for every one *SD* increase in preschool delay, there is a predicted .190 *SD* decrease in BMI).

The first row of the table above shows that among the 164 participants whose BMI scores were obtained in 2002 to 2004, there was a highly significant relation between preschool delay and BMI when the participants were in their 30s. Of these 164 participants, 93 responded to the present, economics, survey. As shown in the second row, among these 93 participants, BMI scores were not significantly predicted from their preschool waiting behavior, even though both the predictor and criterion variables were the same as the analysis reported in the first row. As can be seen by comparing the second row of the table with the third row, using the newly obtained BMI scores for these 93 participants results in an almost identical result. The fourth row of the table reports the results obtained after applying the rank normalization transformation we used for the results reported in the primary analysis section. Finally, the fifth row shows the same analysis reported in the fourth row, among the 113 participants in the present economics survey.

Temporal stability in BMI

Among the 93 participants for whom we have BMI scores from 2002-2004 and 2012, the temporal stability correlation was .872, *p*<.001. BMI appears to be relatively stable between the two follow-ups. This also supports the reliability of BMI scores.

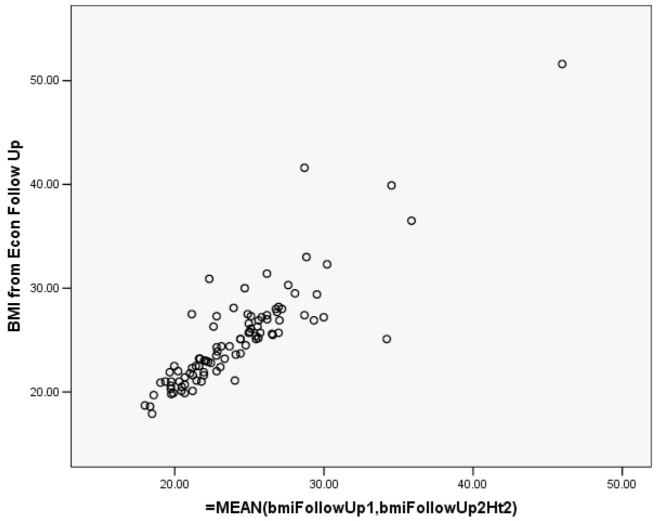

To shed light on the differences between the 2002-2004 and 2012 samples, we made scatterplots of all 164 Ss from the 2002-2004 data, and indicated which of these subjects are also included in the 2012 sample (represented in green).

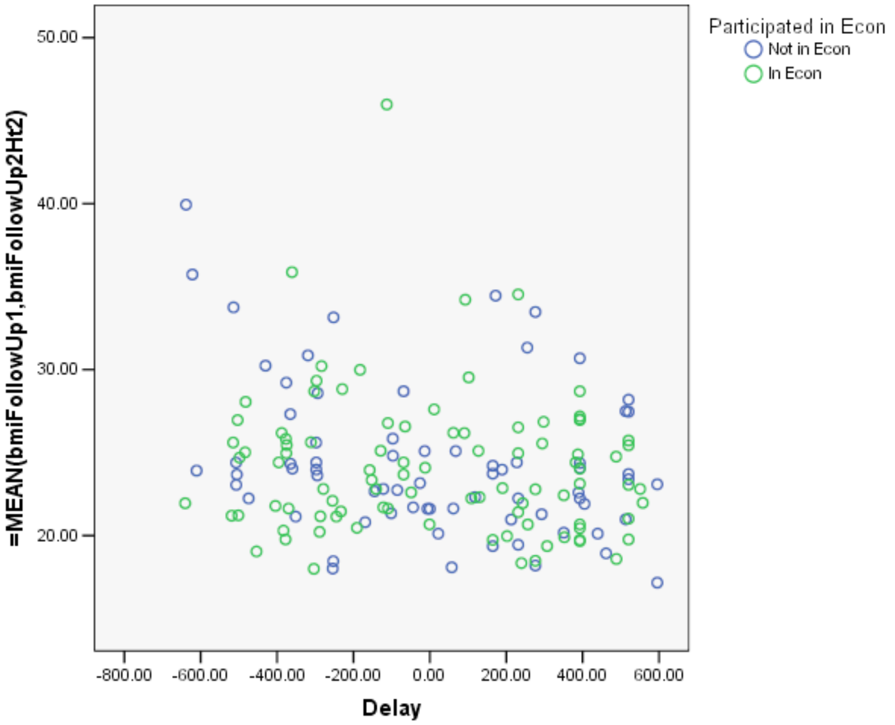

The scatterplot suggests the sample reported in Schlam et al., 2013 included a few participants with extreme BMI scores but who did not participate in the economics follow-up. As such, we explored the possibility that the observed delay-BMI relation would be attenuated if these more extreme scores were pulled in. Specifically, we Winsorized (at the 5th and 95th percentiles) the BMI data from Schlam et al. (2013). Using the Winsorized BMI variable, the delay-BMI relation remained significant (r= -.193, p = .013).

In summary, the difference between the results reported in Schlam et al. (2013) and those obtained in the present economics follow-up appears to be due primarily to the fact that those participants who contributed to the significant relations between preschool delay and BMI in adulthood, as reported in 2013, did not respond to the present, economics, survey.

8. Correlation of delay and BMI data in diagnostic and non-diagnostic subsamples – analysis resulted from editorial processes

We regress BMI (as measured in 2002-2004) on the original measure of delay deviation and sex separately in the diagnostic and non-diagnostic subsamples. Delay deviation is defined as the individual wait time minus the individual's condition mean wait time. BMI and original delay deviation are standardized to mean 0, SD 1 in the full sample of 164.

	BMI (2002, standardized)		
	(1) Full Sample	(2) Diagnostic Subsample	(3) Non-Diagnostic Subsample
dev1st (standardized)	-0.190***	-0.032	-0.239***
	(0.072)	(0.138)	(0.084)
sex	-0.712***	-0.730**	-0.717***
	(0.145)	(0.296)	(0.168)
Constant	0.404***	0.533**	0.369***
	(0.109)	(0.244)	(0.123)
Observations	164	37	127
R^2	0.166	0.152	0.183

Standard errors in parentheses
* $p < 0.1$, ** $p < 0.05$, *** $p < 0.01$

To verify these results are not influenced by outliers, below we explore the effect of winsorizing the data at the 5th and 95th percentile. In the first row, we plot a histogram of the delay deviation for the subsample using either the original delay deviation or a version winsorized at the 5th and 95th percentile of the subgroup (diagnostic or non-diagnostic). Similarly, the second row plots the original or winsorized BMI. The third row displays a scatter plot of delay deviation and BMI along with the correlation. Winsorizing the data does not have a large effect on the correlation between delay deviation and BMI in either the diagnostic or non-diagnostic conditions.

Diagnostic Condition

Original Data | Winsorized

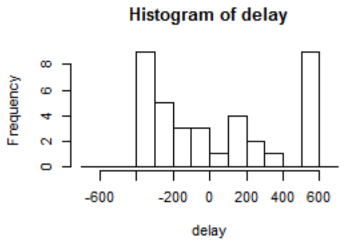

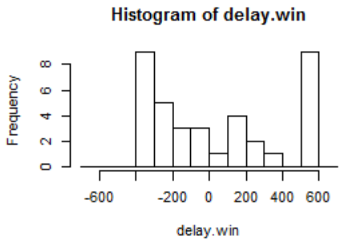

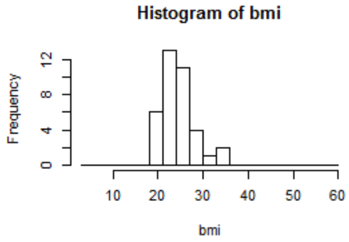

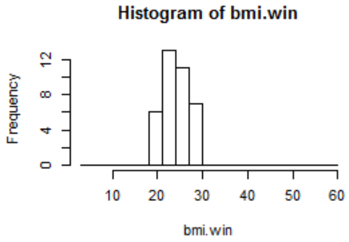

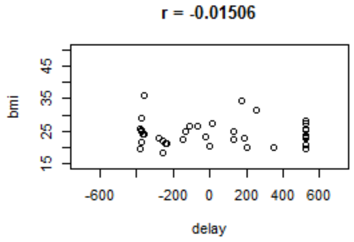

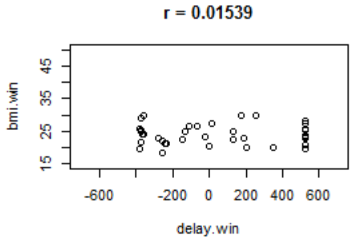

Non-Diagnostic Conditions

Original Data | Winsorized

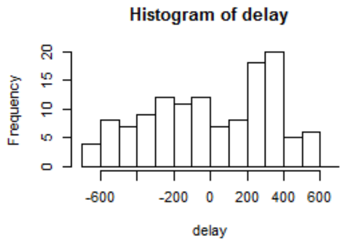

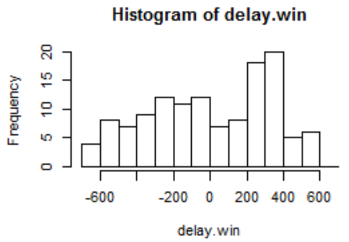

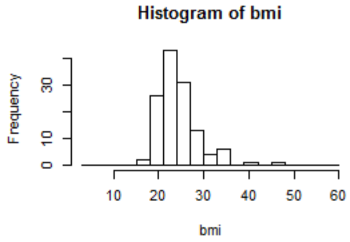

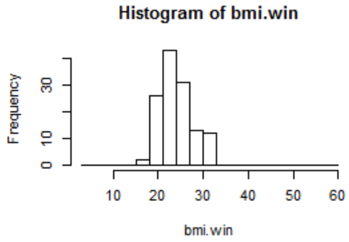

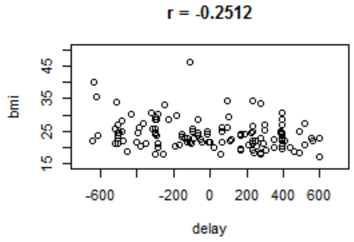

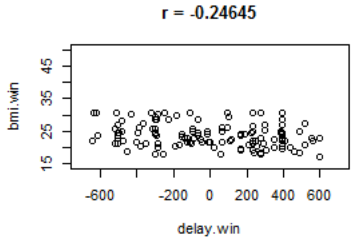

9. Delay choice as independent variable

We regress each of the primary outcome variables (except delay choice) on delay choice, controlling for sex.

Measure of self-regulation is <u>delay choice</u>: rank-normalized percent of later choices

OLS Regressions: coefficients, standard errors, and nominal *p*-value

	(1)<br>Net worth	(2)<br>Perm income	(3)<br>Wealth income ratio	(4)<br>High interest-rate debt (reverse)	(5)<br>Credit card misuse (reverse)	(6)<br>Savings rate	(7)<br>Financial health	(8)<br>Education years	(9)<br>Forward looking behaviors	(10)<br>Social status
Delay choice	0.186*	0.136	0.142	0.086	0.084	0.139	0.124	0.100	0.054	0.062
	(0.103)	(0.102)	(0.104)	(0.089)	(0.084)	(0.096)	(0.100)	(0.093)	(0.100)	(0.094)
Male	-0.006	-0.073	0.038	0.105	0.068	0.270	0.165	-0.052	-0.608***	0.257
	(0.194)	(0.193)	(0.195)	(0.168)	(0.158)	(0.179)	(0.189)	(0.177)	(0.189)	(0.177)
Perm income						0.303***				
						(0.094)				
Constant	0.026	0.050	0.007	-0.097	-0.087	-0.067	-0.044	0.059	0.234**	-0.080
	(0.120)	(0.119)	(0.121)	(0.104)	(0.097)	(0.113)	(0.115)	(0.108)	(0.115)	(0.108)
Observations	108	111	108	108	112	105	112	112	112	112
R^2	0.030	0.018	0.018	0.012	0.010	0.141	0.020	0.011	0.091	0.022

Standard errors in parentheses
* $p < 0.1$, ** $p < 0.05$, *** $p < 0.01$

Average of the coefficients: 0.111 Bootstrapped standard error: 0.061
Coefficients with the predicted sign: 10/10

10. Primary analyses including age at time of economic survey as additional control variable

We test the robustness of our primary analyses to including age at the time of the economic survey as an additional control variable.
Measure of self-regulation is RNSRI: index that includes RND and RNCCQ at age 17, 27, and 37 all equally weighted.
OLS Regressions: coefficients, standard errors, and nominal *p*-value

	(1) Net worth	(2) Perm income	(3) Wealth income ratio	(4) High interest-rate debt (reverse)	(5) Credit card misuse (reverse)	(6) Delay choice	(7) Savings rate	(8) Financial health	(9) Education years	(10) Forward looking behaviors	(11) Social status
RNSRI	0.305***	0.326***	0.084	0.091	0.173**	0.160*	0.000	0.242***	0.247***	0.351***	0.142
	(0.096)	(0.092)	(0.099)	(0.083)	(0.076)	(0.087)	(0.095)	(0.091)	(0.085)	(0.087)	(0.089)
Male	0.000	-0.059	0.055	0.078	0.036	-0.054	0.236	0.155	-0.038	-0.591***	0.317*
	(0.196)	(0.190)	(0.203)	(0.171)	(0.158)	(0.179)	(0.184)	(0.189)	(0.177)	(0.180)	(0.183)
Current age (years)	0.012	-0.058	0.056	0.048	0.046	-0.097**	-0.034	-0.055	-0.075*	-0.012	-0.003
	(0.047)	(0.045)	(0.048)	(0.041)	(0.037)	(0.042)	(0.044)	(0.045)	(0.042)	(0.042)	(0.043)
Perm income							0.325***				
							(0.101)				
Constant	-0.533	2.710	-2.589	-2.314	-2.205	4.466**	1.523	2.462	3.488*	0.791	0.051
	(2.154)	(2.072)	(2.229)	(1.876)	(1.721)	(1.953)	(2.032)	(2.057)	(1.925)	(1.958)	(1.997)
N	106	109	106	106	110	109	103	110	110	110	110
R^2	0.091	0.120	0.021	0.027	0.060	0.076	0.134	0.078	0.098	0.208	0.049

Standard errors in parentheses
* $p < 0.1$, ** $p < 0.05$, *** $p < 0.01$

Average of the coefficients: 0.193
Coefficients with the predicted sign: 10/11
Measure of self-regulation is RND: Expected deviation from predicted wait time in uncensored log seconds (measured in a delay of gratification study at Bing preschool)

OLS Regressions: coefficients, standard errors, and nominal *p*-value

	(1) Net worth	(2) Perm income	(3) Wealth income ratio	(4) High interest-rate debt (reverse)	(5) Credit card misuse (reverse)	(6) Delay choice	(7) Savings rate	(8) Financial health	(9) Education years	(10) Forward looking behaviors	(11) Social status
RND	0.090	-0.067	0.145	-0.014	0.036	0.105	-0.052	-0.061	0.151^{*}	0.094	-0.136
	(0.099)	(0.097)	(0.097)	(0.083)	(0.078)	(0.087)	(0.089)	(0.094)	(0.087)	(0.092)	(0.088)
Male	-0.022	-0.046	0.014	0.103	0.065	-0.133	0.276	0.189	-0.062	-0.623^{***}	0.308^{*}
	(0.200)	(0.197)	(0.197)	(0.168)	(0.159)	(0.178)	(0.180)	(0.192)	(0.177)	(0.188)	(0.181)
Current age (years)	0.009	-0.055	0.050	0.046	0.037	-0.090^{**}	-0.041	-0.055	-0.085^{**}	-0.013	0.013
	(0.048)	(0.047)	(0.047)	(0.040)	(0.038)	(0.042)	(0.043)	(0.046)	(0.042)	(0.045)	(0.043)
Perm income							0.330^{***}				
							(0.092)				
Constant	-0.406	2.545	-2.303	-2.241	-1.777	4.167^{**}	1.816	2.464	3.933^{**}	0.821	-0.697
	(2.194)	(2.153)	(2.166)	(1.844)	(1.738)	(1.947)	(1.997)	(2.105)	(1.944)	(2.063)	(1.980)
N	109	112	109	109	113	112	106	113	113	113	113
R^2	0.009	0.019	0.034	0.016	0.013	0.052	0.150	0.026	0.056	0.095	0.043

Standard errors in parentheses
* $p < 0.1$, ** $p < 0.05$, *** $p < 0.01$

Average of the coefficients: 0.026
Coefficients with the predicted sign: 6/11

11. Relationship between an aggregate index of the primary capital formation variables and measures of self-regulation

We construct the capital formation index by averaging the 11 primary capital formation variables for the 102 subjects with no missing outcome variables and then rank-normalizing this average. In the first table, we regress the capital formation index on RND and RNSRI. In columns 1 and 2, we use the full econ sample with no missing outcome variables. In columns 3 and 4, we regress the capital formation index on RND in the diagnostic and non-diagnostic subsamples, separately. In the second table, we break up the RNSRI, regressing the capital formation index on RND and the RNCCQ broken down first by age and then by subscale.

OLS Regressions: coefficients, standard errors, and nominal *p*-value

	(1) Capital Formation Index	(2) Capital Formation Index	(3) Capital Formation Index (Diagnostic Subsample)	(4) Capital Formation Index (Non-Diagnostic Subsample)
RND	0.130		0.350**	-0.008
	(0.100)		(0.164)	(0.130)
RNSRI		0.430***		
		(0.091)		
Male	-0.005	0.017	-0.253	0.085
	(0.202)	(0.188)	(0.395)	(0.235)
Constant	0.003	0.011	0.034	-0.046
	(0.129)	(0.120)	(0.263)	(0.150)
N	102	99	27	75
R^2	0.017	0.188	0.163	0.002

Standard errors in parentheses
* $p < 0.1$, ** $p < 0.05$, *** $p < 0.01$

OLS Regressions: coefficients, standard errors, and nominal *p*-value

	(1)<br>Capital Formation Index	(2)<br>Capital Formation Index
RND	0.059	0.078
	(0.097)	(0.097)
Age 17 RNCCQ (parent-rating)	0.101	
	(0.113)	
Age 27 RNCCQ (self-rating)	0.223	
	(0.155)	
Age 37 RNCCQ (self-rating)	0.135	
	(0.151)	
RNCCQ: Attention		0.083
		(0.123)
RNCCQ: Coping		0.059
		(0.118)
RNCCQ: Goal Pursuit		0.258^{**}
		(0.118)
RNCCQ: Concern for Others		-0.057
		(0.108)
RNCCQ: Delay Ability		0.167
		(0.114)
RNCCQ: General Cognitive Ability		0.092
		(0.099)
Male	0.011	0.042
	(0.202)	(0.204)
Constant	0.013	-0.006
	(0.125)	(0.124)
N	99	99
R^2	0.177	0.215

Standard errors in parentheses
* $p < 0.1$, ** $p < 0.05$, *** $p < 0.01$

12. Correlation matrix of all primary outcome variables and components of the RNSRI

See Excel file “Correlation Table.xlsx” for correlations between all primary outcome variables and components of the RNSRI in the survey subsample.

13. The effects of the normalizing transformations on the correlation between RND and Age 17 (1984) RNCCQ index

In prior publications, wait time was measured as the deviation in seconds from the condition mean wait time (referred to here as Delay Deviation). In this section, we examine the effects of the normalizing transformations on the correlation between RND and Age 17 RNCCQ index.

The top row in the table below, highlighted in yellow, shows the correlations between the delay measure used in previous studies (Delay Deviation) and 5 configurations of the CCQ Index. (For these calculations, the CCQ data obtained in 1984 were used. This includes 163 subjects for whom both RND and Age 17 CCQ are available.) The first two columns show the CCQ index of self-regulation calculated with no use of the INT as it would typically be done when forming a psychological scale score. Column 1 calculates the CCQ index using all the items on the scales with each item weighted equally in the total. In Column 2, we calculate the individual scales first (attention, goal pursuit, delay ability, etc.), and then calculate a mean across the scales. Hence in this calculation, each scale is weighted equally regardless of the number of items in it. The next two columns use these same approaches, employing the rank-normalization before calculating the index. In Column 3, individual items are rank-normalized prior to forming a mean across all items. In Column 4, individual items are rank-normalized prior to the calculation of scale scores. These scale scores are then rank-normalized before calculating a mean across the scale scores. In Column 5, the rank-normalization is applied to means calculated in for Column 4. The scores used to calculate the correlations in Column 5 thus represent the RNCCQ index (in this case for 1984) that is used in the present analysis. Accordingly, moving from left to right across columns demonstrates the impact of progressively employing the rank-normalization to formulate the RNCCQ indices.

The rows of the table represent different calculations of waiting time. Row 1 represents Delay Deviation, the measure of waiting used in all prior publications, calculated as deviation in seconds from the condition mean wait time.

Row 2 represents the deviation from predicted waiting times in log seconds prior to using the inverse normal transformation. The procedures and logic used to develop this measure are described in detail in Detailed variable definitions and summary statistics: self-regulation. This measure statistically controls for condition, age, sex, and censoring and is defined as:

Log predicted delay deviation = expectation of actual uncensored log wait time – empirical Bayes prediction of the uncensored log wait time.

The third row is the inverse normal transformation applied to the scores obtained in row 2. This is the RND measure that is currently being used in the manuscript. Moving down the rows then represents the impact on obtained correlations by first using the modified calculation of delay deviation (Row 2) used in the present paper, and then the application of the inverse normal transformation to that modified measure (Row 3).

Age 17 CCQ:	(1)<br>Mean of items	(2)<br>Mean of scales	(3)<br>Mean of RN Items	(4)<br>Mean of RN Scales	(5)<br>RNCCQ
Delay Deviation	0.240***	0.224***	0.227***	0.212***	0.202***
	[p=0.002]	[p=0.004]	[p=0.004]	[p=0.006]	[p=0.010]
Log predicted delay deviation	0.188**	0.162**	0.173**	0.151*	0.133*
	[p=0.016]	[p=0.039]	[p=0.027]	[p=0.054]	[p=0.091]
RND	0.184**	0.163**	0.172**	0.154**	0.140*
	[p=0.018]	[p=0.037]	[p=0.028]	[p=0.049]	[p=0.074]

p-values in brackets
$^{*}\ p < 0.1$, $^{**}\ p < 0.05$, $^{***}\ p < 0.01$

The attenuation of the correlations from the first to the second column is consistent with the possibility that the application of INT to each of the subscales before averaging them together to form an overall index potentially increases noise when the subscales contain unequal numbers of items. For example, the General Cognitive Ability subscale consists of only 3 items, while the Coping subscale consists of 8 items. Assuming that the amount of noise contained in each item is equal, the General Cognitive Ability scale contains a greater amount of noise compared to the Coping subscale, because the former is an average of only 3 items, while the latter is an average of 8 items.

In addition, in 1984, there was only one item, CCQ item #65, on the Delay subscale. The other two items in the Delay subscale were added in later years. The fact that there was only one item in this subscale in 1984 potentially makes the issue described above more consequential. In addition, this item contained some very confusing language, making it difficult to see if endorsing this item simply indicates a greater ability to delay gratification, or if endorsing it also implies excessive and unnecessary delay of gratification. In the late 1980s we recognized this problem and removed the confusing language from subsequent follow-ups. On its own, this may not be a serious issue because it involves only one of the many items that make up the overall CCQ Index, but the fact that INT was applied every step of the way (first at the item level, then at the subscale level), and the fact that in 1984 this was the only item in the Delay subscale, amplifies the potential problem.

14. Independent variable is RNCCQ – analysis resulted from editorial processes

OLS regressions for primary outcome variables: coefficients, standard errors, and nominal *p*-value

Note RNCCQ is aggregated over ages 17, 27, and 37.

	(1) Net worth	(2) Perm income	(3) Wealth income ratio	(4) High interest-rate debt (reverse)	(5) Credit card misuse (reverse)	(6) Delay choice	(7) Savings rate	(8) Financial health	(9) Education years	(10) Forward looking behaviors	(11) Social status
RNCCQ	0.304^{***}	0.404^{***}	0.026	0.081	0.154^{**}	0.129	-0.007	0.288^{***}	0.251^{***}	0.345^{***}	0.202^{**}
	(0.095)	(0.089)	(0.099)	(0.083)	(0.077)	(0.089)	(0.097)	(0.090)	(0.086)	(0.087)	(0.087)
Male	0.032	-0.03	0.066	0.093	0.05	-0.056	0.23	0.174	-0.026	-0.568^{***}	0.334^{*}
	(0.195)	(0.184)	(0.204)	(0.171)	(0.159)	(0.184)	(0.183)	(0.187)	(0.178)	(0.179)	(0.180)
Perm income							0.336^{***}				
							(0.10)				
Constant	0.001	0.023	-0.03	-0.099	-0.093	0.001	-0.06	-0.061	0.01	0.224^{**}	-0.113
	(0.120)	(0.112)	(0.125)	(0.105)	(0.096)	(0.111)	(0.114)	(0.112)	(0.107)	(0.108)	(0.109)
N	106	109	106	106	110	109	103	110	110	110	110
R^2	0.091	0.164	0.002	0.011	0.036	0.021	0.129	0.091	0.074	0.203	0.072

Standard errors in parentheses
* $p < 0.1$, ** $p < 0.05$, *** $p < 0.01$

	Average of coefficients (bootstrapped SE)	Coefficients with the predicted sign
RNCCQ	0.198 (0.04)	10/11

Independent variable: RNCCQ (aggregated over ages 17, 27, and 37)

OLS regressions for secondary outcome variables: coefficients, standard errors, and nominal *p*-value

	(1) Diet, exercise, and BMI	(2) Smoking and alcohol	(3) Preventative health and dental care	(4) Less procrast-ination	(5) Consider consequences	(6) Savings rate	(7) Verbal SAT	(8) Quant SAT	(9) Education scale	(10) Emotional state & relationship
RNCCQ	0.405***	0.029	0.118	0.231**	0.153**	0.130	0.289**	0.199	0.272***	0.103
	(0.088)	(0.091)	(0.090)	(0.093)	(0.077)	(0.092)	(0.141)	(0.136)	(0.081)	(0.096)
Male	-0.283	-0.465**	-0.656***	-0.153	0.042	0.249	-0.255	0.791**	0.023	0.244
	(0.182)	(0.189)	(0.186)	(0.192)	(0.159)	(0.192)	(0.364)	(0.352)	(0.167)	(0.198)
Constant	0.104	0.189*	0.251**	0.054	-0.064	-0.081	0.065	-0.270	-0.026	-0.083
	(0.109)	(0.114)	(0.112)	(0.116)	(0.096)	(0.120)	(0.207)	(0.200)	(0.100)	(0.119)
N	110	110	110	110	110	103	31	31	110	110
R^2	0.187	0.055	0.121	0.062	0.036	0.034	0.148	0.196	0.096	0.023

Standard errors in parentheses
* $p < 0.1$, ** $p < 0.05$, *** $p < 0.01$

	Average of coefficients	Coefficients with the predicted sign
RNCCQ	0.193	10/10

D. Robustness analyses

We test the robustness of our analyses to the following:

1. We replicate the primary analyses using RND (seconds) to test the sensitivity of our model to the assumption of log-normality in analysis of wait time
2. We replicate the primary analyses winsorizing RNSRI and RND at the 5th and 95th percentile
3. Instead of estimating a model with a linear effect of RND, we introduce a spline with knots at the 33.3^{rd} and 66.6^{th} percentiles
4. We test robustness of analyses with age 17, 27, and 37 RNCCQ indices to the imputation method used to populate missing values. We test two alternative methods:
    a. Averaging of available data years (with no imputation). In the RNSRI, we give the available RNCCQ indices ¾ weight and RND ¼ weight
    b. A multiple imputation approach (see Detailed variable definitions and summary statistics: self-regulation for detailed description)
5. In prior publications, wait time has been measured as the deviation in seconds from the condition mean wait time (referred to here as Delay Deviation). As a robustness check, we re-test the primary analysis using Delay Deviation instead of our measure of RND (rank-normalized expected deviation from predicted wait time in uncensored log seconds derived from the Tobit random effects model)
6. We test robustness of our primary findings to assumptions made during data cleaning by dropping respondents for whom we made assumptions and repeating relevant analyses

1. Assume normality (rather than log-normality) of wait times in marshmallow task. Measure of self-regulation is a modified version of RND: expected deviation from predicted wait time in uncensored seconds (rank-normalized).

We replicate the primary analyses using RND (seconds) to test the sensitivity of our model to the assumption of log-normality in analysis of wait time. RND (seconds) assumes normality of wait times instead of log normality and is calculated as the expected deviation from predicted wait time in uncensored log seconds. See Detailed variable definitions and summary statistics: self-regulation for more detailed description.

OLS Regressions: coefficients, standard errors, and nominal *p*-value

	(1) Net worth	(2) Perm income	(3) Wealth income ratio	(4) High interest-rate debt (reverse)	(5) Credit card misuse (reverse)	(6) Delay choice	(7) Savings rate	(8) Financial health	(9) Education years	(10) Forward looking behaviors	(11) Social status
RND (sec)	0.066	-0.094	0.148	0.027	0.072	0.037	-0.069	-0.072	0.119	0.127	-0.131
	(0.098)	(0.096)	(0.097)	(0.083)	(0.077)	(0.088)	(0.089)	(0.094)	(0.088)	(0.091)	(0.088)
Male	-0.016	-0.039	0.013	0.094	0.056	-0.118	0.277	0.194	-0.053	-0.629***	0.307*
	(0.200)	(0.197)	(0.198)	(0.168)	(0.158)	(0.181)	(0.180)	(0.193)	(0.180)	(0.187)	(0.180)
Perm income							0.340***				
							(0.091)				
Constant	0.009	0.013	0.000	-0.098	-0.092	0.010	-0.079	-0.077	0.036	0.234**	-0.121
	(0.124)	(0.120)	(0.122)	(0.104)	(0.096)	(0.111)	(0.113)	(0.117)	(0.110)	(0.113)	(0.109)
N	109	112	109	109	113	112	106	113	113	113	113
R^2	0.004	0.010	0.022	0.004	0.010	0.005	0.144	0.013	0.017	0.102	0.041

Standard errors in parentheses
* $p < 0.1$, ** $p < 0.05$, *** $p < 0.01$

Average of the coefficients: 0.021
Coefficients with the predicted sign: 7/11

2. Winsorizing RNSRI and RND variables at the 5th and 95th percentiles

Measure of self-regulation is RNSRI: index of behavior including measure of RND, but also including Age 17, 27, and 37 RNCCQ indices (winsorized at the 5th and 95th percentiles)

OLS Regressions: coefficients, standard errors, and nominal *p*-value

	(1) Net worth	(2) Perm income	(3) Wealth income ratio	(4) High interest-rate debt (reverse)	(5) Credit card misuse (reverse)	(6) Delay choice	(7) Savings rate	(8) Financial health	(9) Education years	(10) Forward looking behaviors	(11) Social status
RNSRI (winsorized)	0.278***	0.306***	0.081	0.069	0.169**	0.116	-0.013	0.223**	0.270***	0.311***	0.103
	(0.099)	(0.097)	(0.103)	(0.086)	(0.080)	(0.093)	(0.097)	(0.097)	(0.090)	(0.092)	(0.093)
Male	-0.026	-0.078	0.043	0.094	0.060	-0.117	0.266	0.168	-0.046	-0.623***	0.276
	(0.192)	(0.188)	(0.198)	(0.167)	(0.155)	(0.180)	(0.179)	(0.188)	(0.174)	(0.179)	(0.180)
Perm income							0.347***				
							(0.097)				
Constant	0.004	0.017	-0.018	-0.101	-0.101	0.004	-0.075	-0.077	0.022	0.218**	-0.114
	(0.119)	(0.115)	(0.123)	(0.103)	(0.095)	(0.110)	(0.113)	(0.115)	(0.106)	(0.109)	(0.110)
Observations	109	112	109	109	113	112	106	113	113	113	113
R^2	0.069	0.085	0.006	0.009	0.041	0.018	0.139	0.054	0.076	0.172	0.033

Standard errors in parentheses
* $p < 0.1$, ** $p < 0.05$, *** $p < 0.01$

Average of the coefficients: 0.174
Coefficients with the predicted sign: 10/11

Measure of self-regulation is RND: Expected deviation from predicted wait time in uncensored log seconds (winsorized at the 5th and 95th percentiles)

OLS Regressions: coefficients, standard errors, and nominal *p*-value

	(1) Net worth	(2) Perm income	(3) Wealth income ratio	(4) High interest-rate debt (reverse)	(5) Credit card misuse (reverse)	(6) Delay choice	(7) Savings rate	(8) Financial health	(9) Education years	(10) Forward looking behaviors	(11) Social status
RND (winsorized)	0.095	-0.091	0.167	-0.004	0.064	0.070	-0.063	-0.104	0.145	0.098	-0.174*
	(0.107)	(0.105)	(0.106)	(0.090)	(0.084)	(0.095)	(0.097)	(0.101)	(0.095)	(0.099)	(0.095)
Male	-0.018	-0.045	0.019	0.101	0.061	-0.120	0.273	0.194	-0.050	-0.618***	0.305*
	(0.199)	(0.197)	(0.197)	(0.168)	(0.158)	(0.181)	(0.179)	(0.192)	(0.179)	(0.187)	(0.179)
Perm income							0.341***				
							(0.091)				
Constant	0.010	0.016	-0.001	-0.102	-0.094	0.011	-0.078	-0.077	0.035	0.230**	-0.120
	(0.123)	(0.120)	(0.122)	(0.104)	(0.096)	(0.111)	(0.113)	(0.117)	(0.109)	(0.114)	(0.109)
N	109	112	109	109	113	112	106	113	113	113	113
R^2	0.007	0.008	0.024	0.003	0.007	0.008	0.142	0.017	0.021	0.094	0.051

Standard errors in parentheses

* $p < 0.1$, ** $p < 0.05$, *** $p < 0.01$

Average of the coefficients: 0.018

Coefficients with the predicted sign: 6/11

3. Include RND spline with knots at 33.3rd and 66.7th percentiles

Measure of self-regulation is <u>RND</u>: Expected deviation from predicted wait time in uncensored log seconds
OLS Regressions: coefficients, standard errors, and nominal *p*-value

	(1) Net worth	(2) Perm income	(3) Wealth income ratio	(4) High interest-rate debt (reverse)	(5) Credit card misuse (reverse)	(6) Delay choice	(7) Savings rate	(8) Financial health	(9) Education years	(10) Forward looking behaviors	(11) Social status
RND (spline1)	-0.223	-0.162	-0.297	-0.200	-0.660***	-0.091	-0.391	-0.126	0.171	-0.358	-0.111
	(0.275)	(0.278)	(0.273)	(0.236)	(0.214)	(0.257)	(0.262)	(0.270)	(0.256)	(0.260)	(0.252)
RND (spline2)	-0.006	-0.330	0.400	0.110	0.809***	-0.085	0.280	-0.506	0.296	0.137	-0.608*
	(0.365)	(0.368)	(0.364)	(0.314)	(0.284)	(0.340)	(0.346)	(0.357)	(0.338)	(0.344)	(0.334)
RND (spline3)	0.522*	0.278	0.355	0.067	-0.054	0.440*	-0.085	0.431	-0.074	0.491*	0.338
	(0.277)	(0.278)	(0.276)	(0.238)	(0.212)	(0.251)	(0.252)	(0.266)	(0.252)	(0.257)	(0.249)
Male	-0.019	-0.068	0.059	0.123	0.179	-0.134	0.336*	0.152	-0.040	-0.593***	0.257
	(0.202)	(0.203)	(0.201)	(0.174)	(0.156)	(0.186)	(0.187)	(0.196)	(0.186)	(0.189)	(0.184)
Perm income							0.326***				
							(0.093)				
Constant	-0.244	0.003	-0.460	-0.303	-0.909***	-0.103	-0.465	-0.018	0.031	-0.180	0.017
	(0.321)	(0.324)	(0.319)	(0.276)	(0.250)	(0.302)	(0.313)	(0.314)	(0.298)	(0.303)	(0.294)
N	109	112	109	109	113	112	106	113	113	113	113
R^2	0.048	0.025	0.058	0.012	0.107	0.038	0.158	0.050	0.028	0.145	0.077

Standard errors in parentheses
* $p < 0.1$, ** $p < 0.05$, *** $p < 0.01$

	Average of coefficients
Delay spline 1	-0.223
Delay spline 2	0.045
Delay spline 3	0.246

<u>Graphical Representation of Spline vs. Linear Effects</u>:

X-axis is RND. Y-axis is the linear combination of constant and RND effects (linear or spline) predicted at each RND level.

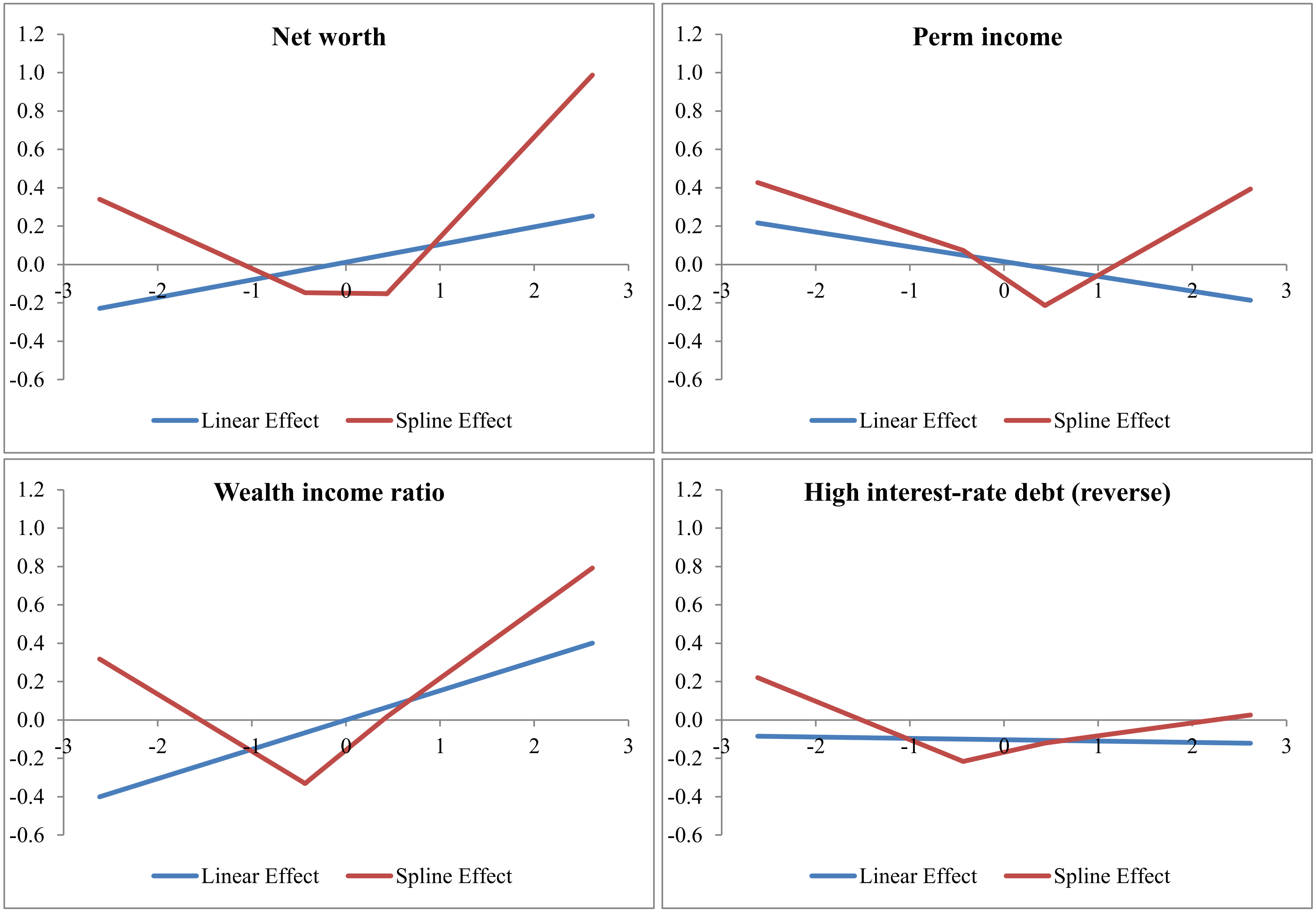

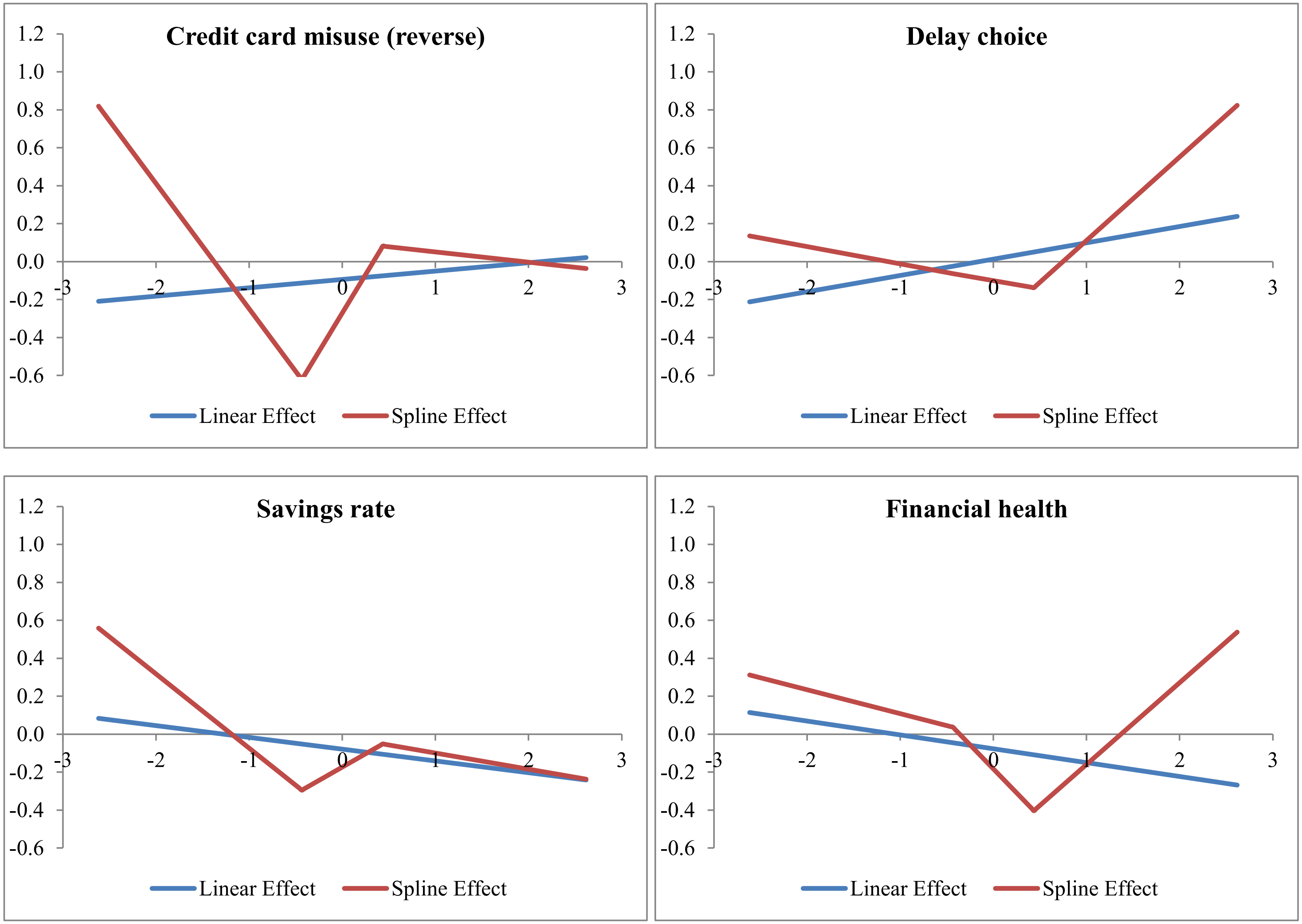
Credit card misuse (reverse)
1.2
1.0
0.8
0.6
0.4
0.2
0.0
-0.2
-0.4
-0.6
-3
-2
-1
0
1
2
3
Linear Effect
Spline Effect
Delay choice
Linear Effect
Spline Effect
Savings rate
Linear Effect
Spline Effect
Financial health
Linear Effect
Spline Effect

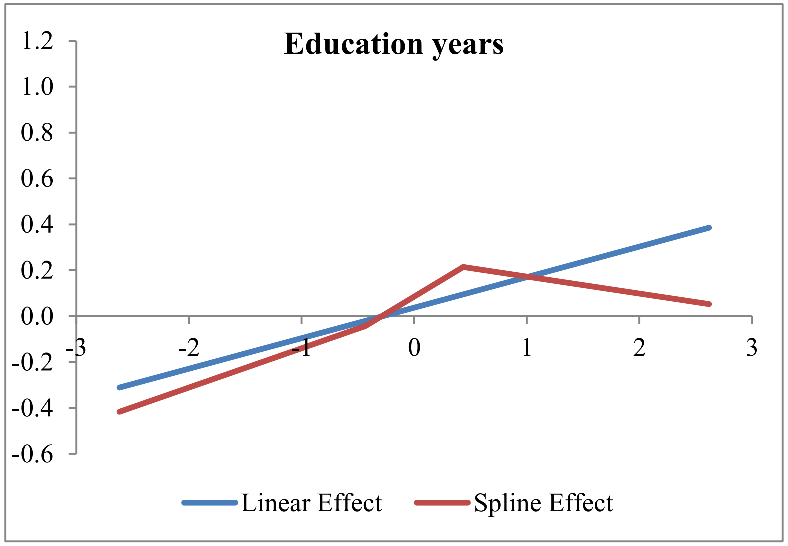
Education years
1.2
1.0
0.8
0.6
0.4
0.2
0.0
-0.2
-0.4
-0.6
-3
-2
-1
0
1
2
3
Linear Effect
Spline Effect

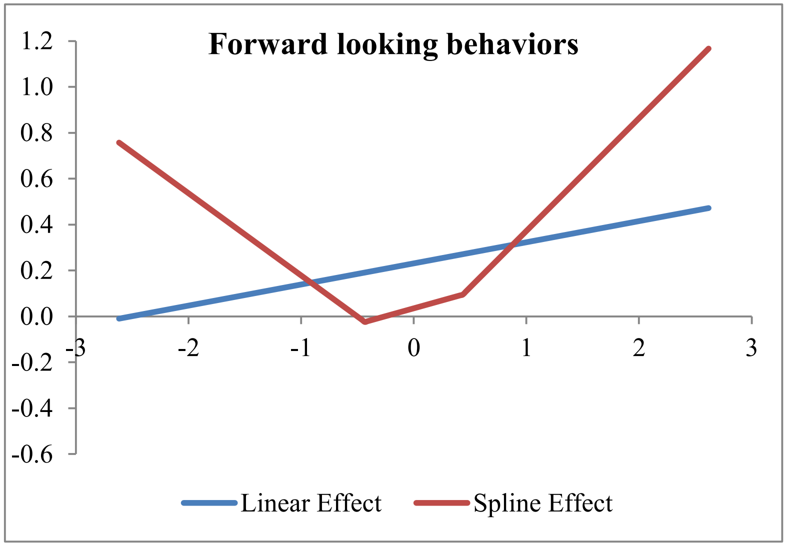
Forward looking behaviors
1.2
1.0
0.8
0.6
0.4
0.2
0.0
-0.2
-0.4
-0.6
-3
-2
-1
0
1
2
3
Linear Effect
Spline Effect

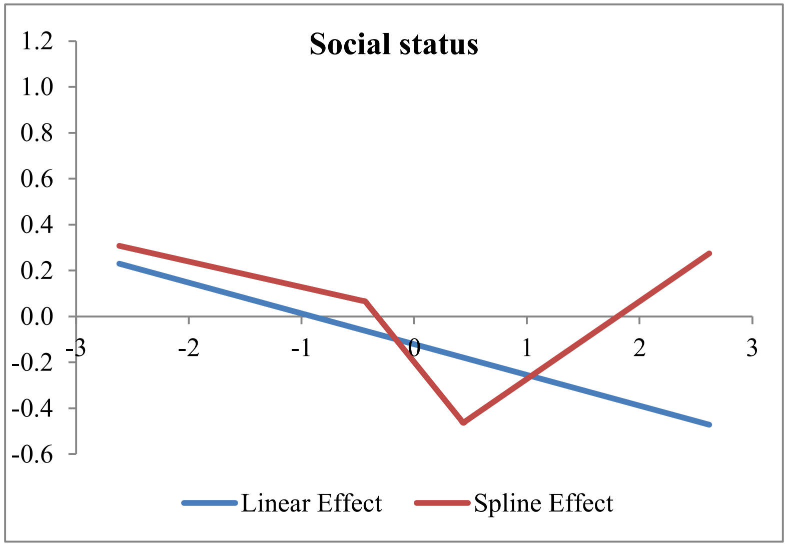
Social status
1.2
1.0
0.8
0.6
0.4
0.2
0.0
-0.2
-0.4
-0.6
-3
-2
-1
0
1
2
3
Linear Effect
Spline Effect

4. Robustness to imputation of RNCCQ indices

Average of available measures: to construct the RNSRI, we average non-missing RNCCQ indices (i.e., only include the available data years, with no imputation). In the RNSRI, we give the available RNCCQ indices ¾ weight and RND ¼ weight.

Measure of self-regulation is RNSRI (with no imputation): index of behavior including measure of RND, but also including age 17, 27, and 37 RNCCQ indices.

OLS Regressions: coefficients, standard errors, and nominal *p*-value

	(1) Net worth	(2) Perm income	(3) Wealth income ratio	(4) High interest-rate debt (reverse)	(5) Credit card misuse (reverse)	(6) Delay choice	(7) Savings rate	(8) Financial health	(9) Education years	(10) Forward looking behaviors	(11) Social status
RNSRI (avg avail.)	0.270***	0.238**	0.094	0.084	0.163**	0.147*	-0.060	0.170*	0.250***	0.299***	0.039
	(0.097)	(0.095)	(0.100)	(0.084)	(0.077)	(0.089)	(0.093)	(0.093)	(0.086)	(0.088)	(0.089)
Male	-0.011	-0.077	0.059	0.081	0.035	-0.068	0.229	0.143	-0.051	-0.603***	0.311*
	(0.197)	(0.195)	(0.203)	(0.171)	(0.159)	(0.183)	(0.183)	(0.192)	(0.178)	(0.182)	(0.184)
Perm income							0.352***				
							(0.097)				
Constant	0.023	0.043	-0.024	-0.092	-0.088	0.005	-0.058	-0.049	0.019	0.237**	-0.104
	(0.121)	(0.118)	(0.125)	(0.105)	(0.096)	(0.111)	(0.114)	(0.116)	(0.107)	(0.110)	(0.111)
N	106	109	106	106	110	109	103	110	110	110	110
R^2	0.069	0.058	0.009	0.012	0.041	0.027	0.132	0.035	0.074	0.175	0.027

Standard errors in parentheses
* $p < 0.1$, ** $p < 0.05$, *** $p < 0.01$

Average of the coefficients: 0.154
Coefficients with the predicted sign: 10/11

Multiple imputation (MI): we fill in missing RNCCQ indices using a multiple imputation approach.

See Detailed variable definitions and summary statistics: self-regulation for detailed description.

Measure of self-regulation is RNSRI (MI): index of behavior including measure of RND, but also including age 17, 27, and 37 RNCCQ indices

OLS Regressions: coefficients, standard errors, and nominal *p*-value

	(1) Net worth	(2) Perm income	(3) Wealth income ratio	(4) High interest-rate debt (reverse)	(5) Credit card misuse (reverse)	(6) Delay choice	(7) Savings rate	(8) Financial health	(9) Education years	(10) Forward looking behaviors	(11) Social status
RNSRI (MI)	0.236**	0.298***	0.008	0.089	0.161*	0.150	-0.060	0.177*	0.154	0.347***	0.065
	(0.108)	(0.104)	(0.113)	(0.092)	(0.084)	(0.096)	(0.106)	(0.104)	(0.101)	(0.096)	(0.103)
Male	0.003	-0.066	0.064	0.086	0.040	-0.065	0.228	0.149	-0.049	-0.590***	0.314*
	(0.200)	(0.194)	(0.204)	(0.171)	(0.160)	(0.184)	(0.183)	(0.193)	(0.183)	(0.182)	(0.184)
Perm income							0.354***				
							(0.100)				
Constant	0.014	0.038	-0.029	-0.095	-0.089	0.004	-0.058	-0.052	0.018	0.233**	-0.106
	(0.122)	(0.117)	(0.125)	(0.105)	(0.096)	(0.111)	(0.114)	(0.116)	(0.110)	(0.109)	(0.111)
Observations	106	109	106	106	110	109	103	110	110	110	110

Standard errors in parentheses
* $p < 0.1$, ** $p < 0.05$, *** $p < 0.01$

Average of the coefficients: 0.148
Coefficients with the predicted sign: 10/11

5. Original delay deviation measure (wait time in seconds minus condition mean wait times in seconds)

In prior publications, wait time was measured as the deviation in seconds from the condition mean wait time (referred to here as Delay Deviation). As a robustness check, we re-test the primary analysis using Delay Deviation instead of our measure of RND (expected deviation from predicted wait time in uncensored log seconds derived from the Tobit random effects model)

OLS Regressions: coefficients, standard errors, and nominal *p*-value (standardized coefficients shown on next page). Delay Deviation = deviation in seconds from condition mean divided by 100 so coefficients represent the predicted increase in the outcome given the subject waited 100s more

	(1) Net worth	(2) Perm income	(3) Wealth income ratio	(4) High interest-rate debt (reverse)	(5) Credit card misuse (reverse)	(6) Delay choice	(7) Savings rate	(8) Financial health	(9) Education years	(10) Forward looking behaviors	(11) Social status
Delay Deviation/100 (Orig)	0.014	-0.033	0.042	0.009	0.019	-0.003	-0.026	-0.033	0.042^{*}	0.039	-0.047^{*}
	(0.028)	(0.028)	(0.028)	(0.024)	(0.022)	(0.026)	(0.026)	(0.027)	(0.025)	(0.026)	(0.025)
Male	0.006	-0.076	0.066	0.103	0.081	-0.113	0.247	0.162	-0.006	-0.582^{***}	0.255
	(0.198)	(0.196)	(0.197)	(0.167)	(0.158)	(0.182)	(0.180)	(0.192)	(0.179)	(0.186)	(0.179)
Perm income							0.338^{***}				
							(0.091)				
Constant	-0.005	0.038	-0.033	-0.105	-0.107	0.010	-0.058	-0.055	0.005	0.204^{*}	-0.086
	(0.123)	(0.120)	(0.122)	(0.104)	(0.097)	(0.112)	(0.114)	(0.117)	(0.110)	(0.114)	(0.110)
N	109	112	109	109	113	112	106	113	113	113	113
R^2	0.002	0.014	0.022	0.005	0.008	0.004	0.147	0.021	0.025	0.105	0.052

Standard errors in parentheses

* $p < 0.1$, ** $p < 0.05$, *** $p < 0.01$

Average of the coefficients: 0.002

Coefficients with the predicted sign: 6/11

Original delay deviation measure (wait time in seconds – condition mean wait times in seconds) – all standardized variables (subtract mean and divide by SD)

OLS Regressions: standardized coefficients (except male) and standard errors, and nominal *p*-value

	(1) Net worth	(2) Perm income	(3) Wealth income ratio	(4) High interest-rate debt (reverse)	(5) Credit card misuse (reverse)	(6) Delay choice	(7) Savings rate	(8) Financial health	(9) Education years	(10) Forward looking behaviors	(11) Social status
Delay Deviation (Orig)	0.049	-0.115	0.146	0.035	0.080	-0.012	-0.092	-0.114	0.159*	0.136	-0.174*
	(0.098)	(0.096)	(0.097)	(0.098)	(0.095)	(0.096)	(0.093)	(0.095)	(0.094)	(0.091)	(0.093)
Male	0.006	-0.076	0.066	0.123	0.100	-0.123	0.258	0.164	-0.007	-0.583***	0.274
	(0.199)	(0.196)	(0.197)	(0.198)	(0.196)	(0.197)	(0.187)	(0.195)	(0.195)	(0.186)	(0.192)
Perm income							0.352***				
							(0.095)				
Constant	-0.000	0.027	-0.019	-0.046	-0.037	0.046	-0.081	-0.061	0.002	0.217*	-0.102
	(0.123)	(0.120)	(0.122)	(0.123)	(0.119)	(0.121)	(0.118)	(0.119)	(0.118)	(0.113)	(0.117)
Observations	109	112	109	109	113	112	106	113	113	113	113
R^2	0.002	0.014	0.022	0.005	0.008	0.004	0.147	0.021	0.025	0.105	0.052

Standard errors in parentheses
* $p < 0.1$, ** $p < 0.05$, *** $p < 0.01$

Average of the coefficients: 0.009
Coefficients with the predicted sign: 6/11

6. Drop subjects for whom we made assumptions

We test robustness of our primary findings to assumptions made during data cleaning by dropping respondents for whom we made assumptions and repeating relevant analyses. This analysis excludes:

- Subjects who were assumed to report income in thousands of dollars
- Subjects who were missing number of adults in the household for the permanent income calculation (assumed based on marriage status)
- Subjects who were assumed to report monthly debt
- Subjects for whom we made an assumption about the interest rate on their debt
- Subjects who did not report credit card debt (assumed 0)

Measure of self-regulation is RNSRI: index of behavior including measure of RND, but also including Age 17, 27, and 37 RNCCQ indices

OLS Regressions: coefficients, standard errors, and nominal *p*-value

	(1) Net worth	(2) Perm income	(3) Wealth income ratio	(4) High interest-rate debt (reverse)	(5) Credit card misuse (reverse)	(6) Savings rate
RNSRI	0.296***	0.320***	0.037	0.066	0.204**	-0.059
	(0.096)	(0.098)	(0.109)	(0.098)	(0.090)	(0.103)
Male	-0.016	-0.134	0.077	0.133	0.029	0.266
	(0.197)	(0.197)	(0.217)	(0.205)	(0.191)	(0.194)
Perm income						0.365***
						(0.108)
Constant	0.054	0.072	-0.066	-0.164	-0.161	-0.103
	(0.121)	(0.121)	(0.135)	(0.130)	(0.116)	(0.123)
Observations	103	98	93	77	84	93
R^2	0.087	0.104	0.003	0.011	0.059	0.132

Standard errors in parentheses
* $p < 0.1$, ** $p < 0.05$, *** $p < 0.01$

Measure of self-regulation is RND: Expected deviation from predicted wait time in uncensored log seconds (measured in the marshmallow task at Bing preschool)

OLS Regressions: coefficients, standard errors, and nominal *p*-value

	(1)<br>Net worth	(2)<br>Perm income	(3)<br>Wealth income ratio	(4)<br>High interest-rate debt (reverse)	(5)<br>Credit card misuse (reverse)	(6)<br>Savings rate
RND	0.089	-0.096	0.141	-0.022	0.041	-0.109
	(0.098)	(0.099)	(0.103)	(0.099)	(0.094)	(0.094)
Male	-0.055	-0.063	0.011	0.150	0.060	0.330*
	(0.201)	(0.204)	(0.212)	(0.198)	(0.190)	(0.191)
Perm income						0.347***
						(0.099)
Constant	0.054	0.008	-0.028	-0.171	-0.168	-0.131
	(0.124)	(0.126)	(0.133)	(0.127)	(0.117)	(0.121)
Observations	106	101	96	80	87	96
R^2	0.008	0.011	0.021	0.008	0.003	0.153

Standard errors in parentheses
* $p < 0.1$, ** $p < 0.05$, *** $p < 0.01$

VI. Survey Instrument

For the complete survey instrument, see the pdf "Bing Survey.pdf."

VII. Pre-registration Document

For the complete pre-registration document, see the pdf "Pre-registration Document.pdf."

VIII. References

1. Shoda Y, Mischel W, Peake PK (1990) Predicting adolescent cognitive and self-regulatory competencies from preschool delay of gratification: Identifying diagnostic conditions. *Dev Psychol* 26:978-986.
2. Mischel W, Shoda Y, Peake PK (1988) The nature of adolescent competencies predicted by preschool delay of gratification. *J Pers Soc Psychol* 54:687-696.
3. Ayduk Ö, *et al*. (2000) Regulating the interpersonal self: strategic self-regulation for coping with rejection sensitivity. *J Pers Soc Psychol* 79:776-792.
4. Ayduk Ö, *et al*. (2008) Rejection sensitivity and executive control: Joint predictors of borderline personality features. *J Res Pers* 42:151-168.
5. Schlam TR, Wilson NL, Shoda Y, Mischel W, Ayduk Ö (2013) Preschoolers' delay of gratification predicts their body mass 30 years later. *J Pediatr* 162:90-93.
6. Casey BJ, *et al*. (2011) Behavioral and neural correlates of delay of gratification 40 years later. *Proc Natl Acad Sci U S A* 108:14998-15003.
7. Moffitt TE, *et al*. (2011) A gradient of childhood self-control predicts health, wealth, and public safety. *Proc Natl Acad Sci U S A* 108:2693–2698.
8. Cognitive Economics Project. Cognition and Aging in the U.S.A. Decision Making Survey.
9. Block J, Block JH (1980) *The California Child Q-set* (Consulting Psychologists Press, Palo Alto, CA).
10. Richard F D, Bond Jr C, Stokes-Zoota J (2003) One Hundred Years of Social Psychology Quantitatively Described. *Rev Gen Psychol* 7:331–363.
11. Mischel W, Ebbesen EB (1970) Attention in delay of gratification. *J Pers Soc Psychol* 16:329-337.
12. Mischel W, Ebbesen EB, Zeiss AR (1972) Cognitive and attentional mechanisms in delay of gratification. *J Pers Soc Psychol* 21:204-218.
13. Rabe-Hesketh S, Skrondal A, Pickles A (2004) GLLAMM manual. *U.C. Berkeley Division of Biostatistics Working Paper Series* 160. Available at: http://biostats.bepress.com/ucbbiostat/paper160 [Accessed December 30, 2016].
14. Skrondal A, Rabe-Hesketh S (2004) *Generalized latent variable modeling: Multilevel, longitudinal, and structural equation model* (CRC Press, Boca Raton).
15. Von Hippel PT (2007) Regression with missing Ys: An improved strategy for analyzing multiply imputed data. *Sociol Methodol* 37:83-117.
16. Lee KJ, Carlin JB (2010) Multiple imputation for missing data: fully conditional specification versus multivariate normal imputation. *Am J Epidemiol* 171:624-632.
17. Little RJA, Rubin DB (2002) *Statistical Analysis with Missing Data* (Wiley, Hoboken, NJ). 2nd Ed.
18. Schafer JL, Olsen MK (1998) Multiple imputation for multivariate missing-data problems: A data analyst's perspective. *Multivar Behav Res* 33:545-571.
19. StataCorp (2013) *Stata 13 Multiple Imputation Manual* (Stata Press, College Station, TX).
20. Graham JW, Olchowski AE, Gilreath TD (2007) How many imputations are really needed? Some practical clarifications of multiple imputation theory. *Prev Sci* 8:206-213.
21. Rubin DB (1987) *Multiple Imputation for Nonresponse in Surveys* (Wiley, New York).

22. Ware Jr JE, Sherbourn CD (1992) The MOS 36-Item Short-Form Health Survey (SF-36): I. Conceptual framework and item selection. *Med Care* 30:473-483.
23. Benjamini Y, Hochberg Y (1995) Controlling the false discovery rate: A practical and powerful approach to multiple testing. *J R Stat Soc Series B* 57:289-300.
24. Efron B, Tibshirani R, Storey JD, Tusher V (2001) Empirical Bayes analysis of a microarray experiment. *J Amer Statist Assoc* 96:1151-1160.
25. Efron B (2010) *Large-Scale Inference: Empirical Bayes Methods for Estimation, Testing, and Prediction* (Cambridge University Press, New York).
