# Supplementary material for: Predicting mid-life capital formation with pre-school delay of gratification and life-course measures of self-regulation
Source: J Econ Behav Organ. Author manuscript; Available in PMC 2021 Jan 8. (PMC7792663; doi:10.1016/j.jebo.2019.08.016)
Supplement: 4 [file NIHMS1543524-supplement-4.pdf]

Bing Economic Analysis Plan

Contributors: Daniel J. Benjamin, David Laibson, Walter Mischel, Philip K. Peake, Yuichi Shoda, Alexandra Steiny, Nicole L. Wilson

Date Started: 8/2013
Last Updated: 7/8/2014

Contents

I. Format of the paper ... 3
II. Transformed variables ... 3
III. Primary analyses ... 3
  A. Primary outcome variables ... 4
  B. Primary right hand side variables ... 4
IV. Secondary analyses ... 4
  A. Secondary outcome variables ... 4
  B. Secondary sets of right hand side variables ... 5
  C. Secondary tests ... 5
V. Robustness checks ... 5
VI. Approach to multiple hypothesis testing ... 6
  A. Primary analyses ... 6
  B. Secondary analyses ... 6
  C. Priors for Bayesian analyses ... 7
VII. Ex-ante hypotheses and notes on analysis ... 7
VIII. Definitions ... 7
  A. Raw delay time ... 7
  B. Expected deviation from predicted delay time (uncensored log seconds and seconds) ... 8
  C. Self-control measured in 1984 (parent), 1993 (self), and 2003 (self) ... 8
  D. Self-regulatory competence: index of behavior to 2003 ... 10
  E. Age in months at time of original delay experiment ... 10
  F. Gender ... 11
  G. Net Worth ... 11
  H. Permanent Income ... 12
  I. Net worth / permanent income ... 12
  J. Annual amount of interest above 6% on high cost debt ... 13
  K. Percent of later choices in money sooner vs. later tradeoffs ... 13
  L. Measure of misuse of credit cards ... 14
  M. Savings Rate ... 14
  N. General measure of financial situation ... 15
  O. Educational attainment (years of education post-high school) ... 15
  P. Education Scale from Ayduk et al (2000) ... 15

Q. Quantitative and verbal SAT scores ... 16
R. Index of forward-looking behaviors ... 16
S. Social Status ... 18
T. Current emotional state and satisfaction with present relationship ... 19

IX. Analyses run prior to registering ... 20

X. Detailed Methodology ... 22
A. Treatment of delay time: Tobit random effects model ... 22
1. Brief Summary ... 22
2. Motivation and Overview ... 22
3. Data ... 24
4. Model ... 24
5. Calculation of the deviation from the predicted delay (key independent variable in our analyses) 26
B. Bayesian data analysis ... 27
C. Discussion of priors for Bayesian analysis ... 28
1. Mean of the correlation between outcome measures and self-control measures ... 28
2. Standard deviation of the correlation between outcome measures and self-control measures ... 29
D. False discovery rate control ... 29
E. Multiple imputation of self-control scores for robustness checks ... 31
1. More formal description of our multiple imputation procedure ... 32
2. Methodology after generating the imputed datasets ... 33

XI. References ... 34

I. Format of the paper

- There will be four sets of analyses reported in the paper and appendix:
  - Primary analyses run on the full sample, identified ex-ante in this document
  - Secondary analyses are additional hypotheses (i.e., not fundamental) of the data identified ex-ante in this document
  - Ex-post analyses are exploratory analyses that we will determine ex-post, after data analysis
  - Robustness checks are additional tests to check the robustness of results to assumptions made in the main analyses
- In the main body of the paper, we will report all primary analyses identified ex-ante
- We will also have an "additional analyses" section in the paper or supplementary materials which will report select findings from secondary and ex-post analyses (identified as such)
- All secondary and ex-post analyses not reported in the additional analyses section of the main paper will be reported in the supplementary materials
- All primary and secondary analyses will be recorded in the Open Science Framework prior to conducting any analyses

II. Transformed variables

- All variables (with the exception of the binary indicator for male gender) are transformed to have mean zero, variance one, and a normal-distribution shape by:
  1. Calculating a percentile rank of the underlying variable
  2. Applying the inverse normal function to the percentile rank

III. Primary analyses

- We will test, in separate regressions, the relationship between each of 11 primary outcomes and 2 primary measures of self-regulatory and delay ability for a total of 22 primary analyses
- We will control for gender in all analyses (note that we do not control for age at the time of the delay task because, in the delay time analyses where age would be relevant, the variable we use—deviation from expected delay time—already takes age into account)
- Each regression will be of the following form

$$y = \alpha + \beta_1 \times x + \beta_2 \times male$$

  where y is one of the primary outcomes, x is one of the primary right hand side variables (deviation from predicted delay time or self-regulatory competence), *male* is a dummy for being male, and α is a constant. β_1 is the coefficient of interest
- In analyses with savings rate as the primary outcome measure, we will also include permanent income as an additional covariate

A. Primary outcome variables

- Net worth n=109
- Permanent income n=112
- Net worth / permanent income n=109
- Annual amount of interest above 6% on high cost debt n=109
- Percent of later choices in money sooner vs. later tradeoffs n=112
- Measure of misuse of credit cards n=113
- Savings rate with permanent income as a control on the RHS n=106
- General measure of financial situation n=113
- Educational attainment in years n=113
- Index of forward-looking behaviors n=113
- Social status n=113

B. Primary right hand side variables

- We will test the relationship between all primary outcomes and the following two key independent variables:
    - Delay ability: Expected deviation from predicted delay time in uncensored log seconds (measured in the marshmallow task at Bing preschool)
    - Self-regulatory competence: index of behavior including measure of delay ability, but also including self-control measures in 1984, 1993, and 2003

IV. Secondary analyses

A. Secondary outcome variables

- We will test each of the five components of the index of forward-looking behaviors variable as separate secondary left-hand-side variables. These include:
    - Diet, exercise, and BMI
    - Smoking and alcohol use
    - Preventative health and dental care
    - Agreement with statements about procrastination
    - Carefully consider future consequences of current financial decisions
- We will test savings rate as an outcome variable without controlling for permanent income as a secondary analysis of savings behavior
- Because of small sample size for these measures, we will test Quantitative and Verbal SAT scores as two secondary outcome variables
- We will test the education scale as described in Ayduk et al (2000) as an additional outcome variable
- We will test current emotional state and satisfaction with present relationship as an additional outcome variable. This is a secondary outcome as we consider it to be a noisy measure of emotional well-being and happiness in relationships that focuses on current emotional states and relationships rather than stable emotional and personality traits

B. Secondary sets of right hand side variables

- We will test the following sets of secondary RHS variables in combination with all primary and secondary outcome variables:
  - Delay ability measured in preschool (deviation from predicted delay in log seconds) and an aggregate self-control index
    - $y = \alpha + \beta_1 \times \textit{delay ability} + \beta_2 \times (\textit{self} - \textit{control index}) + \beta_3 \times \textit{male}$
  - Delay ability measured in preschool (deviation from predicted delay in log seconds) and separate self-control indices in each year (1984, 1993, and 2003)
    - $y = \alpha + \beta_1 \times \textit{delay ability} + \beta_2 \times (\textit{self} - \textit{control index in } 1984) + \beta_3 \times (\textit{self} - \textit{control index in } 1993) + \beta_4 \times (\textit{self} - \textit{control index in } 2003) + \beta_5 \times \textit{male}$
  - Delay ability measured in preschool (deviation from predicted delay in log seconds) and each of the six subscales of the self-control index (averaged across years)
    - $y = \alpha + \beta_1 \times \textit{delay ability} + \beta_2 \times \textit{attention subscale} + \beta_3 \times \textit{coping subscale} + \beta_4 \times \textit{goal pursuit subscale} + \beta_5 \times \textit{concern for others subscale} + \beta_6 \times \textit{delay subscale} + \beta_7 \times \textit{general cognitive ability subscale} \;\; \beta_8 \times \textit{male}$

C. Secondary tests

- Our primary analyses will be run on the full sample. Secondarily, we will run the primary analyses with the key independent variable of expected deviation from predicted delay time in uncensored log seconds for the diagnostic condition (rewards exposed/spontaneous ideation) separately from the non-diagnostic conditions (pooling conditions with suggested ideation and/or rewards covered). We hypothesize that we will see larger effects in the diagnostic condition than in the remaining conditions and will test this in two ways:
  - After orienting the coefficients such that higher values correspond to higher self-control, we will test this hypothesis by running an F-test to test whether the sum of coefficients in the diagnostic condition is significantly different from the sum of coefficients in the non-diagnostic conditions
  - We will also test the differential predictive power of delay time in the diagnostic condition by testing the following model on the full sample where *y* represents each of the primary outcome variables and *diagnostic* is a binary indicator for being in the diagnostic condition. β_3 will be the coefficient of interest in these secondary tests
    - $y = \alpha + \beta_1 \times \textit{delay ability} + \beta_2 \times \textit{diagnostic} + \beta_3 \times \textit{delay ability} \times \textit{diagnostic} + \beta_4 \times \textit{male}$

V. Robustness checks

- We will replicate the primary analyses using deviation from predicted delay time in uncensored seconds (rather than log seconds) to test the sensitivity of our model to the assumption of log-normality in analysis of delay time in the marshmallow task
- We will replicate the primary analyses winsorizing the key delay ability or self-regulatory competence variable at the 5th and 95th percentile
- We will replicate the primary analyses with the key independent variable of expected deviation from predicted delay time in uncensored log seconds. In these secondary tests,

instead of estimating a model with a linear effect of deviation in delay time, we will introduce a spline with knots at the 33.3rd and 66.6th percentiles of deviation in delay time

- We will test robustness of all analyses with self-control scores in the 1984, 1993, and 2003 surveys to the imputation method used to populate missing values. Robustness checks will include:
    - Averaging of available data years (with no imputation). In the self-regulatory competence variable, we will give the available self-control indices ¾ weight and delay ability ¼ weight. In secondary analyses of the self-control subscales, we will average across available years
    - A multiple imputation approach for analyses with the self-regulatory competence variable (see Section X for detailed methodology)
- In past analyses, delay time in the marshmallow task has been measured as the deviation in seconds from the condition mean delay time. As a robustness check, we will re-test the primary analysis using deviation in seconds from the condition mean delay time instead of expected deviation from predicted delay time in uncensored log seconds derived from the Tobit random effects model
- We will test robustness of our primary findings to assumptions made during data cleaning by dropping respondents for whom we made assumptions and repeating relevant analyses

VI. Approach to multiple hypothesis testing

A. Primary analyses

- To address the issue of multiple hypothesis testing, we will report the following for each primary test:
    1. Coefficient, standard error, and nominal *p*-value
    2. Indication of significance from an analysis controlling the false discovery rate at 0.1. This analysis will be done separately for each of the two primary RHS variables (delay ability and the self-regulatory competence index). (see Section X for detailed methodology)
    3. Bayesian "best guess" and SE for each coefficient given the priors listed in Section C below (see Section X for detailed methodology)
- We will also report the results of an enrichment analysis that tests joint significance of our primary hypotheses using a Wald test. We will evaluate significance using an empirical p-value calculated from a distribution of Wald statistics created by randomly resampling the key independent variable from the sample (with replacement), repeating all analyses, and re-running the Wald test. We evaluate significance using a 0.05 empirical p-value threshold. We will run separate empirical Wald tests for each of the two primary RHS variables (delay ability and the self-regulatory competence index)

B. Secondary analyses

- For analyses with secondary outcomes, we will report coefficients, standard errors, nominal *p*-values, and Bayesian "best guess" and SE for each coefficient given the priors listed in Section C below
- For secondary tests (i.e., comparison of diagnostic to non-diagnostic conditions) and secondary sets of independent variables, we will report coefficients, standard errors, and nominal *p*-values

C. Priors for Bayesian analyses

- We will set our priors for Bayesian analysis based on the key independent variable in the primary analyses:
    - Correlations with delay ability (deviation in delay time measured in the marshmallow task): mean of prior distribution = 0.05
    - Correlations with multi-year self-regulatory competence index: mean of prior distribution = 0.15
- We assume a standard deviation of the prior correlation of 0.2 for all analyses
- See <u>Section X</u> for detailed methodology and discussion of priors

VII. Ex-ante hypotheses and notes on analysis

- We hypothesize that behavior in the marshmallow delay task will not have much explanatory power on mid-life outcomes. Performance on the task is a noisy measure of self-control and the effect is diluted as subjects' life trajectories have likely changed in the 30-40 years since the marshmallow task. We hypothesize that we will not find statistically significant results in our primary analyses predicting mid-life outcomes from behavior in the marshmallow delay task
- We hypothesize that correlations with indices of self-control behavior over the first 40 years of life will have more predictive power than performance on the marshmallow task alone. These correlations are likely to be higher because: (1) self-control is measured as recently as late 30s, much closer to the mid-life outcomes than the marshmallow task, (2) these measures have been successful in earlier work predicting life outcomes, and (3) index scores tend to be more successful than single measures of individual differences since they average out more noise
- We expect that the self-regulatory competence index will have low correlation with the outcome variables
- In this wave of data collection, we have responses from 113 of the original 550 Bing participants. We recognize that attrition and media attention to previous results from this sample are important issues; however, we make no assumptions of how these issues may impact results
- The focus of this paper is limited to the relationships and variables outlined in this document. Future analyses may test hypotheses using additional variables from the same wave of data collection. The analyses we plan to do in this project will be restricted to predictions of the outcomes measured using the survey this team conducted. The approach to handling multiple testing problems outlined in this analysis plan is designed specifically for this set of analyses will not affect future analyses addressing different questions, using variables based on other waves, past and future, of data collection from the Bing sample

VIII. Definitions

A. Raw delay time

- Raw delay time (measured in seconds) taken from the subject's first delay-of-gratification experiment
- Delay time ranges from 1 second to 900 seconds (the maximum possible delay time for the study design)
- Delay times are categorized into 21 conditions to account for differences in study designs across subjects (e.g., spontaneous vs. suggested ideation, rewards exposed vs. obscured, type of rewards, type of ideation, etc.)

B. Expected deviation from predicted delay time (uncensored log seconds and seconds)

- We believe raw delay times differ meaningfully across treatment conditions, by age, and by gender and therefore calculate each subject's expected deviation from the delay time predicted by a Tobit random effects model conditional on their age, gender, treatment condition, and accounting for the censoring of delay times at 900 seconds
- See Section X for detailed methodology

C. Self-control measured in 1984 (parent), 1993 (self), and 2003 (self)

- Reports of self-control were assessed at three time points using a modified versions of the California Child Q-set (Block & Block, 1969) that was completed by participants' parents with respect to their children's self-control ability in 1984, and by the participants' themselves in 1993, and then again in 2003
    - Note on self-control scores in 1984: There were two follow up surveys, one in 1982 and one in 1984. At each follow-up, both parents (i.e., mother and father) were asked to complete the CCQ. So, CCQ scores from 1984 are actually based on between 1 (from 1 parent at one time point) and 4 (from 2 parents at 2 time points) responses. At each time point, the responses were first standardized within each rater (i.e., mother and/or father), and then all responses (again, between 1 and 4 total) were averaged
    - 1993 and 2003 CCQ data were standardized within participants prior to computing self-control indices
- The self-control index is made up of 31 CCQ items split into 6 subscales (attention, coping, goal pursuit, concern for others, delay, and general cognitive ability). The final index variable was created using expert ratings, responses from 191 Smith college students, and the Bing data in 84, 93, and 03
- A preliminary set of self-control CCQ items was selected based on three expert ratings of each item in the full q-set for its relevance to aspects of self-control. The 37 items deemed relevant were administered to an independent sample of Smith College students. A principal components analysis revealed that all but one of these items loaded positively on the first unrotated factor. The remaining 36 items were examined using a confirmatory principal component analysis using ratings obtained in 1984, 1993, and 2003. Five items were eliminated from the analysis due to failure to demonstrate consistent positive loadings on the first unrotated principal component. From the remaining 31 items, two subscales consisting of 3 items each were formed on face value for the items relation to delay of gratification and general cognitive ability. The remaining 25 items were then factor analyzed within the Smith sample using principal axis factoring and oblique rotation noting that we fully expect positive correlations among the subscale. The four oblique factors resulting from this analysis were preliminarily attention, coping, goal pursuit, and concern for others. The item content of these scales as well as the delay and general cognitive ability subscale are listed below:
- Attention Subscale
    - *Is attentive and able to concentrate*
    - *Is planful, thinks ahead*
    - *Is restless and fidgety* (lower score corresponds to higher self-control; reverse scored)
    - *Is easily distracted* (lower score corresponds to higher self-control; reverse scored)
    - *Is reflective; thinks and deliberates before speaking or acting*
    - *Uses and responds to reason*
- Coping Subscale
    - *Can recuperate or recover after stressful experiences*

    - *Tends to withdraw and disengage when under stress* (lower score corresponds to higher self-control; reverse scored)
    - *Tends to go to pieces under stress; becomes rattles and disorganized*
    - *Overreacts to minor frustrations; is easily irritated and/or angered* (lower score corresponds to higher self-control; reverse scored)
    - *Reverts to more immature behavior when under stress* (lower score corresponds to higher self-control; reverse scored)
    - *Tends to become rigidly repetitive or immobilized under stress* (lower score corresponds to higher self-control; reverse scored)
    - *Exhibits self-control when frustrated*
    - *Tends to get sidetracked by minor setbacks or obstacles* (lower score corresponds to higher self-control; reverse scored)
- Goal Pursuit Subscale
    - *Is persistent in activities; does not give up easily*
    - *Is competent, skillful*
    - *Has high standards of performance for self*
    - *Is resourceful in initiating activities*
    - *Becomes strongly involved in the things I do*
    - *Is productive, gets things done*
- Concern for Others Subscale
    - *Shows concern for moral issues (e.g., reciprocity, fairness, and the welfare of others)*
    - *Can be trusted, is dependable*
    - *Is considerate and thoughtful of others*
    - *Is calm and relaxed; easygoing*
    - *Is curious and exploring; eager to learn and open to new experiences*
- Delay Subscale
    - *Settles for smaller but immediately available outcomes rather than pursuing larger ones* (lower score corresponds to higher self-control; reverse scored)
    - *Frequently yields to temptation* (lower score corresponds to higher self-control; reverse scored)
    - *Is unable to delay gratification; cannot wait for satisfactions* (lower score corresponds to higher self-control; reverse scored)
- General Cognitive Ability Subscale
    - *Genuinely values intellectual and cognitive matters*
    - *Has high intellectual capability*
    - *Is verbally fluent; can express ideas well in language*
- The following items were not included in the 1984 follow-up and thus are excluded from the 1984 self-control index
    - *Is easily distracted*
    - *Exhibits self-control when frustrated*
    - *Tends to get sidetracked by minor setbacks or obstacles*
    - *Is productive, gets things done*
    - *Is considerate and thoughtful of others*
    - *Settles for smaller but immediately available outcomes rather than pursuing larger ones*
    - *Frequently yields to temptation*
    - *Genuinely values intellectual and cognitive matters*

- Note: “Tends to spend extra money rather than save or invest” was excluded from consideration for the index as it is too descriptive of the primary outcome variables
- The items are aggregated in the following way:
    - Each item is transformed using the inverse-normal transformation
    - Transformed items are averaged at the year-subscale level giving equal weight to each available item
    - The subscale score is then transformed using the inverse-normal transformation
    - For subjects with a self-control measure in at least one year, missing subscales are imputed from subscale data in available years. For example, to impute a 1984 attention score for someone who has a 1993 and 2003 attention score, we run a regression of 1984 scores on 1993 and 2003 scores and use the estimated coefficients to predict the 1984 attention score for the individual. Similarly, for someone who is missing both 1984 and 1993 attention scores, we predict the 1984 attention score from a regression on 2003 attention scores
    - Imputed scores for each subscale are transformed using the inverse-normal transformation
    - Subscales are then averaged within each year (giving equal weight to each subscale) to obtain aggregate self-control indices in 1984, 1993, and 2003
- For the secondary analyses, we create two aggregate measures:
    - An aggregate self-control index across all three years is created by applying the inverse normal transformation to each year’s self-control index and averaging across the three years, giving each year equal weight
    - We also construct aggregate subscales of the self-control index across all three years by averaging the three transformed subscales, giving each year equal weight (e.g., to create the attention subscale, we average across the 1984, 1993 and 2003 transformed attention subscales)

D. Self-regulatory competence: index of behavior to 2003

- We will create a measure of self-regulatory competence as an index of behavior that includes the expected deviation from predicted delay time (in uncensored log seconds) and self-control indices in 1984, 1993, and 2003. The index will be an average of each of the four measures (transformed by the inverse normal transformation), giving equal weight to each measure. The final index will be re-transformed using the inverse normal percentile transformation to obtain a measure of persistent self-regulatory competence
    - Expected deviation from predicted delay time in uncensored log seconds
    - Self-control index in 1984
    - Self-control index in 1993
    - Self-control index in 2003
- This aggregate measure will only be calculated for subjects with at least one self-control score

E. Age in months at time of original delay experiment

- Subject’s age in months when first completed the marshmallow delay experiment

F. Gender

- Binary variable for whether the subject is male
  - *What is your sex? Male/Female*

G. Net Worth

- Net worth = sum of assets – sum of debt
- Assets
  - *What is the current balance or total value of financial assets held by your household in the following types of accounts: Checking accounts, Savings accounts, Money market accounts, Certificates of deposit (CDs), Tax-deferred retirement accounts (e.g., IRAs, 401(k) accounts, Keogh accounts), Other tax-deferred savings accounts (e.g., education savings accounts, health savings accounts), Bonds not included in tax-deferred accounts, Stocks not included in tax-deferred accounts, Other assets not listed above*
  - *Please indicate the <u>total market value</u> for each of the following types of assets. That is, what would they be worth if they were sold today? If you are not sure of the exact figures, please provide a rough estimate. Your primary residence, Additional residence(s) or vacation home(s), not including rental properties, Rental properties, Other real estate, Business(es) or farm(s), Vehicles (any cars, trucks, boats, trailers, motor homes, airplanes or other vehicles), Other assets such as trusts, commodities, valuable art, jewelry, coins, collectibles, or other items*
- Debt
  - *Please indicate the <u>amount you currently owe</u> for any of the following items. If you are not sure of the exact figures, please provide a rough estimate. Home mortgage for your primary residence, Second mortgage, home equity loans or lines of credit, or home improvement loans for your primary residence, Mortgage(s), home equity loans or lines of credit, or other loans for your secondary residence(s), Mortgage(s), home equity loans, lines of credit, or other loans for your rental property, Other real estate loans, Business or farm loans, Vehicle loans, Credit cards or other revolving accounts (only if you carry forward a balance; do not include balances that are paid off each month), Installment loans for major purchases (e.g., appliances or furniture), Educational loans, Other personal loans, Medical bills*
- Note: Three respondents were assumed to have filled in monthly mortgage payments rather than total amount owed. All three had high interest rates (6.5%) so we assumed they were unable to re-finance and therefore had <20% home equity. We assumed these respondents had 25 years left on a 30-year loan and were therefore paying almost all interest in their monthly payments. One respondent's implied debt (monthly payments * 12 / interest rate) was more than the value of the home, so we assumed 0% home equity. For the other two respondents, we assume 10% home equity (midpoint between 0 and 20%)
- Note: Two respondents were assumed to fill in monthly vehicle payments rather than total amount owed. We calculate the outstanding balance assuming the respondent is 3 years into a 6-year loan
- Note: One respondent was assumed to fill in monthly education loan payments rather than total amount owed. We calculate the outstanding balance assuming the respondent is 15 years into a 20-year loan

H. Permanent Income

- Permanent income is defined as the average of household income in the last calendar year and at age 35 divided by the number of adults living in the household:

$$\frac{\text{Average of household income in the last calendar year and at age 35 (scaled for inflation)}}{\text{Number of adults in the household}}$$

- We use the average of household income in the last calendar year and at age 35 (scaled for inflation) if both are available and non-zero. If either measure is missing or zero, we use only the available, non-zero income
    - Household income in last calendar year is the sum of household income from the following sources:
    - *In the last calendar year, what was your pre-tax household income from the following sources? Income from wages or salaries, Income from self-employment, Income from public and private pensions, Income from Social Security, Income from unemployment benefits, Income from any other social benefits, assistance, or grants (e.g., veteran's benefits, disability benefits), Income from investments, savings, insurance, or property (including dividends, interest, and rent from rental property), Income from any other sources*
- Stated total household income at 35 is scaled for inflation to the last calendar year using the CPI when the respondent was 35 and the CPI in the year prior to when the respondent completed the survey
    - *What was the total pre-tax annual income of your household when you were 35 years old? (If you don't know the exact figure, please give your best estimate.)*
- Number of adults currently living in the household
    - *Including yourself, how many people in each of the following age groups is currently living in your household? Adults*
    - Note: if this question was left blank, we assume two adults if married and one adult if never married or divorced

I. Net worth / permanent income

- Net worth divided by permanent income

J. Annual amount of interest above 6% on high cost debt

- Respondents selected an interest rate bucket for each type of debt. We assumed the interest rate is the midpoint of the selected category (i.e., if selected 5-8% bucket, the interest rate is assumed to be 6.5%)
- “High Cost Debt” is any debt for which the interest rate is greater than 6%
    - We chose the 6% interest rate threshold as an interest rate above which there is, for the most part, no good excuse to be paying that high an interest rate. We assume subjects with debt at an interest rate above 6% are either:
        1. Financially incompetent (e.g., have not refinanced their mortgage rates despite low interest rates in the last few years)
        2. In financial distress (e.g., have high leverage ratio on their collateralized debts or have bad credit)
- For each high cost debt category we calculate the amount of interest above 6% as the dollar amount of debt multiplied by the interest rate above 6%

$$\sum_{All\ high\ cost\ debt\ categories} (interest\ rate - 6\%) \times total\ amount\ owed$$

- *Please indicate the <u>amount you currently owe</u> for any of the following items. If you are not sure of the exact figures, please provide a rough estimate. Home mortgage for your primary residence, Second mortgage, home equity loans or lines of credit, or home improvement loans for your primary residence, Mortgage(s), home equity loans or lines of credit, or other loans for your secondary residence(s), Mortgage(s), home equity loans, lines of credit, or other loans for your rental property, Other real estate loans, Business or farm loans, Vehicle loans, Credit cards or other revolving accounts (only if you carry forward a balance; do not include balances that are paid off each month), Installment loans for major purchases (e.g., appliances or furniture), Educational loans, Other personal loans, Medical bills*
- *Please indicate the <u>interest rate</u> for each of those items. If you are not sure of the exact figures, please provide your best estimate.*

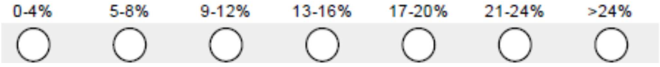

K. Percent of later choices in money sooner vs. later tradeoffs

- Percent of questions for which the respondent selected the “money later” instead of the “money sooner” option in the tradeoff questions
    - *Which would you prefer? (A) getting \$X today (B) getting \$Y thirty days from today*
    - *Which would you prefer? (A) getting \$X thirty days from today (B) getting \$Y sixty days from today*

L. Measure of misuse of credit cards

- The measure of misuse of credit cards is calculated as the average of the transformed responses (percentile inverse normal) to the following four questions about the subject's credit card usage. Percentile ranks for the sample are calculated after recoding the variables such that a higher score corresponds to higher misuse of credit cards.
    1. **Amount of carried credit card debt:** *Please indicate the amount you currently owe for any of the following items: Credit cards or other revolving accounts (only if you carry forward a balance; do not include balances that are paid off each month)*
    2. **Annual amount of interest above 6% on high cost credit card debt:** *Please indicate the amount you currently owe for any of the following items: Credit cards or other revolving accounts (only if you carry forward a balance; do not include balances that are paid off each month). Please indicate the <u>interest rate</u> for each of those items. If you are not sure of the exact figures, please provide your best estimate*
    3. **Binary measure of having been denied credit for a credit card in the last year:** *Have you been denied credit for any type of credit card within the last calendar year?*
    4. **Number of late payments in the last year:** *How many times have you made a late payment on any credit card bills in the last calendar year?*
- Note: For respondents who did not fill in a value for carried credit card debt, we assume $0 credit card debt if they reported non-zero debt in other debt categories in the same question and either report owing $0 on their credit cards each month or report paying off 100% of their credit card bill each month

M. Savings Rate

- Savings rate over the last few years as a percentage of income
- We will run regressions on savings rate with and without permanent income as a control:
    - Without controlling for permanent income, we will measure the effect of delay of gratification on savings rate (possibly through income)
    - Controlling for permanent income, we will measure whether delay of gratification impacts savings rate above and beyond the relationship through income
- Note: Reported savings rates of 70% and higher are suspect and excluded from analyses (n=4). These four respondents are believed to be out of the work force or second-earners in their household
- *On average each year, over the past few (e.g., 3-5) years, what percent of your income have you saved? Please include all forms of saving, including retirement accounts and other savings accounts.*

N. General measure of financial situation

- The general measure of financial situation is calculated as the average of the transformed responses (percentile inverse normal) to the following three questions about the subject's financial situation. Percentile ranks for the sample are calculated after recoding the variables such that a higher score corresponds to a better financial situation
    1. *In general, would you say you (and your family living with you) have more money than you need, just enough for your needs, or not enough to meet your needs? (more money = better financial situation)*
    2. *How difficult is it for you (and your family living with you) to pay your monthly bills? Very difficult – Not at all difficult (less difficult = better financial situation)*
    3. *Using a scale from 0 to 10 where 0 means "the worst possible financial situation" and 10 means "the best possible financial situation," how would you rate your financial situation these days? (higher rating = better financial situation)*

O. Educational attainment (years of education post-high school)

- We calculate the number of years of post-high school education based on reported degrees received. This is calculated as the sum of the base years of education and any additional years
- "Base years of education" is determined by the highest of the following degrees
    - Associate's Degree: 2 years post-high school
    - Bachelor's Degree: 4 years post-high school
    - Master's Degree: 6 years post-high school
    - PhD: 9 years post high-school
- "Additional years" are added for each of the following degrees
    - Law Degree: + 3 years
    - Medical Degree: + 4 years if highest degree is BA or MA, + 3 years if PhD
    - Second Master's degree: + 2 years
    - Teaching Credential: + 1 year
    - One year post-Master's program: + 1 year
- *For any degree(s) that you hold, please specify below the type of degree you have earned and specify the college, university, or institution that granted it*
- *What is the highest level of education you have attained? High school diploma or equivalent, Some college education, College degree, Some graduate or professional education, Graduate or professional degree*

P. Education Scale from Ayduk et al (2000)

- Highest educational level: high school = 1, correspondence courses = 2, college = 3, master's = 4, and PhD = 5
- We will try to find out exactly how categorization was done in the earlier paper (e.g., how law degrees were treated), but without any more information, we will classify medical degrees with doctorates, law degrees as between a Master's and PhD/Medical Degree (4.5), and 2-year degrees with correspondence courses

Q. Quantitative and verbal SAT scores

- Parent-reported quantitative and verbal SAT scores from the 1984 follow-up (subjects were about 18 years old)

R. Index of forward-looking behaviors

- Z-scores are calculated by taking inverse normal of the percentile ranks for each question after recoding the variables such that a higher score corresponds to better self-regulation. To create the composite measure, Z-scores are aggregated first by sub-section (e.g., diet, exercise), then by category (e.g., diet+ exercise + BMI), and finally across all five categories. Scores are renormalized (using the percentile transformation) at each step of the aggregation
- Note: each of the 5 subcategories below will be tested separately as secondary analyses
- Diet + Exercise + BMI
    - Diet
        - *In a typical week, how often do you choose your food (the type and/or amount) with health and fitness concerns in mind? (more meals = better regulated)*
        - *In a typical week, how often do you eat more than you think you should eat? (more meals = less regulated)*
        - *Please indicate the degree to which the following statements describe your general tendencies: I tend to eat healthfully. (more like me = better regulated)*
    - Exercise
        - *How many of those hours [that you are active] represent exercise primarily intended to improve or maintain your health or fitness? (more hours = better regulated)*
        - *Please indicate the degree to which the following statements describe your general tendencies: I exercise often. (more like me = better regulated)*
    - BMI calculated as (weight in pounds / (height in inches * height in inches)) x 703
        - *How tall are you? Approximately how much do you currently weigh? (higher BMI = less regulated)*
        - Note: percentile and inverse normal calculations for BMI are done separately for men and women (i.e., z-scores are normally distributed for men and women separately)
- Smoking and Alcohol
    - Smoking behavior: each subject's smoking behavior is ranked such that non-smokers have the lowest rank and smokers are ranked by number of packs per week. (higher rank smoking behavior = less regulated)
        - *Do you smoke (including cigarettes, cigars, pipes, or anything else)? (smoker = less regulated)*
        - *If you smoke cigarettes, about how many packs do you smoke per week? (more packs per week = less regulated)*
        - One smoker did not indicate packs per week and is assumed to smoke the average number of packs per week of other non-smokers. Two smokers indicated 0 packs per week
    - Alcohol consumption for people who drink alcohol. This measure excludes people who reported 0 or missing for all three measures (days per week when drinking, drinks per day, and max drinks in last month) as they may be recovering alcoholics, not drink for religious reasons, etc.

        - Number of drinks in a typical week calculated as days per week when drinking alcohol x typical number of drinks per day when drinking. (more drinks per week = less regulated)
            - *On average, how many days per week do you drink alcohol?*
            - *On a day when you drink, about how many alcohol drinks do you typically have?*
        - *In the past month, what is the maximum number of alcohol drinks you've had in one day (24 hours)? (more drinks = less regulated)*
- Preventative health and dental care
    - Preventative healthcare
        - *When your doctor gives you a prescription (excluding birth control), do you follow it exactly (for example, by taking all of the medication and taking it on the prescribed schedule)? (always adherent = more regulated)*
        - *How often do you visit your doctor for a routine check-up or preventive care? (more often = more regulated)*
    - Preventative dental care
        - *How often do you visit your dentist for a routine check-up or cleaning? (more often = more regulated)*
        - *How often do you floss your teeth? (more often = more regulated)*
- Agreement with statements about procrastination
    - *Please indicate the degree to which the following statements describe your general tendencies: (more like me = less regulated)*
        - *I needlessly delay finishing jobs, even though they are important.*
        - *I postpone starting in on things I don't like to do.*
        - *When I have a deadline, I wait until the last minute.*
        - *I delay making tough decisions.*
- Carefully consider future consequences of current financial decisions
    - *Please indicate the degree to which the following statements describe your general tendencies: I usually carefully consider the future consequences of my current financial decisions. (more like me = more regulated)*

S. Social Status

- Higher scores correspond to higher placement on the ladder of people in the US and transformed using the inverse normal percentile transformation
- *Think of this ladder as representing where people stand in the United States. At the top of the ladder are the people who are the best off - - those who have the most money, the most education and the most respected jobs. At the bottom are the people who are the worst off - - who have the least money, least education, and the least respected jobs or no job. The higher up you are on this ladder, the closer you are to the people at the very top; the lower you are, the closer you are to the people at the very bottom. Where would you place yourself on this ladder? Please select the number that corresponds to the rung on the ladder where you think you stand at this time in your life, relative to other people in the United States.*

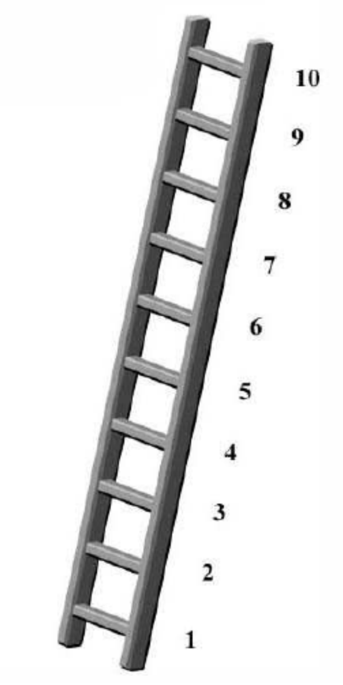

T. Current emotional state and satisfaction with present relationship

- This is a secondary outcome as we consider it a noisy measure of emotional well-being and happiness in relationships that focuses on current emotional states and relationships rather than stable emotional and personality traits
  - The MOS SF-36 is designed to assess patient outcomes after a procedure or treatment and focuses on emotions in the last 4 weeks
  - Rather than being able to measure happiness across all past relationships, the satisfaction with present relationship measure is dependent on the individual's current relationship and their perception of "the average couple"
- After creating the aggregate variables for each subsection listed below, Z-scores are calculated by taking the inverse normal of the percentile ranks for each category. To create the composite measure, Z-scores are averaged across all five categories and renormalized at the end
- <u>Role limitations due to emotional problems in the last 4 weeks</u>: Scored based on the Medical Outcomes Study 36-item short-form (MOS SF-36) where "Yes" = 0 and "No" = 100. The three scores are averaged to a composite score and then transformed using the percentile-rank inverse normal distribution.
  - *During the past 4 weeks, have you had any of the following problems with your work or other regular daily activities as a result of any emotional issues (such as feeling depressed or anxious)?*
    - *Cut down the amount of time you spent on work or other activities*
    - *Accomplished less than you would like*
    - *Were limited in the kind of work or other activities*
    - *Had difficulty performing the work or other activities (for example, it took extra effort)*
    - *Didn't do work or other activities as carefully as usual*
- <u>Health interfering with social activities in the last 4 weeks</u>: Scored based on the MOS SF-36 as described below. The two scores are averaged to a composite score and then transformed using the percentile-rank inverse normal distribution.
  - *During the past 4 weeks, to what extent has your physical health or emotional problems interfered with your normal social activities with family, friends, neighbors, or groups? (Not at all = 100, Slightly=75, Moderately=50, Quite a bit=25, Extremely=0)*
  - *During the past 4 weeks, how much of the time has your physical health or emotional problems interfered with your social activities with family, friends, neighbors, or groups? (All of the time=0, Most of the time=25, Some of the time=50, A little of the time=75, None of the time=100)*
- <u>Emotions felt in the last 4 weeks</u>: Scored based on the MOS SF-36 in increments of 20 points from 0 to 100 in the directions described below (e.g., more often = higher score). Scores are averaged to a composite score and then transformed with the inverse-normal transformation
  - *These questions are about how you feel and how things have been with you during the past 4 weeks. For each question, please give the answer that comes closest to the way you have been feeling. How much of the time during the past 4 weeks... (Responses: All of the time, Most of the time, A good bit of the time, Some of the time, A little of the time, None of the time)*
    - *Have you been a very nervous person? (more often = lower score)*
    - *Have you felt so down in the dumps that nothing could cheer you up? (more often = lower score)*
    - *Have you felt calm and peaceful? (more often = higher score)*
    - *Have you felt downhearted and blue? (more often = lower score)*
    - *Have you been a happy person? (more often = higher score)*

- Energy/fatigue in the last 4 weeks: Scored based on the MOS SF-36 in increments of 20 points from 0 to 100 in the direction described below (e.g., more often = higher score). The scores are averaged to a composite score and then transformed using the percentile-rank inverse normal distribution
    - *These questions are about how you feel and how things have been with you during the past 4 weeks. For each question, please give the answer that comes closest to the way you have been feeling. How much of the time during the past 4 weeks... (All of the time = lowest, None of the time = highest)*
        - *Did you feel full of pep? (more often = higher score)*
        - *Did you have a lot of energy? (more often = higher score)*
        - *Did you feel worn out? (more often = lower score)*
        - *Did you feel tired? (more often = lower score)*
- Satisfaction with present relationship: Z-scores are calculated by taking inverse normal of the percentile ranks for each of the following three questions after recoding the variables such that a higher score corresponds to happier in relationship. Scores are averaged and then re-normalized (using the percentile transformation) before including in the aggregate measure.
    - *All things considered, compared to the average couple, how happy are you in your present relationship?*
    - *Do you ever wish that you had not married, committed to, or moved in with your partner?*
    - Binary variable equal to 1 if selected "marry or commit to the same person"
        - *If you had your life to live over again, which of these would you most likely do? Marry or commit to the same person, Marry or commit to a different person, Not marry or commit to anyone at all*
    - Note: Individuals who did not answer the relationship questions (e.g., people who are divorced or not in a relationship) are missing this component of the index

IX. Analyses run prior to registering

- We ran the Tobit random effects model to obtain individual deviation from predicted delay time (in uncensored seconds and log seconds) conditional on treatment condition, age, and gender
    - We looked at models that varied the covariates (none, a quadratic effect of age) and at the non-Tobit random effects model
- Comparison of raw delay times and deviation from condition mean delay time by gender
- Distribution of genders across conditions
- Correlations of raw delay times and age at delay task
- For each of the dependent variables, we cleaned the data and looked at summary statistics of the raw variable and the transformed variable if relevant (summary statistics include: mean, min, max, median, standard deviation, count of non-missing, and histograms). We also looked at the summary statistics for the individual components of the misuse of credit card variable and the aggregate measure of high self-regulation
- In constructing the permanent income measure, we looked at the correlations between current reported total income, aggregated current income from different sources, and reported total income at age 35. We looked at total assets and debts for responses that were suspect (i.e., respondents who were inconsistent in reported income and outliers in all three income measures) to assess validity of reported values
- In creating the net worth to permanent income ratio we looked at the relationship between net worth/permanent income and each of the individual measures to investigate whether outliers were driven by one measure or another

- We looked at the correlation between savings rate and permanent income to determine whether we wanted to include permanent income as a control variable in the analysis of savings rate
- We looked at family structure (i.e., marital status and number of children), gender, employment status, income, assets, and debts to assess the validity of high reported savings rates. We also looked at the distributions of reported savings rates by gender and among non-working respondents
- For outliers in net worth, total assets, or total debt, we looked at the relationship between family structure (i.e., marital status and number of children), age, income, assets, and debt variables to assess validity of reported values
- For subjects with non-zero credit card debt, we checked for consistency (within and between subjects) in responses to all questions about credit card use
- For individuals who were missing a value for credit card debt, we looked at income, assets, debts, and responses to other credit card questions to determine whether credit card debt was $0 or a true missing
- We compared the aggregated distribution of debt across categories (e.g., mortgages, business loans, credit card debt) to the distribution of annual interest above 6% owed on debt across categories to verify that our high cost debt variable emphasized categories we assume to be high cost debt (e.g., credit card debt) and underweights debt categories we assume to have lower interest rates (e.g., mortgage debt, education loans). We also looked at the distribution of interest rates across debt categories
- For educational attainment, we confirmed consistency between reported highest educational level attained and individual degrees received
- We compared BMI by gender
- We looked at the inverse hyperbolic sine transformation for all transformed variables but ultimately decided to use the inverse normal percentile rank transformation
- We plotted raw delay times (seconds) and deviation from condition mean delay time (seconds) against transformed deviation in delay time (uncensored log seconds)
- We imputed missing self-control scores in each of the three survey years (1984, 1993, and 2003) using two methods
    - A single regression imputation model, in which missing years are populated with the predicted value from a regression of the missing year on all available years
    - A multivariate normal multiple imputation model including all primary and secondary left and right hand side variables (see <u>Section X</u> for detailed methodology)
- We ran simulations of the Wald test and false discovery rate control analyses using data simulated under various assumptions of the true effect sizes and number of hypotheses tested
- Principal components analysis of self-control CCQ items in the Smith college sample and Bing 1984, 1993, and 2003 data

X. Detailed Methodology

A. Treatment of delay time: Tobit random effects model

1. Brief Summary

Using a random effects model, we take an empirical Bayes approach to estimating a predicted delay time for each subject controlling for his or her age, gender, and experimental condition. We treat the condition effect on delay time as a random effect drawn from a normal distribution. We include age and a male dummy as covariates in the model. The approach is Bayesian in that a prior distribution is used to inform the estimates of the condition effects (the prior matters most in conditions with few subjects). The approach is empirical in the sense that the prior distribution of random effects is estimated from the data. In a simple version of the method (not what we actually use), the estimated mean delay time for a condition is a weighted average of the sample mean of delay time and the estimated overall mean of delay times across all conditions. For conditions with larger N, the estimated mean for the condition will be more heavily weighted toward the observed sample mean. In the version of the method we actually use for primary analyses, we assume delay times are log-normally distributed, we use a Tobit random effects model to address the truncation of delay times at 900 seconds, and we control for age and gender in the model.

2. Motivation and Overview

We would like to compare subjects' delay times in the marshmallow experiment. We believe delay times differ meaningfully across some experimental conditions (i.e., individuals are expected to wait longer when rewards are covered rather than exposed), by age, and by gender and therefore would like to standardize individual performance in the delay of gratification task across conditions, ages, and gender.

To predict delay time for each individual, we include a linear effect of age and a male dummy in the regression model. Because we have a small *N* in some conditions, we use a random effects model to estimate an expected effect on delay time for each experimental condition, controlling for age and gender. We assume that the condition effects are draws from a distribution with an underlying mean and standard deviation. The random effects model predicts a condition effect that is a weighted average of the observed effect of the condition (conditional on age and gender) and the mean of all condition effects (assumed to be 0). Because we are treating the differences between conditions as random effects rather than fixed effects, we estimate only six parameters in the model: a constant term, the effect of being one month older, the effect of being male, the variance of the condition random effects, and the variance of the idiosyncratic error.

We use a Tobit random effects model to account for the fact that the structure of the original experiments imposed a maximum delay time of 900 seconds (or 15 min). The Tobit random effects model accounts for this censoring of delay times and generates estimates of underlying, uncensored delay times.

The distribution of all 543 raw delay times has many observations at a short delay time as well as a long right tail (truncated at 900 seconds). We therefore assume that individual delay times are log-normally distributed. The distribution of mean raw delay times in the 21 conditions also appears to have a long

right tail, so we also treat the underlying condition means as log-normally distributed. All primary analyses are run using the natural log of delay time in seconds.

For a set of secondary analyses, we will test sensitivity of the results to the assumption that delay times are log-normally distributed by repeating the Tobit random effects analysis for delay time in seconds (rather than log-seconds). This analysis provides us with individual deviations from predicted delay times in seconds (given condition, age, and gender), which we use as an alternative key independent variable.

Here is the distribution of all 543 raw delay times (seconds and log of seconds):

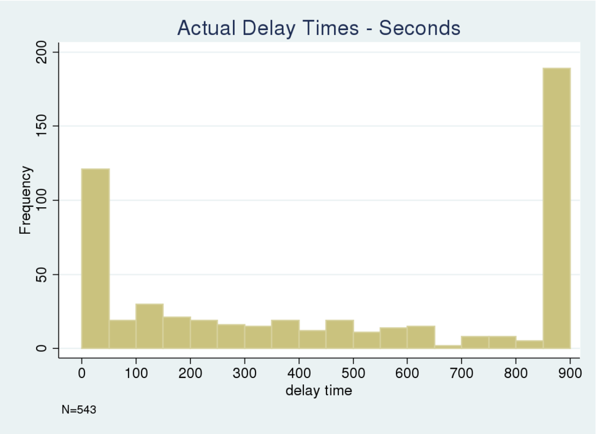

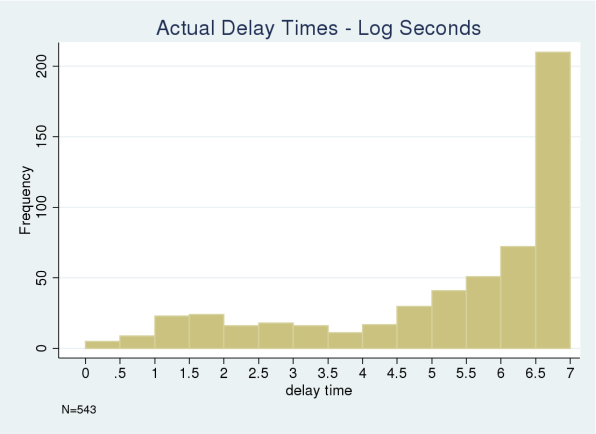

Here is the distribution of 21 Condition Means (mean seconds and log of mean seconds):

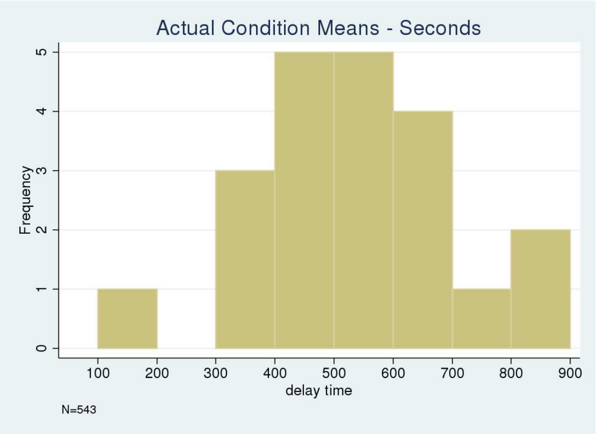

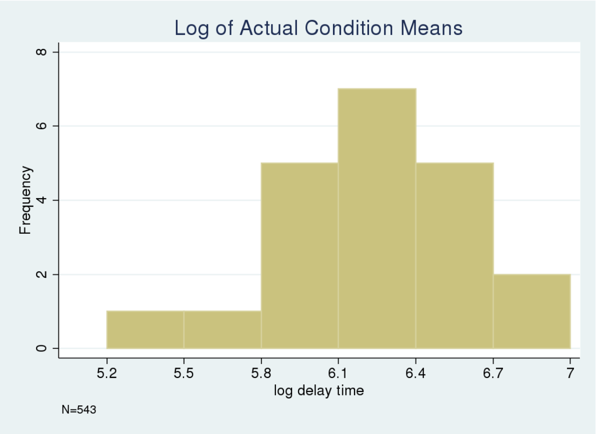

From the random effects model, we predict an expected delay time for each person in uncensored log seconds conditional on their age, gender, and experimental condition.

In a linear case (non-Tobit), the estimated effect on log delay time of being in a condition X can be thought of as a weighted average of 0 (i.e., no effect) and the difference between the sample mean log delay time in condition X and the mean log delay time across all conditions, controlling for age and gender. For conditions with larger *N*, the estimated condition effect will be more heavily weighted toward

the observed deviation from the underlying mean log delay time. The Tobit random effects model accounts for the truncation of delay and estimates the (latent) uncensored log delay time for each subject.

Finally, we calculate each subject's expected deviation from the delay time predicted by the regression model as ln(subject's actual delay time) – predicted ln(delay time). If the subject's delay time is at the boundary of 900 seconds, we calculate the expected value of the uncensored delay time (given that the subject would have waited at least 900 seconds) and use the estimated uncensored delay time in place of the subject's actual delay time at the boundary. This expected deviation from the predicted delay is the variable that will ultimately be used as an independent variable in our key analyses.

3. Data

There are 21 conditions (or groups) with a total of 543 subjects for which delay time, age, and gender data are available (N ranges from 3-165 per condition). The 2012 survey includes a subset of 113 of these subjects across 20 conditions (N ranges from 1-34 per condition).

Notes: One subject has a delay time of 0 seconds. We assume this is measurement error as some time must have elapsed before signaling for the return of the experimenter. We assign this subject to delay time of 1 second. There are 8 observations at exactly 600 seconds (5 of which are in the same condition). We believe these subjects to be true 600s observations.

4. Model

There are 21 groups with N_g observations in each group. The natural log of delay time for individual i in group g is y_{gi} and is censored above at ln(900 seconds). The underlying, uncensored, log delay time is y^*_{gi}.

Each individual's uncensored delay time is $y^*_{gi} = \alpha + \beta_1 \times age_{gi} + \beta_2 \times male_{gi} + \eta_g + \varepsilon_{gi}$, where age_{gi} is age in months at the delay task, $male_{gi}$ is a binary indicator of gender, η_g is the group random effect $\sim N(0, {\sigma_\eta}^2)$ and ε_{gi} is the idiosyncratic error $\sim N(0, {\sigma_\varepsilon}^2)$.

The likelihood for each observed delay time y_{gi} in group g consists of two parts:

$$L_{gi} = \begin{cases} \dfrac{1}{\sigma_\varepsilon}\phi\left(\dfrac{y_{gi} - \left(\alpha + \beta_1 \times age_{gi} + \beta_2 \times male_{gi} + \eta_g\right)}{\sigma_\varepsilon}\right) & \text{if uncensored} \\ 1 - \Phi\left(\dfrac{\ln(900) - \left(\alpha + \beta_1 \times age_{gi} + \beta_2 \times male_{gi} + \eta_g\right)}{\sigma_\varepsilon}\right) & \text{if censored} \end{cases}$$

or

$$L_{gi} = \begin{cases} \frac{1}{\sigma_\varepsilon}\phi\left(\frac{y_{gi} - \left(\alpha + \beta_1 \times\ age_{gi} + \beta_2 \times\ male_{gi} +\ \eta_g\right)}{\sigma_\varepsilon}\right) \text{ if uncensored} \\ \Phi\left(\frac{\left(\alpha + \beta_1 \times\ age_{gi} + \beta_2 \times\ male_{gi} +\ \eta_g\right) - \ln(900)}{\sigma_\varepsilon}\right) \text{ if censored} \end{cases}$$

where ϕ is the standard normal probability density function and Φ is the standard normal cumulative distribution function. So, the likelihood contribution for each individual is the probability density of the normal distribution for uncensored observations and the likelihood of being censored if the observed individual is at the boundary.

The likelihood for each group g, integrating over all random effects η_g, is

$$L_g = \int_{-\infty}^{\infty}\left(\prod_{i=1}^{N_g}\left[\Phi\left(\frac{\left(\alpha + \beta_1 \times\ age_{gi} + \beta_2 \times\ male_{gi} +\ \eta_g\right) - \ln(900)}{\sigma_\varepsilon}\right)\right]^{B_{gi}} * \left[\frac{1}{\sigma_\varepsilon}\phi\left(\frac{y_{gi} - \left(\alpha + \beta_1 \times\ age_{gi} + \beta_2 \times\ male_{gi} +\ \eta_g\right)}{\sigma_\varepsilon}\right)\right]^{1-B_{gi}}\right)\frac{1}{\sigma_\eta}\phi\left(\frac{\eta_g}{\sigma_\eta}\right)d\eta_g$$

where B_{gi} is an indicator for individual i being at the boundary ($y_{gi} = \ln(900)$).

The likelihood function is maximized with the Stata function GLLAMM using adaptive quadrature, a technique for approximating specific integrals with a weighted sum of function values at some specified points.

We obtain maximum likelihood estimates of α, the constant, β_1, the coefficient on age, β_2, the coefficient on the male dummy, ${\sigma_\eta}^2$, the variance of the condition random effects, and ${\sigma_\varepsilon}^2$, the idiosyncratic variance.

The condition effects on delay times (i.e., η_g) are not directly estimated in the random effects model. We use the Stata GLLAMM procedure to obtain an empirical Bayes prediction of the condition expected delay time (in log seconds). The empirical Bayes predictor is the expected value of the posterior distribution of random effects, $p\left(\eta_g | \mathbf{y}; \widehat{\boldsymbol{\theta}}\right)$ which treats the parameter estimates $\widehat{\boldsymbol{\theta}}$ (the vector of estimates for $\alpha, \beta_1, \beta_2, {\sigma_\eta}^2, {\sigma_\varepsilon}^2$) as known, in addition to the observed delay times $\mathbf{y}$.

If $p\left(\eta_g | \widehat{\boldsymbol{\theta}}\right)$ is the prior distribution of the random effects before "seeing" the data for group g and $p\left(y_{gi} | \eta_g, \widehat{\boldsymbol{\theta}}\right)$ is the likelihood contribution for each individual y_{gi} given $\eta_g, \widehat{\boldsymbol{\theta}}$, then the posterior distribution of the random effects is

$$p\left(\eta_g | \mathbf{y};\ \widehat{\boldsymbol{\theta}}\right) = \frac{p\left(\mathbf{y}, \eta_g | \widehat{\boldsymbol{\theta}}\right)}{p\left(\mathbf{y} | \widehat{\boldsymbol{\theta}}\right)} = \frac{p\left(\eta_g | \widehat{\boldsymbol{\theta}}\right)\prod_{i=1}^{N_g} p\left(y_{gi} | \eta_g, \widehat{\boldsymbol{\theta}}\right)}{\int_{-\infty}^{\infty} p\left(\eta_g | \widehat{\boldsymbol{\theta}}\right)\prod_{i=1}^{N_g} p\left(y_{gi} | \eta_g, \widehat{\boldsymbol{\theta}}\right) d\eta_g}$$

Then the empirical Bayes estimate of the random effect η_g is

$$\eta_g{}^{EB} = E[\eta_g|\,\mathbf{y};\,\widehat{\boldsymbol{\theta}}] = \frac{\int_{-\infty}^{\infty}\eta_g p(\eta_g|\widehat{\boldsymbol{\theta}})\prod_{i=1}^{N_g}p(y_{gi}|\eta_g,\widehat{\boldsymbol{\theta}})\,d\eta_g}{\int_{-\infty}^{\infty}p(\eta_g|\widehat{\boldsymbol{\theta}})\prod_{i=1}^{N_g}p(y_{gi}|\eta_g,\widehat{\boldsymbol{\theta}})\,d\eta_g}$$

For the specific case that we use—the Tobit random effects model—the empirical Bayes estimate for the random effect is

$$\eta_g{}^{EB} = \frac{\int_{-\infty}^{\infty}\eta_g\frac{1}{\sigma_\eta}\phi\left(\frac{\eta_g}{\sigma_\eta}\right)\left\{\prod_{i=1}^{N_g}\left[\Phi\left(\frac{\hat{y}_{gi}-\ln(900)}{\sigma_\varepsilon}\right)\right]^{B_{gi}}\left[\frac{1}{\sigma_\varepsilon}\phi\left(\frac{y_{gi}-\hat{y}_{gi}}{\sigma_\varepsilon}\right)\right]^{1-B_{gi}}\right\}d\eta_g}{\int_{-\infty}^{\infty}\frac{1}{\sigma_\eta}\phi\left(\frac{\eta_g}{\sigma_\eta}\right)\left\{\prod_{i=1}^{N_g}\left[\Phi\left(\frac{\hat{y}_{gi}-\ln(900)}{\sigma_\varepsilon}\right)\right]^{B_{gi}}\left[\frac{1}{\sigma_\varepsilon}\phi\left(\frac{y_{gi}-\hat{y}_{gi}}{\sigma_\varepsilon}\right)\right]^{1-B_{gi}}\right\}d\eta_g}$$

where $\hat{y}_{gi} = \alpha + \beta_1 \times age_{gi} + \beta_2 \times male_{gi} + \eta_g$

(and this formula is calculated using the estimates for $\alpha, \beta_1, \beta_2, \sigma_\eta{}^2, \sigma_\varepsilon{}^2$). The empirical Bayes estimates for the random effects are outputted automatically from the GLLAMM post-estimation procedure.

5. Calculation of the deviation from the predicted delay (key independent variable in our analyses)

Deviation from predicted delay = expectation of actual uncensored log delay time – empirical Bayes prediction of the uncensored log delay time

If not at the boundary, the expectation of actual uncensored log delay time is the log of observed delay time.

If at the boundary, the expectation of actual uncensored log delay time is the expected value of the uncensored log delay time given the subject waited the full 900 seconds. The uncensored delay times are assumed to be log normally distributed with mean equal to the empirical Bayes predicted log delay time for that subject and standard deviation equal to the idiosyncratic standard deviation of the log delay times. The expectation of uncensored delay given the subject is observed at the boundary are calculated in Stata by simulation. We generate normally distributed data (10,000 observations) using the empirical Bayes predicted uncensored log delay time for the subject and idiosyncratic standard deviation. The expected delay time given the subject is at the boundary is the mean of all observations in the simulated data above the boundary.

B. Bayesian data analysis

Fix any one of our dependent variables of interest y (e.g., permanent income). We estimate a multivariate linear regression model, $y_i = \boldsymbol{x_i}'\boldsymbol{\beta} + \varepsilon_i$, where $i = 1, \dots, N$ indexes individuals, $\varepsilon_i \sim_{\text{i.i.d.}} N(0, \eta^2)$, with η^2 known and $\boldsymbol{x_i}$ fixed. Let $x_{ij} \in \boldsymbol{x_i}$ be the key independent variable (either a self-control index or a measure of delay of gratification in childhood) and β_j be the corresponding coefficient in vector $\boldsymbol{\beta}$. We have a Gaussian prior on the population coefficient $\beta_j \sim N(\theta, \nu^2)$. Below, after deriving the posterior distribution for β_j, we discuss how we set values of θ and ν^2.

The OLS estimate for the coefficient vector is given by the usual formula $\widehat{\boldsymbol{\beta}} = (\boldsymbol{X}'\boldsymbol{X})^{-1}\boldsymbol{X}'\boldsymbol{y}$, where $\boldsymbol{y}$ and $\boldsymbol{X}$ denote, respectively, the vector of observed values of the dependent variable and the matrix of observed values of the independent variables across the N individuals. Define the matrix $\boldsymbol{\Sigma} \equiv \eta^2(\boldsymbol{X}'\boldsymbol{X})^{-1}$. As is well known, $\widehat{\boldsymbol{\beta}}|\boldsymbol{\beta}, \boldsymbol{\Sigma} \sim N(\boldsymbol{\beta}, \boldsymbol{\Sigma})$. Hence the conditional distribution of the jth element of $\widehat{\boldsymbol{\beta}}$, denoted $\hat{\beta}_j$, depends only on the jth element of $\boldsymbol{\beta}$, denoted β_j, and the jj^{th} entry of $\boldsymbol{\Sigma}$, denoted σ_j^2. Specifically, $\hat{\beta}_j|\beta_j, \sigma_j^2 \sim N(\beta_j, \sigma_j^2)$. It follows that the vector $(\hat{\beta}_j, \sigma_j^2)$ is a sufficient statistic for the posterior distribution of β_j (i.e., the posterior distribution of β_j depends on $(\boldsymbol{y}, \boldsymbol{X})$ only through $(\hat{\beta}_j, \sigma_j^2)$). We can therefore write the posterior distribution of β_j as $f(\beta_j|\hat{\beta}_j, \sigma_j^2)$, where we use $f(\cdot)$ to denote a probability density function.

The prior for β_j is

$$f(\beta_j) = \frac{1}{\sqrt{2\pi\nu^2}} e^{-\frac{1}{2\nu^2}(\beta_j - \theta)^2},$$

and the likelihood of observing $\hat{\beta}_j$, given the true population β_j and the observed $\boldsymbol{X}$, is

$$f(\hat{\beta}_j|\beta_j, \sigma_j^2) = \frac{1}{\sqrt{2\pi\sigma_j^2}} e^{-\frac{1}{2\sigma_j^2}(\hat{\beta}_j - \beta_j)^2}.$$

Standard calculations yield the posterior distribution,

$$\beta_j|\hat{\beta}_j, \sigma_j^2 \sim N\left(\frac{\nu^2}{\sigma_j^2 + \nu^2}\hat{\beta}_j + \frac{\sigma_j^2}{\sigma_j^2 + \nu^2}\theta, \frac{\sigma_j^2\nu^2}{\sigma_j^2 + \nu^2}\right).$$

In the last part of this section, we explain how we set the parameters of the prior distribution, θ and ν^2. Without loss of generality, orient all the dependent variables so that their expected correlation with the self-control independent variable is positive. Regarding θ:

- When the independent variable x_{ij} is *deviation in delay time measured in the marshmallow task*, we set θ such that the prior on the partial correlation between y_i and x_{ij} (controlling for the other independent variables in $\boldsymbol{x_i}$) has mean 0.05.
- When the independent variable is a *multi-year self-control index*, we set θ such that the prior on the partial correlation between y_i and x_{ij} has mean 0.15.

For all analyses, we will fix the standard deviation of the correlation at 0.2. See subsection C below for discussion of these prior distributions and related existing evidence.

We obtain the value of θ from the assumed partial correlation as follows. The partial correlation between y_i and x_{ij} is equivalent to the coefficient from estimating the same regression using standardized dependent and independent variables (i.e., subtracting the mean and dividing by the standard deviation, so that each resulting standardized variable has mean 0 and variance 1). If we represent this corresponding model with standardized variables as $\tilde{y}_i = \tilde{\boldsymbol{x}}_i{}'\tilde{\boldsymbol{\beta}} + \tilde{\varepsilon}_i$, then as noted, $\tilde{\beta}_j$ is equal to the partial correlation between y and x_j (hereafter we drop the *i* subscripts). Thus, our parameter of interest can be written $\beta_j = \tilde{\beta}_j \left(\frac{SD_y}{SD_{x_j}}\right)$, where SD_y and SD_{x_j} are the respective population standard deviations. We estimate these standard deviations using their sample counterparts.

We set θ as the value of β_j that would correspond to the mean of our prior on the correlation. For example in an analysis with a multi-year self-control index as the independent variable, we would set $\beta_j = 0.15\left(\frac{SD_y}{SD_{x_j}}\right)$, corresponding to a correlation of 0.15. Similarly, we set $\nu^2 = \left(0.2\left(\frac{SD_y}{SD_{x_j}}\right)\right)^2$. As noted above, ν^2 is held fixed when exploring the range of values for θ.

C. Discussion of priors for Bayesian analysis

1. Mean of the correlation between outcome measures and self-control measures

Recall that in our Bayesian data analysis (see Section VI and Subsection B above), we set the mean of our prior distribution such that it corresponds to a partial correlation between each outcome variable and the independent variable of…

- …0.05 when the independent variable is *deviation in delay time measured in the marshmallow task*.
- …0.15 when the independent variable is a *multi-year self-control index*.

For all three independent variables, we set the standard deviation of our prior distribution to correspond to a standard deviation of 0.20 for the partial correlation. In this section, we briefly discuss some of our reasoning and some of the related evidence that helped motivate these calibrations for the parameter values.

In the prior work most similar to our own, Moffitt et al (2011) explore correlations between a multi-year self-control index (a composite of behaviors measured from age 3 to 11) and several wealth outcomes measured at age 32. Controlling for gender, they find correlations with wealth outcomes are in the range of 0.15-0.27. Controlling for childhood socioeconomic status, IQ, and gender, they find correlations with wealth outcomes are in the range of 0.08-0.23. Moffitt et al use different measures of self-control and a different set of outcome variables so these findings are not fully analogous with our own analyses, but

their results provide a helpful point of reference and suggest that our prior mean 0.15 is perhaps slightly conservative.

In a meta-analysis, Richard, Bond, and Stokes-Zoota (2003, p.337) find that the average "person effect"—the estimated relationship between a social behavior and a demographic, personality, or other dispositional variable—across 16,282 estimated effects is 0.19. Compared to this benchmark, 0.15 again seems somewhat conservative.

For correlations between our outcomes and a measure of performance on the marshmallow delay task, we use as our benchmark prior an even lower correlation of 0.05. We hypothesize that behavior in the marshmallow delay task will have less explanatory power than the self-control indices as it is a single measure of self-control ability measured much earlier in life (and further from the outcomes) than the self-control indices.

2. Standard deviation of the correlation between outcome measures and self-control measures

Finally, we discuss how we set the standard deviation for our prior. We note that, unlike for the mean where a value closer to zero is clearly more conservative, it is ambiguous whether a larger or smaller standard deviation is more conservative: a larger standard deviation means that the prior distribution has less overall impact on the conclusions relative to the data, but a smaller deviation shrinks large estimated effects more toward the (conservative) mean of the prior distribution. We think that a standard deviation of 0.20 roughly captures our degree of uncertainty about the strength of the correlation for both independent variables. For example, it means that our approximate 95% confidence interval (mean ± 2 standard deviations) on the correlation between the multi-year self-control index and an outcome variable is (-0.25, 0.55). (By way of comparison, Richard, Bond, and Stokes-Zoota (2003, p.336) find that about 5% of published findings in social psychology report correlation coefficients larger than 0.50.)

D. False discovery rate control

Let $H_{01}, H_{02}, \dots, H_{0N}$ denote N the null hypotheses we want to test. Suppose that of these hypotheses, the null hypothesis is actually true in N_0 cases, and the non-null is true in $N_1 (= N - N_0)$ cases. We want to adopt a decision rule, a rule that uses the p-values to declare R of the hypotheses as significant and N - R as non-significant (e.g., the Bonferroni decision rule would be: reject H_{0i} if $p_i < 0.05/N$). Some of the hypotheses declared significant are actually null; let *a* denote the number of these. Some of the hypotheses declared significant are actually non-null; let *b* denote the number of these.

One approach to avoiding false positives is to adopt a decision rule that bounds the probability that *at least one* true null hypothesis is declared significant. This probability, $\Pr(a > 0)$, is called the family-wise error rate (FWER). The most well-known method of controlling the FWER is the Bonferroni decision rule. In our context, controlling the FWER would not be very informative because, given our relatively small sample size, our power to reject the family-wise null (that at least one of the null hypotheses is true) is very small.

The approach we focus on instead is to adopt a decision rule that bounds the (expected) proportion of true null hypotheses that are declared significant. This proportion, $\frac{a}{R}$, is called the false discovery rate (FDR). Just as a rejection threshold must be chosen in classical testing for a single null hypothesis (conventionally 0.05), controlling the FDR requires specifying a FDR threshold q (conventionally 0.10).

The most widely known method of controlling the FDR is Benjamini and Hochberg's (1995) algorithm. It proceeds as follows. Compute a p-value for each $i = 1, \dots, N$. Re-index the p-values in increasing order: $p_{(1)} \leq p_{(2)} \leq \dots \leq p_{(N)}$. Find the largest index i_{max} such that

$$p_{(i)} \leq \frac{i}{N} q.$$

The decision rule, denoted $BH(q)$, is: declare all hypotheses $i \leq i_{max}$ as significant and all hypotheses $i > i_{max}$ as non-significant.

Benjamini and Hochberg (1995) proved that if $p_1, \dots, p_N$ are mutually independent, then under $BH(q)$,

$$E\left(\frac{a}{R}\right) = \left(\frac{N_0}{N}\right) q \leq q.$$

In words, the expected proportion of false discoveries is at most q. In our application, because the life outcomes we study are correlated, the assumption of independent p-values across hypotheses almost surely does not hold. Below, we return to this point and explain why the decision rule $BH(q)$ typically performs well even when the independence assumption does not hold.

To help clarify that point, and to aid in interpreting the decision rule $BH(q)$, note that $BH(q)$ also has an empirical Bayes interpretation (Efron, Tibshirani, Storey, and Tusher, 2001). Let $F(p)$ denote the true (unknown) cdf of the p-values, and $F_0(p)$ denote the theoretical cdf of the p-values under the null hypothesis. Using Bayes' Rule,

$$\Pr\left(\text{hypothesis } (i) \text{ is actually null} \mid \text{its } p-\text{value } \leq p_{(i)}\right) = \frac{F_0\left(p_{(i)}\right) \Pr(\text{hypothesis } (i) \text{ is actually null})}{F(p_{(i)})}$$

Note that because each $p_{(i)} \sim U(0,1)$, we know that $F_0\left(p_{(i)}\right) = p_{(i)}$. We can estimate $F(p)$ by its empirical counterpart, $\bar{F}(p) \equiv \frac{\#\{p_i \leq p\}}{N}$, and note that (due to the assumption of independence across hypotheses) $\bar{F}\left(p_{(i)}\right) \equiv \frac{i}{N}$. We can replace $\Pr(\text{hypothesis } (i) \text{ is actually null})$ by its average value, $\frac{N_0}{N}$. Substituting,

$$\widehat{\Pr}\left(\text{hypothesis } (i) \text{ is actually null} \mid \text{its } p-\text{value } \leq p_{(i)}\right) = \left(\frac{N_0}{N}\right) \frac{p_{(i)}}{\left(\frac{i}{N}\right)}$$

If the threshold rule holds with equality at i_{max}, $p(i_{max}) = \frac{i_{max}}{N} q$, then

$$\widehat{\Pr}\left(\text{hypothesis } (i_{max}) \text{ is actually null} \mid \text{its } p-\text{value } \leq p_{(i_{max})}\right) = \left(\frac{N_0}{N}\right) q \leq q.$$

All hypotheses $i \leq i_{max}$ are weakly less likely than i_{max} to be null, given their p-values. Therefore, for all hypotheses with $p_{(i)} \leq p_{(i_{max})}$, $\widehat{\Pr}\big(\text{hypothesis } (i) \text{ is actually null} \mid p_{(i)} \leq p\big)$ is at most q. In words, under decision rule $BH(q)$, the probability that a hypothesis declared to be significant is actually null is at most q.

The empirical Bayes justification of the decision rule $BH(q)$ makes it easier to see why the independence assumption is not crucial. In the empirical Bayes argument, the independence assumption is used only in making the claim that $\bar{F}(p)$ is an unbiased estimate of $F(p)$. Efron (2010, Lemma 2.1 and p.55) shows that, in the absence of the independence assumption, $\bar{F}(p)$ is biased upward—and hence the decision rule $BH(q)$ is even more conservative. Moreover, Efron argues that in practice, the bias is small. The main cost of greater correlation in the *p*-values is that the estimated false discovery rate has greater sampling variation.

E. Multiple imputation of self-control scores for robustness checks

As a robustness check, we plan to test the sensitivity of our results to the imputation method used for missing self-control scores in 1984, 1993, and 2003.

To calculate a measure of aggregate self-control, we would like to (1) normalize the self-control scores in each year (1984, 1993, and 2003) using the inverse-normal transformation, (2) average the three scores, and (3) re-normalize the final variable to get an aggregate measure of self-control as measured in 1984, 1993, and 2003. To handle missing data in this robustness check, we perform an additional step between steps 1 and 2 to impute missing self-control scores in each year. All imputation analyses are limited to the 110 subjects with a self-control score for at least one year.

As a robustness check, we use multiple imputation, a technique that incorporates random error into the imputed value to reflect uncertainty. The general idea of multiple imputation is to generate $m > 1$ different imputed datasets, perform the analysis on each of the completed datasets (1 through m), and pool the results of the individual analyses to get a single coefficient and standard error that simultaneously accounts for uncertainty in the coefficient estimates and uncertainty in the imputed values. Like other imputation methods, multiple imputation assumes that the data are missing at random conditional on observed variables, i.e., that the probability that the data are missing does not depend on unobserved variables.

There are different ways to draw the random error that enters into multiple imputation. We adopt one standard approach, in which it is assumed that the variables—those with missing values (the self-control measures) and those used to predict the variables with missing values—are drawn from a multivariate normal distribution. We view this approach as natural in our context because the inverse-normal transformation that we apply to many of the variables in our analysis ensures that their marginal distributions are normal.

A key modeling decision is which variables to include as predictors of the self-control measures. Best practice is to include *all* variables that play into the ultimate analyses of interest, including the dependent variables (von Hippel 2007). This is because excluding variables from the imputation that will be

included in analyses (such as the dependent variables) can lead to coefficient estimates that are biased toward 0.[1] In our case, we specify a multivariate normal imputation model including the three self-control scores, deviation in delay time (both in log-seconds and seconds), gender, all primary outcome variables, and all secondary outcome variables with the exception of SAT scores. All variables are transformed using the inverse-normal transformation with the exception of gender. SAT scores are excluded from the imputation model because the small sample size (n = 31) results in parameter estimates that do not converge; consequently, analyses of correlations between imputed self-control scores and SAT scores may be biased toward 0. We include gender in the model even though it is binary because the multivariate normal imputation procedure has proven to be robust to some types of non-normality, including the presence of binary covariates (e.g., Lee & Carlin 2010). We do not believe inclusion of gender greatly impacts the model, either by model fit or for the prediction of self-control scores.

In the remainder of this section, we describe the multiple imputation procedure in more detail. For a complete treatment, see Little and Rubin (2002) and the Stata Multiple Imputation Manual.

1. More formal description of our multiple imputation procedure

Data augmentation (DA) is the term used to refer to a Bayesian approach to impute values assuming an underlying multivariate normal model. DA first draws a random imputation of missing data given assumed values of the parameters, and then draws new parameters from a Bayesian posterior distribution based on the observed data and values imputed in the previous step. This process is repeated, and the distribution of parameters converges to a posterior distribution of the parameters that averages over the missing data. The distribution of missing data converges to a predictive distribution.

To be more precise, let the data be $\mathbf{X} = (\mathbf{X_o}, \mathbf{X_m})$ where $\mathbf{X_o}$ is the observed part and $\mathbf{X_m}$ is the missing part from a normal distribution $\Pr(\mathbf{X}|\theta) = N(\beta, \Sigma)$**,** where θ are the unknown model parameters (conditional expectations β and elements of the covariance matrix Σ). The goal is to replace missing values with draws from the predictive distribution of the missing data given the observed data $\Pr(\boldsymbol{X_m}|\boldsymbol{X_o})$, which depends on the posterior distribution of the unknown parameters, $\Pr(\theta|\,\boldsymbol{X_o})$. DA augments the observed data with the latent unobserved missing data to estimate the conditional posterior distribution $\Pr(\theta|\,\boldsymbol{X_o}, \boldsymbol{X_m})$. For a current $\theta^{(t)}$, we draw $\boldsymbol{X_m^{(t+1)}}$ from its conditional predictive distribution, $\Pr\left(\boldsymbol{X_m}\middle|\boldsymbol{X_o}, \theta^{(t)}\right)$. Next, we draw $\theta^{(t+1)}$ from its conditional posterior distribution given the augmented data $\Pr\left(\theta\middle|\boldsymbol{X_o}, \boldsymbol{X_m^{(t+1)}}\right)$. This process is repeated until the specified number of imputations has been generated.

[1] It may be counterintuitive to include the dependent variables, but as von Hippel (2007) explains: "imputers sometimes worry that, by including Y in the imputation step, they are assuming something unwarranted about the X-Y relationship. This concern is misplaced. By including Y in the imputations, you are not assuming that Y has any particular relationship with X; the relationship could be positive, negative or zero, and any of these possibilities will be accounted for by the imputation model. On the other hand, if you exclude Y from the imputations, you are making an assumption. You are assuming that there is no direct relationship between X and Y."

To obtain starting values for this procedure, an expectation-maximization (EM) algorithm is used. EM is an iterative method that predicts missing values based on the assumed values for the parameters, uses the predictions to update the parameter estimates, and repeats the process until convergence to ML parameter estimates. For the EM algorithm, we assume a non-informative uniform prior distribution for the model parameters (β, Σ). EM convergence can be measured by the lack of change in the parameter estimates and the resulting log-likelihood. The number of iterations required for convergence is output directly from Stata.

A subset of the draws $\mathbf{X}_{\boldsymbol{m}}^{(\boldsymbol{t})}$ from the DA procedure are ultimately used as the imputed values. Multiple imputations begin after a specified "burn-in" period of b iterations. Because each iteration is dependent on the previous iteration, the values for each imputation are taken every *k* iterations, where *k* is the "burn-between" period. DA convergence is more difficult to measure than EM convergence but can be controlled by the burn-in and burn-between periods. The burn-in period, *b,* is set to obtain convergence to a stationary distribution $\Pr(\theta, \mathbf{X}_{\boldsymbol{m}} | \mathbf{X}_{\boldsymbol{o}})$ prior to recording imputed values. This is typically less that the number of iterations required by EM convergence, so a rule of thumb is to set *b* to a value greater than the reported iterations required for convergence of EM. The burn-between period, *k*, should be set large enough that the autocorrelation for all parameters has died down to 0 by lag *k*. We follow procedures described in the Stata Multiple Imputation manual to select values for *b* and *k*. Imputations are recorded at $t_i = b + (i - 1)k$, for $i = 1 - m$.

While more imputation draws is always better in principle, in practice there is a computational cost. Because our multiple imputation procedure is not very computationally intensive, we use *m* = 100, at the high end of values considered sufficient in the literature (Graham et al 2007).

2. Methodology after generating the imputed datasets

After imputing missing values, we re-transform each imputation of the self-control scores using the inverse-normal transformation. Then, we average the three transformed variables within each imputation to get an aggregate measure of self-control for each imputation run, 1 through *m*. Finally, we apply the inverse-normal transformation again to get the final self-control score for each individual in each imputation run.

Aggregating across simulation runs, pooled regression coefficients and standard errors are calculated according to Rubin's combination rules (Rubin 1987). In particular, the estimated coefficient is the average of the coefficients estimated in each imputed dataset. With *m* imputations,

$$\hat{\theta} = \frac{1}{m}\sum_{i=1}^{m} \hat{\theta}_i.$$

The variance of the estimate is

$$\mathrm{Var}(\hat{\theta}) = W + \left(1 + \frac{1}{m}\right) B,$$

where W is the mean within-imputation variance and B is the between-imputation variance:

$$W = \frac{1}{m}\sum_{i=1}^{m} W_i \text{ and } B = \frac{1}{m-1}\sum_{i=1}^{m}\left(\hat{\theta}_i - \hat{\theta}\right)^2.$$

XI. References

Ayduk, O., Mendoza-Denton, R., Mischel, W., Downey, G., Peake, P. K., & Rodriguez, M. (2000). "Regulating the interpersonal self: strategic self-regulation for coping with rejection sensitivity." *Journal of Personality and Social Psychology*, 79(5), 776.

Benjamini, Y, & Hochberg, Y. (1995). "Controlling the false discovery rate: A practical and powerful approach to multiple testing." *Journal of the Royal Statistical Society Series B*, 57(1), 289-300.

Bruno, G. (2004). *Limited Dependent Panel Data Models: A Comparative Analysis of Classical and Bayesian Inference among Econometric Packages.* No. 41. Society for Computational Economics.

Efron, B., Tibshirani, R., Storey, J.D., & Tusher, V. (2001). "Empirical Bayes analysis of a microarray experiment." *Journal of the American Statistical Association*, 96(456), 1151-1160.

Efron, B. (2010). *Large-Scale Inference: Empirical Bayes Methods for Estimation, Testing, and Prediction.* New York: Cambridge University Press.

Graham, J. W., Olchowski, A. E., & Gilreath, T. D. (2007). "How many imputations are really needed? Some practical clarifications of multiple imputation theory." *Prevention Science*, 8(3), 206-213.

Greene, W. (2008). *Econometric Analysis (6th Edition).* Upper Saddle River: Pearson Prentice Hall.

Kvam, P & Vidakovic B. (2007). *Nonparametric statistics with applications to science and engineering.* Hoboken, NJ: Wiley.

Lee, K. J., & Carlin, J. B. (2010). "Multiple imputation for missing data: fully conditional specification versus multivariate normal imputation." *American Journal of Epidemiology*, 171(5), 624-632.

Little, R. J. A., & Rubin, D. B. (2002). *Statistical Analysis with Missing Data*. 2nd ed. Hoboken, NJ: Wiley.

Moffitt, T. E., Arseneault, L., Belsky, D., Dickson, N., Hancox, R. J., Harrington, H., ... & Caspi, A. (2011). "A gradient of childhood self-control predicts health, wealth, and public safety." *Proceedings of the National Academy of Sciences*, 108(7), 2693-2698.

Nisbett, R. E. (1980). The trait construct in lay and professional psychology. In L. Festinger (Ed.), *Retrospections on social psychology*. (pp. 109–130). New York: Oxford University Press.

Rabe-Hesketh, S., Skrondal, A., & Pickles, A. (2004). "GLLAMM manual."

Rabe-Hesketh, S. (2004, Feb 29). Xttobit [The Stata Listserver]. Retrieved from http://www.stata.com/statalist/archive/2004-02/msg00789.html

Richard, F. D., Bond Jr, C. F., & Stokes-Zoota, J. J. (2003). "One Hundred Years of Social Psychology Quantitatively Described." *Review of General Psychology*, 7(4), 331.

Rubin, D.B. (1987). *Multiple Imputation for Nonresponse in Surveys*. J. Wiley & Sons, New York.

Schafer, J. L., & Olsen, M. K. (1998). "Multiple imputation for multivariate missing-data problems: A data analyst's perspective." *Multivariate behavioral research*, 33(4), 545-571.

Skrondal, A., & Rabe-Hesketh, S. (2004). *Generalized latent variable modeling: Multilevel, longitudinal, and structural equation models.* CRC Press.

StataCorp. (2013). Stata 13 Multiple Imputation Manual. College Station, TX: Stata Press.

StataCorp. (2013). Stata 13 XTTOBIT Manual. College Station, TX: Stata Press.

Vittinghoff, E., Glidden, D.V., Shiboski, S.C. & McCulloch, C.E. (2005) *Regression methods in biostatistics: linear, logistic, survival, and repeated measures models*. (pp. 75-76). New York, Springer.

Von Hippel, P. T. (2007). "Regression with missing Ys: An improved strategy for analyzing multiply imputed data." *Sociological Methodology*, 37(1), 83-117.
