# Supplementary material for: Predicting mid-life capital formation with pre-school delay of gratification and life-course measures of self-regulation
Source: J Econ Behav Organ. Author manuscript; Available in PMC 2021 Jan 8. (PMC7792663; doi:10.1016/j.jebo.2019.08.016)
Supplement: 5 [file NIHMS1543524-supplement-5.zip › Code/Readme.rtf]

This folder contains the code used to generate the findings in “Predicting mid-life capital formation with pre-school delay of gratification and life-course measures of self-regulation” by Daniel J. Benjamin, David Laibson, Walter Mischel, Philip K. Peake,  Yuichi Shoda, Alexandra Steiny Wellsjo, and Nicole L. Wilson.Data Availability:Data collection for the Bing Longitudinal Study now spans nearly 50 years. Due to a host of historical issues connected to the consenting procedures in this work and promises made to participants to protect both their privacy and the confidentiality of their responses, it has been a long-standing policy and IRB requirement that we not share the data connected with this work with outside parties or agencies. We believe it is imperative that we honor the promises made to our participants who have generously provided input to the project over the years. Stata do-files:Tobit Random Effects Analysis - uses the original Bing data to estimate a tobit random effects model and generate predicted delay times.Primary Analyses - this runs all of the primary analyses as described in the text (as well as select secondary analyses).Secondary Analyses - this runs all of the remaining secondary analyses described in the online appendix. MI Analysis - this runs the robustness analysis using a multiple imputation approach for filling in missing RNCCQ indices.Ex-Post Analyses - the runs all ex-post analyses. Avg Bootstrap - this constructs the average coefficient across all 11 outcomes and calculates bootstrapped standard errors.
